# Publisher Correction: Biosynthesis of strychnine

**DOI:** 10.1038/s41586-022-05177-z

**Published:** 2022-08-05

**Authors:** Benke Hong, Dagny Grzech, Lorenzo Caputi, Prashant Sonawane, Carlos E. Rodríguez López, Mohamed Omar Kamileen, Néstor J. Hernández Lozada, Veit Grabe, Sarah E. O’Connor

**Affiliations:** 1 Department of Natural Product Biosynthesis, Max-Planck Institute for Chemical Ecology, Jena, Germany; 2 Microscopic Imaging Service Group, Max-Planck Institute for Chemical Ecology, Jena, Germany

**Keywords:** Biochemistry, Plant sciences

Correction to: *Nature* 10.1038/s41586-022-04950-4Published online 6 July 2022

In the version of this article initially published, in the fourth paragraph of the main text, there was a typographical error in the first sentence, now reading in part, “…(see Supplementary Fig. 6 for the phylogenetic relationship of *C. roseus* and *S. nux-vomica*),” where Supplementary Fig. 7 was originally cited. The error has been corrected in the HTML and PDF versions of the article.

