# Supplementary material for: Biosynthesis of strychnine
Source: Nature. 2022 Jul 6;607(7919):617–22. doi: 10.1038/s41586-022-04950-4 (PMC9300463; doi:10.1038/s41586-022-04950-4)
Supplement: Supplementary file 1 — This file contains Supplementary Methods, Supplementary Figs. 1–30, details regarding the synthesis of the compounds, NMR spectra data and Supplementary References. [file 41586_2022_4950_MOESM1_ESM.pdf]

nature portfolio
https://doi.org/10.1038/s41586-022-04950-4

Supplementary information

Biosynthesis of strychnine

In the format provided by the authors and unedited

Supplementary information for

Biosynthesis of Strychnine

Benke Hong,[1] Dagny Grzech,[1] Lorenzo Caputi,[1] Prashant Sonawane,[1] Carlos E. Rodríguez López,[1] Mohamed Omar Kamileen,[1] Néstor J. Hernández Lozada,[1] Veit Grabe,[2] Sarah E. O'Connor[1]§

[1] Max-Planck Institute for Chemical Ecology, Department of Natural Product Biosynthesis; Hans-Knoll Strasse 8, 07745, Jena, Germany.
[2] Max-Planck Institute for Chemical Ecology, Microscopic Imaging Service Group; Hans-Knoll Strasse 8, 07745, Jena, Germany.

§Corresponding author. Email: oconnor@ice.mpg.de

Table of Contents

1. Materials and Methods **1**

2. Supplementary Figures **12**

Fig. 1. Brief timeline of strychnine research 12

Fig. 2. Proposed biosynthetic pathway for diboline, strychnine, and brucine 12

Fig. 3. Atom numbering and ring annotation of strychnine and brucine 12

Fig. 4. Metabolic analysis of *Strychnos nux-vomica*. 13

Fig. 5. Metabolic analysis of *Strychnos sp.* 14

Fig. 6. Phylogenetic relationship of *Strychnos* and *Catharanthus roseus* 14

Fig. 7. Candidate genes for the biosynthesis of geissoschizine **1** in *S. nux-vomica*. 15

Fig. 8. Phylogenetic tree of *Snv*GO (Cluster-4032.29856) with previously characterized CYPs from other organisms. 16

Fig. 9. Functional characterization of *Snv*WS with **4** and **5**. 17

Fig. 10. Mechanistic hypothesis for *Snv*WS 19

Fig. 11. Kinetic characterization of *Snv*WS with **4** and **5** 20

Fig. 12. *Snv*AT and *Sp*AT are targeted to the cytoplasm 21

Fig. 13. Derivatization of **9** 22

Fig. 14. Phylogenetic tree of *Snv*AT and *Sp*AT with previously characterized BAHD acyltransferases 23

Fig. 15. Protein model of *Snv*AT and *Sp*AT 24

Fig. 16. Site directed mutagenesis of *Sp*AT 25

Fig. 17. Site directed mutagenesis of *Snv*AT 26

Fig. 18. Docking model of *Snv*AT with malonyl-CoA 27

Fig. 19. Comparison of *in vivo* and *in vitro* reactivity of *Sp*AT and *Snv*AT under various conditions 28

Fig. 20. *In vitro* reactivity of *Sp*AT at various pH 30

Fig. 21. *In vitro* reactivity of *Snv*AT at various pH 31

Fig. 22. Time course of strychnine and isostrychnine accumulation 32

Fig. 23. *In vitro* assays of prestrychnine. 33

Fig. 24. Proposed mechanism for the strychnine **10** and isostrychnine **11** formation. 34

Fig. 25. Phylogenetic tree of cytochromes P450 that highly expressed (FPKM ≥ 20) in the root of *S. nux-vomica* with previously characterized CYPs from other organisms.. 35

Fig. 26. Functional characterization of *Snv*OMT with 11-OH strychnine **16**. 36

Fig. 27. Functional characterization of *Snv*OMT with 10-deMe brucine **18**. 37

Fig. 28. Reconstitution of brucine **15** pathway in *N. benthamiana* from geissoschizine **1**... 38

Fig. 29. Average LC-MS peak area (three biological replicates) of products produced in *N. benthamiana* after expression of indicated enzymes and substrates infiltration... 39

Fig. 30. Brief summary of the reported method for synthesis of (±)-geissoschizine 40

3. Synthesis of compounds **40**

4. NMR spectra **47**

5. References **84**

6. Supplementary Tables

Supplementary Table 1. Co-expression analysis of root highly expressed (FPKM ≥ 20) P450s in *S. nux-vomica* genes with *Snv*GO

Supplementary Table 2. Root highly expressed methyltransferases (FPKM ≥ 20) in *S. nux-vomica*

Supplementary Table 3. List of primers used in this study

Materials and Methods

Plant materials

Strychnos nux-vomica and *Strychnos sp.* plant samples were collected at the Jena Botanical Garden and Bonn University Botanic Garden.

Chemicals

All solvents used for extractions, chemical synthesis and preparative HPLC were of HPLC grade, whilst solvents for UPLC/MS analysis were of MS grade. All solvents were purchased from Fisher Scientific. Strychnine **10** and brucine **15** standards were purchased from Sigma-Aldrich. Synthesis of compounds that are not commercially available is described in **Synthesis of compounds** section.

Carbenicillin and gentamycin were purchased from Formedium. Rifampicin was from Sigma Aldrich. Spectinomycin was from Fisher Scientific.

Molecular biology kits

All gene and fragment amplifications were performed using Platinum™ Superfi™ polymerase (Thermo Fisher) or Phusion™ High-Fidelity DNA polymerase (New England Biolabs) whilst colony PCR reactions were performed using Phire II master mix (Thermo Fisher). All primers were purchased from Sigma Aldrich. PCR product purifications were performed using the Zymo Research PCR clean-up kit. Plasmid purifications were performed using Promega Wizard miniprep kits. cDNA was prepared using Superscript™ IV VILO™ master mix and TURBO™ DNase (Thermo Fisher). All restriction enzymes were purchased from NEB.

RNA purification, sequencing and transcriptome analysis

Total RNA was extracted using the RNeasy Plant Mini Kit (Qiagen) from the leaf, stem, and root of *Strychnos* plants (2 biological replicates each) according to the manufacturer's instructions. RNA quality was assessed by Nanodrop. Samples with RNA concentration ranging from 100-500 ng/µL and acceptable quality were sent to Novogene (https://en.novogene.com), where mRNA library preparation and RNA-seq analysis were performed according to the service provider's standard protocol.

RNA integrity and quantitation were assessed using the RNA Nano 6000 Assay Kit of the Bioanalyzer 2100 system (Agilent). A total amount of 1 µg RNA per sample was used as input material for the RNA sample preparations. Sequencing libraries were generated using NEBNext Ultra™ RNA Library Prep Kit for Illumina® (NEB, USA) following manufacturer's recommendations. The library preparations were sequenced on NovaSeq PE150 platform (Illumina). Clean data (clean reads) were obtained by trimming reads containing adapter and removing poly-N sequences and reads with low quality from raw data. At the same time, Q20, Q30 and GC content of the clean data were calculated. All the downstream analyses were based on the clean data with high quality. Trinity v.2.6.6 is used to perform the transcriptome assembly. After that, we use CORSET v.4.6 software to remove the redundance from Trinity results. Gene expression levels were estimated by RSEM v.1.2.28 and differential expression analysis using DESeq2 v.1.26.0. CDS prediction can be divided into two steps: (1) BLAST unigene according to the priority of NR and Swissprot databases. If the information matched, candidate coding regions prediction can be divided into two steps: (1) BLAST unigene according to the priority of NR and Swissprot databases. If the

information matched, CDS is extracted from unigene sequences and translated into peptide sequences based on the standard codon table (from 5' to 3'); (2) Unigenes with no hits in BLAST are analyzed with ESTScan to predict their coding regions and determine their sequence direction. To achieve comprehensive gene functional annotation, four databases are applied. The software and parameters used in databases are as follows: NT: NCBI BLAST, the evalue threshold is 1e-5; NR, SwissProt: Diamond. For NR and SwissProt databases, the evalue threshold is 1e-5; PFAM, hmmscan, the evalue threshold is 0.01.

Coexpression analysis

Coexpression analysis was done using CoExpNetViz software[35]. For *S. nux-vomica*, genes highly expressed in root (FPKM ≥ 20 in two biological replicates) were selected for coexpression analysis. Geissoschizine oxidase (*Snv*GO) was used as 'bait' with correlation threshold set at 5 and 95 for lower and higher percentile rank, respectively. Lists of coexpressed genes were additionally filtered according to protein family and Pearson's correlation coefficient ($r \geq 0.95$). For *Strychnos sp.*, genes expressed in root (FPKM ≥ 5 in two biological replicates) were selected for coexpression analysis. geissoschizine oxidase (*Sp*GO), norfluorocurarine synthase 1 (*Sp*NS1), norfluorocurarine synthase 2 (*Sp*NS2), norfluorocurarine oxidase (*Sp*NO), and Wieland-Gumlich aldehyde synthase (*Sp*WS) were used as 'baits' in coexpression analysis using the same parameters. Lists of coexpressed genes were additionally filtered according to Pearson's correlation coefficient ($r > 0.6$) for each bait. The analysis was performed using *S. nux-vomica and Strychnos sp.* RNA-Seq transcriptome data from different tissues. The coexpression network was visualized with the Cytoscape (v.3.9.0).[36]

LC-MS analysis

Method 1

Samples were analyzed using an Elute LC system (Bruker Daltonik, Bremen, Germany) coupled via electrospray ionization (ESI) to a Maxis II quadrupole-time-of-flight (qTOF) MS (Bruker Daltonik; Bremen, Germany). LC-MS data was collected by Bruker otofControl 5.2.109/Hystar 5.1.5.1. Ionization was performed via pneumatic-assisted electrospray ionization in positive mode (ESI+) with a capillary voltage of 3500 V and an end plate offset of 500 V; a nebulizer pressure of 2.5 bar was used, with nitrogen at 250°C and a flow of 11 L/min as the drying gas. Acquisition was done at 12 Hz following a mass range from 80 to 1000 *m/z*, with data dependent MS/MS and an active exclusion window of 0.2 min, a reconsideration threshold of 1.8-fold change. Fragmentation was triggered on an absolute threshold of 400 and limited to a total cycle time range of 0.5 s. For collision energy, the stepping option model (from 20 to 50 eV) was used. At the beginning of each run, an injection of a sodium formate-isopropanol solution was performed and the m/z values were re-calibrated using the expected cluster ion *m/z* values. At the beginning of each run, the first 1 min of LC input was redirected to waste and was run for 1 min with 5% acetonitrile isocratically. Samples were run in at a flowrate of 0.6 mL/min in a formic acid 0.1% - acetonitrile (plus 0.1% formic acid) gradient linearly increasing from 5% to 15% acetonitrile in 5 minutes (from minute 1 to 6) using analytical Phenomenex Kinetex XB-C18 column (100 x 2.1mm, 2.6 µm; 100 Å; column temperature 40°C). 2 µL of samples were injected. This method was used to analyze all samples except geissoschizine **1,** akuammicine **3,** and desoxy Wieland-Gumlich aldehyde **7**.

Method 2

As with Method 1, at the beginning of each run, the first 1 min of LC input was redirected to waste and was run for 1 min with 10% acetonitrile isocratically. Samples were run at a flowrate of 0.6 mL/min in a formic acid 0.1% - acetonitrile (plus 0.1% formic acid) gradient linearly increasing from 10% to 30% acetonitrile in 5 minutes (from minute 1 to 6) using analytical Phenomenex Kinetex XB-C18 column (100 x 2.1mm, 2.6 µm; 100 Å; column temperature 40°C). 2 µL of samples were injected. This method was used to analyze akuammicine **3**.

Method 3

As with Method 1, at the beginning of each run, the first 1 min of LC input was redirected to waste and was runed for 1 min with 15% acetonitrile isocratically. Samples were run at a flowrate of 0.6 mL/min in an ammonia 0.025%- acetonitrile gradient linearly increasing from 15% to 50% acetonitrile in 4 minutes (from minute 1 to 5) using analytical Waters Acquity UPLC BEH-C18 column (2.1 x 50 mm; 1.7 µm; column temperature 40°C). 2 µL of samples were injected. This method was used to analyze geissoschizine **1** and desoxy Wieland-Gumlich aldehyde **7**.

Note: The chromatograms shown in each panel are all on the same y-axis. When abundance of the compound is too low, a sub-panel that is zoomed in is shown.

Metabolite analysis of *Strychnos nux-vomica* and *Strychnos sp.*

The same plant material used for RNA analysis was also subjected to metabolomic analysis. Plant material (leaf, stem, and root) was snap frozen in liquid nitrogen, ground with mortar and pestle and extracted with MeOH and sonicated for 15 min at room temperature. The volume of MeOH was normalized on fresh tissue weight (1:100 = mg:µL). Plant material was centrifuged at 16000*g* for 10 minutes and the supernatant filtered with Fisher polytetrafluoroethylene (PTFE) syringe filters (0.22 µm). The resulting methanolic extracts were diluted 1:10 in MeOH and analyzed by untargeted UPLC/MS (Method 1). Total ion chromatograms are shown for each tissue with standards.

Candidate gene cloning

The full-length sequences of the genes of interest were amplified from cDNA using the primers listed in Supplementary Table 3 by using Phusion High-Fidelity DNA Polymerase (New England Biolabs) or Platinum Superfi polymerase (Thermo Fisher) according to the manufacturer's instructions. For transient expression in *N. benthamiana*, the PCR products were purified from agarose gel and ligated into the modified 3Ω1 vector[37] (digested with BsaI) using the In-Fusion kit (Clontech Takara).This modified 3Ω1 vector contains CCDB resistance marker instead of LacZ (presents in normal 3Ω1 vector), which enables easy selection of transformations and reduce false positive background. In-Fusion reactions were transformed into competent *E. coli* Stellar cells (Clontech Takara). Recombinant colonies were selected on LB agar plates supplemented with spectinomycin (100 µg/mL). Positive clones were identified by colony PCR using sequencing primers designed for the specific vector used (Supplementary Table 3). Plasmids were isolated from positive colonies grown overnight. Identities of the inserted sequences were confirmed by Sanger sequencing. The constructs were then used to transform electrocompetent *Agrobacterium tumefaciens* strain GV3101 by electroporation. Recombinant colonies were selected on LB agar containing rifampicin (50 µg/mL), gentamycin (50 µg/mL) and spectinomycin (250 µg/mL). Single colonies were grown in 10 mL of LB (50 µg/mL rifampicin, 50 µg/mL gentamycin, and 250 µg/mL

spectinomycin) at 28 °C for 1 day, glycerol stocks (50% glycerol and 1 mL culture) were prepared and stored at −80 °C for future use.

For heterologous expression in SoluBL21 *E. coli,* coding sequence (CDS) of *Snv*WS, *Sp*AT, *Snv*AT and *Snv*OMT were amplified from 3Ω1 constructs with the primers listed in Supplementary Table 3. The PCR products were purified from agarose gel and ligated into the pOPINF vector[38] (digested with HindIII and KpnI) using the In-Fusion kit (Clontech Takara). Infusion reactions were transformed into competent E. coli Stellar cells (Clontech Takara). Recombinant colonies were selected on LB agar plates supplemented with carbenicillin (100 μg/mL). Positive clones were identified by colony PCR using sequencing primers designed for the specific vector used (Supplementary Table 3). Plasmids were isolated from positive colonies grown overnight. Identities of the inserted sequences were confirmed by Sanger sequencing.

5’ RACE for *Sp*AT and *Snv*AT

*Sp*AT and *Snv*AT missing 5’ end in transcriptome. The full length sequences were obtained by Takara SMARTer® RACE 5'/3' Kit. 5’-RACE-Ready cDNA were prepared and 5’ end of *Sp*AT and *Snv*AT were amplified from cDNA with this kit using the primers listed in Supplementary Table 3 according to the manufacturer’s instructions. The PCR products were purified from agarose gel and ligated into the pOPINF vector (digested with HindIII and KpnI) using the In-Fusion kit (Clontech Takara). In-Fusion reactions were transformed into competent *E. coli* Stellar cells (Clontech Takara). Recombinant colonies were selected on LB agar plates supplemented with carbenicillin (100 μg/mL). Positive clones were identified by colony PCR using sequencing primers designed for the specific vector used (Supplementary Table 3). Plasmids were isolated from positive colonies grown overnight. Identities of the inserted sequences were confirmed by Sanger sequencing.

Transient expression of candidate genes in *N. benthamiana*

Agrobacterium strains that contained the gene constructs were grown in 10 mL of LB with antibiotics (50 μg/mL rifampicin, 50 μg/mL gentamycin, and 250 μg/mL spectinomycin) for 16 h at 28 °C, then the cells were centrifuged at 2000*g* for 20 min and the supernatant was removed. The cell pellet was re-suspended in 5 mL infiltration buffer (50 mM MES, 2 mM Na_3PO_4, 27.8 mM glucose, 10 mM $MgCl_2$ and 100 μM acetosyringone) and centrifuged at 2000*g* for 10 min. The supernatant was removed and the cell pellet was re-suspended in 10 mL infiltration buffer. For individually tested strains, *Agrobacterium* suspensions were diluted to an optical density OD_{600} 0.6. When multiple constructs were infiltrated simultaneously, the corresponding *A. tumefaciens* cell cultures were mixed so that the final OD_{600} of each would be 0.4. After incubation at room temperature for 2 h, infiltration was performed using a 1 mL syringe without needle on underside side of 4-5 week-old *N. benthamiana* leaves. After 3 days, 100 μM substrates in water with 1 % DMSO was infiltrated into the underside side of previously *Agrobacterium*-infiltrated leaves with a needleless 1-ml syringe. For co-infiltration of geissoschzine **1** and disodium malonate as substrates, 200 μM (±)-geissochizne and 4 mM disodium malonate in water with 1% DMSO was used. Leaves were harvested 2 days post-infiltration. Each experiment was tested 3 times. Biological replicates consisted of several leaves all from different tobacco plants.The second pair of fully expanded leaves (counting from the apical meristem side) in each plant were used for experiment.

Crude protein extracts from *N. benthamiana*

Wild type *N. benthamiana* leaves was used to prepare crude protein extracts. Tissue were snap frozen in liquid nitrogen and was homogenized using a mortar and pestle with liquid N_2 and a small scoop of polyvinylpolypyrrolidone (PVPP). Extract the powdered tissue with ice-cold extraction buffer at 5 mL buffer per 1 g of powered tissue. Incubate the mixture in a cold room (4 ^{o}C) for 30 min with periodic gentle inversions. Spin sown extracted homogenate at 4000g for 10 min at 4 ^{o}C to pellet plant cell debris. Filter supernatant through Miracloth (Merck-Millipore) and collect flow through in a pre-cooled tube. Centrifuge the filtered extract at 35000g for 30 min at 4 ^{o}C. The supernatant was removed and aliquoted into prechilled microfuge tubes, which were then snap frozen in liquid N_2 and stored at –80 °C. This crude protein lysate was used as the bulk buffer for *in vitro* reactions. Extraction buffer was used as negative controls.
Extraction buffer: 50 mM Tris-HCl pH 7.4, 50 mM glycine, 5% glycerol, 0.5 M NaCl, 1 mM phenylmethylsulfonyl fluoride, EDTA free protease inhibitor cocktail (1 tablet in 50 mL).

In vitro assays with *N. benthamiana* leaf disk

*Sp*AT and *Snv*AT were tested using *N. benthamiana* leaf disk method. Leaves were infiltrated with *Agrobacterium* strains that contained the gene construct of interests were used to harvest leaf disks. At 3 days post *Agrobacterium* infiltration, leaf disk were collected using a 10 mm hole puncher and placed into a 48-well plate (10 mm diameter well) with cover containing 200 µL HEPES buffer (50 mM, pH 7.5). For *Sp*AT, Wieland-Gumlich aldehhyde (2 µL, 1 mM in Methanol) was added into the well containing the leaf disk. For *Snv*AT and AAE13, Wieland-Gumlich aldehhyde (2 µL, 1 mM in Methanol) and disodium malonate (2 mM, final concentration) was added into the well containing the leaf disk, then sealed with the provided cover and wrapped with parafilm to avoid excessive evaporation of buffer. The plate was then incubated in a growth chamber (16 hours light, 8 hours dark 22-25 °C, 40-65% relative humidity) overnight (16 h). Then place the disk in 2 mL Eppendorf tube and flash freeze in liquid nitrogen. Tissue was ground to a fine powder using a Qiagen Tissue homogenizer with 2-mm-diameter stainless steel beads at 25 Hz for 2 min. MeOH (100 µL per disk) was added and were sonicated for 15 min at room temperature, centrifuged at 15,000 rpm for 5 min to pellet plant debris, and the remaining solvent was filtered through 0.22-µm PTFE filters before analysis by LC–MS (Method 1).

Sample harvest and derivatizations

Harvest

Leaves from *N. benthamiana* transient expression experiments were weighed and snap frozen in liquid N_2 and homogenized on a TissueLyser II (Qiagen) using 2-mm-diameter stainless steel beads, with shaking at 25 Hz for 2 min. MeOH (5 µl per mg) was added and were sonicated for 15 min at room temperature, centrifuged at 15,000 rpm for 5 min to pellet plant debris, and the remaining solvent was filtered through 0.22-µm PTFE filters before analysis by high-resolution LC–MS. Metabolites were identified by comparing the retention times and mass fragments of standard compounds.

Derivatizations

Trimethylsilyldiazomethane (TMSD)[39]
Methanolic extracts of *N. benthamiana* leaves that produced **9** were prepared using aforementioned method. To 100 µL methanolic extracts were added 100 µL TMSD (0.6 M in hexane). After incubating for 1 h at room temperature, dried under Ar_2 flow, and resuspended in 100 µL MeOH for LC-MS analysis.

Derivatized product was identified by comparing the retention time and mass fragments with the synthetic standard compound.

Trimethylsilyldiazomethane (TMSD) and sodium borohydride ($NaBH_4$)
To 100 µL methanolic extracts were added 1 µL formic acid and 100 µL TMSD (0.6 M in hexane). After incubating for 1 h at room temperature, 100 µL $NaBH_4$ (0.2 M in MeOH) was added and further incubated for 1 h, dried under Ar_2 flow, and resuspended in 100 µL MeOH for LC-MS analysis. Derivatized product was identified by comparing the retention times and mass fragments of standard compound.

Feeding experiment

Deuterium labeled Wieland-Gumlich aldehyde (20 mg) was dissolved in 1 mL MeOH and 200 µL 1 M HCl was added, the solvents were removed under Ar_2 flow. The resulting HCl salt was resuspended in 50 mL deionized water and the pH adjusted to 6.5 with saturated sodium bicarbonate. Then more water was added to a final concentration of 1 mM. Three *S. nux-vomica* (4-month-old) were taken from the pot and washed with deionized water to remove the soil. The roots were submerged in 20 mL 1 mM Wieland-Gumlich aldehyde solution in a 50 mL falcon tube. The roots were submerged in deionized water as negative controls. The plants were put in the growth chamber (12 h light, 30 ^{o}C day temperature, 24 ^{o}C night temperature, 70% humidity). Metabolite analysis of the roots was performed at the 3rd day, 7th day and 14th day using aforementioned metabolites analysis method.

Protein expression and purification

Genes cloned into pOPINF vectors (*Snv*WS, *Snv*AT, *Sp*AT and *Snv*OMT) were transformed into SoluBL21 *E. coli* strain for protein expression and carbenicillin (100 µg/mL) was used for selection. Single colonies grown in LB agar media were picked and grown overnight in 10 mL LB media at 37 °C. The next day, 1 mL of the overnight cultures were used to inoculate 100 mL of 2xYT media. The fresh cultures were grown at 37 °C until OD_{600} of 0.6-0.8 was reached, followed by induction with 250 µM IPTG and incubation at 18 °C for 16-18 h. For purification, the cells were harvested by centrifugation (10 min at 3200*g*), resuspended in 10 mL of binding buffer (50 mM tris-HCl pH 8, 50 mM glycine, 500 mM sodium chloride, 20 mM imidazole, 5% v/v glycerol, pH 8) containing in addition 0.2 mg/mL Lysozyme and EDTA free protease inhibitor cocktail (Roche cOmplete^{TM}) and incubated for 30 min on ice. Cells were lysed by sonication using a Sonics Vibra Cell at 40% amplitude, 3s ON, 2s OFF, 4 min total. The crude lysates were centrifuged at 35,000*g* for 15 min and the cleared lysates were incubated with 250 µL Ni-NTA agarose beads (Qiagen) for 30 min at 4 °C. Next, the beads were sedimented by centrifugation at 1000*g* for 2 min and washed 3 times with binding buffer before eluting the proteins with elution buffer (50 mM tris-HCl pH 8, 50 mM glycine, 500 mM Sodium Chloride, 250 mM imidazole, 5% v/v glycerol, pH 8). Dialysis and buffer exchange was performed using Buffer A4 (20 mM HEPES pH 7.5; 150 mM NaCl) in centrifugal concentrators with size exclusion of 10 or 30 KDa depending on the protein size. Proteins were aliquoted in 30 µL, snap-frozen and stored at -80 ^{o}C.

Enzymatic assays

In vitro assays of *Snv*WS

*Snv*WS was tested using norfluorocurarine **4** and 18-OH norfluorocurarine **5** as substrates. Reaction mixtures consisted of *Snv*WS (1 µM) in a total volume of 100 µL HEPES pH 7.5 (50 mM); substrate (50 µM), NADPH (500 µM). Reactions were incubated at 30 ^{o}C for 12 hours. Negative controls consisted of boiled

*Snv*WS (90 ^{o}C, 10 min) in the reaction mixture. After incubation, the reactions were quenched by addition of 1 volume of MeOH, filtered through 0.22-µm PTFE filters and analyzed by untargeted LC/MS (Method 1 and Method 3).

Kinetic analysis of *Snv*WS using norfluorocurarine 4 as a substrate

For steady state enzyme kinetics, enzyme assays (100 µl reaction volume, 3 replicates) contained 1 µM of purified *Snv*WS, 200 µM NADPH, norfluorocurarine **4** at various concentrations (2, 5, 8, 10, 20, 30, 40, and 50 µM) and 50 mM HEPES buffer (pH 7.5). Reactions were initiated by the addition of enzyme and incubated at 30°C. Reaction were quenched by the addition of 1 volume of MeOH at 10 min (less than 10% substrate consumption) and filtered through 0.22-µm PTFE filters. Samples were analyzed by reversed-phase chromatography on an Dionex UltiMate 3000 UHPLC (Thermo Scientific) with an Bruker EVOQ Elite electrospray ionization using analytical Phenomenex Kinetex XB-C18 column (100 x 2.1mm, 2.6 µm; 100 Å; column temperature 40°C). Water with 0.1% TFA (A) and ACN (B) were used as the mobile phase components at a flow rate of 0.6 mL/min with the following gradient: 0-0.5 min, 5% B; 0.5-3.5 min, 5-35% B; 3.5-4.0 min, 35% B; 4.0-4.1 min, 35-5% B; 4.1-5.5 min, 5% B. The reaction rate was assessed via the consumption of **4**, which was quantified by generating a standard curve with standard of **4**. Data fitting was performed using GraphPad Prism 8.0.2. K_M, k_{cat} and k_{cat}/K_M represent the mean of three independent replicates.

Kinetic analysis of *Snv*WS using 18-OH norfluorocurarine 5 as a substrate

A similar procedure as that above was followed to determine the enzymatic kinetics of *Snv*WS using varying concentrations of 18-OH norfluorocurarine **5**. Enzyme assays (100 µl reaction volume, 3 replicates) contained 500 nM of purified *Snv*WS, 200 µM NADPH, 18-OH norfluorocurarine **5** at various concentrations (2, 5, 8, 10, 20, 30, 40, and 50 µM) and 50 mM HEPES buffer (pH 7.5). Reactions were initiated by the addition of enzyme and incubated at 30°C. Reaction were quenched by the addition of 1 volume of MeOH at 5 min (less than 10% substrate consumption) and filtered through 0.22-µm PTFE filters and analyzed by same method as above. The reaction rate was assessed via the consumption of **5**, which was quantified by generating a standard curve with standard of **5**. Data fitting was performed using GraphPad Prism 8.0.2. K_M, k_{cat} and k_{cat}/K_M values represent the mean of three independent replicates.

***In vitro* assays of *Snv*AT and *Sp*AT**

*Snv*AT and *Sp*AT were tested using Wieland-Gumlich aldehyde **6** as substrate in various conditions. Assays were performed in a total volume of 100 µL indicated buffer (pH 5.5 to 9.5) and concentration (5 mM or 50 mM), substrate (50 µM); acetyl-CoA or malonyl-CoA (50 µM or 500 µM) and enzyme (1 µM). Reactions were incubated at 25 ^{o}C or 37 ^{o}C for 12 hours. After incubation, the reactions were quenched by addition of 1 volume of MeOH, filtered through 0.22-µm PTFE filters and analyzed by LC-MS (Method 1).

***In vitro* assays of *Snv*WS with *Snv*AT and *Snv*WS with *Sp*AT**

*Snv*WS with *Snv*AT and *Snv*WS with *Sp*AT were coupled *in vitro* assays using 18-OH norfluorocurarine **5** as substrate.

*Snv*WS with *Snv*AT: Assays were performed in a total volume of 100 µL HEPES pH 7.5 (50 mM); **5** (50 µM); NADPH (500 µM); Malonyl-CoA (500 µM); and *Snv*WS (1 µM) and *Snv*AT (1 µM). Reactions were incubated at 37 ^{o}C for 12 hours. After incubation, the reactions were quenched by addition of 1 volume of MeOH, filtered through 0.22-µm PTFE filters and analyzed by LC-MS (Method 1).

*Snv*WS with *Sp*AT: Assays were performed in a total volume of 100 µL HEPES pH 7.5 (50 mM); **5** (50 µM); NADPH (500 µM); acetyl-CoA (500 µM); and *Snv*WS (1 µM) and *Sp*AT (1 µM). Reactions were incubated at 37 °C for 12 hours. After incubation, the reactions were quenched by addition of 1 volume of MeOH, filtered through 0.22-µm PTFE filters and analyzed by LC-MS (Method 1).

***In vitro* assays of prestrychnine**

Methanolic extracts from *N. benthamiana* leaves which produce prestrychnine **9** was incubated with *N. benthamiana* crude protein extracts and recombinant *Snv*AT. For *N. benthamiana* crude protein extracts, prestrychnine (10 µL) was incubated with *N. benthamiana* crude protein extracts (90 µL) at 37 °C for 24 h. Extraction buffer (90 µL) was used as negative controls in the reaction mixtures.
For *Snv*AT, assays consisted of *Snv*AT (1 µM) in a total volume of 100 µL buffer (50 mM HEPES pH 7.5 or 50 mM MES pH 5.5); prestrychnine (10 µL). Reactions were incubated at 37 °C for 24 hours. Boiled *Snv*AT (90 °C, 10 min) was used as controls in the reaction mixture. After incubation, the reactions were quenched by addition of 1 volume of MeOH, filtered through 0.22-µm PTFE filters and analyzed by LC-MS (Method 1).

***In vitro* assays of *Snv*OMT**

*Snv*OMT was tested using 10-OH strychnine **12**, 11-OH strychnine **16**, 11-deMe brucine **14** and 10-deMe brucine **18** as substrates. Assays were performed in 100 µL volume of 50 mM HEPES pH 7.5; substrate (50 µM); SAM (200 µM); and *Snv*OMT (1 µM). Negative controls consisted of boiled *Snv*OMT (90 °C, 10 min) in the reaction mixture. Reactions were incubated at 30 °C for 12 hours. After incubation, the reactions were quenched by addition of 1 volume of MeOH, filtered through 0.22-µm PTFE filters and analyzed by LC-MS (Method 1).

Phylogenetic tree

The phylogenetic tree was inferred with the Maximum Likelihood method and Poisson correction model.[40] The tree was constructed in MEGAX software[41] using the Bootstrap test with 1000 replications[42], and a discrete Gamma distribution (5 categories). The phylogenetic tree was visualized with iTOL.[43]

Protein crystal modeling

Homology models of *Snv*AT and *Sp*AT were built using the Swiss-Model server.[44] PDB 6LPV was used as a template.[29] The model was visualized with PyMOL.

*Snv*WS model created using Swiss-Model server[44] based on the structure of *C. roseus* heteroyohimbine synthase THAS2 (PDB 5H81)[23]. Model quality as measured by QMEANDisCo Global score[45] was 0.83 ± 0.05. Molecular docking of 18-OH norfluorocurarine was performed using AutoDock Vina.[46]

Docking model of *Snv*AT with malonyl-CoA was generated using a modified version of Alphafold[47], ColabFold[48]. Predicted local-distance difference test (lDDT)[49] values for relevant areas of the binding pocket are (>80). Molecular docking of malonyl-CoA was performed using AutoDock Vina.[46]

Subcellular localization and confocal microscopy analysis

Agrobacterium strains harboring *Snv*AT:RFP or *Sp*AT:RFP were transiently expressed together with free GFP (localized to cytoplasm) in *N. benthamiana* epidermal cells. After 3 days postinfiltration, leaf disks

(~0.4 cm diameter) were collected and analyzed for fluorescence with confocal microscopy using the following parameters Fluorescence of leaf discs mounted in water was observed using a Plan-Apochromat 20x/0.8 air objective on a cLSM 880 (both Zeiss, Oberkochen, Germany) microscope equipped with a 488 nm argon laser for excitation. Micrographs were taken sequentially, with GFP (cytosolic marker) emission filtered between 490 and 550 nm and red fluorescent protein (RFP) signals filtered between 550 and 650 nm. To improve the signal-to-noise ratio, each scan was sampled four times and averaged. The software was ZEN black 2.1 V.14.0.18.201 (Zeiss, Oberkochen, Germany).

NMR analysis

NMR spectra were measured on 400 MHz and 500 MHz Bruker Advance III HD spectrometers (Bruker Biospin GmbH, Rheinstetten, Germany). $CDCl_3$ and CD_2Cl_2 were used as solvents. NMR spectra were referenced to the residual solvent signals at dH 7.26 and dC 77.0 ppm for $CDCl_3$, and dH 5.32 and dC 54.0 ppm for CD_2Cl_2. For spectrometer control and data processing Bruker TopSpin ver. 3.6.1 was used.

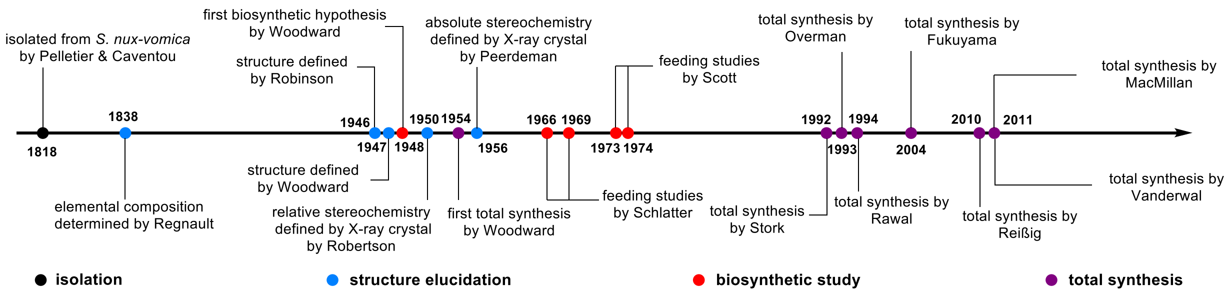

Fig. 1. Brief timeline of strychnine research.[4,50]

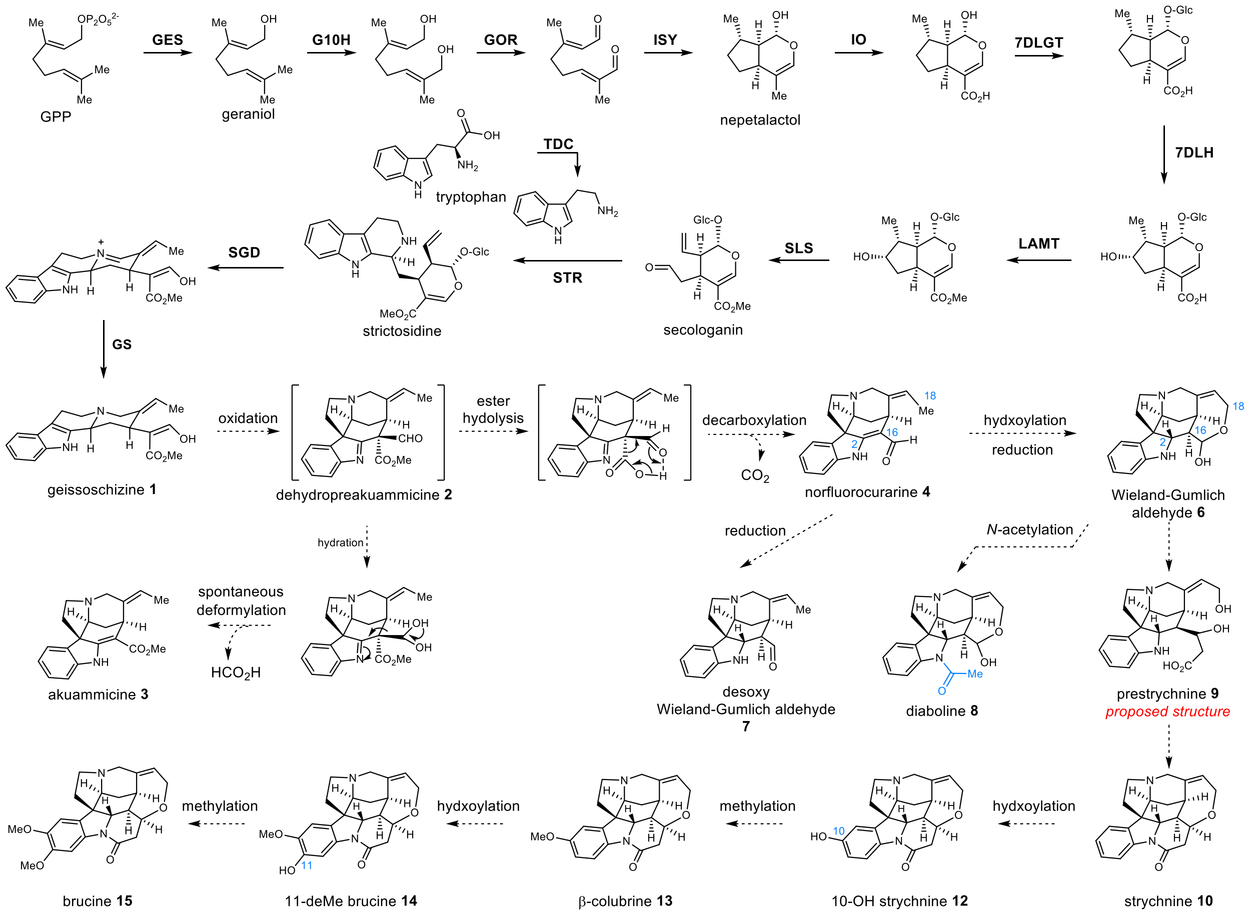

Fig. 2. Proposed biosynthetic pathway for diboline, strychnine, and brucine. Dashed lines represent the uncharacterized steps.

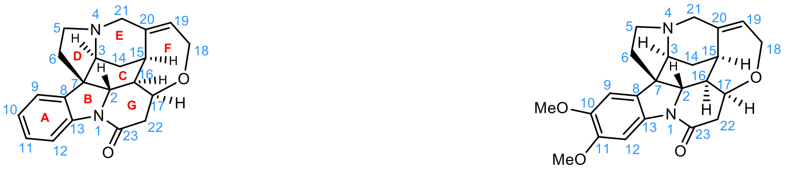

strychnine

brucine

Fig. 3. Atom numbering and ring annotation of strychnine and brucine

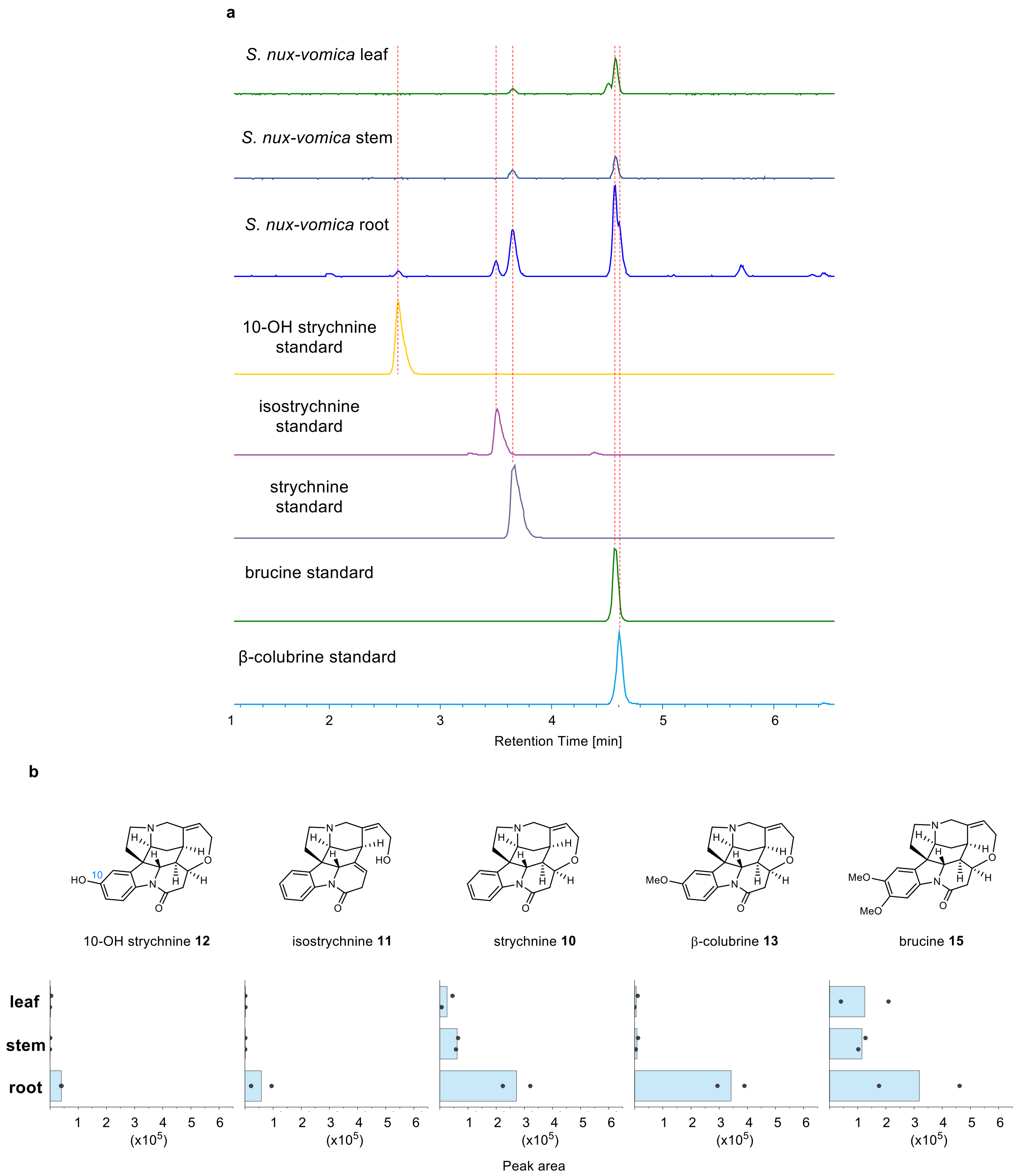

Fig. 4. Metabolic analysis of *Strychnos nux-vomica*. a. Total ion chromatogram (TIC) of methanolic extracts of tissues (leaf, stem, and root) from *S. nux-vomica* detected by LC-MS (Method 1). **b**. Average LC-MS peak area (n = 2 biological replicates) of strychnos alkaloids detected in different tissues of *S. nux-vomica.* Values represent the mean of n = 2 biological replicates. Source data are provided with this paper.

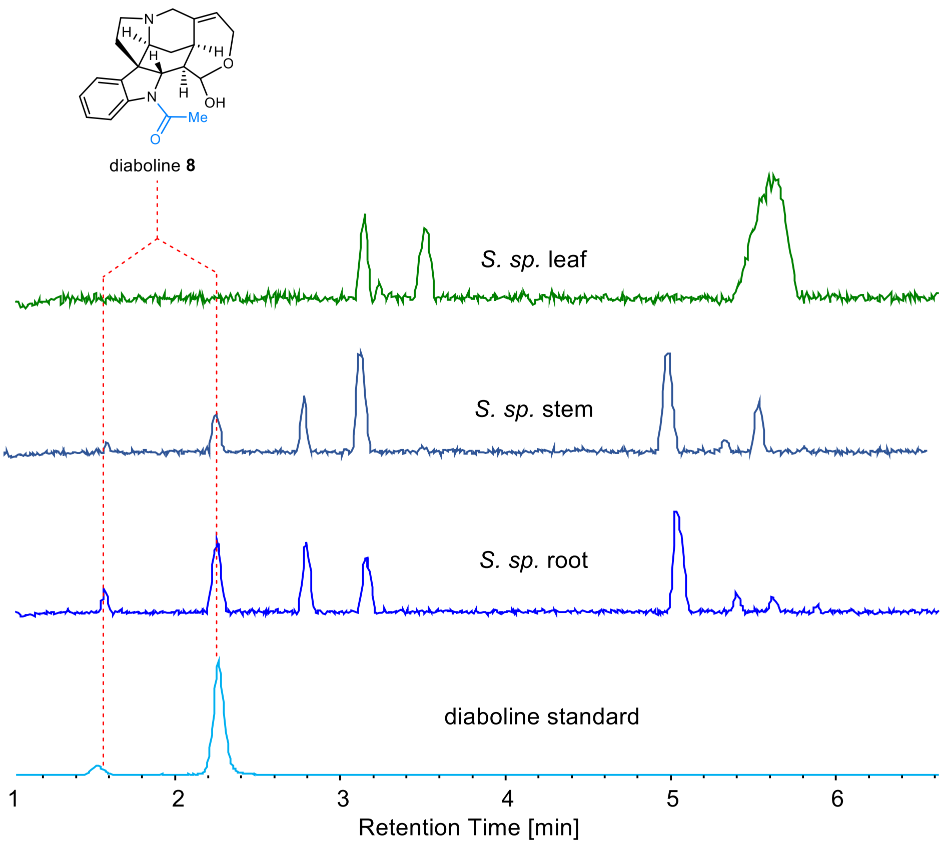

Fig. 5. Metabolic analysis of *Strychnos sp.*. Total ion chromatogram (TIC) of methanolic extracts of tissues (leaf, stem, and root) from *Strychnos sp.* detected by LC-MS (Method 1). Diaboline **8** is observed as two peaks in the chromatogram due to the hemiacetal diastereomers.

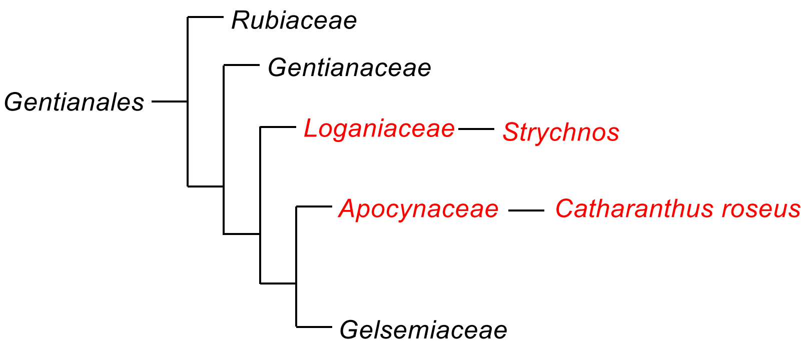

Fig. 6. Phylogenetic relationship of *Strychnos* and *Catharanthus roseus*.[51]

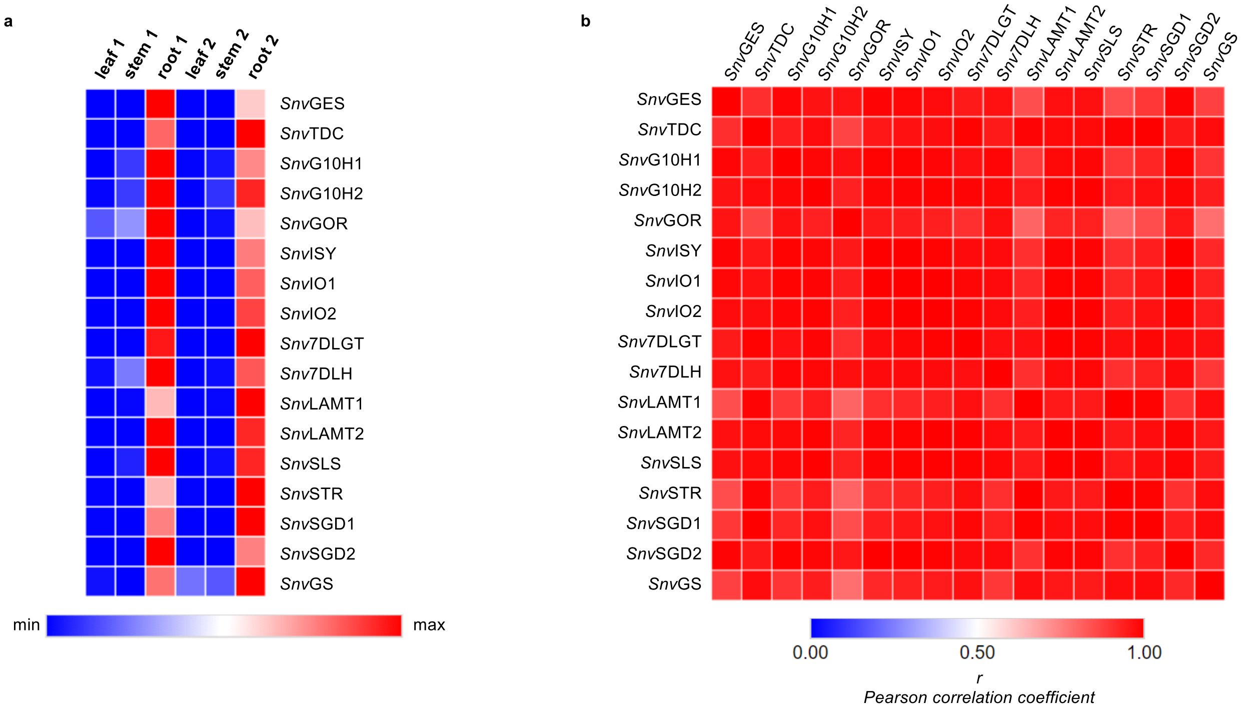

Fig. 7. Candidate genes for the biosynthesis of geissoschizine in *S. nux-vomica.* The candidate enzymes were predicted from the *S. nux-vomica* transcriptome based on a homology search with known genes from the geissoschizine producer *Catharanthus roseus*. Enzymes with high expression in root tissue were chosen as representative candidates, though were not functionally characterized in this study. **a**. Expression profiles of candidate genes for geissoschizine **1** biosynthesis in *S. nux-vomica.* Transcripts are quantified based on fragments per kilobase of transcript per million mapped reads (FPKM) values derived from the *S. nux-vomica* transcriptomes. Sample sets 1 and 2 represent two biological replicates. **b**. Coexpression analysis of candidate genes for geissoschizine **1** biosynthesis in *S. nux-vomica*. The heatmaps were generated by Morpheus (https://software.broadinstitute.org/morpheus). Source data are provided with this paper.

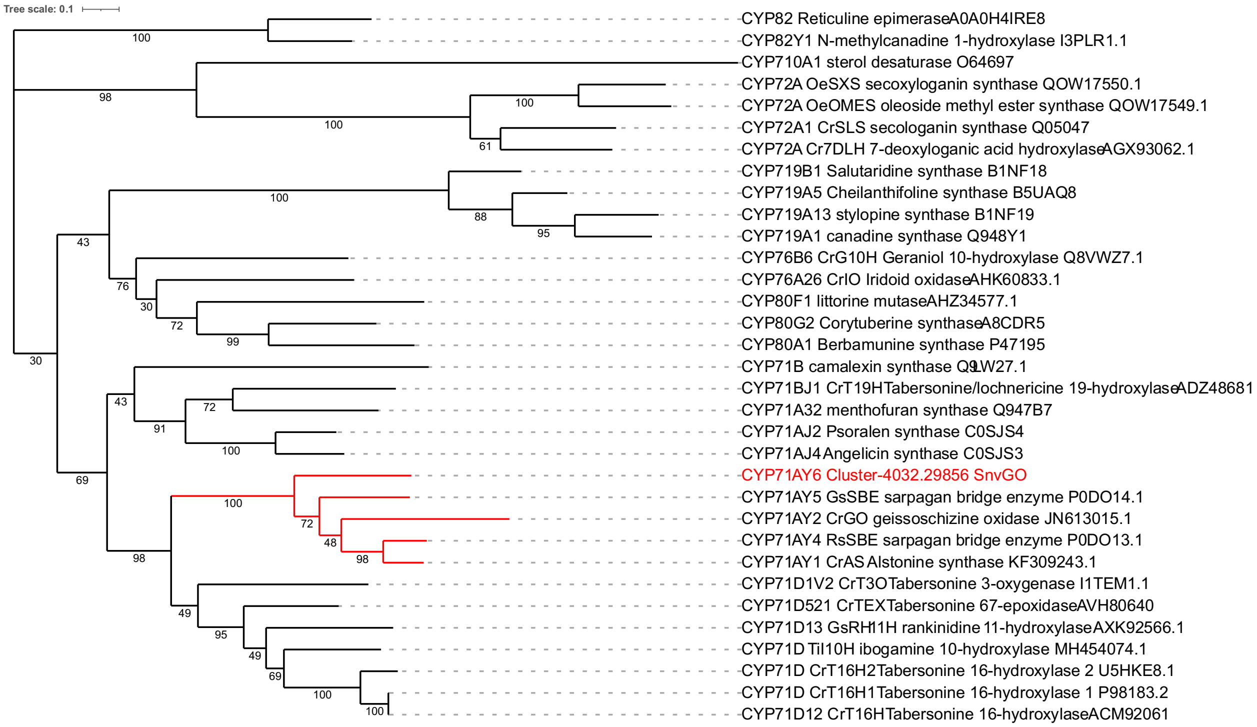

Fig. 8. Phylogenetic tree of *Snv*GO (Cluster-4032.29856) with previously characterized CYPs from other organisms. CYP71AY family is highlighted in red.

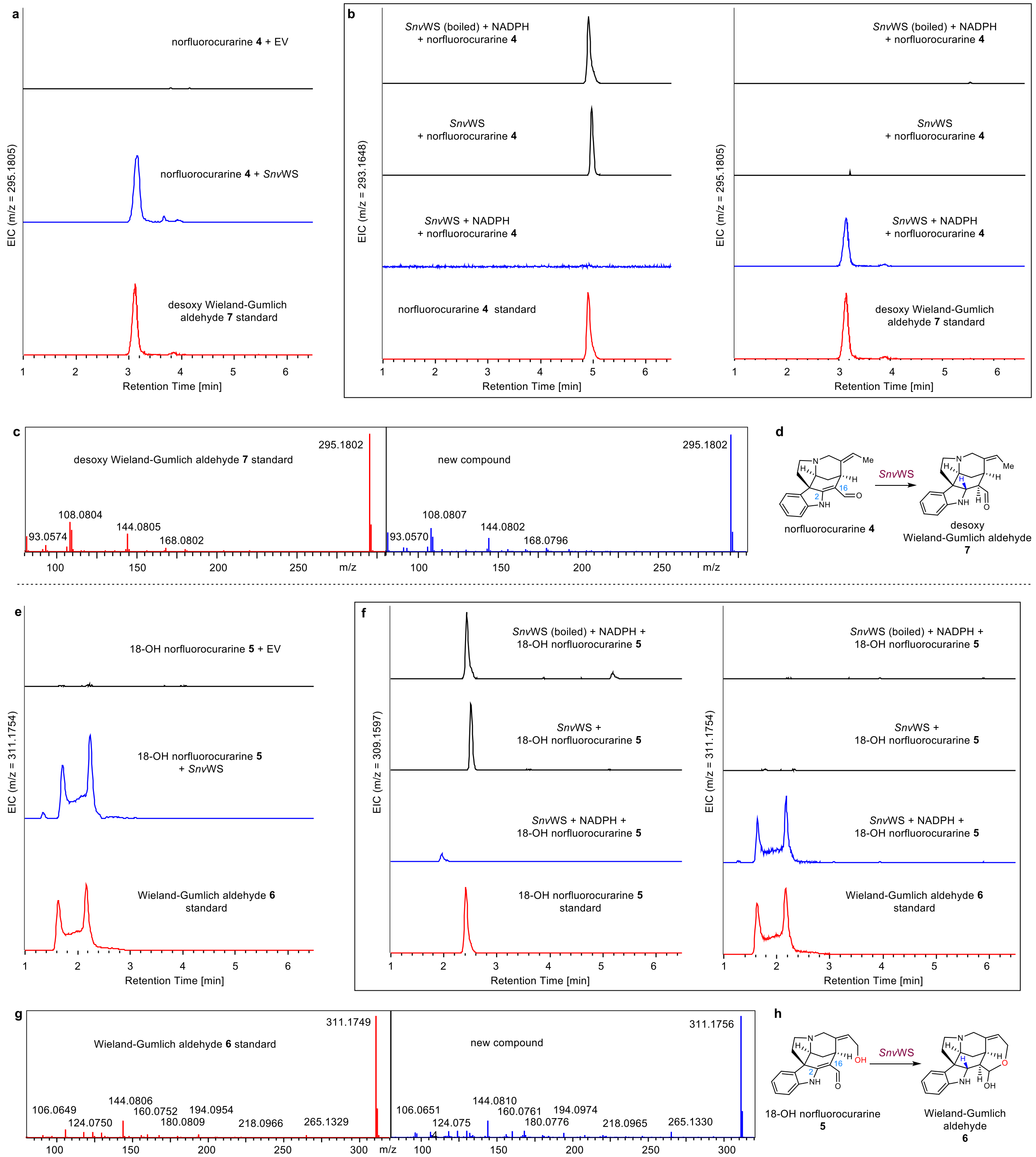

Fig. 9. Functional characterization of *Snv*WS with norfluorocurarine 4 and 18-OH norfluorocurarine 5. a. Transient expression of *Snv*WS in *N. benthamiana* with co-infiltration of norfluorocurarine **4**. Extracted ion chromatograms for desoxy Wieland-Gumlich aldehyde **7** (*m/z* $[M+H]^+$ = 295.1805 ± 0.05). This experiment was repeated three times with similar results. **b**. *In vitro* assays using purified *Snv*WS from SoluBL21 *E. coli.* Extracted ion chromatograms for norflurocurarine **4** (*m/z* $[M+H]^+$ = 293.1648 ± 0.05, left) and desoxy Wieland-Gumlich aldehyde **7** (*m/z* $[M+H]^+$ = 295.1805 ± 0.05, right). This experiment was repeated more than three times with similar results. **c**. MS/MS (20 to 50 eV) spectra of produced desoxy

Wieland-Gumlich aldehyde **7** (blue) compared to synthetic standard (red). **d.** Reaction catalyzed by *Snv*WS. **e**. Transient expression of *Snv*WS in *N. benthamiana* with co-infiltration of 18-OH norfluorocurarine **5**. Extracted ion chromatograms for Wieland-Gumlich aldehyde **6** (*m/z* $[M+H]^+$ = 311.1754 ± 0.05). This experiment was repeated three times with similar results. **f**. *In vitro* assays using purified *Snv*WS from SoluBL21 *E. coli.* Extracted ion chromatograms for 18-OH norflurocurarine **5** (*m/z* $[M+H]^+$ = 309.1567 ± 0.05, left) and Wieland-Gumlich aldehyde **6** (*m/z* $[M+H]^+$ = 311.1754 ± 0.05, right). This experiment was repeated more than three times with similar results. **g**. MS/MS (20 to 50 eV) spectra of generated Wieland-Gumlich aldehyde **6** (blue) compared to synthetic standard (red). **h**. Reaction catalyzed by *Snv*WS.

a

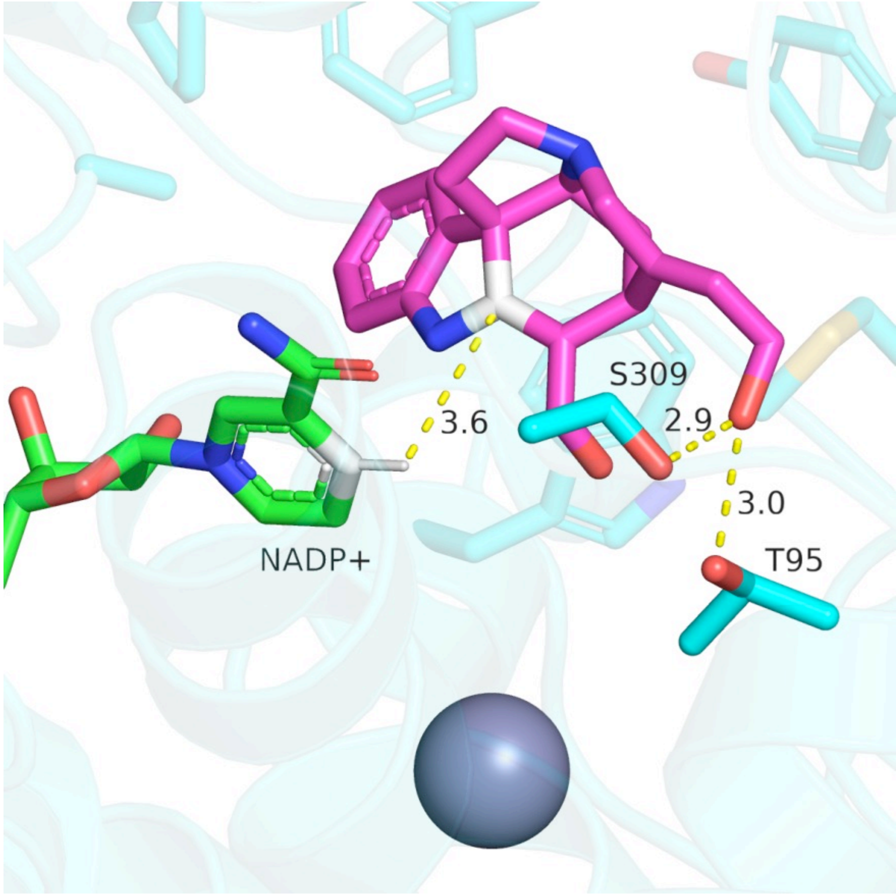

b

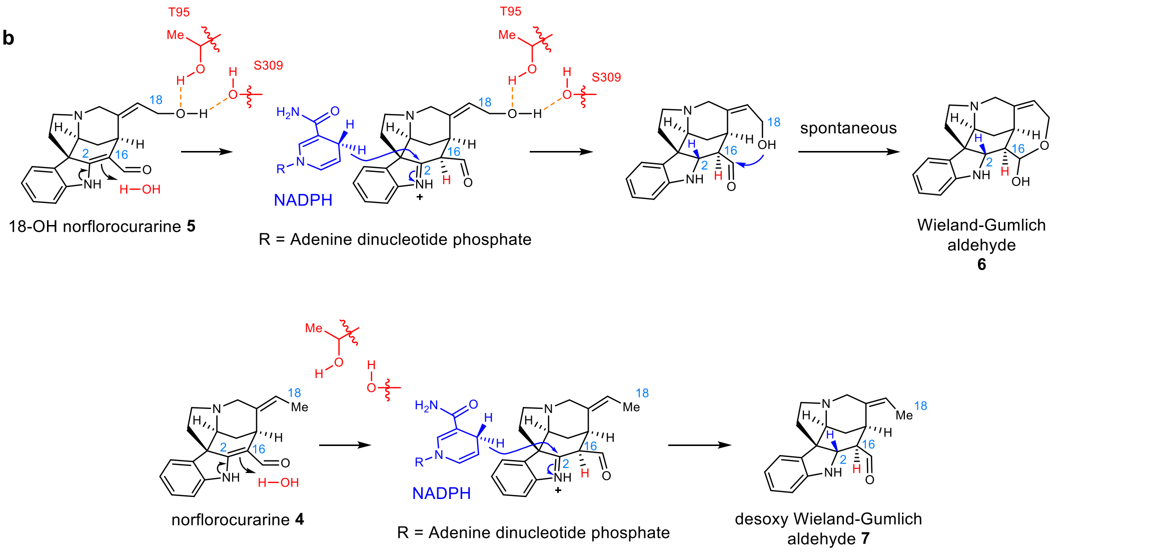

Fig. 10. Mechanistic hypothesis for *Snv*WS. a. Docking model of *Snv*WS with 18-OH norflurocurarine **5** (pink carbons). Zinc ion is displayed as grey spheres. T95 and S309 in *Snv*WS are probably responsible for the substrate binding via forming hydrogen bonds with C18 hydroxyl group in 18-OH norfluorocurarine **5**. **b.** Proposed mechanism for the stereoselective formation of C2 and C16 chiral center in **6** and **7**.

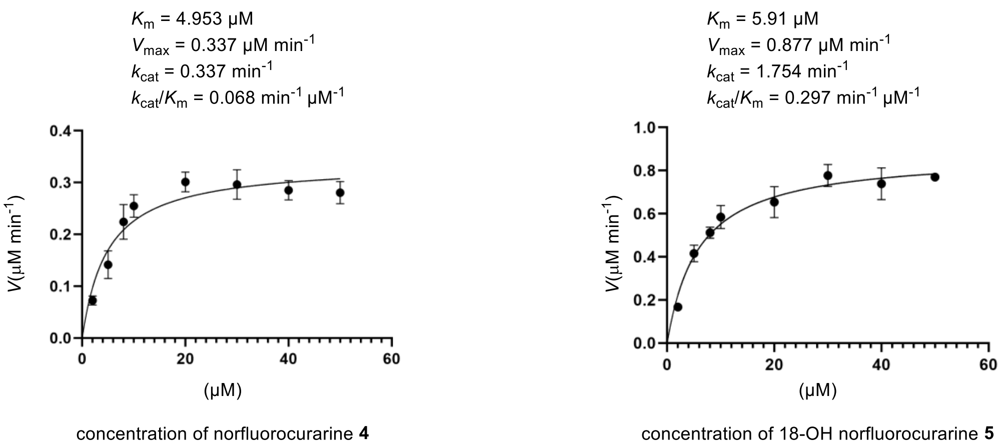

Fig. 11. Kinetic characterization of *Snv*WS for 4 and 5. Values represent the mean of three independent assays (n = 3). Error bars represent the standard error of three independent assays (n = 3). Source data are provided with this paper.

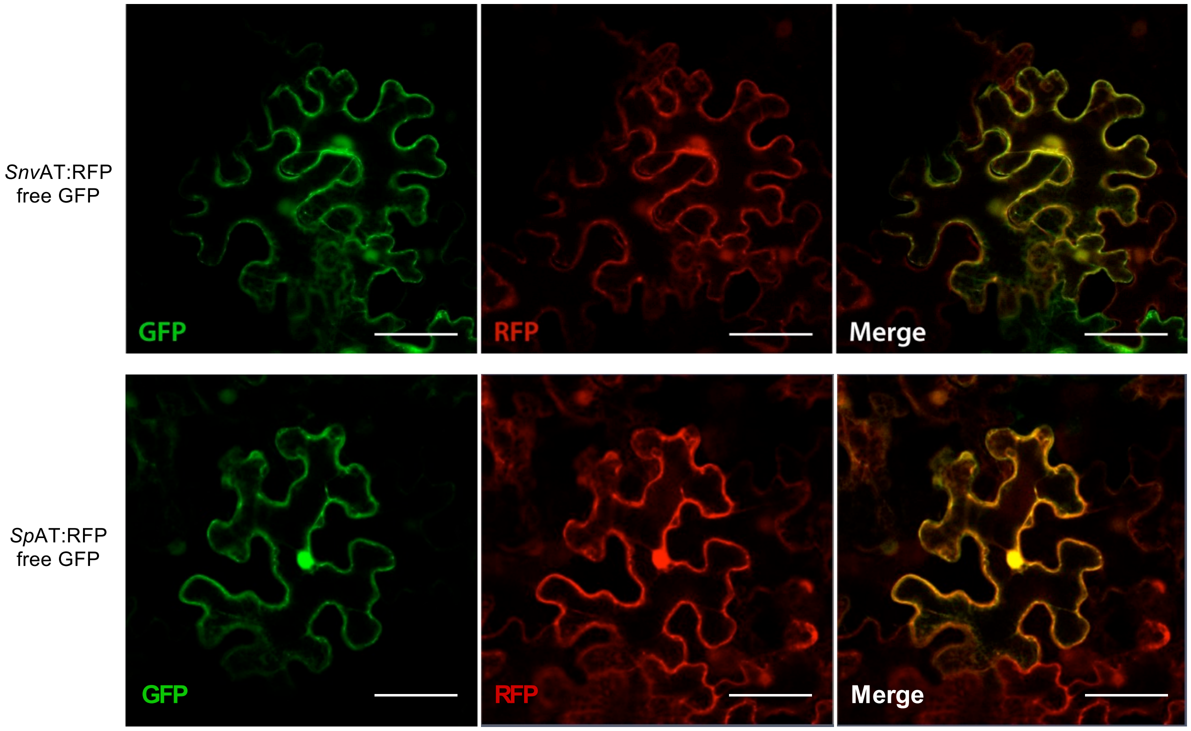

Fig. 12. *Snv*AT and *Sp*AT are targeted to the cytoplasm. Confocal images of transient expression of *Snv*AT and *Sp*AT fused to a fluorescent reporter (*Snv*AT:RFP and *Sp*AT:RFP) in *N. benthamiana* together with a free GFP (localized to cytoplasm). The panel contain images of GFP, RFP and both channels merged together. Scale bar – 50 µm. This experiment was repeated at least three times.

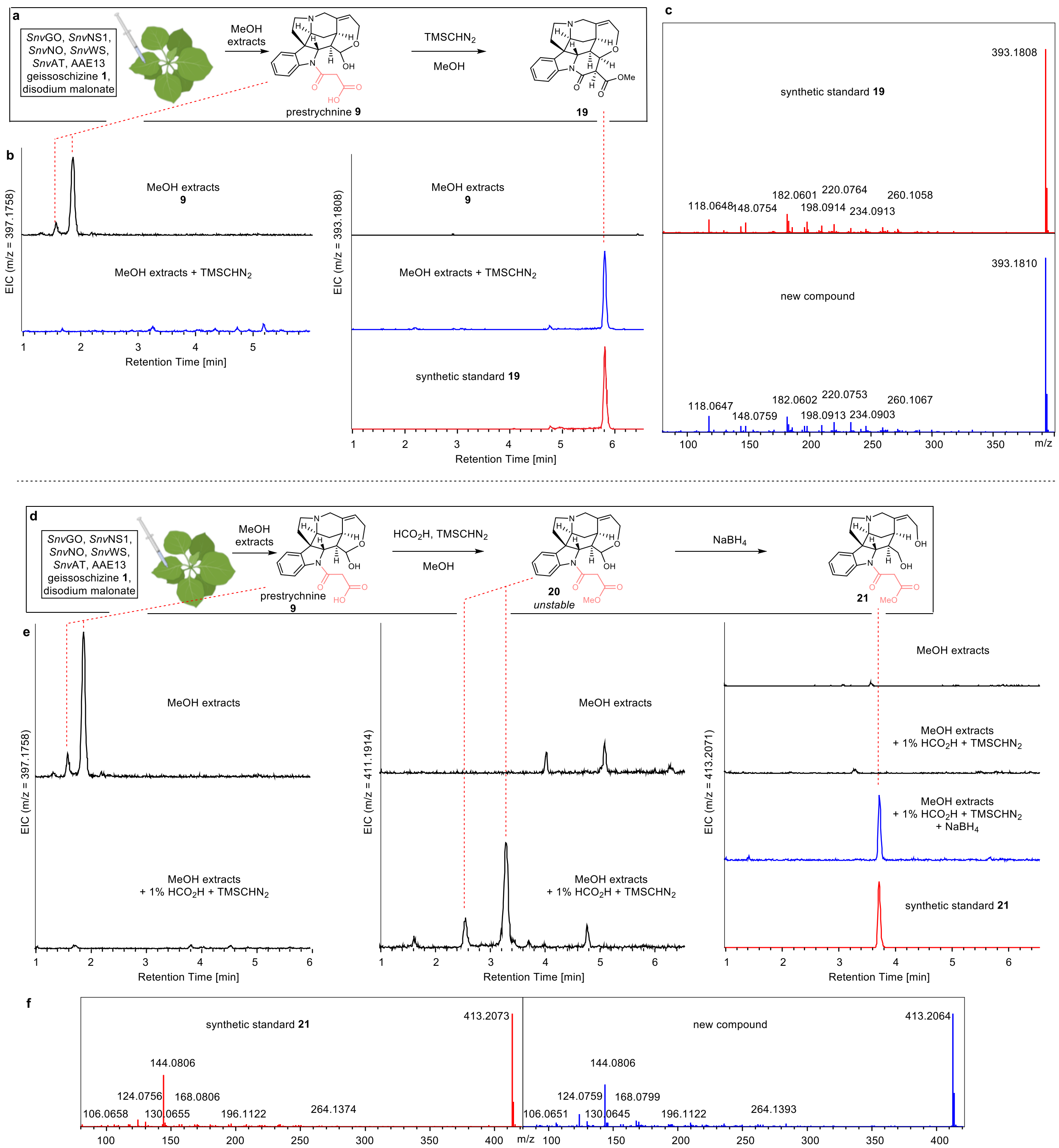

Fig. 13. Derivatization of 9. a. Reaction mediated by Trimethylsilyldiazomethane (TMSD). **b**. Extracted ion chromatograms for prestrychnine **9** (*m/z* $[M+H]^+$ = 397.1758 ± 0.05, left) and **19** (*m/z* $[M+H]^+$ = 393.1808 ± 0.05, right). This experiment was repeated three times with similar results. **c**. MS/MS (20 to 50 eV) spectra of generated *m/z [M+H]+* 393.1808 in MeOH extracts after derivatization compared to synthetic standard (red). **d**. Reaction mediated by Trimethylsilyldiazomethane (TMSD) and sodium borohydride ($NaBH_4$). **e.** Extracted ion chromatograms for prestrychnine **9** (*m/z* $[M+H]^+$ = 397.1758 ± 0.05, left), **20** (*m/z* $[M+H]^+$ = 411.1914 ± 0.05**,** middle) and **21** (*m/z* $[M+H]^+$ = 413.2071 ± 0.05, right). This experiment was repeated three times with similar results. **f**. MS/MS (20 to 50 eV) spectra of generated *m/z* $[M+H]^+$ = 413.2064 in MeOH extracts after derivatization compared to synthetic standard (red).

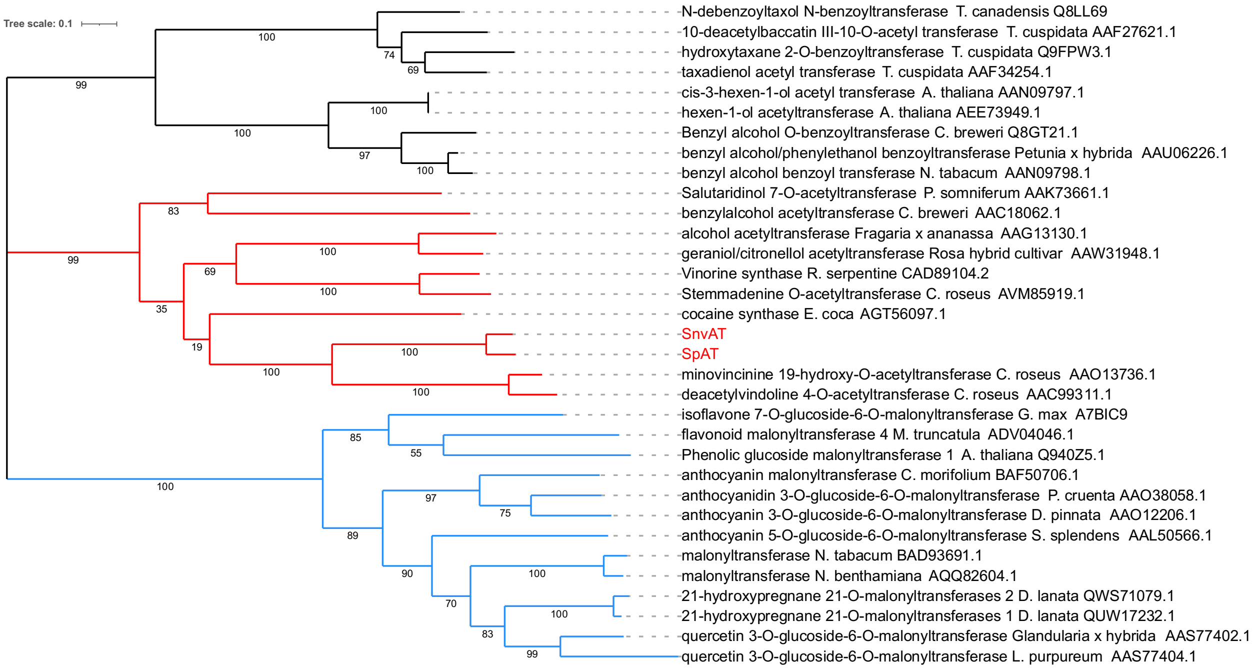

Fig. 14. Phylogenetic tree of *Snv*AT and *Sp*AT with previously characterized BAHD acyltransferases. Acetyltransferase clade is highlighted in red, malonyltransferase clade is highlighted in teal.

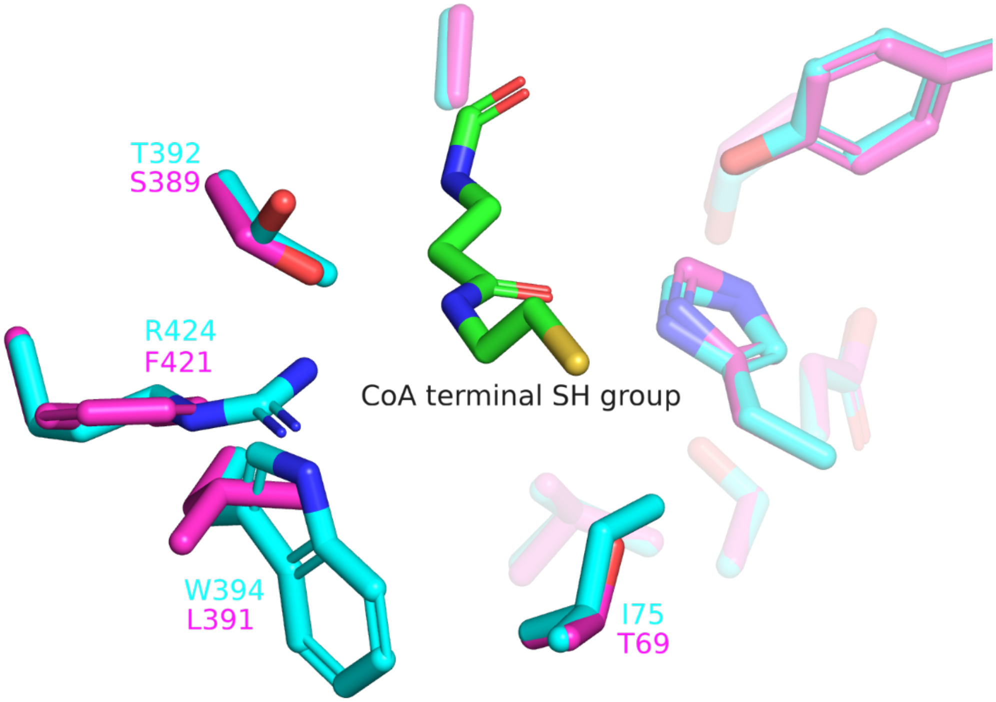

Fig. 15. Protein model of *Snv*AT and *Sp*AT. The model was created based on *Arabidopsis thaliana* spermidine hydroxycinnamoyl transferase (PDB ID: 6LPV, 24% AA identity) containing Coenzyme A (green).[29] The residues (close to the terminal thiol group of Coenzyme A) that are probably responsible for the cofactor binding are highlighted and are numbered based on the *Snv*AT (cyan) and *Sp*AT (pink).

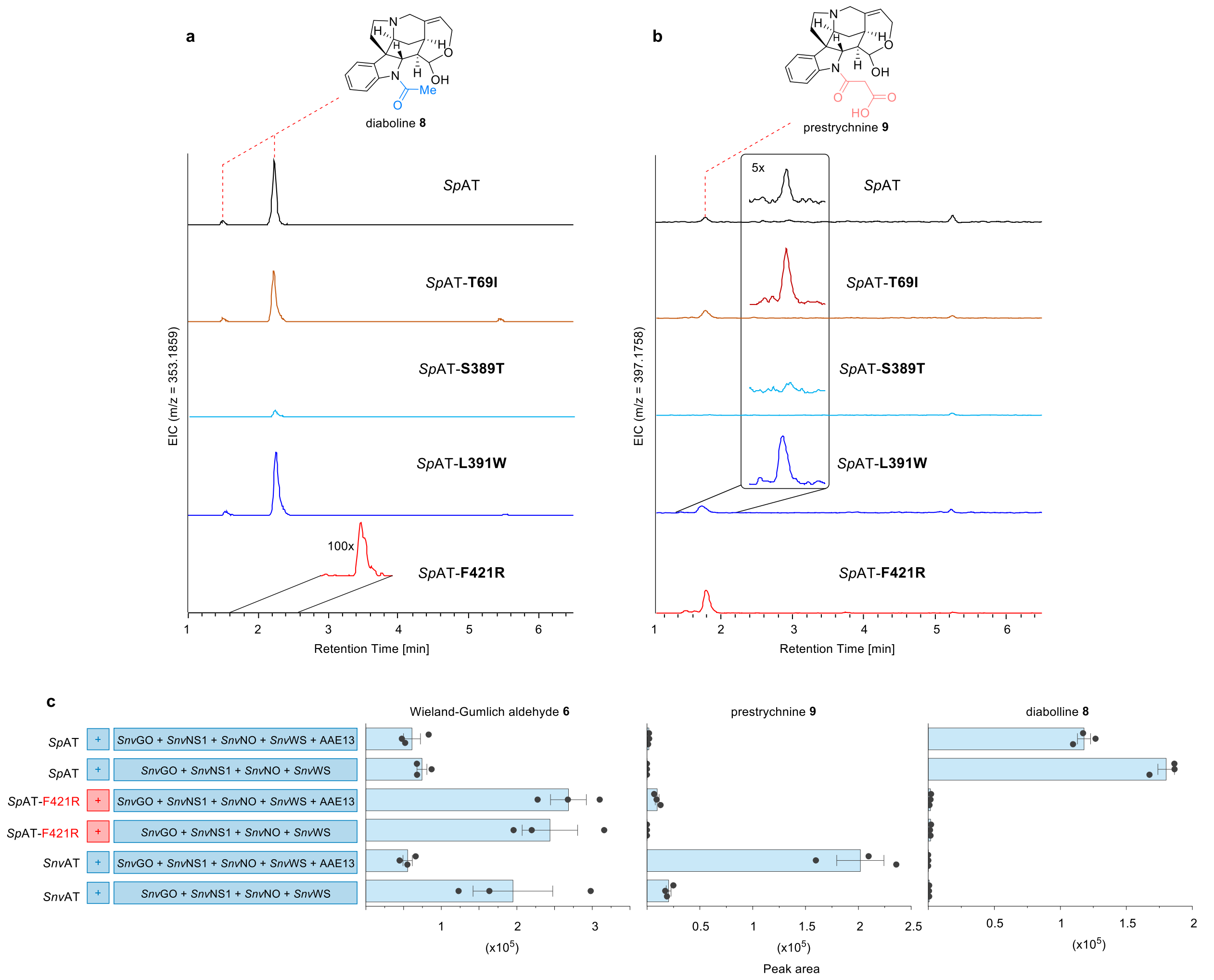

Fig. 16. Site directed mutagenesis of *Sp*AT. a. Extracted ion chromatograms for diaboline **8** (m/z $[M+H]^+$ = 353.1859 ± 0.05) from *N. benthamiana* leaves transiently expressing *Snv*GO, *Snv*NS1, *Snv*NO, *Snv*WS, and mutated versions of *Sp*AT compared to wild-type *Sp*AT in the presence of geissoschizine **1**. These experiments were repeated three times with similar results. **b**. Extracted ion chromatograms prestrychnine **9** (m/z $[M+H]^+$ = 397.1758 ± 0.05) from *N. benthamiana* leaves transiently expressing *Snv*GO, *Snv*NS1, *Snv*NO, *Snv*WS, AAE13 and mutated versions of *Sp*AT compared to wild-type *Sp*AT in the presence of geissoschizine **1** and disodium malonate. These experiments were repeated three times with similar results. **c.** Average LC-MS peak area (n = 3 biological replicates) of prestrychnine **9** and diaboline **8** that produced by *Snv*AT, *Sp*AT and *Sp*AT-F421R mutant. Data are presented as mean ± SEM (n = 3). Source data are provided with this paper.

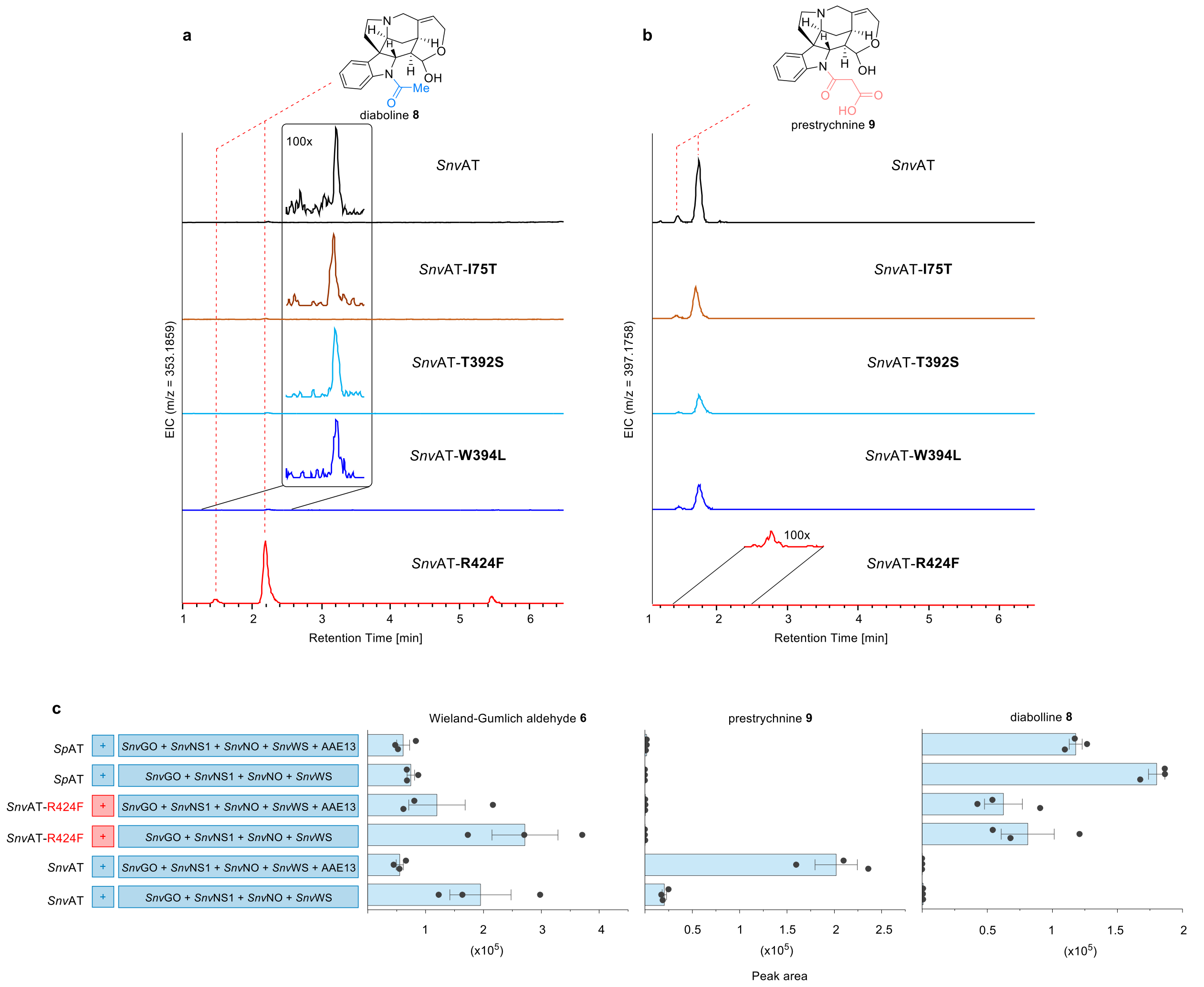

Fig. 17. Site directed mutagenesis of *Snv*AT. a. Extracted ion chromatograms for diaboline **8** (m/z $[M+H]^+$ = 353.1859 ± 0.05) from *N. benthamiana* leaves transiently expressing *Snv*GO, *Snv*NS1, *Snv*NO, *Snv*WS, and mutated versions of *Snv*AT compared to wild-type *Snv*AT in the presence of geissoschizine **1**. These experiments were repeated three times with similar results. **b.** Extracted ion chromatograms for prestrychnine **9** (m/z $[M+H]^+$ = 397.1758 ± 0.05) from *N. benthamiana* leaves transiently expressing *Snv*GO, *Snv*NS1, *Snv*NO, *Snv*WS, AAE13 and mutated versions of *Snv*AT compared to wild-type *Snv*AT in the presence of geissoschizine **1** and disodium malonate. These experiments were repeated three times with similar results. **c.** Average LC-MS peak area (n = 3 biological replicates) of prestrychnine **9** and diaboline **8** that produced by *Snv*AT, *Sp*AT and *Snv*AT-R424F mutant in *N. benthamiana*. Date are presented as mean ± SEM (n = 3). Source data are provided with this paper.

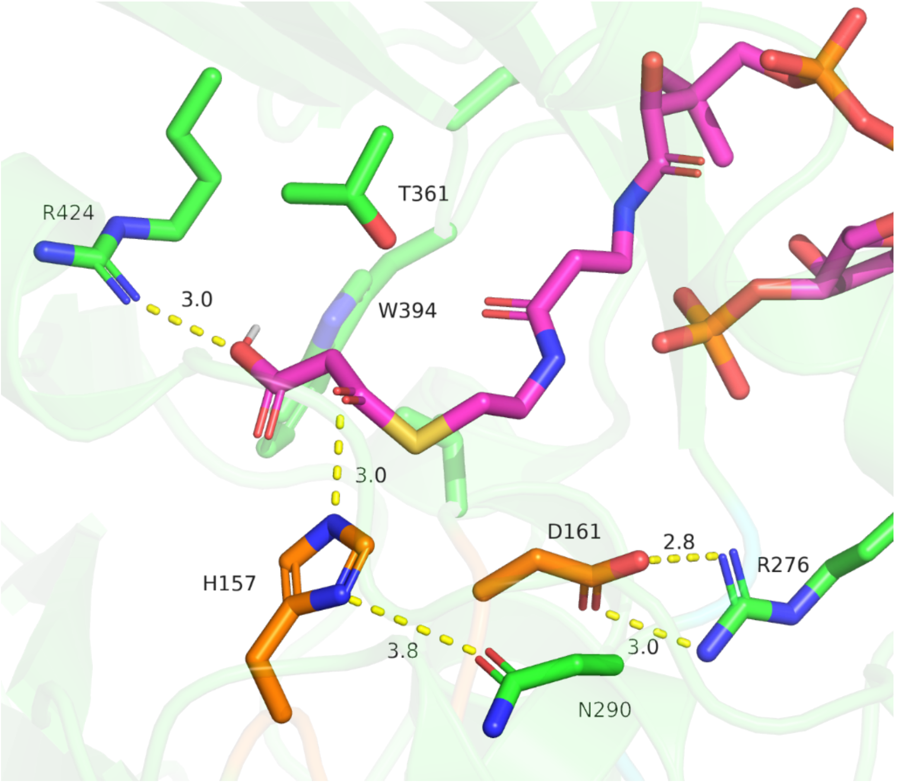

Fig. 18. Docking model of *Snv*AT with malonyl-CoA. Malonyl-CoA is shown with pink carbons. BAHD acyl transferase protein, HXXXD motif highlighted in orange with conserved interactions.[30] Malonyl-CoA molecule docked in the model predicts hydrogen bonding of the terminal carboxylic acid with R424.

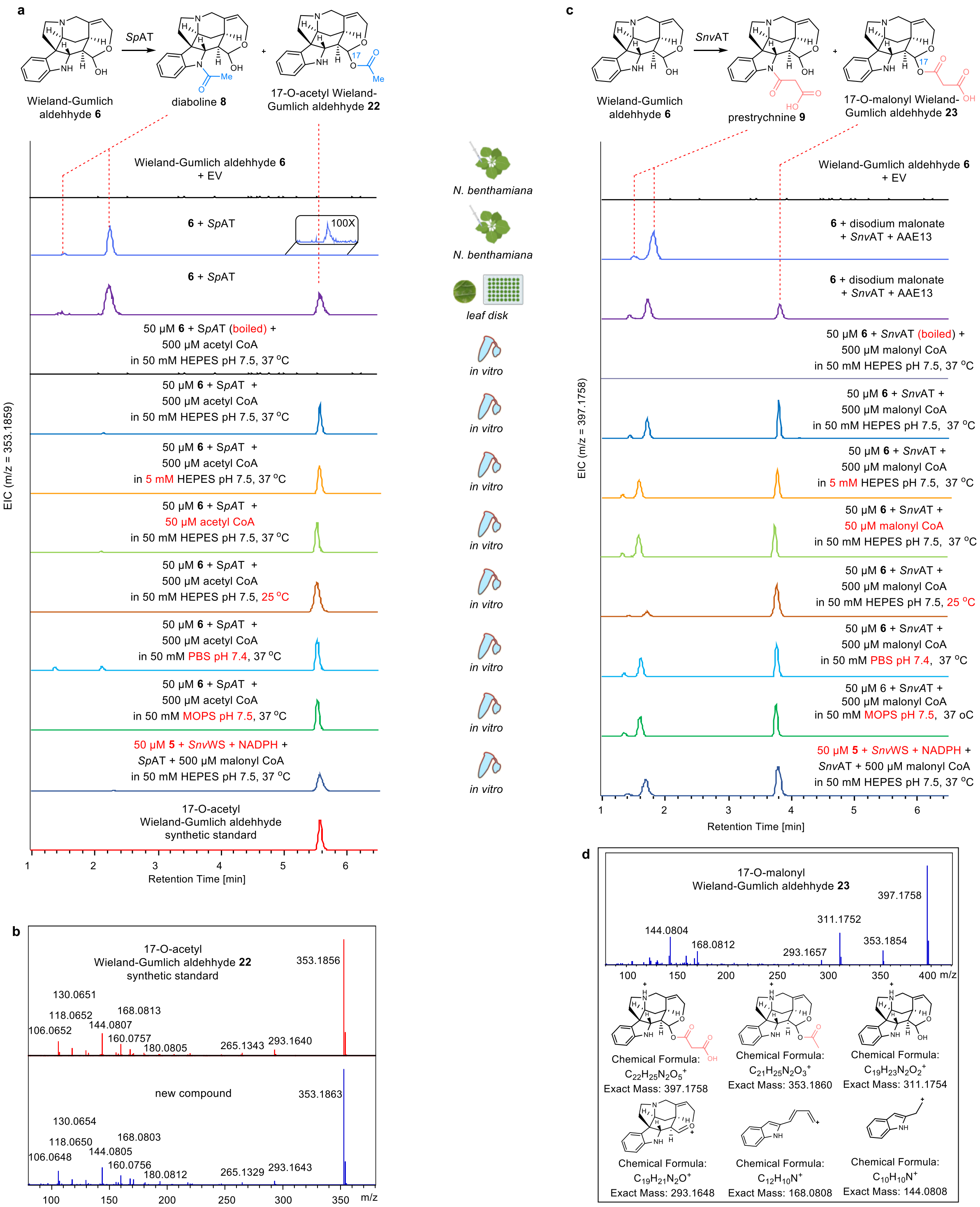

Fig. 19. Comparison of *in vivo* and *in vitro* reactivity of *Sp*AT and *Snv*AT under various conditions. a. Reactivity of *Sp*AT. *In vivo:* Transient expression of *Sp*AT in *N. benthamiana* with co-infiltration of Wieland-Gumlich aldehyde **6**. *In vitro:* Enzymatic assays were performed in a total volume of 100 μL indicated buffer (50 mM or 5 mM); **6** (50 μM); acetyl-CoA (50 μM or 500 μM) and *Sp*AT (1 μM, purified from SoluBL21 *E. coli.* or *N. benthaminana*). Reactions were incubated at 25 °C or 37 °C for 12 hours. Extracted ion chromatograms for generated *m/z* $[M+H]^+$ = 353.1859 ± 0.05. These experiments were repeated three

times with similar results. **b**. MS/MS (20 to 50 eV) spectra of generated 17-O-acetyl Wieland-Gumlich aldehyde (blue) compared to synthetic standard (red). **c.** Reactivity of *Snv*AT. *In vivo:* Transient expression of *Snv*AT and AAE13 in *N. benthamiana* with co-infiltration of Wieland-Gumlich aldehyde **6** and disodium malonate. *In vitro*: Enzymatic assays were performed in a total volume of 100 µL indicated buffer (50 mM or 5 mM); **6** (50 µM); malonyl-CoA (50 µM or 500 µM) and *Snv*AT (1 µM, purified from SoluBL21 *E. coli.* or *N. benthaminana*). Reactions were incubated at 25 ^{o}C or 37 ^{o}C for 12 hours. Extracted ion chromatograms for generated *m/z* $[M+H]^+$ = 397.1758 ± 0.05. These experiments were repeated three times with similar results. **d**. MS/MS (20 to 50 eV) spectra and putative ion fragments of generated 17-O-malonyl Wieland-Gumlich aldehyde **23** (*m/z* $[M+H]^+$ 397.1758).

Note: The differences between *in vitro* selectivities under different conditions compared with the robust *in vivo* selectivities is likely due to a suboptimal reaction environment for the purified proteins compared to a cellular environment. We also speculate that the equilibration between the closed hemiacetal form and the open aldehyde form of the Wieland-Gumlich aldehyde plays a substantial role in this selectivity. NMR analysis indicates that the open form of the Wieland-Gumlich aldehyde is not observed *in vitro*, and the availability of the open form of the Wieland-Gumlich aldehyde may be more accessible in the cellular environment.

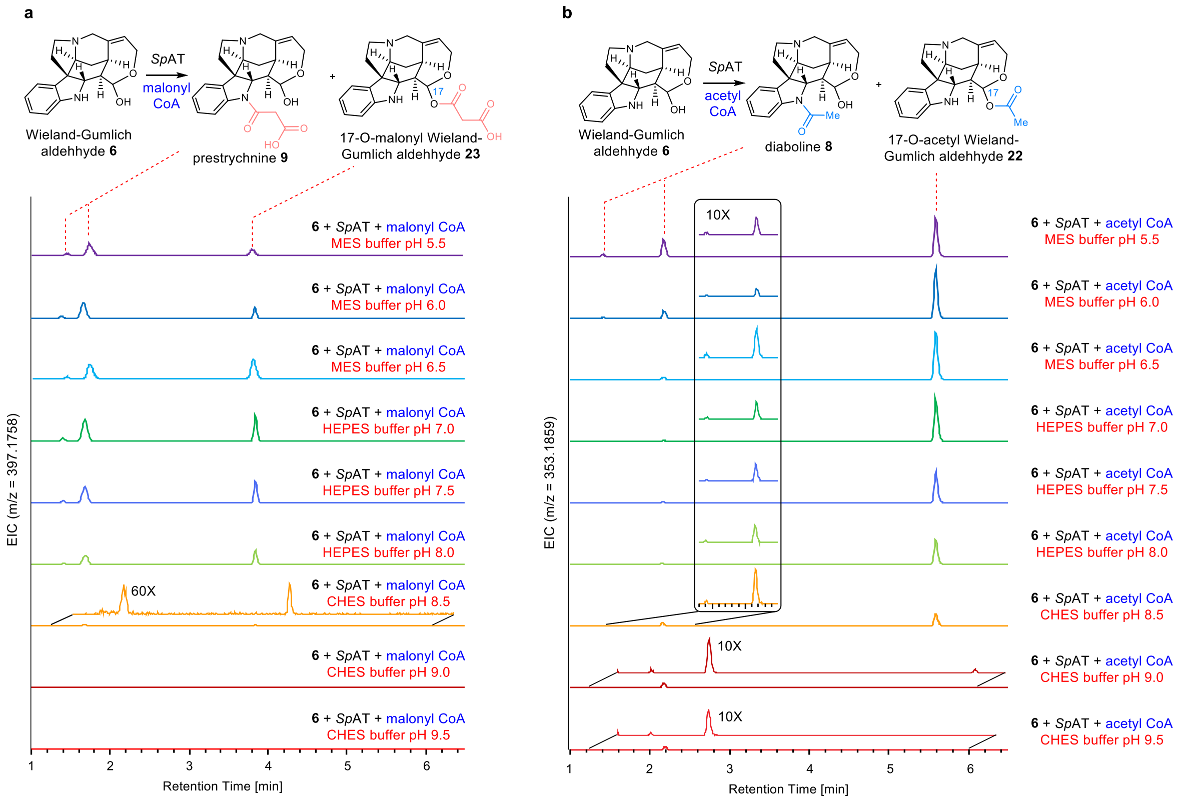

Fig. 20. *In vitro* reactivity of *Sp*AT at various pH. a. *In vitro* activity of *Sp*AT using malonyl-CoA as an acyl donor at various pH. Enzymatic assays were performed in a total volume of 100 µL indicated buffer (50 mM, pH 5.5 to 9.5); Wieland-Gumlich aldehyde **6** (50 µM); malonyl-CoA (500 µM) and *Sp*AT (1 µM). Reactions were incubated at 37 °C for 12 hours. Extracted ion chromatograms for generated *m/z* $[M+H]^+$ = 397.1758 ± 0.05. These experiments were repeated two times with similar results. **b.** *In vitro* activity of *Sp*AT using acetyl-CoA as an acyl donor. Enzymatic assays were performed in a total volume of 100 µL indicated buffer (50 mM, pH 5.5 to 9.5); Wieland-Gumlich aldehyde Wieland-Gumlich aldehyde **6** (50 µM); acetyl-CoA (500 µM) and *Sp*AT (1 µM). Reactions were incubated at 37 °C for 12 hours. Extracted ion chromatograms for generated *m/z* $[M+H]^+$ = 353.1859 ± 0.05. These experiments were repeated two times with similar results.

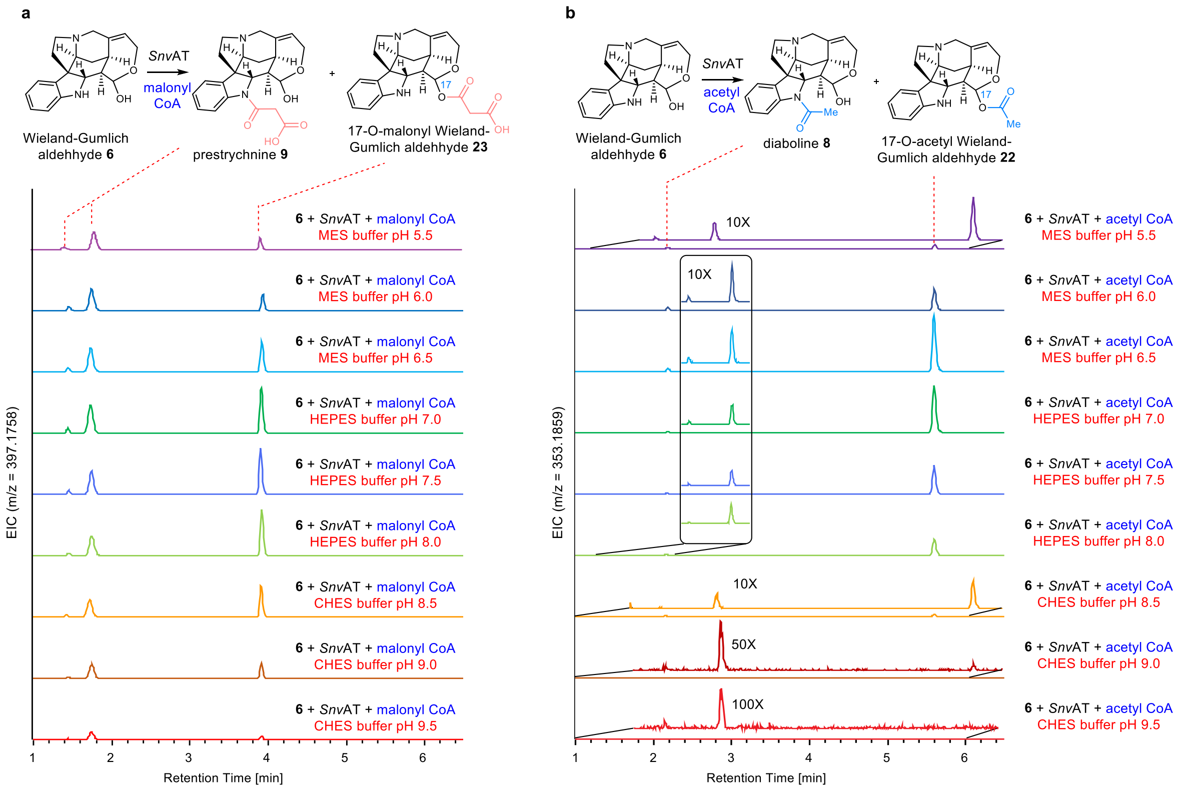

Fig. 21. *In vitro* reactivity of *Snv*AT at various pH. a. *In vitro* activity of *Snv*AT using malonyl-CoA as an acyl donor. Enzymatic assays were performed in a total volume of 100 µL indicated buffer (50 mM, pH 5.5 to 9.5); Wieland-Gumlich aldehyde Wieland-Gumlich aldehyde **6** (50 µM); malonyl-CoA (500 µM) and *Snv*AT (1 µM). Reactions were incubated at 37 °C for 12 hours. Extracted ion chromatograms for generated *m/z* $[M+H]^+$ = 397.1758 ± 0.05. These experiments were repeated two times with similar results. **b.** *In vitro* activity of *Snv*AT using acetyl-CoA as an acyl donor. Enzymatic assays were performed in a total volume of 100 µL indicated buffer (50 mM, pH 5.5 to 9.5); Wieland-Gumlich aldehyde Wieland-Gumlich aldehyde **6** (50 µM); acetyl-CoA (500 µM) and *Snv*AT (1 µM). Reactions were incubated at 37 °C for 12 hours. Extracted ion chromatograms for generated *m/z* $[M+H]^+$ = 397.1758 ± 0.05. These experiments were repeated two times with similar results.

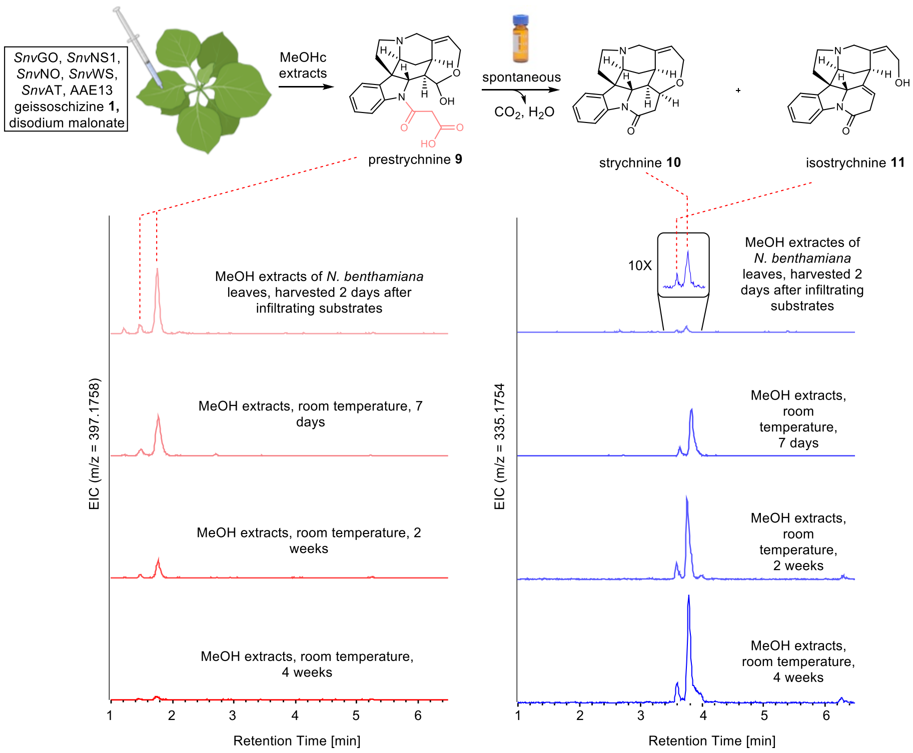

Fig. 22. Time course of strychnine and isostrychnine accumulation. Methanolic extracts of *N. benthamiana* leaves that produce prestrychnine **9** were stored at room temperature (25 °C). The samples were analyzed directly by untargeted LC-MS (Method 1) at 1st, 7th, 14th and 28th day. Extracted ion chromatograms for prestrychnine **9** (*m/z* $[M+H]^+$ = 397.1758 ± 0.05, left) and strychnine **10** and isostrychnine **11** (*m/z* $[M+H]^+$ = 335.1754 ± 0.05, right). This experiment was repeated three times with similar results.

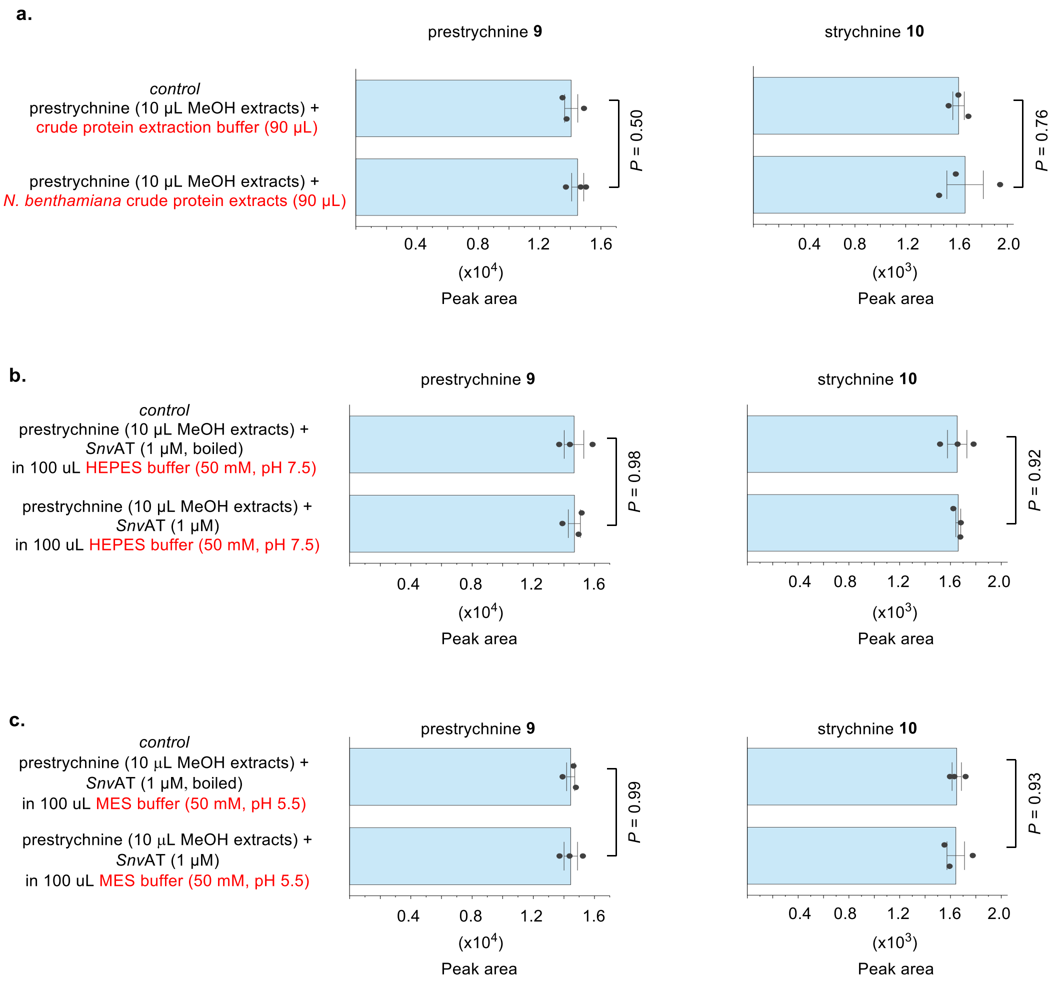

Fig. 23. *In vitro* assays of prestrychnine. Assays were performed using crude protein extracts from *N. benthamiana* and recombinant *Snv*AT. **a**. Incubating prestrychnine (10 µL MeOH extracts from *N. benthamiana* leaves) with crude protein extracts from *N. benthamiana* (90 µL). **b**. Incubating prestrychnine (10 µL MeOH extracts) with recombinant *Snv*AT (1 µM) in HEPES buffer (50 mM, pH 7.5, physiological pH). **c**. Incubating prestrychnine (10 µL MeOH extracts) with recombinant *Snv*AT (1 µM) in MES buffer (50 mM, pH 5.5, in which the best N-malonyl selectivity was overserved *in vitro*). Average LC-MS peak area (n = 3 independent assays) of prestrychnine **9** and strychnine **10** after incubation. Date are presented as mean ± SEM (n = 3). Statistical comparisons were calculated by Student's t test (two-tailed) compared to negative control. Source data are provided with this paper.

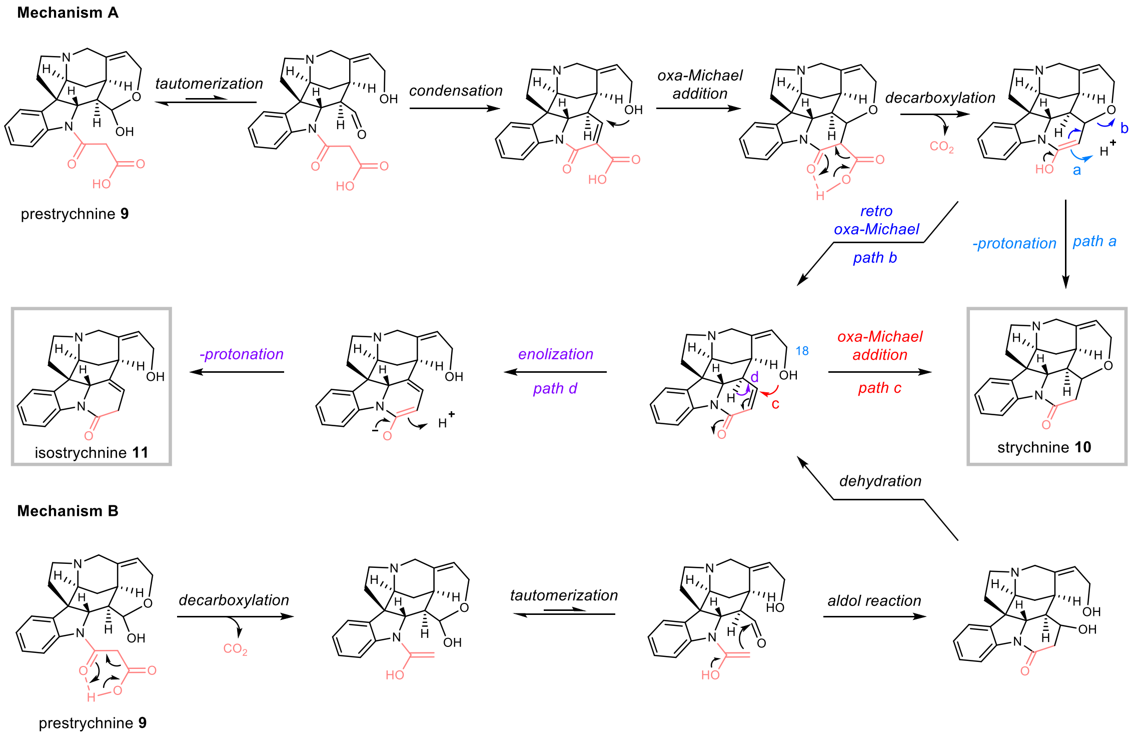

Fig. 24. Proposed mechanism for the strychnine 10 and isostrychnine 11 formation.

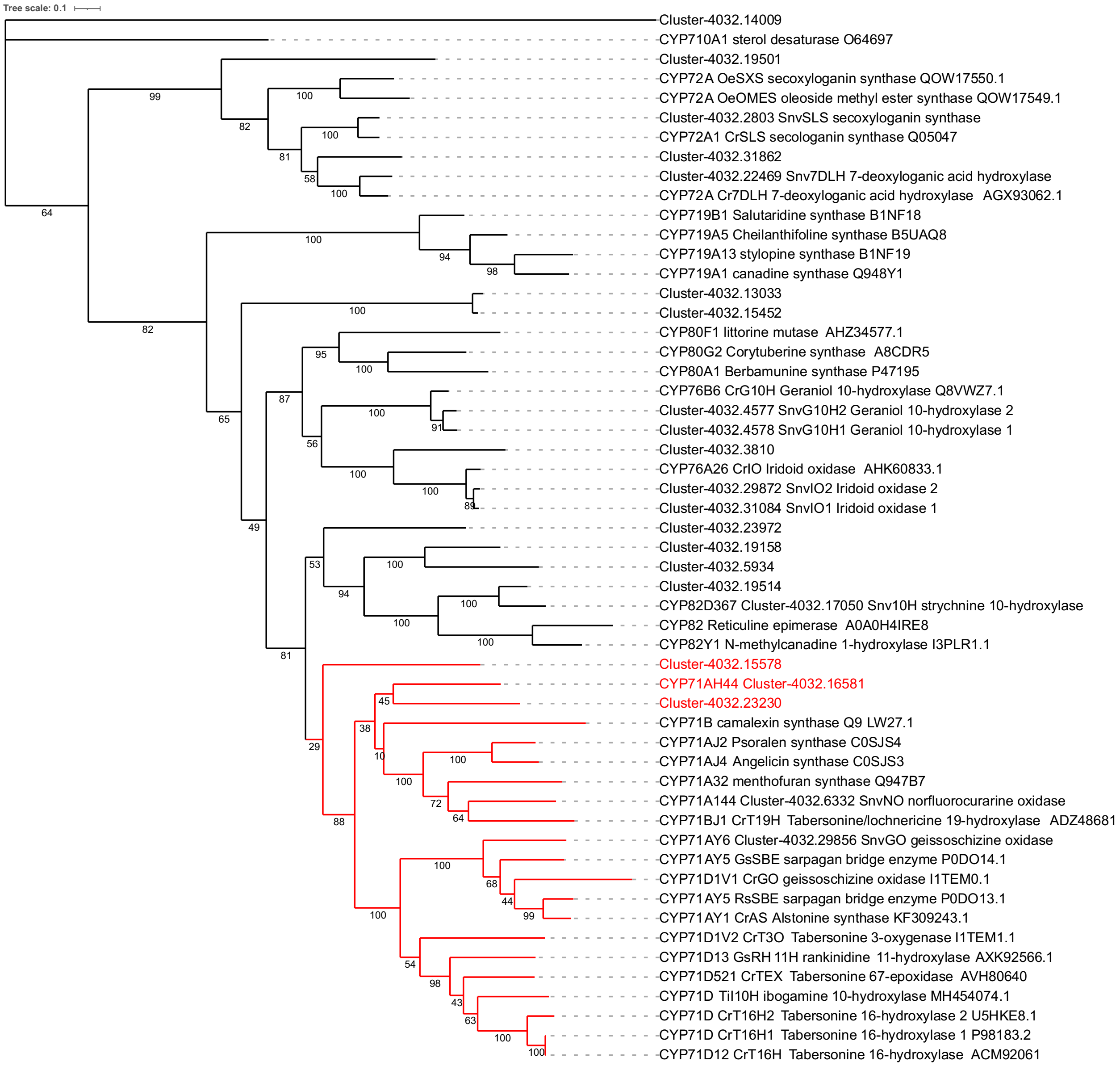

Fig. 25. Phylogenetic tree of cytochromes P450 that highly expressed (FPKM ≥ 20) in the root of *S. nux-vomica* with previously characterized CYPs from other organisms. CYP71 clade and candidate cytochromes P450 are highlighted in red.

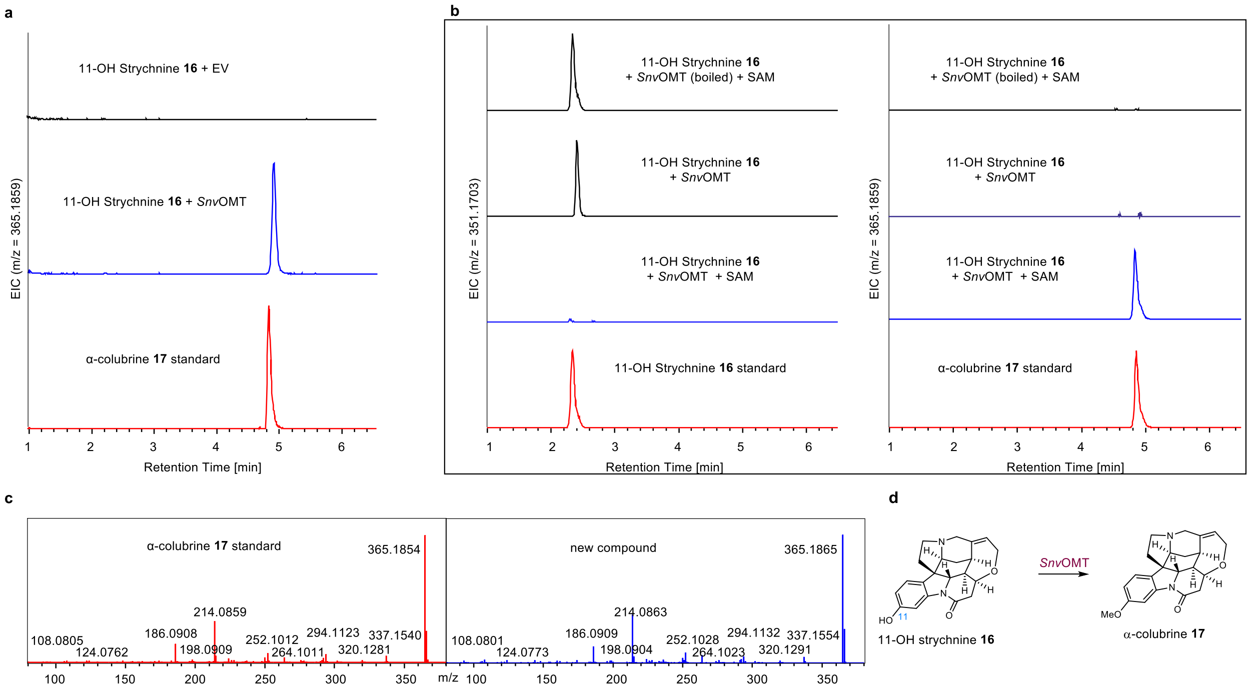

Fig. 26. Functional characterization of *Snv*OMT with 11-OH strychnine 16. a. Transient expression of *Snv*OMT in *N. benthamiana* with co-infiltration of 11-OH strychnine **16**. Extracted ion chromatograms for α-colubrine **17** (*m/z* $[M+H]^+$ = 365.1859 ± 0.05). This experiment was repeated three times with similar results. **b.** *In vitro* assays using purified *Snv*OMT from SoluBL21 *E. coli.* Extracted ion chromatograms for 11-OH strychnine **16** (*m/z* $[M+H]^+$ = 351.1703 ± 0.05, left) and α-colubrine **17** (*m/z* $[M+H]^+$ = 365.1859 ± 0.05, right). This experiment was repeated three times with similar results. **c**. MS/MS (20 to 50 eV) spectra of generated α-colubrine (blue) compared to synthetic standard (red). **d**. Reaction catalyzed by *Snv*OMT.

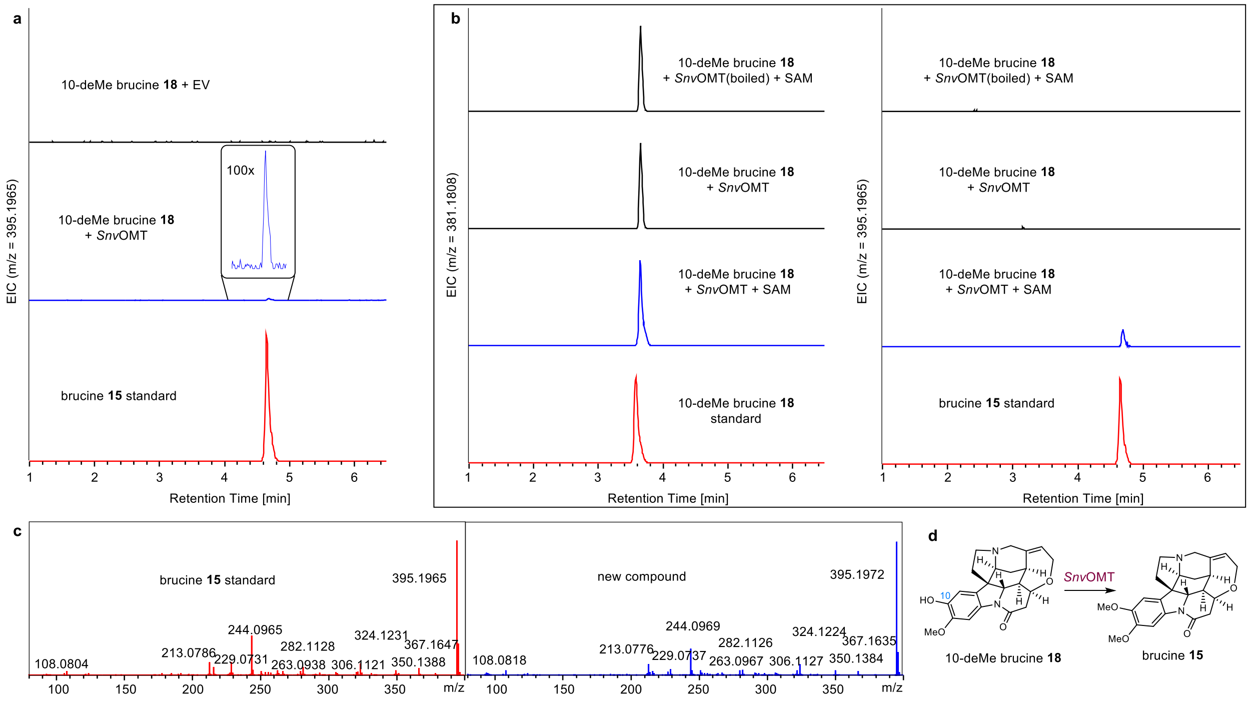

Fig. 27. Functional characterization of *Snv*OMT with 10-deMe brucine 18. a. Transient expression of *Snv*OMT in *N. benthamiana* with co-infiltration of 10-deMe brucine **18**. Extracted ion chromatograms for brucine **15** (*m/z* $[M+H]^+$ = 395.1965 ± 0.05). This experiment was repeated three times with similar results. **b**. *In vitro* assays using purified *Snv*OMT from SoluBL21 *E. coli.* Extracted ion chromatograms for 10-deMe brucine **18** (*m/z* $[M+H]^+$ = 381.1808 ± 0.05, left) and brucine **15** (*m/z* $[M+H]^+$ = 395.1965 ± 0.05, right). This experiment was repeated three times with similar results. **c**. MS/MS (20 to 50 eV) spectra of generated brucine **15** (blue) compared to synthetic standard (red). **d**. Reaction catalyzed by *Snv*OMT.

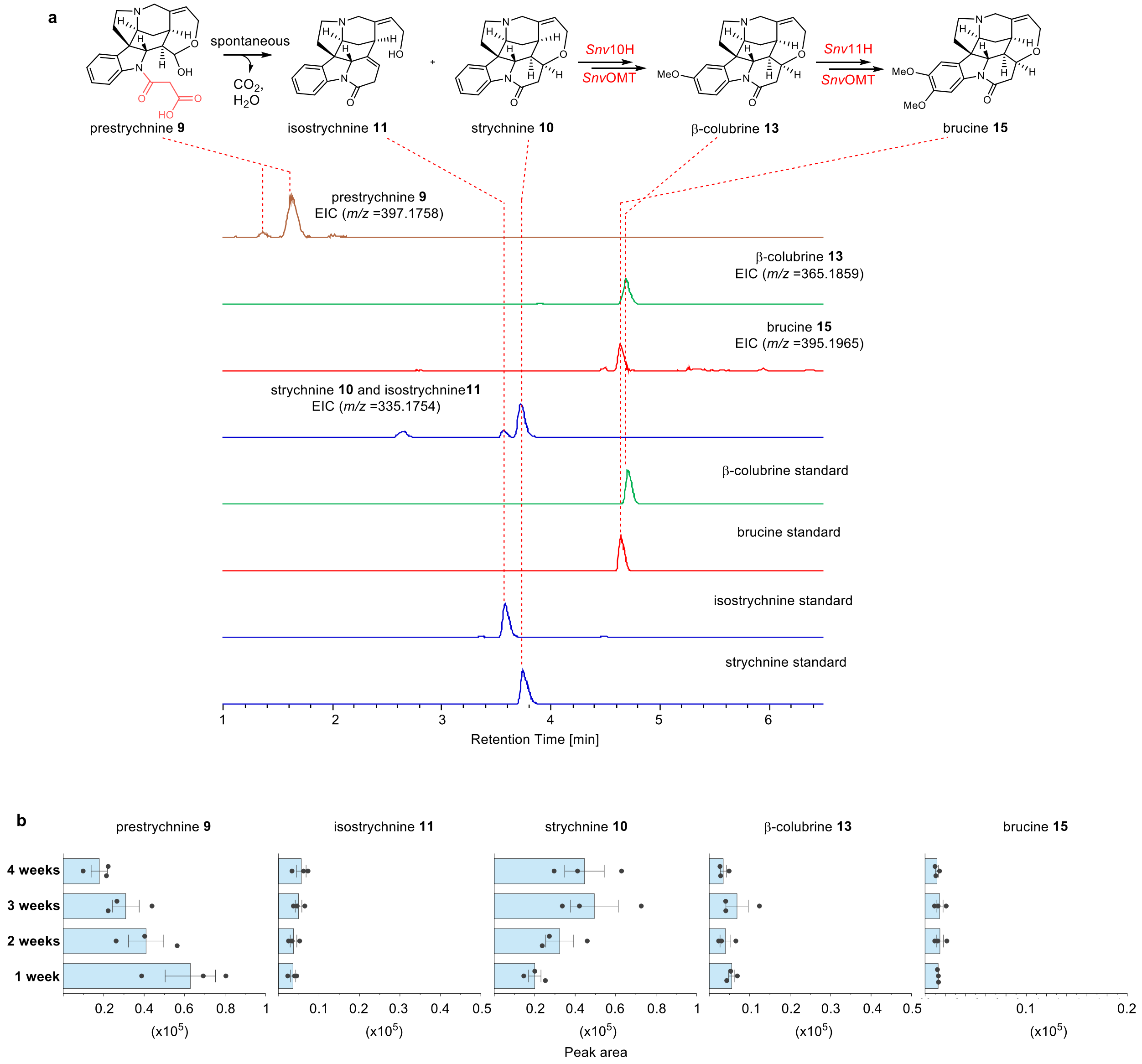

Fig. 28. Reconstitution of brucine 15 pathway in *N. benthamiana* from geissoschizine 1. a. Transient expression of *Snv*GO, *Snv*NS1, *Snv*NO, *Snv*WS, *Snv*AT, *Snv*10H, *Snv*OMT *Snv*11H, and AAE13 in *N. benthamiana* with co-infiltration of geissoschizine **1** and disodium malonate. Leaves were harvested at the 1st, 2nd, 3rd, and 4th weeks later after substrates infiltration. Extracted ion chromatograms for prestrychnine **9** (*m/z* $[M+H]^+$ = 397.1758 ± 0.05), strychnine **10** and isostrychnine **11** (*m/z* $[M+H]^+$ = 335.1754 ± 0.05), β-colubrine **13** (*m/z* $[M+H]^+$ = 365.1859 ± 0.05), and brucine **15** (*m/z* $[M+H]^+$ = 395.1965 ± 0.05). This experiment was repeated three times with similar results. **b**. Average LC-MS peak area (*n* = 3 biological replicates) of prestrychnine **9**, strychnine **10**, isostrychnine **11**, β-colubrine **13**, brucine **15** that produced in *N. benthamiana.* Date are presented as mean ± SEM (*n* = 3). Source data are provided with this paper.

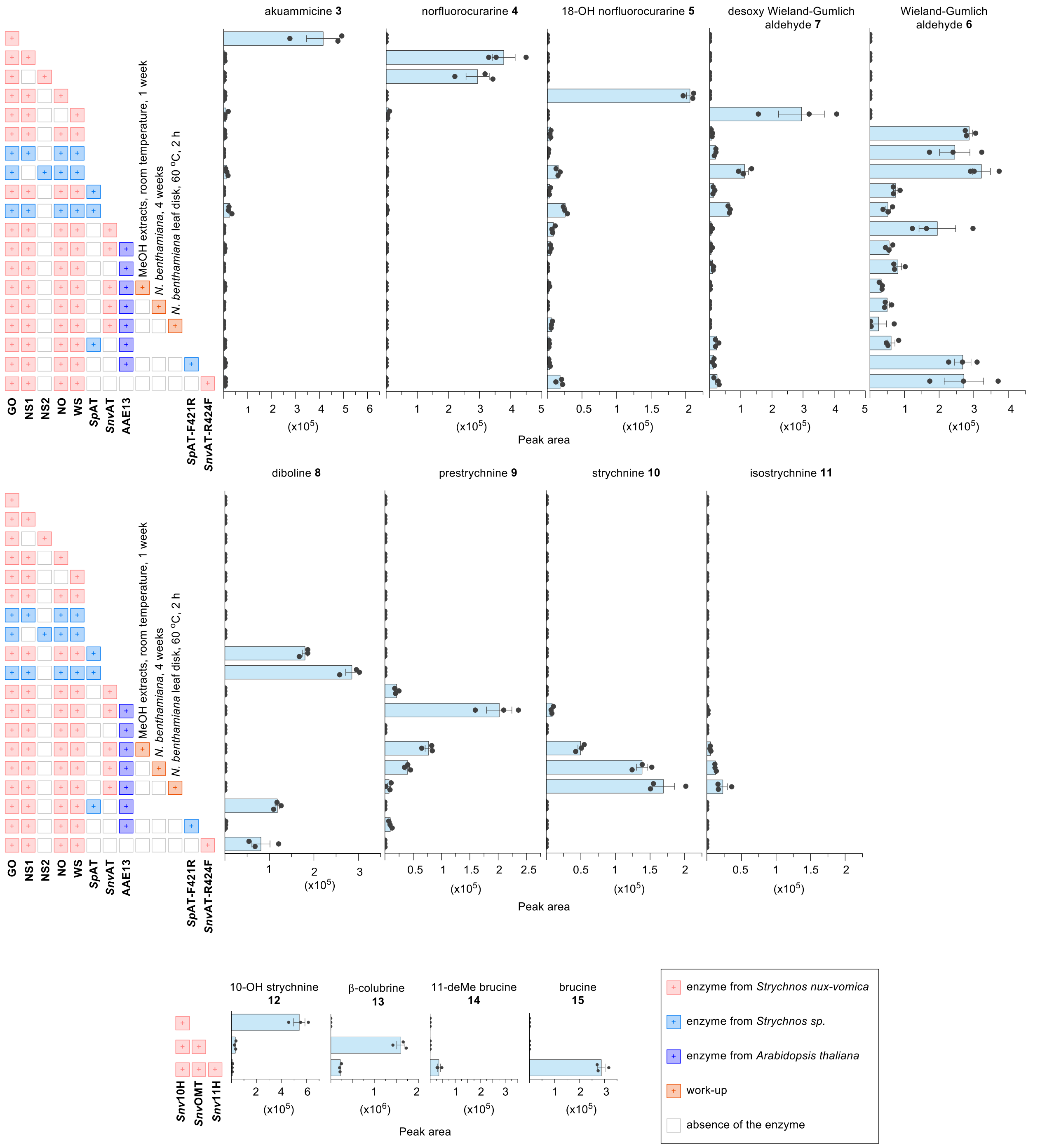

Fig. 29. Average LC-MS peak area (n = 3 biological replicates) of products produced in *N. benthamiana* after expression of indicated enzymes and substrates infiltration. Date are presented as mean ± SEM (n = 3). Source data are provided with this paper.

Synthesis of compounds:

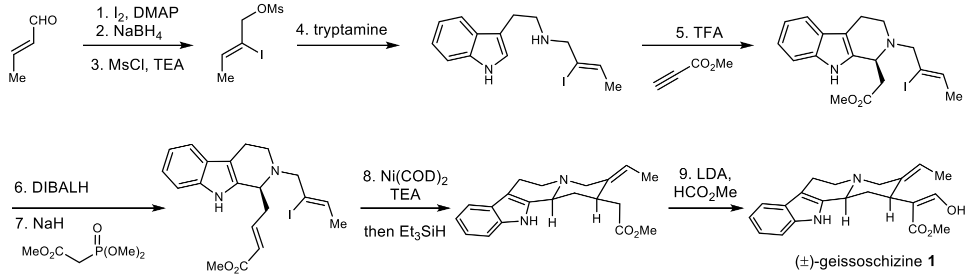

Fig. 30. Brief summary of the reported method for synthesis of (±)-geissoschizine

Approximately 200 mg (±)-geissoschizine **1** was synthesized according to the reported method.[52]

^{1}H-NMR (500 MHz, $CDCl_3$) δ 8.01 (bs, 1H), 7.85 (s, 1H), 7.47 (d, *J* = 7.7 Hz, 1H), 7.33 (d, *J* = 8.0 Hz, 1H), 7.16 (t, *J* = 7.4 Hz, 1H), 7.10 (t, *J* = 7.4 Hz, 1H), 5.41 (d, *J* = 6.5 Hz, 1H), 4.49 (d, *J* = 11.2 Hz, 1H), 3.99 – 3.91 (m, 1H), 3.83 (d, *J* = 5.9 Hz, 1H), 3.68 (s, 3H), 3.26 – 3.14 (m, 2H), 3.13 – 2.99 (m, 1H), 2.82 (d, *J* = 15.2 Hz, 1H), 2.76 – 2.68 (m, 1H), 2.62 (dt, *J* = 26.8, 10.6 Hz, 1H), 2.10 (t, *J* = 12.5 Hz, 1H), 1.81 (d, *J* = 5.9 Hz, 3H);
^{13}C-NMR (126 MHz, $CDCl_3$) δ 170.4, 161.2, 136.5, 133.1, 132.8, 126.5, 122.1, 121.8, 119.8, 118.3, 110.9, 108.1, 107.7, 59.1, 53.5, 51.1, 50.5, 33.8, 27.7, 20.4, 13.1;
HRMS (ESI) $[M+H]^+$ calculated for $C_{21}H_{25}N_2O_3$: 353.1859, found 353.1864.

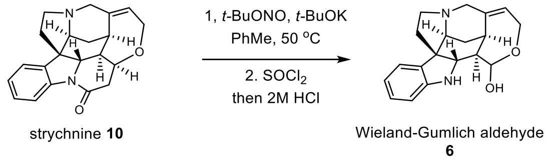

Wieland-Gumlich aldehyde **6** was synthesized according to the reported method.[53,54] To a stirred solution of strychnine **10** (3.13 g, 9.36 mmol) in anhydrous toluene (150 mL) was added *tert*-Butyl nitrite (25 mL, 210.2 mol) and *tert*-BuOK (2.5 g, 22.28 mmol) at room temperature. The reaction mixture was stirred at 50 °C for 2 h. After cooling to room temperature, saturated aqueous NH_4Cl solution (30 mL) was added and vigorous stirring was continued for 15 min. Water and toluene were removed in *vacuo*, the yellow residue was redissolved in $CHCl_3$/MeOH 4:1 (150 mL), and stirred for 30 min. The mixture was filtered and concentrated in *vacuo*. The resulting residue was added to thionyl chloride (15 mL) at 0 °C, and stirred at room temperature for 3 h. Thionyl chloride was removed in *vacuo*, and 2 M HCl (50 mL) was added. After stirring at 100 °C for 3 h, the mixture was basified with 20% ammonia at 0 °C, extracted with dichloromethane (5 x 50 mL), dried over anhydrous Na_2SO_4, filtered and concentrated in *vacuo*. The residue was purified on silica gel chromatography (DCM/MeOH/Et_3N = 100/10/1) to provide Wieland-Gumlich aldehyde **6** (1.3 g, 45% for 2 steps). The structure of **6** was confirmed by 2D-NMR. See P48-50.

Data of **6: ^{1}H-NMR** (400 MHz, $CDCl_3$) ^{1}H NMR (400 MHz, $CDCl_3$) δ 7.07 (td, *J* = 7.6, 1.1 Hz, 1H), 7.02 (t, *J* = 7.0 Hz, 1H), 6.79 (dd, *J* = 14.0, 6.6 Hz, 1H), 6.73 (d, *J* = 7.9 Hz, 0.85H), 6.62 (d, *J* = 7.7 Hz, 0.17H), 5.83 (s, 0.74H), 5.55 (s, 0.15H), 5.18 (s, 0.18H), 5.01 (d, *J* = 1.2 Hz, 1.36H), 4.77 (d, *J* = 15.0 Hz, 0.15H), 4.22 (dd, *J* = 14.2, 7.0 Hz, 0.79H), 3.98 – 3.84 (m, 2H), 3.79 (d, *J* = 10.6 Hz, 0.79H), 3.70 (d, *J* = 14.6 Hz, 1H), 3.52 (d, *J* = 10.5 Hz, 0.18H), 3.33 (s, 0.19H), 3.22 (dd, *J* = 9.7, 7.9 Hz, 1H), 2.86-2.79 (m, 1H), 2.71 – 2.61 (m, 1.69H), 2.27-2.16 (m, 0.83H), 2.08 (dd, *J* = 12.4, 6.2 Hz, 1H), 1.79 (d, *J* = 10.4 Hz, 0.8H), 1.67 – 1.50 (m, 2H), 1.43 (d, *J* = 14.1 Hz, 0.25H);

^{13}C-NMR (101 MHz, $CDCl_3$) δ 150.1, 150.0, 140.6, 132.4, 131.0, 128.1, 126.2, 122.1, 122.0, 119.7, 119.0, 111.8, 110.3, 105.7, 62.4, 60.9, 59.4, 59.0, 54.4, 54.1, 53.5, 53.1, 51.1, 51.0, 46.5, 39.0, 38.5, 32.0, 28.2, 26.2;
HRMS (ESI) $[M+H]^+$ calculated for $C_{19}H_{23}N_2O_2$: 311.1754, found 311.1749.

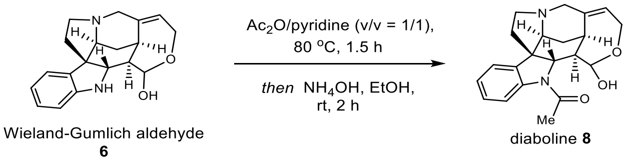

Diaboline **8** was synthesized according to the reported method.[58] To a stirred solution of Wieland-Gumlich aldehyde **6** (30 mg, 0.097 mmol) in anhydrous pyridine (0.25 mL) was added Ac_2O (0.25 mL) at room temperature. Then the solution was heated at 80 °C for 1.5 h. After cooling to room temperature, the solvents were removed in *vacuo.* The residue was redissolved in 20% ammonium/EtOH (1/1, 3 mL) and stirred at room temperature for 2 h. The solution was diluted with water (10 mL) and extracted with DCM (3 x 5 mL), dried over anhydrous Na_2SO_4, filtered and concentrated in *vacuo*. The residue was purified on silica gel chromatography (DCM/MeOH = 5/1) to provide diaboline **8** (20 mg, 59%).

Data of diaboline **8**: **^{1}H-NMR** (400 MHz, CD_2Cl_2) δ 7.87 (d, *J* = 7.9 Hz, 0.52H), 7.30 – 7.05 (m, 3.48H), 5.88 (s, 0.57H), 5.79 (s, 0.31H), 5.27 (s, 0.5H), 5.23 (s, 0.31H), 4.88 – 4.68 (m, 1.3H), 4.21 (d, *J* = 11.1 Hz, 0.54H), 3.98 (s, 1.11H), 3.74-3.64 (m, 2H), 3.40 (s, 1H), 3.38 – 3.29 (m, 1.6H), 2.91-2.83 (m, 1.5H), 2.80 – 2.69 (m, 1.3H), 2.41 – 2.27 (m, 3H), 2.26-2.16 (m, 1.2H), 1.98 – 1.87 (m, 1.39H), 1.71-1.63 (m, 1.25H), 1.52 (d, *J* = 10.9 Hz, 1H), 1.42-1.33 (m, 2H);
^{13}C-NMR (101 MHz, CD_2Cl_2) δ 170.1, 143.1, 142.8, 135.0, 128.2, 128.0, 126.9, 125.1, 123.1, 122.1, 119.9, 118.0, 94.0, 65.2, 64.7, 59.7, 59.4, 55.6, 52.9, 51.9, 51.9, 47.2, 46.2, 39.0, 38.9, 29.0, 28.9, 25.7, 25.1, 23.5, 23.4;
HRMS (ESI) $[M+H]^+$ $C_{21}H_{25}N_2O_3$: 353.1860, found 353.1858.

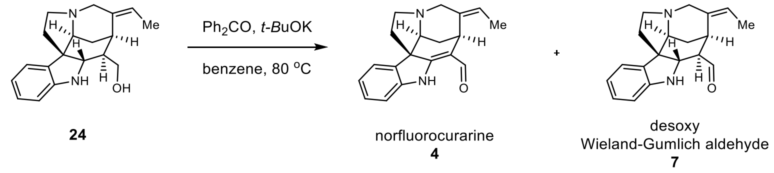

Norfluorocurarine **4** and desoxy Wieland-Gumlich aldehyde **7** were synthesized according to the reported method.[55-57] To a stirred solution of **24**[55,56] in anhydrous benzene (3 mL) was added benzophenone (111 mg, 0.608 mmol) and potassium *tert*-butoxide (56 mg, 0.499 mmol) at room temperature. The solution was degassed following the freeze-pump-thaw technique. Then the solution was heated at 80 °C for 1 h. After the mixture was cooled to room temperature, the reaction was quenched with saturated aqueous sodium bicarbonate (5 mL), extracted with dichloromethane (3 x 5 mL), dried over anhydrous Na_2SO_4, filtered and concentrated in *vacuo*. The residue was purified on silica gel chromatography (DCM/MeOH = 20/1 to 5/1) to provide norfluorocurarine **4** (6 mg, 15%) and desoxy Wieland-Gumlich aldehyde **7** (23 mg, 57%). The structures of **4** and **7** were confirmed by 2D-NMR. See P53-58.

Data of norfluorocurarine **4**: **^{1}H-NMR** (500 MHz, $CDCl_3$) δ 10.24 (s, 1H), 9.41 (s, 1H), 7.38 (d, *J* = 7.4 Hz, 1H), 7.24 (td, *J* = 7.8, 1.1 Hz, 1H), 7.02 (td, *J* = 7.6, 0.9 Hz, 1H), 6.94 (d, *J* = 7.8 Hz, 1H), 5.60 (dd, *J* = 13.7, 6.8 Hz, 1H), 4.45 (s, 1H), 4.24 – 4.15 (m, 1H), 3.80 (t, *J* = 13.6 Hz, 1H), 3.60 (td, *J* = 12.3, 6.0 Hz, 1H), 3.24 – 3.12 (m, 2H), 2.63 (ddd, *J* = 14.1, 3.8, 2.5 Hz, 1H), 2.54 – 2.44 (m, 1H), 2.08 – 1.98 (m, 1H), 1.66 (dt, *J* = 6.5, 2.0 Hz, 3H), 1.44 – 1.36 (m, 1H);

^{13}C-NMR (126 MHz, $CDCl_3$) δ 188.8, 167.3, 142.8, 134.4, 128.7, 125.8, 122.6, 122.1, 121.3, 111.3, 110.8, 61.5, 57.3, 55.9, 55.5, 45.0, 30.7, 30.2, 13.5;
HRMS (ESI) $[M+H]^+$ calculated for $C_{19}H_{21}N_2O$: 293.1648, found 293.1651.

Data of desoxy Wieland-Gumlich **7**: **^{1}H-NMR** (400 MHz, $CDCl_3$) δ 9.69 (s, 1H), 7.09-7.04 (dd, *J* = 10.1, 6.3 Hz, 2H), 6.76 (t, *J* = 7.4 Hz, 1H), 6.61 (d, *J* = 7.6 Hz, 1H), 5.58 – 5.48 (m, 1H), 4.08 (s, 1H), 3.93 (d, *J* = 9.5 Hz, 1H), 3.66 (s, 1H), 3.59 (d, *J* = 14.4 Hz, 1H), 3.35 (d, *J* = 3.3 Hz, 1H), 3.30 – 3.21 (m, 1H), 3.05 (d, *J* = 14.6 Hz, 1H), 2.89 – 2.81 (m, 1H), 2.55 (dd, *J* = 12.8, 7.6 Hz, 1H), 2.49 (dd, *J* = 9.5, 3.7 Hz, 1H), 2.15 (dt, *J* = 13.6, 3.6 Hz, 1H), 1.88 – 1.81 (m, 2H), 1.62 (dd, *J* = 7.0, 1.8 Hz, 3H);
^{13}C-NMR (101 MHz, $CDCl_3$) δ 203.6, 148.6, 133.1, 131.6, 128.3, 122.0, 119.2, 109.6, 63.8, 61.2, 60.2, 57.4, 53.6, 53.3, 50.8, 41.4, 28.1, 13.3;
HRMS (ESI) $[M+H]^+$ calculated for $C_{19}H_{23}N_2O$: 295.1804, found 295.1802.

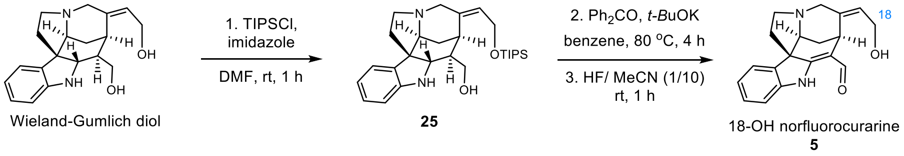

To a stirred solution of Wieland-Gumlich diol[55,56] (50 mg, 0.16 mmol) in anhydrous DMF (1 mL) was added imidazole (109 mg, 1.6 mmol) and triisopropylsilylchloride (34.3 µL, 0.16 mmol) at 0 °C. After being stirred at room temperature for 1 h, the reaction was quenched with saturated aqueous sodium bicarbonate (5 mL), extracted with dichloromethane (3 x 5 mL), dried over anhydrous Na_2SO_4, filtered and concentrated in *vacuo*. The resulting residue (50 mg) was used directly in the next step.

To a stirred solution of above residue in anhydrous benzene (5 mL) was added benzophenone (90 mg, 0.0961 mmol) and potassium *tert*-butoxide (48 mg, 0.428 mmol) at room temperature. The solution was degassed following the freeze-pump-thaw technique and the solution was then heated at 80 °C for 4 h. After the mixture was cooled to room temperature, the reaction was quenched with saturated aqueous sodium bicarbonate (5 mL), extracted with dichloromethane (3 x 5 mL), dried over anhydrous Na_2SO_4, filtered and concentrated in *vacuo*. The resulting crude was dissolved in 48% aq. HF/MeCN (10/1, 2.2 mL) and stirred at room temperature for 1 h. Then the mixture was poured into saturated aqueous sodium bicarbonate (10 mL), extracted with dichloromethane (3 x 5 mL), dried over anhydrous Na_2SO_4, filtered and concentrated in *vacuo*. The residue was purified on silica gel chromatography (DCM/MeOH = 5/1) to provide 18-OH norfluorocurarine **5** (6 mg, 12% for 3 steps). The structure of **5** was confirmed by 2D-NMR. See P59-61.

Data of 18-OH norfluorocurarine **5**: **^{1}H-NMR** (400 MHz, $CDCl_3$) δ 10.31 (s, 1H), 9.35 (s, 1H), 7.34 (d, *J* = 7.3 Hz, 1H), 7.23 (td, *J* = 7.7, 1.1 Hz, 1H), 7.01 (td, *J* = 7.5, 0.9 Hz, 1H), 6.94 (d, *J* = 7.8 Hz, 1H), 5.61 (s, 1H), 4.26 (s, 2H), 4.25 (s, 1H), 4.12 (d, *J* = 15.7 Hz, 1H), 3.83 (s, 1H), 3.66 (s, 1H), 3.46 (td, *J* = 12.6, 5.8 Hz, 1H), 3.15 (dd, *J* = 12.0, 6.1 Hz, 1H), 3.09 (d, *J* = 15.8 Hz, 1H), 2.65 – 2.57 (m, 1H), 2.44 (td, *J* = 12.6, 6.6 Hz, 1H), 1.95 (dd, *J* = 12.3, 5.1 Hz, 2H), 1.34 (d, *J* = 13.8 Hz, 1H);
^{13}C-NMR (101 MHz, $CDCl_3$) δ 188.1, 168.5, 142.6, 139.6, 136.0, 128.5, 127.4, 122.6, 121.2, 111.3, 110.8, 61.4, 58.5, 57.9, 56.1, 56.1, 45.6, 30.9, 30.5;
HRMS (ESI) $[M+H]^+$ $[M+H]^+$ calculated for $C_{19}H_{21}N_2O_2$: 309.1598, found 309.1597.

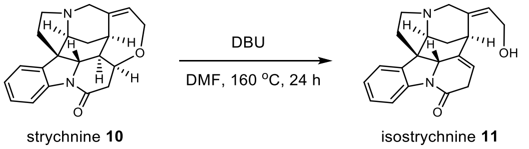

Isostrychnine **11** was synthesized according to the reported method.[59] A stirred solution of strychnine **10** (167 mg, 0.5 mmol) and DBU (745 µL, 5 mmol) in DMF (4 mL) was degassed by bubbling argon for 1 h. The reaction mixture was stirred at 160 °C for 24 h. After cooling to room temperature, the solvents were removed in *vacuo*. The residue was purified on silica gel chromatography (DCM/MeOH = 50/1) to provide isostrychnine **11** (24 mg, 14%). The structure of **11** was confirmed by 2D-NMR. See P62-64.

Data of isostrychnine **11**: **^{1}H-NMR** (400 MHz, $CDCl_3$) δ 8.15 (d, *J* = 7.8 Hz, 1H), 7.29 – 7.21 (m, 2H), 7.10 (td, *J* = 7.5, 1.0 Hz, 1H), 5.91 – 5.85 (m, 1H), 5.66 (t, *J* = 6.2 Hz, 1H), 4.35 – 4.26 (m, 3H), 3.83 (s, 1H), 3.69 (d, *J* = 14.6 Hz, 1H), 3.62 (s, 1H), 3.39 – 3.30 (m, 1H), 3.13 (dd, *J* = 17.5, 6.6 Hz, 1H), 3.06-3.0 (m, 2H), 2.99 – 2.89 (m, 1H), 2.33 (dt, *J* = 13.6, 8.1 Hz, 1H), 2.25 – 2.15 (m, 2H), 1.51 (d, *J* = 14.2 Hz, 1H);
^{13}C-NMR (101 MHz, $CDCl_3$) 168.3, 141.7, 141.4, 136.3, 133.7, 128.6, 128.2, 124.3, 122.7, 120.9, 114.7, 66.6, 62.7, 58.3, 53.3, 52.4, 52.3, 45.4, 36.9, 34.5, 25.2;
HRMS (ESI) $[M+H]^+$ calculated for $C_{21}H_{23}N_2O_2$: 335.1754, found 335.1751.

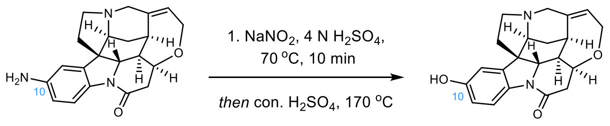

10-NH$_2$ strychnine **26** 10-OH strychnine **12**

10-OH strychnine **12** was synthesized according to the reported method.[60] To 10-NH_2 strychnine **26**[60] (70 mg, 0.2 mmol) in 4N H_2SO_4 (0.5 mL) was added a solution of $NaNO_2$ (16 mg, 0.24 mmol) in water (0.2 mL) and stirred at 70 °C for 10 minutes to form the diazonium salt. The diazonium solution was then added to a stirred solution of concentrated H_2SO_4 (1 mL), water (0.4 mL) and Na_2SO_4 (1 g) heated to 170 °C. After 30 minutes the reaction was cooled to room temperature and basified with 20% NH_4OH (5 mL), extracted with DCM (3 x 5 mL), dried over anhydrous Na_2SO_4, filtered and concentrated in *vacuo*. The residue was purified on silica gel chromatography (DCM/MeOH = 5/1) to provide 10-OH strychnine **12** (24 mg, 34%).

Data of 10-OH strychnine **12**: **^{1}H-NMR** (400 MHz, $CDCl_3$) δ 8.00 (d, *J* = 8.7 Hz, 1H), 6.80 (dd, *J* = 8.7, 2.4 Hz, 1H), 6.66 (d, *J* = 2.4 Hz, 1H), 6.01 (s, 1H), 4.31 (dt, *J* = 8.3, 3.1 Hz, 1H), 4.21 – 4.02 (m, 4H), 3.90 (d, *J* = 10.5 Hz, 1H), 3.75 (d, *J* = 14.4 Hz, 1H), 3.23 (t, *J* = 8.8 Hz, 3H), 3.13 (dd, *J* = 17.4, 8.5 Hz, 1H), 2.97 – 2.87 (m, 1H), 2.84 (d, *J* = 14.7 Hz, 1H), 2.65 (dd, *J* = 17.4, 3.2 Hz, 1H), 2.44 (dt, *J* = 14.5, 4.3 Hz, 1H), 1.97 (dd, *J* = 13.0, 5.8 Hz, 2H), 1.87 – 1.76 (m, 2H), 1.61 (d, *J* = 14.5 Hz, 1H), 1.35 (dt, *J* = 10.4, 3.0 Hz, 1H);
^{13}C-NMR (101 MHz, $CDCl_3$) δ 168.4, 154.7, 138.8, 134.8, 133.0, 129.5, 118.1, 116.6, 108.7, 77.8, 64.5, 60.2, 60.2, 52.4, 52.2, 50.2, 48.2, 43.1, 42.3, 31.5, 26.8;
HRMS (ESI) $[M+H]^+$ calculated for $C_{21}H_{23}N_2O_2$: 351.1709, found 351.1704.

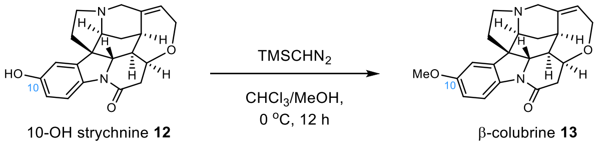

10-OH strychnine **12** β-colubrine **13**

β-colubrine **13** was synthesized according to the reported method.[60] To a stirred solution of 10-OH strychnine **12** (1.7 mg, 0.0049 mmol) in $CHCl_3$/MeOH (v/v = 1/1, 1 mL) was added $TMSCHN_2$ (0.6 M in hexane, 160 µL, 0.097 mmol) at 0 °C. After stirring at the same temperature for 12 h, the solvents were removed in *vacuo*. The residue was purified on silica gel chromatography (DCM/MeOH = 10/1) to provide β-colubrine strychnine **13** (1.5 mg, 83%). The structure of **13** was confirmed by 2D-NMR. See P66-68.

Data of β-colubrine **13**: **^{1}H-NMR** (400 MHz, $CDCl_3$) δ 8.01 (d, *J* = 8.8 Hz, 1H), 6.81 (dd, *J* = 8.8, 2.6 Hz, 1H), 6.76 (d, *J* = 2.5 Hz, 1H), 6.06 (s, 1H), 4.30 (dt, *J* = 8.4, 3.2 Hz, 1H), 4.18 (dd, *J* = 13.9, 7.0 Hz, 1H), 4.13 (s, 1H), 4.07 (dd, *J* = 13.5, 5.9 Hz, 1H), 3.90 (d, *J* = 10.6 Hz, 1H), 3.85 (d, *J* = 15.7 Hz, 1H), 3.79 (s, 3H), 3.45 (s, 1H), 3.22 (s, 1H), 3.13 (dd, *J* = 17.5, 8.5 Hz, 1H), 2.97 (d, *J* = 7.9 Hz, 1H), 2.91 (d, *J* = 14.6 Hz, 1H), 2.66 (dd, *J* =

17.5, 3.3 Hz, 1H), 2.45 – 2.36 (m, 1H), 2.03 – 1.94 (m, 2H), 1.55 (d, *J* = 14.3 Hz, 1H), 1.31 (dt, *J* = 10.3, 3.2 Hz, 1H);
^{13}C-NMR (101 MHz, $CDCl_3$) δ 168.6, 157.1, 137.6, 135.8, 132.6, 130.4, 117.1, 113.9, 108.2, 77.7, 64.4, 60.5, 60.0, 55.8, 52.5, 52.1, 50.3, 47.8, 42.2, 42.0, 31.3, 26.3;
HRMS (ESI) $[M+H]^+$ calculated for $C_{22}H_{25}N_2O_3$: 365.1860, found 365.1860.

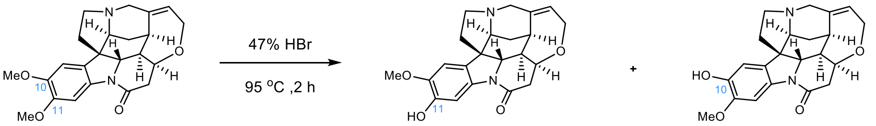

brucine **15** 11-deMe brucine **14** 16% 10-deMe brucine **18** 67%

11-deMe brucine **14** and 10-deMe brucine **18** were synthesized according to the reported method.[61] The solution of brucine **15** (200 mg, 0.507 mmol) in 47% HBr (1 mL) was heated to 95 °C for 2 h. After cooling to room temperature, the solution was basified with 20% ammonia (5 mL), extracted with $CHCl_3$ (3 x 5 mL), dried over anhydrous Na_2SO_4, filtered and concentrated in *vacuo*. The residue was purified by prepared TLC (DCM/MeOH/Et_3N = 100/5/1) to provide 10-deMe brucine **18** (131 mg, 67%) and 11-deMe brucine **14** (32 mg, 16%). The structures of **14** and **18** were confirmed by 2D-NMR. See P69-74.

Data of 11-deMe brucine **14**: **^{1}H-NMR** (500 MHz, $CDCl_3$) δ 7.78 (s, 1H), 6.73 (s, 1H), 6.20 (s, 1H), 4.32 (dt, *J* = 8.4, 3.2 Hz, 1H), 4.28 (s, 1H), 4.21 (dd, *J* = 14.0, 7.0 Hz, 1H), 4.08 (dd, *J* = 13.5, 5.7 Hz, 2H), 3.99 (d, *J* = 14.2 Hz, 1H), 3.91 (d, *J* = 2.0 Hz, 1H), 3.89 (s, 3H), 3.70 – 3.63 (m, 1H), 3.28 (s, 1H), 3.14 (dd, *J* = 17.6, 8.5 Hz, 1H), 3.09 – 3.04 (m, 2H), 2.65 (dd, *J* = 17.5, 3.2 Hz, 1H), 2.44 (dt, *J* = 15.0, 4.2 Hz, 1H), 2.03 (td, *J* = 13.2, 7.1 Hz, 2H), 1.64 (d, *J* = 15.0 Hz, 1H), 1.35 (dt, *J* = 10.5, 3.1 Hz, 1H);
^{13}C-NMR (101 MHz, $CDCl_3$) δ 168.5, 146.8, 144.2, 136.0, 132.7, 120.1, 104.5, 104.0, 77.6, 64.2, 60.4, 59.6, 56.6, 52.3, 52.1, 50.1, 47.5, 42.1, 41.5, 31.0, 29.7, 25.8;
HRMS (ESI) $[M+H]^+$ calculated for $C_{22}H_{25}N_2O_4$: 381.1809, found 381.1807.

Data of 10-deMe brucine **18**: **^{1}H-NMR** (400 MHz, $CDCl_3$) δ 7.80 (s, 1H), 6.66 (s, 1H), 5.96 (t, *J* = 6.8 Hz, 1H), 4.29 (dt, *J* = 8.4, 3.2 Hz, 1H), 4.15 (dd, *J* = 13.8, 7.0 Hz, 1H), 4.05 (dd, *J* = 13.8, 6.0 Hz, 1H), 3.97 (s, 1H), 3.92 (s, 3H), 3.82 (d, *J* = 10.4 Hz, 1H), 3.73 (d, *J* = 14.6 Hz, 1H), 3.26 – 3.19 (m, 1H), 3.17 (s, 1H), 3.11 (dd, *J* = 17.4, 8.5 Hz, 1H), 2.89 – 2.77 (m, 2H), 2.67 (dd, *J* = 17.4, 3.2 Hz, 1H), 2.38 (dt, *J* = 14.4, 4.3 Hz, 1H), 1.78 (dd, *J* = 10.5, 5.9 Hz, 2H), 1.53 (d, *J* = 14.5 Hz, 1H), 1.29 (dd, *J* = 7.4, 2.9 Hz, 1H);
^{13}C-NMR (101 MHz, $CDCl_3$) δ 168.6, 147.5, 143.5, 139.2, 134.8, 128.9, 123.2, 108.0, 100.9, 77.8, 64.5, 60.1, 59.9, 56.2, 52.4, 52.0, 50.1, 48.2, 42.5 42.3, 31.4, 26.6;
HRMS (ESI) $[M+H]^+$ calculated for $C_{22}H_{25}N_2O_4$: 381.1809, found 381.1813.

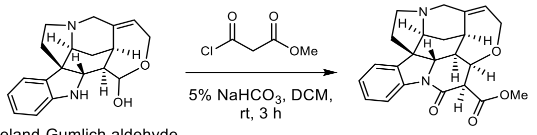

Wieland-Gumlich aldehyde **6** **19**

To a stirred solution of Wieland-Gumlich aldehyde **6** (50 mg, 0.161 mmol) in DCM (3 mL) and 5% aqueous $NaHCO_3$ (2 mL) was added methyl malonyl chloride (52 µL, 0.483 mmol) at room temperature. After stirring at the same temperature for 3 h, the reaction was diluted by water (5 mL), extracted with DCM (3 x 5 mL), dried over anhydrous Na_2SO_4, filtered and concentrated in *vacuo*. The residue was purified on silica gel chromatography (DCM/MeOH = 10/1) to **19** (15 mg, 24%). The structure of **19** was confirmed by 2D-NMR. See P75-77.

Data of **19**: **^{1}H-NMR** (400 MHz, $CDCl_3$) δ 8.05 (d, J = 8.1 Hz, 1H), 7.31 – 7.27 (m, 1H), 7.22 (d, J = 7.0 Hz, 1H), 7.17 – 7.11 (m, 1H), 6.04 (s, 1H), 4.71 (t, J = 4.0 Hz, 1H), 4.20 – 4.06 (m, 3H), 3.88 (s, 3H), 3.85 (s, 1H), 3.81

(d, J = 5.3 Hz, 1H), 3.69 (d, J = 4.2 Hz, 1H), 3.39-3.25 (m, 1H), 3.27 (s, 1H), 3.01 – 2.85 (m, 2H), 2.43 – 2.36 (m, 1H), 2.08-1.95 (m, 2H), 1.54 (d, J = 14.6 Hz, 1H), 1.40 (dt, J = 10.4, 3.4 Hz, 1H);
^{13}C-NMR (101 MHz, $CDCl_3$) δ 169.2, 165.7, 141.6, 138.41, 131.24, 130.25, 129.3, 125.0, 122.4, 116.9, 79.6, 64.1, 60.5, 60.4, 58.9, 52.9, 52.3, 51.9, 50.4, 47.7, 42.0, 30.8, 26.0;
HRMS (ESI) $[M+H]^+$ calculated for $C_{23}H_{25}N_2O_4$: 393.1809, found 393.1808.

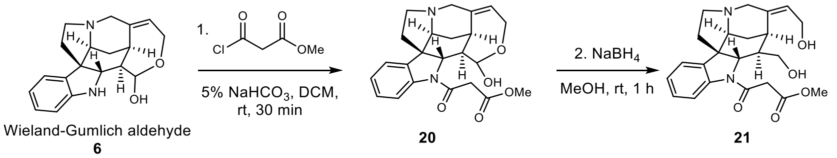

To a stirred solution of Wieland-Gumlich aldehyde **6** (50 mg, 0.161 mmol) in DCM (3 mL) and 5% aqueous $NaHCO_3$ (2 mL) was added methyl malonyl chloride (52 µL, 0.483 mmol) at room temperature. After stirring at the same temperature for 30 min, the reaction was diluted by water (5 mL), extracted with DCM (3 x 5 mL), dried over anhydrous Na_2SO_4, filtered and concentrated in *vacuo*. The resulting residue was redissolved in MeOH (5 mL), $NaBH_4$ (6.1 mg, 0.161 mmol) was added. After 1 h, the reaction was quenched with saturated aqueous $NaHCO_3$ (5 mL), extracted with DCM (3 x 5 mL), dried over anhydrous Na_2SO_4, filtered and concentrated in *vacuo*. The residue was purified on silica gel chromatography (DCM/MeOH = 10/1) to **21** (8 mg, 12%). The structure of **21** was confirmed by 2D-NMR. See P78-80.
Note: intermediate **20** is unstable, it will covert to **19** during silica gel or prepared HPLC purification.

Data of **21**: **^{1}H-NMR** (400 MHz, $CDCl_3$) δ 7.30 – 7.24 (m, 2H), 7.23-7.19 (m, 1H), 7.08 (d, *J* = 7.9 Hz, 1H), 5.78 (t, *J* = 7.2 Hz, 1H), 4.73 (d, *J* = 11.2 Hz, 1H), 4.31 (dd, *J* = 11.9, 9.2 Hz, 1H), 3.92 (dd, *J* = 12.0, 6.4 Hz, 1H), 3.89 – 3.81 (m, 1H), 3.78 – 3.72 (m, 4H), 3.71 – 3.64 (m, 2H), 3.61 (s, 1H), 3.31 – 3.17 (m, 2H), 3.06 (d, *J* = 3.2 Hz, 1H), 2.90 (ddd, *J* = 12.5, 8.7, 4.1 Hz, 1H), 2.54 – 2.41 (m, 1H), 2.04 (dt, *J* = 13.5, 3.2 Hz, 1H), 1.85 (ddd, *J* = 14.0, 10.1, 4.0 Hz, 1H), 1.69 – 1.59 (m, 2H);
^{13}C-NMR (101 MHz, $CDCl_3$) δ 167.6, 165.8, 139.7, 139.3, 138.9, 128.0, 125.9, 125.0, 122.8, 116.6, 68.9, 63.4, 61.3, 58.1, 57.4, 53.8, 52.7, 52.3, 47.3, 41.7, 41.6, 31.3, 28.3;
HRMS (ESI) $[M+H]^+$ calculated for $C_{23}H_{28}N_2O_5$: 413.2071, found 413.2073.

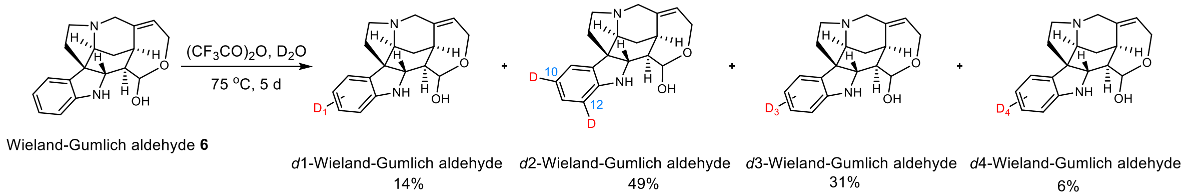

Deuterium labeled Wieland-Gumlich aldehyde was synthesized according to the reported method.[12] A solution of 100 µL D_2O and 700 µL $(CF_3CO)_2O$ was added to Wieland-Gumlich aldehyde **6** (100 mg, 0.322 mmol) and the solution was degassed following the freeze-pump-thaw technique. Then the solution was heated at 75 °C for 3 days. After the mixture was cooled to room temperature, the solvents carefully removed under vacuum. The residue was dissolved in 5 ml H_2O, basified with saturated aqueous sodium bicarbonate (5 mL), extracted with dichloromethane (5 x 5 mL), dried over anhydrous Na_2SO_4, filtered and concentrated in *vacuo*. The residue was purified on silica gel chromatography (DCM/MeOH/Et_3N = 100/10/1) to provide deuterium labeled Wieland-Gumlich aldehyde (23 mg, 23%). Based on the MS spectrum, the ratio of *d*1: *d*2: *d*3: *d*4 = 14:49:31:6 (See Extended Data Fig. 7b). The major component *d*2-Wieland-Gumlich aldehyde is characterized by NMR and HRMS. The proton at positions 10 and 12 were deuterated.

Data of the major component *d*2-Wieland-Gumlich aldehyde: **^{1}H-NMR** (400 MHz, $CDCl_3$) ^{1}H NMR (400 MHz, $CDCl_3$) δ 7.10 – 7.07 (m, 1H), 7.06 – 7.01 (m, 1H), 6.84-6.74 (m, 0.22H), 5.83 (s, 0.81H), 5.55 (s, 0.15H), 5.17

(s, 0.15H), 5.00 (s, 0.8H), 4.87 (s, 0.711H), 4.76 (d, J = 15.6 Hz, 0.21H), 4.23 (dd, J = 14.2, 7.0 Hz, 0.84H), 4.11 (s, 0.25H), 4.00 – 3.87 (m, 2.18H), 3.81 (d, J = 8.2 Hz, 1.05H), 3.73-3.65 (m, 1.14H), 3.51 (s, 0.17H), 3.32 (s, 0.21H), 3.30 – 3.19 (m, 1.15H), 2.82 (ddd, J = 12.5, 10.2, 6.3 Hz, 1.15H), 2.75 – 2.56 (m, 2H), 2.28-2.23 (m, 1H), 2.07 (dd, J = 12.5, 6.2 Hz, 1H), 1.81 (d, J = 10.6 Hz, 0.42H), 1.70 – 1.50 (m, 2H), 1.44 (d, J = 14.4 Hz, 0.25H);
^{13}C NMR (101 MHz, $CDCl_3$) δ 149.7, 149.6, 140.6, 133.0, 131.0, 128.0, 127.9, 126.2, 122.0, 121.9, 120.3, 112.4, 112.0, 105.6, 62.5, 60.8, 60.7, 59.5, 59.1, 54.7, 54.2, 53.6, 53.1, 51.2, 51.1, 46.4, 39.0, 38.5, 32.1, 32.1, 28.1, 26.3, 26.2;
HRMS (ESI) $[M+H]^+$ calculated for $C_{19}H_{21}D_2N_2O_2$: 313.1880, found 313.1873.

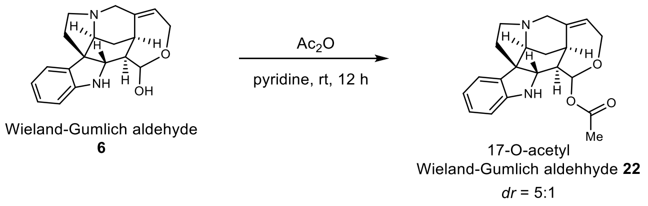

To a stirred solution of Wieland-Gumlich aldehyde **6** (20 mg, 0.0645 mmol) in anhydrous pyridine (0.5 mL) was added Ac_2O (6.4 µL, 0.0645 mmol) at room temperature. Then the solution was stirred at room temperature for 12 h. The solvent was removed in *vacuo.* The residue was purified on preparative TLC plate silica gel (DCM/MeOH/Et_3N = 100/10/1) to provide 17-O-acetyl Wieland-Gumlich aldehhyde **22** (8.8 mg, 39%). The structure was confirmed by 2D-NMR. See P82-83.

Data of 17-O-acetyl Wieland-Gumlich aldehyde **22**: **^{1}H-NMR** (400 MHz, $CDCl_3$) δ 7.12 (t, J = 6.8 Hz, 1H), 6.86-6.70 (m, 1H), 6.75-6.66 (m, 1H), 6.09 (s, 1H), 5.89 (s, 0.32H), 5.24 (s, 0.47H), 5.07 (s, 1H), 4.81-4.77 (m, 0.29H), 4.38 – 4.21 (m, 2H), 4.03-3.96 (m, 2H), 3.87 (d, J = 10.4 Hz, 1H), 3.77 – 3.56 (m, 1H), 3.08 – 2.93 (m, 2H), 2.80 (s, 1H), 2.39 (d, J = 15.1 Hz, 1H), 2.22-2.17 (m, 1H), 2.05 (s, 3H), 1.88 (d, J = 10.3 Hz, 1H), 1.84 – 1.74 (m, 1H), 1.74 – 1.59 (m, 1H);
^{13}C-NMR (101 MHz, $CDCl_3$) δ 176.8, 149.4, 135.9, 134.0, 131.3, 129.1, 122.2, 120.5, 112.2, 104.9, 62.2, 60.5, 59.7, 54.7, 52.7, 50.7, 45.9, 37.7, 31.4, 25.2, 22.6;
HRMS (ESI) $[M+H]^+$ $C_{21}H_{25}N_2O_3$: 353.1860, found 353.1859.

NMR spectra of compounds

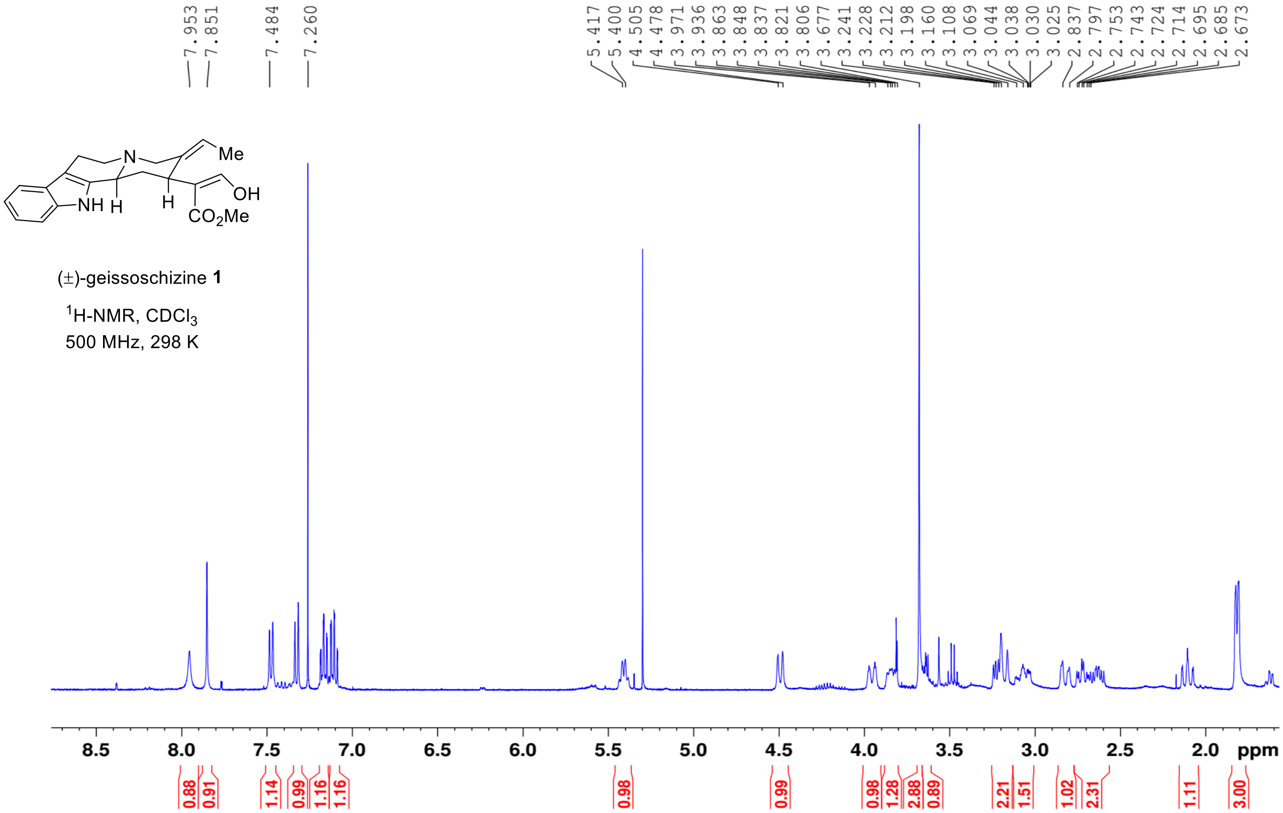

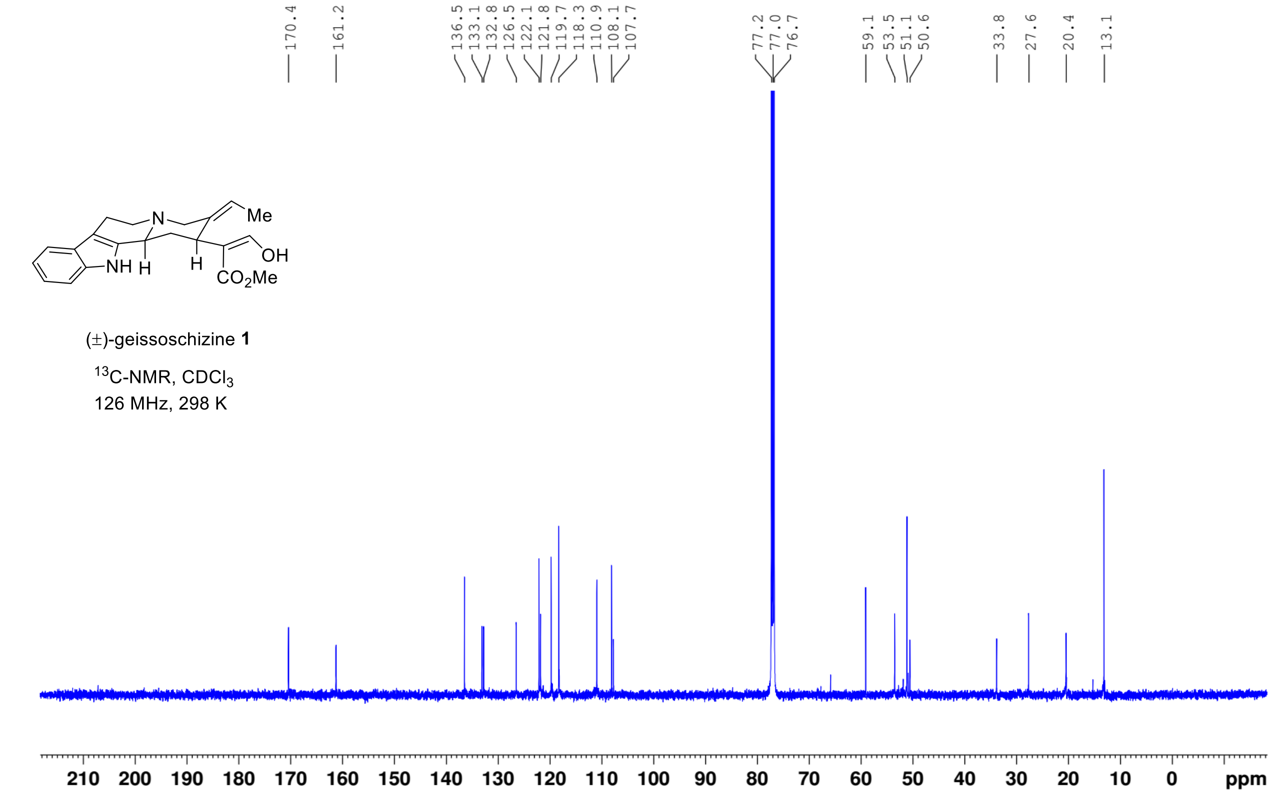

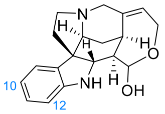

Wieland-Gumlich aldehyde **6**

^{1}H-NMR, $CDCl_3$
400 MHz, 298 K

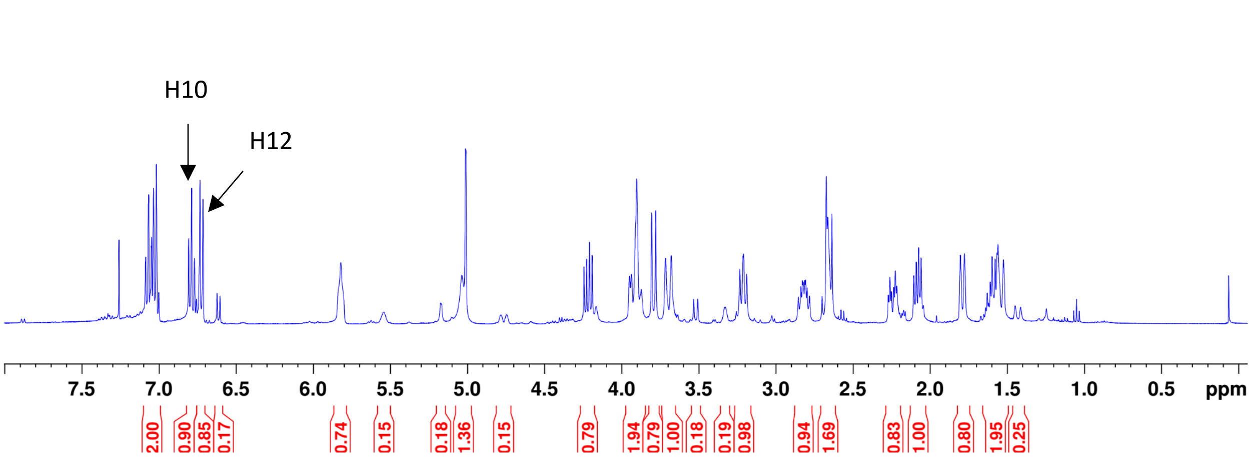

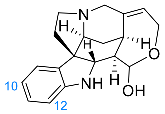

Wieland-Gumlich aldehyde **6**

^{13}C-NMR, $CDCl_3$
101 MHz, 298 K

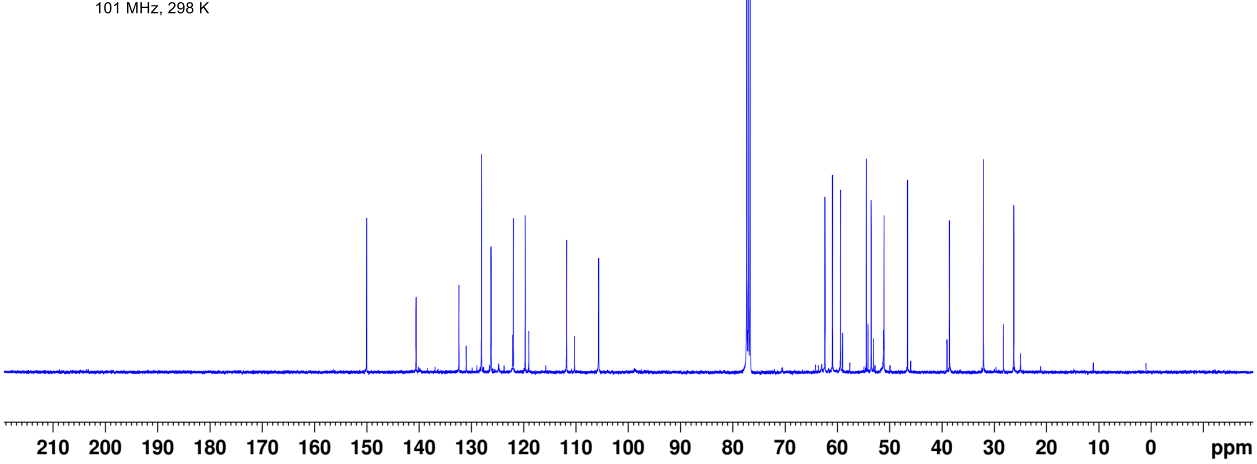

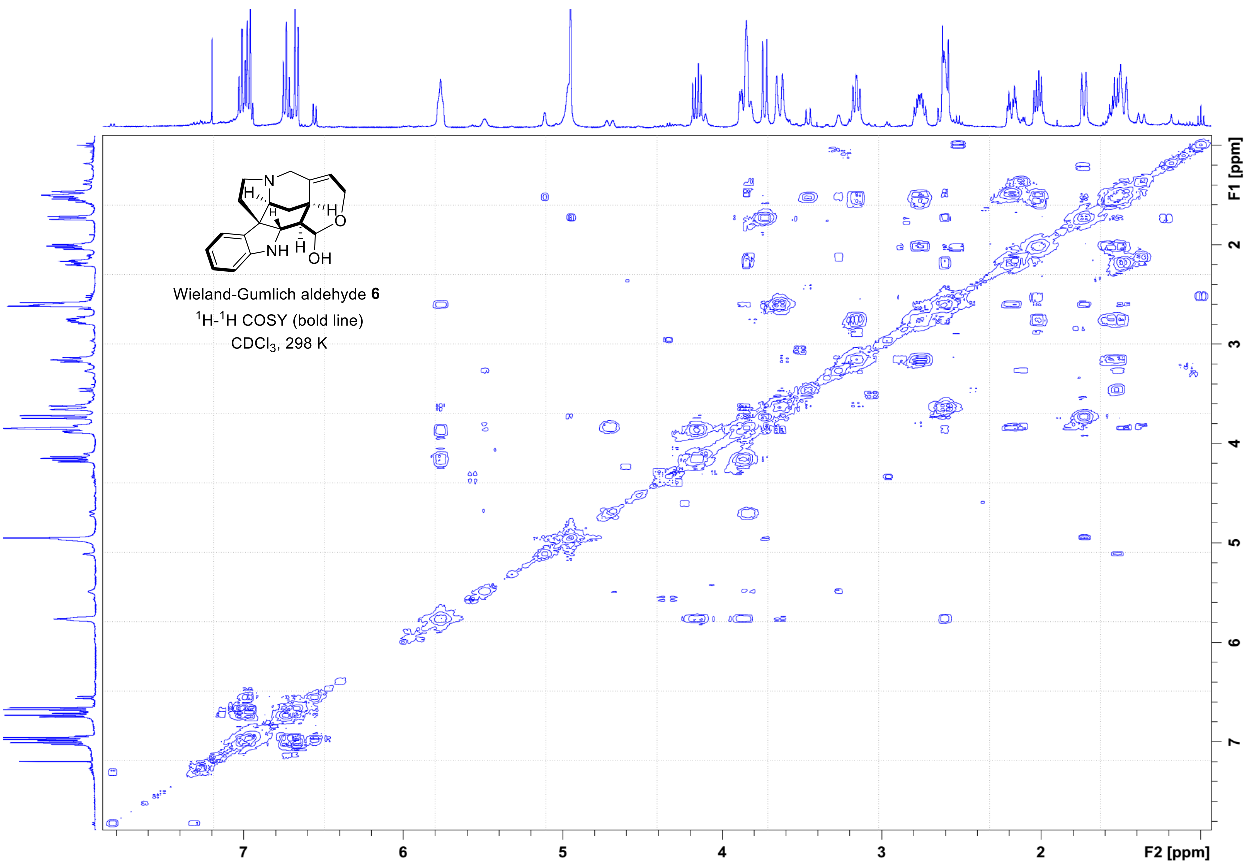
Wieland-Gumlich aldehyde 6
1H-1H COSY (bold line)
CDCl3, 298 K
F1 [ppm]
F2 [ppm]

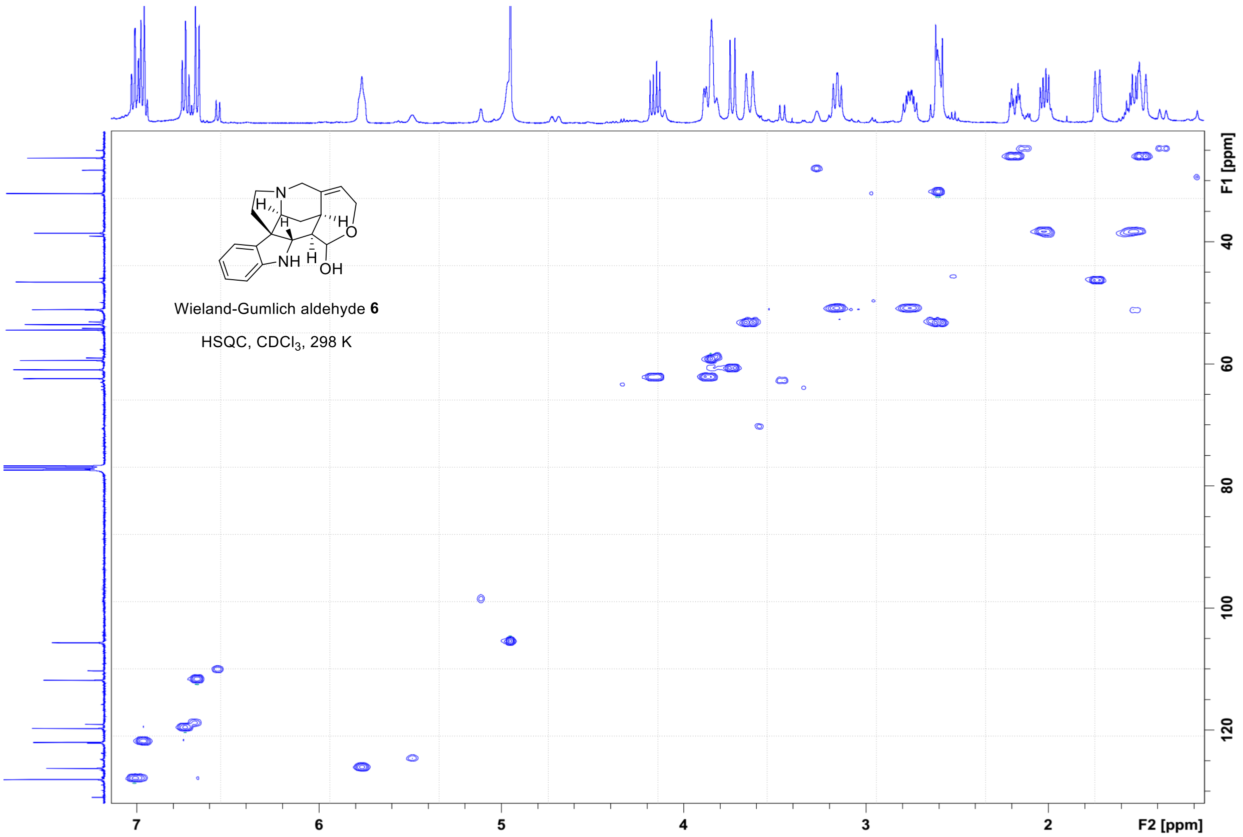
Wieland-Gumlich aldehyde 6
HSQC, CDCl3, 298 K
F1 [ppm]
F2 [ppm]

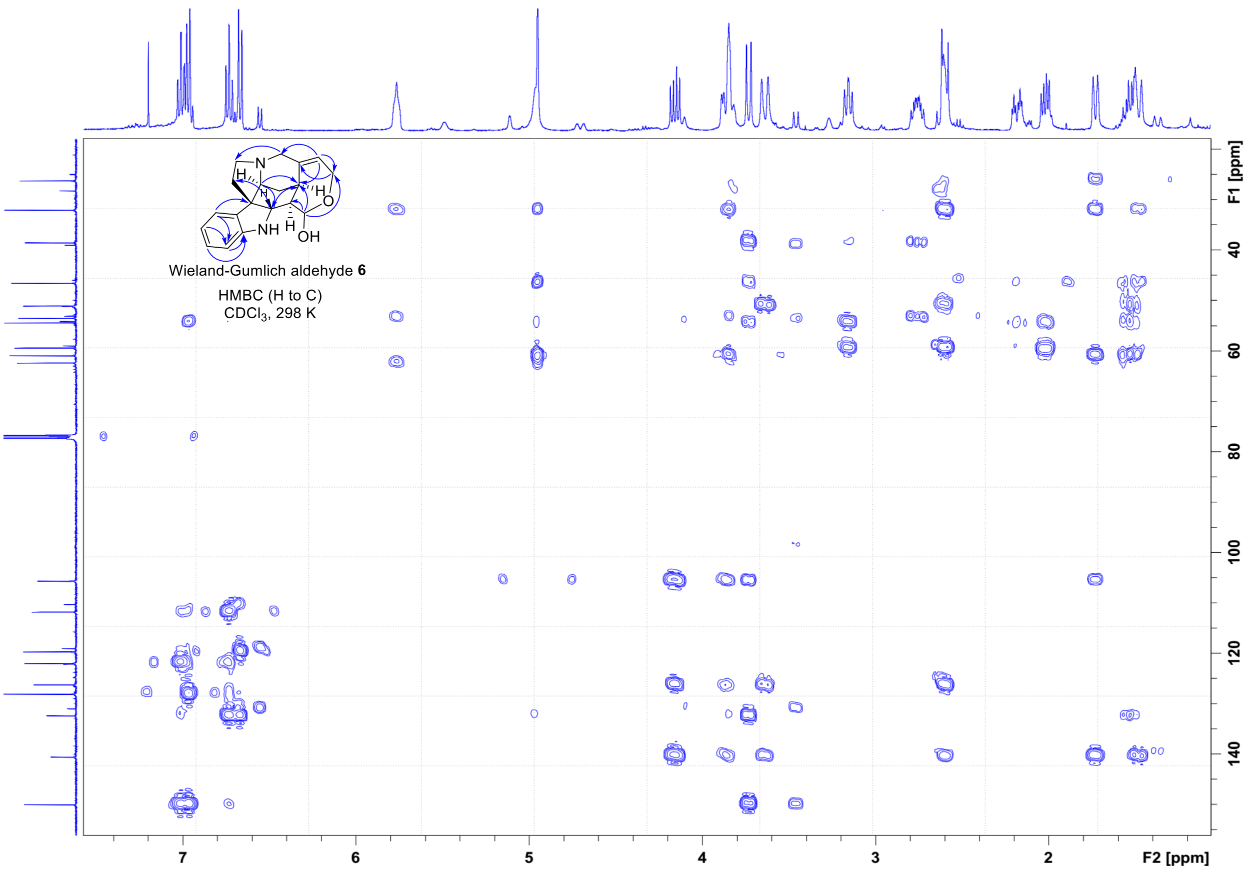
Wieland-Gumlich aldehyde 6
HMBC (H to C)
CDCl3, 298 K
F1 [ppm]
F2 [ppm]

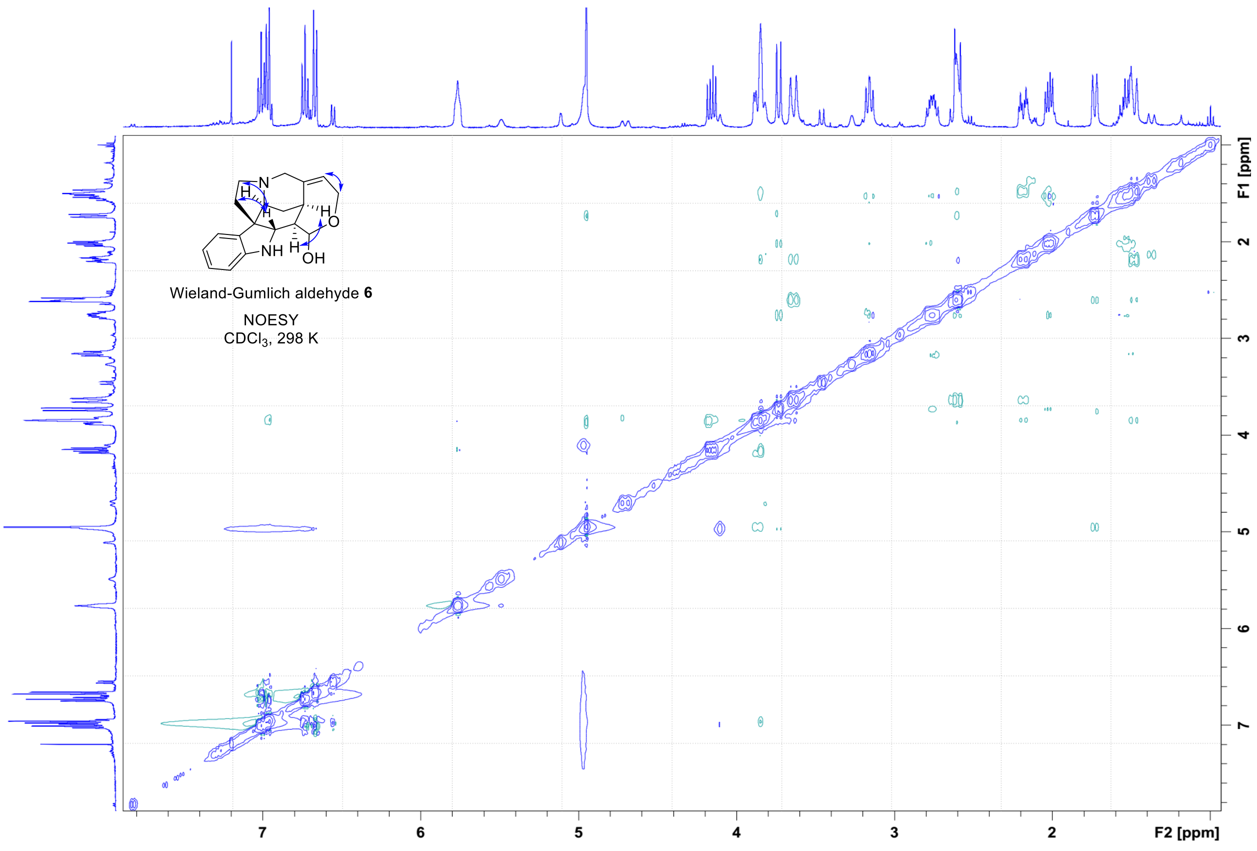
Wieland-Gumlich aldehyde 6
NOESY
CDCl3, 298 K
F1 [ppm]
F2 [ppm]

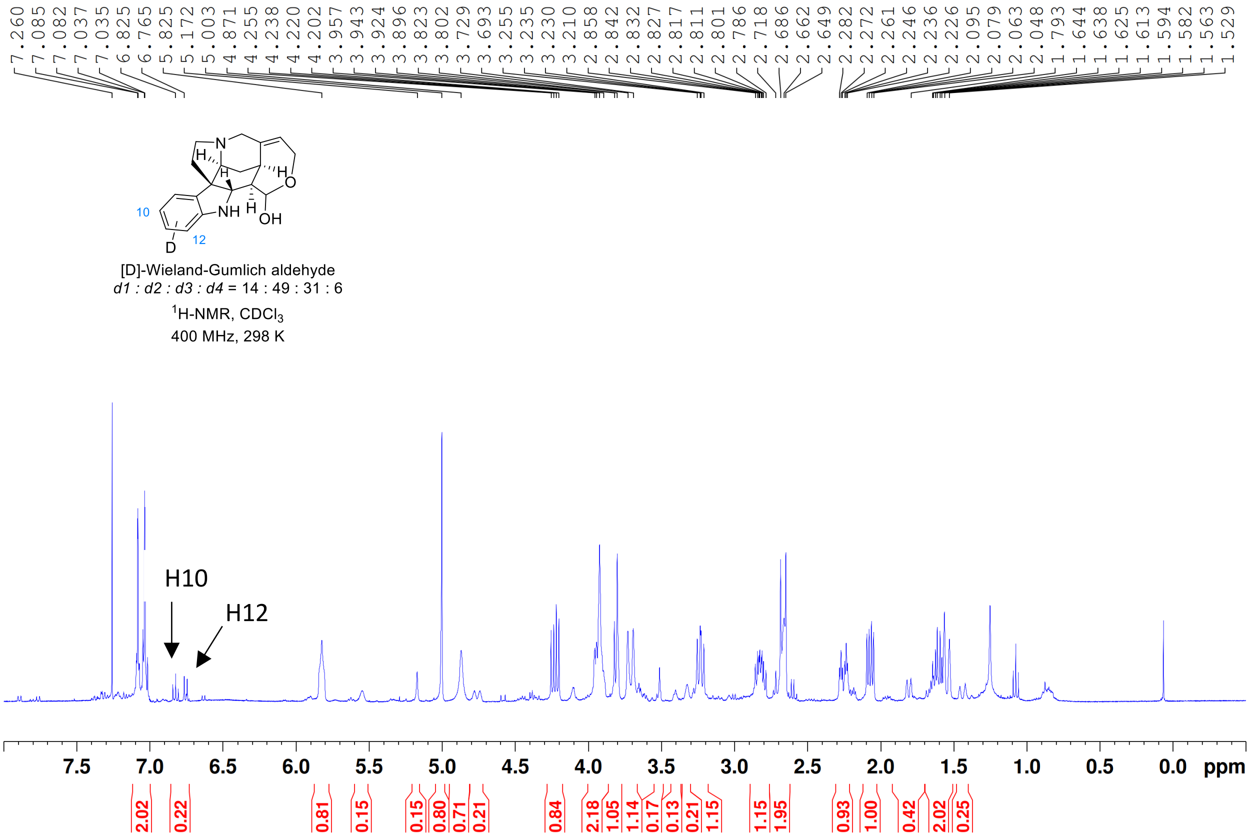
[D]-Wieland-Gumlich aldehyde
d1 : d2 : d3 : d4 = 14 : 49 : 31 : 6
1H-NMR, CDCl3
400 MHz, 298 K
H10
H12
ppm

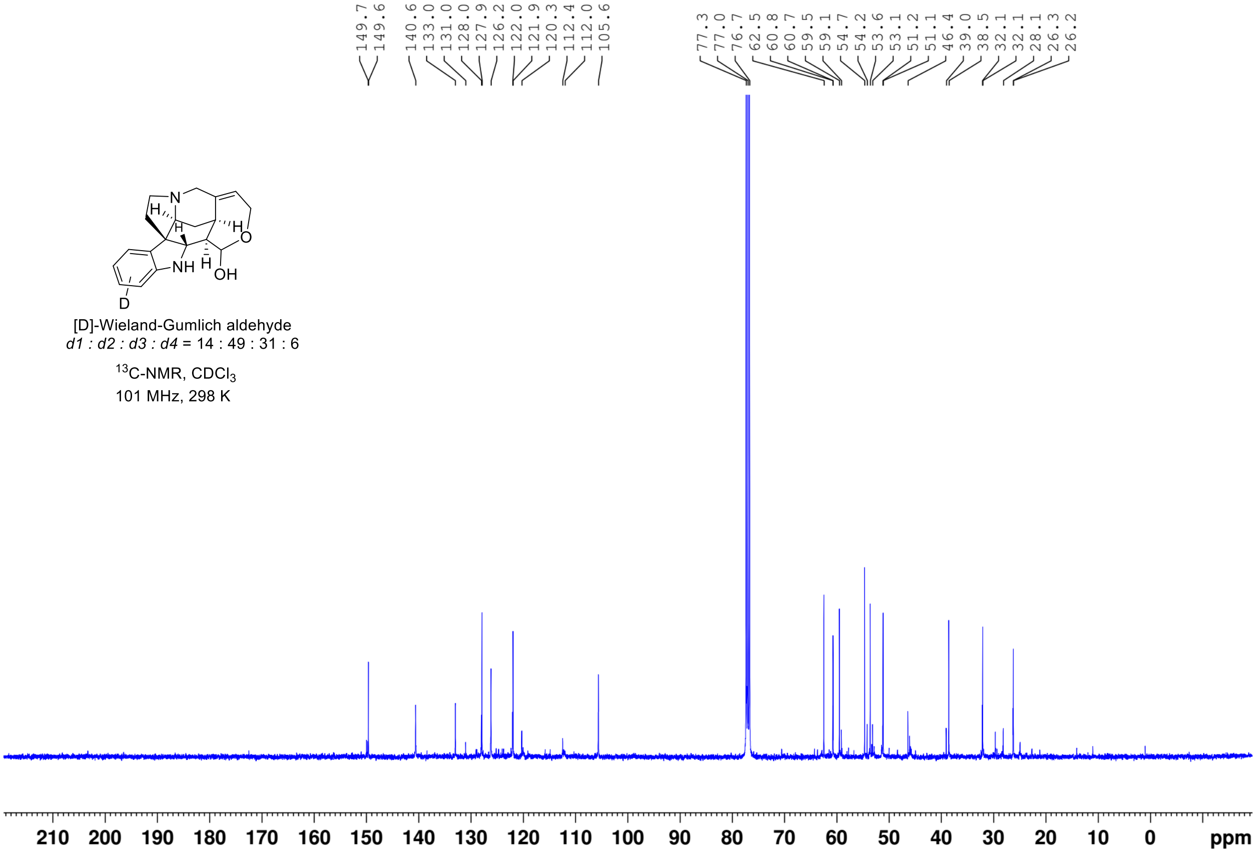
[D]-Wieland-Gumlich aldehyde
d1 : d2 : d3 : d4 = 14 : 49 : 31 : 6
13C-NMR, CDCl3
101 MHz, 298 K
ppm

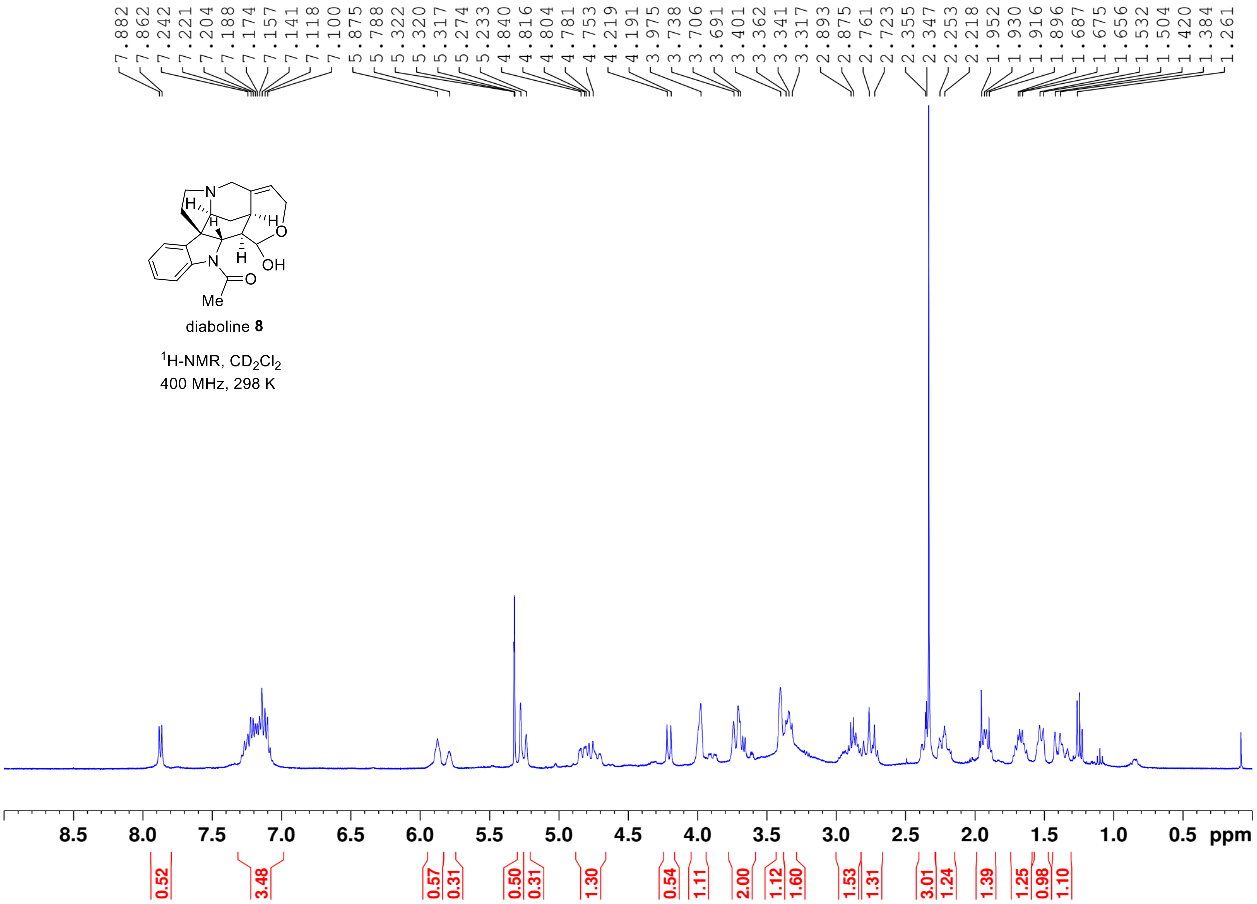
7.882 7.862 7.242 7.221 7.204 7.188 7.174 7.157 7.141 7.118 7.100 5.875 5.788 5.322 5.320 5.317 5.274 5.233 4.840 4.816 4.804 4.781 4.753 4.219 4.191 3.975 3.738 3.706 3.691 3.401 3.362 3.341 3.317 2.893 2.875 2.761 2.723 2.355 2.347 2.253 2.218 1.952 1.930 1.916 1.896 1.687 1.675 1.656 1.532 1.504 1.420 1.384 1.261
N
H
H
H
O
N
H
OH
O
Me
diaboline 8
^{1}H-NMR, CD_2Cl_2
400 MHz, 298 K
8.5 8.0 7.5 7.0 6.5 6.0 5.5 5.0 4.5 4.0 3.5 3.0 2.5 2.0 1.5 1.0 0.5 ppm
0.52 3.48 0.57 0.31 0.50 0.31 1.30 0.54 1.11 2.00 1.12 1.60 1.53 1.31 3.01 1.24 1.39 1.25 0.98 1.10

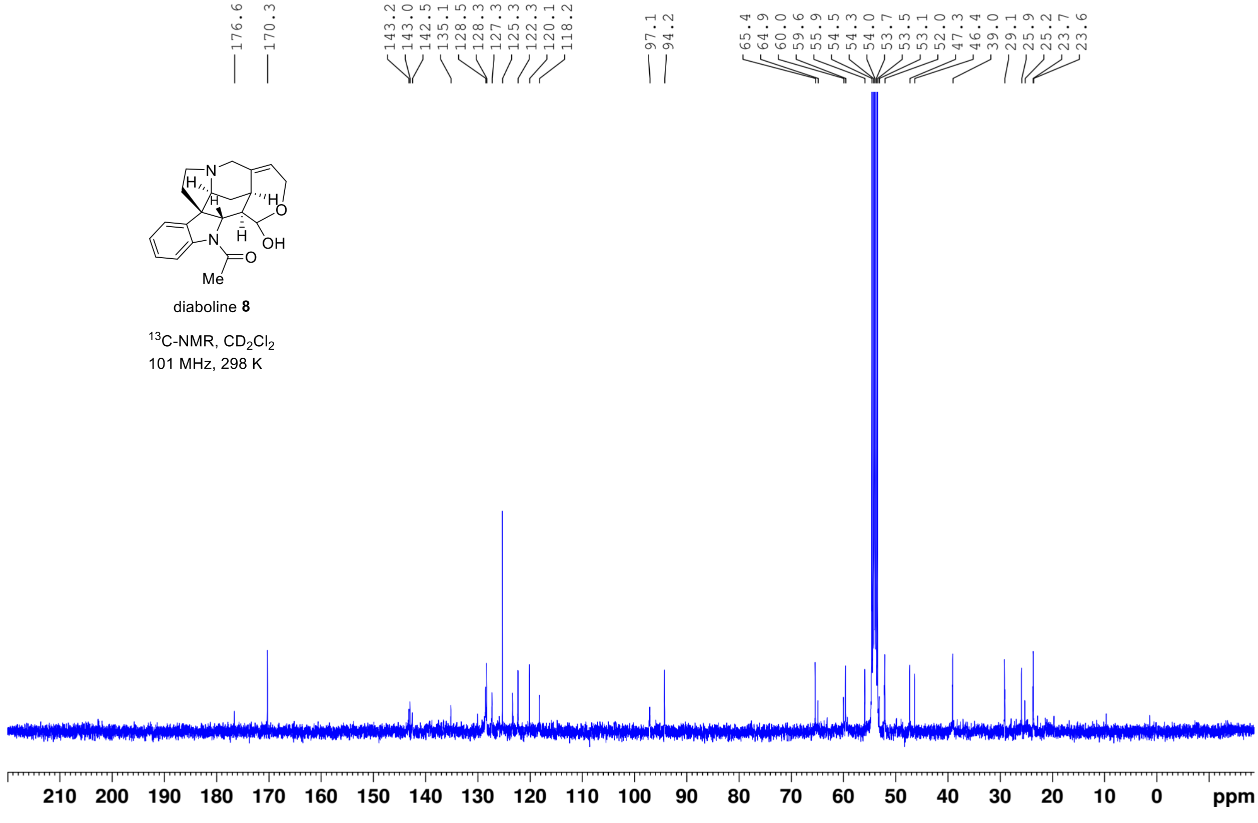
176.6 170.3 143.2 143.0 142.5 135.1 128.5 128.3 127.3 125.3 122.3 120.1 118.2 97.1 94.2 65.4 64.9 60.0 59.6 55.9 55.5 54.5 54.3 54.0 53.7 53.5 53.1 52.0 47.3 46.4 39.0 29.1 25.9 25.2 23.7 23.6
N
H
H
H
O
N
H
OH
O
Me
diaboline 8
^{13}C-NMR, CD_2Cl_2
101 MHz, 298 K
210 200 190 180 170 160 150 140 130 120 110 100 90 80 70 60 50 40 30 20 10 0 ppm

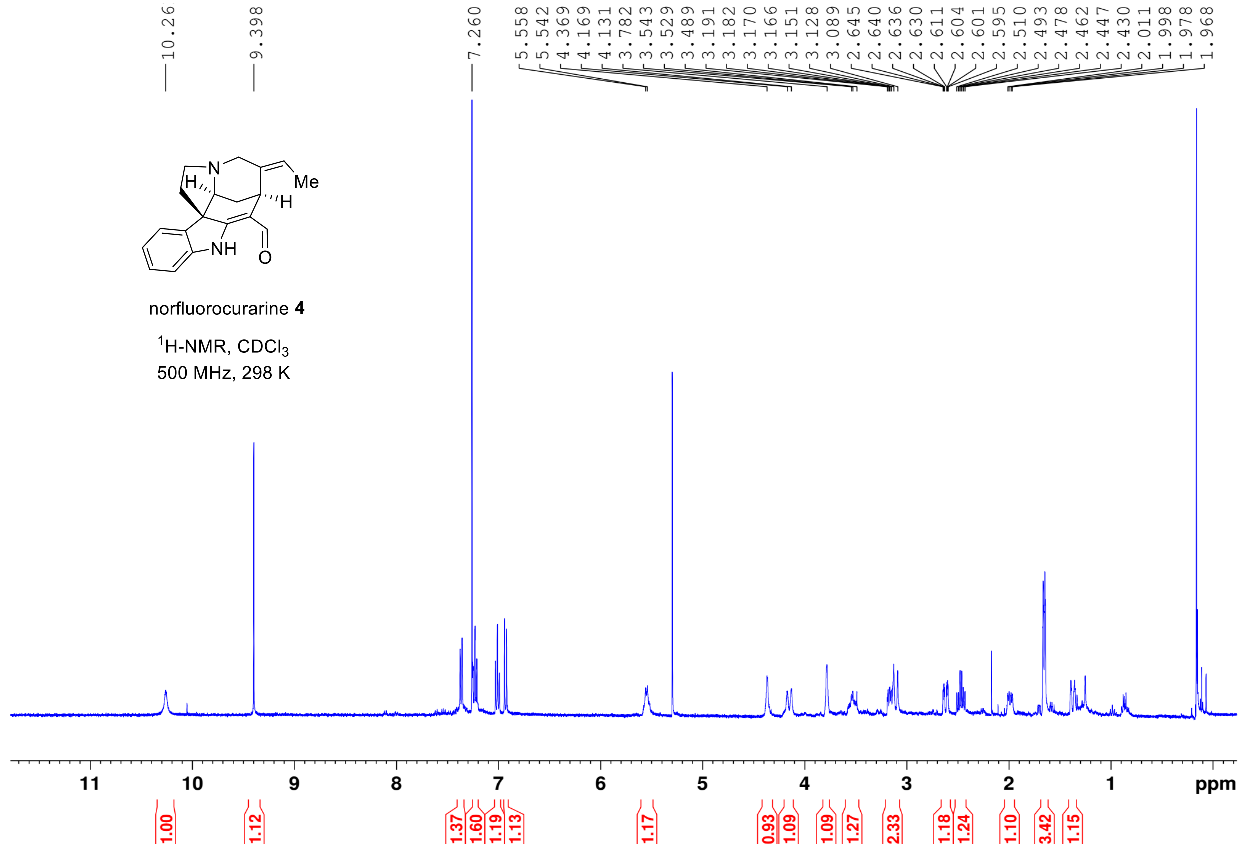
norfluorocurarine 4
^{1}H-NMR, CDCl$_3$
500 MHz, 298 K
10.26
9.398
7.260
5.558 5.542 4.369 4.169 4.131 3.782 3.543 3.529 3.489 3.191 3.182 3.170 3.166 3.151 3.128 3.089 2.645 2.640 2.636 2.630 2.611 2.604 2.601 2.595 2.510 2.493 2.478 2.462 2.447 2.430 2.011 1.998 1.978 1.968
1.00 1.12 1.37 1.60 1.19 1.13 1.17 0.93 1.09 1.09 1.27 2.33 1.18 1.24 1.10 3.42 1.15
11 10 9 8 7 6 5 4 3 2 1 ppm

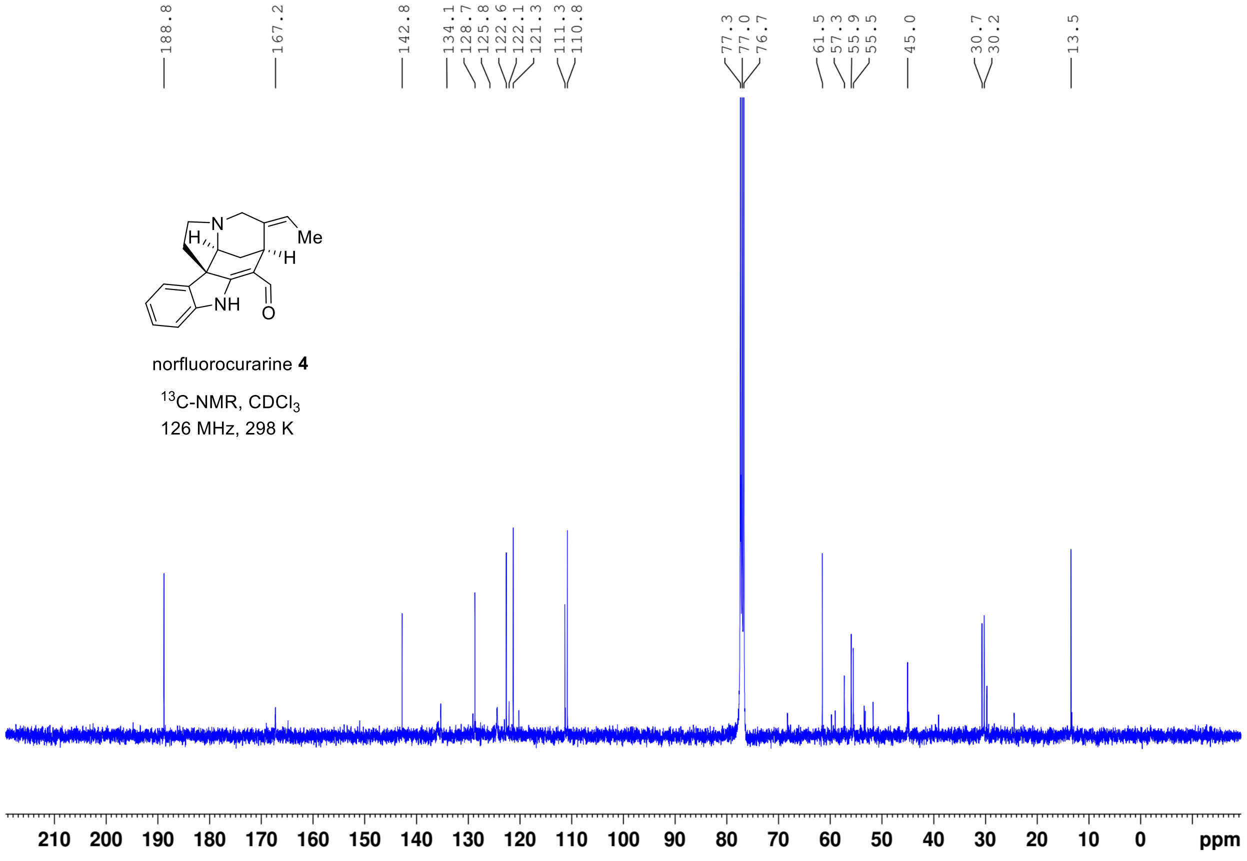
norfluorocurarine 4
^{13}C-NMR, CDCl$_3$
126 MHz, 298 K
188.8
167.2
142.8
134.1 128.7 125.8 122.6 122.1 121.3 111.3 110.8
77.3 77.0 76.7
61.5 57.3 55.9 55.5
45.0
30.7 30.2
13.5
210 200 190 180 170 160 150 140 130 120 110 100 90 80 70 60 50 40 30 20 10 0 ppm

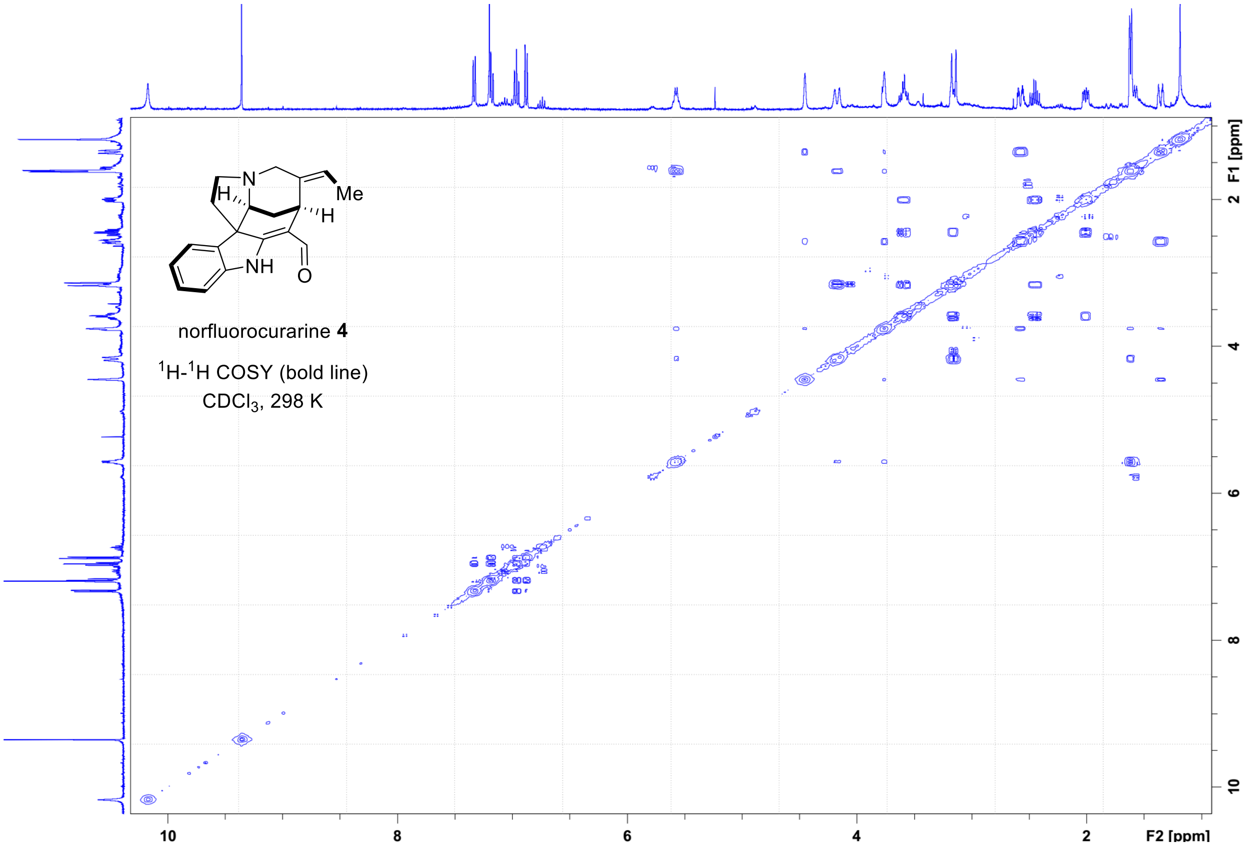
N
Me
H
H
NH
O
norfluorocurarine 4
1H-1H COSY (bold line)
CDCl3, 298 K
F1 [ppm]
F2 [ppm]

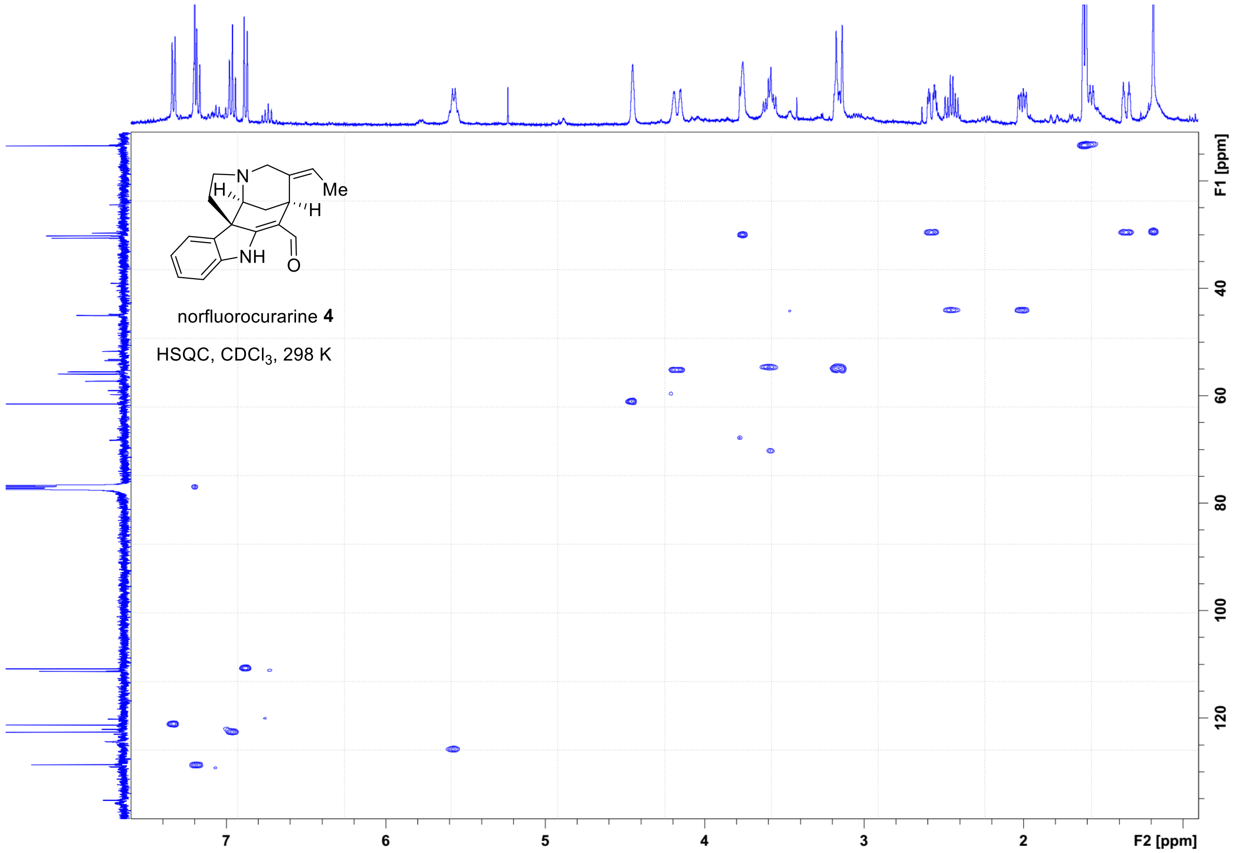
N
Me
H
H
NH
O
norfluorocurarine 4
HSQC, CDCl3, 298 K
F1 [ppm]
F2 [ppm]

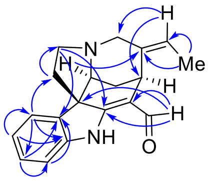

norfluorocurarine **4**

HMBC (H to C)
$CDCl_3$, 298 K

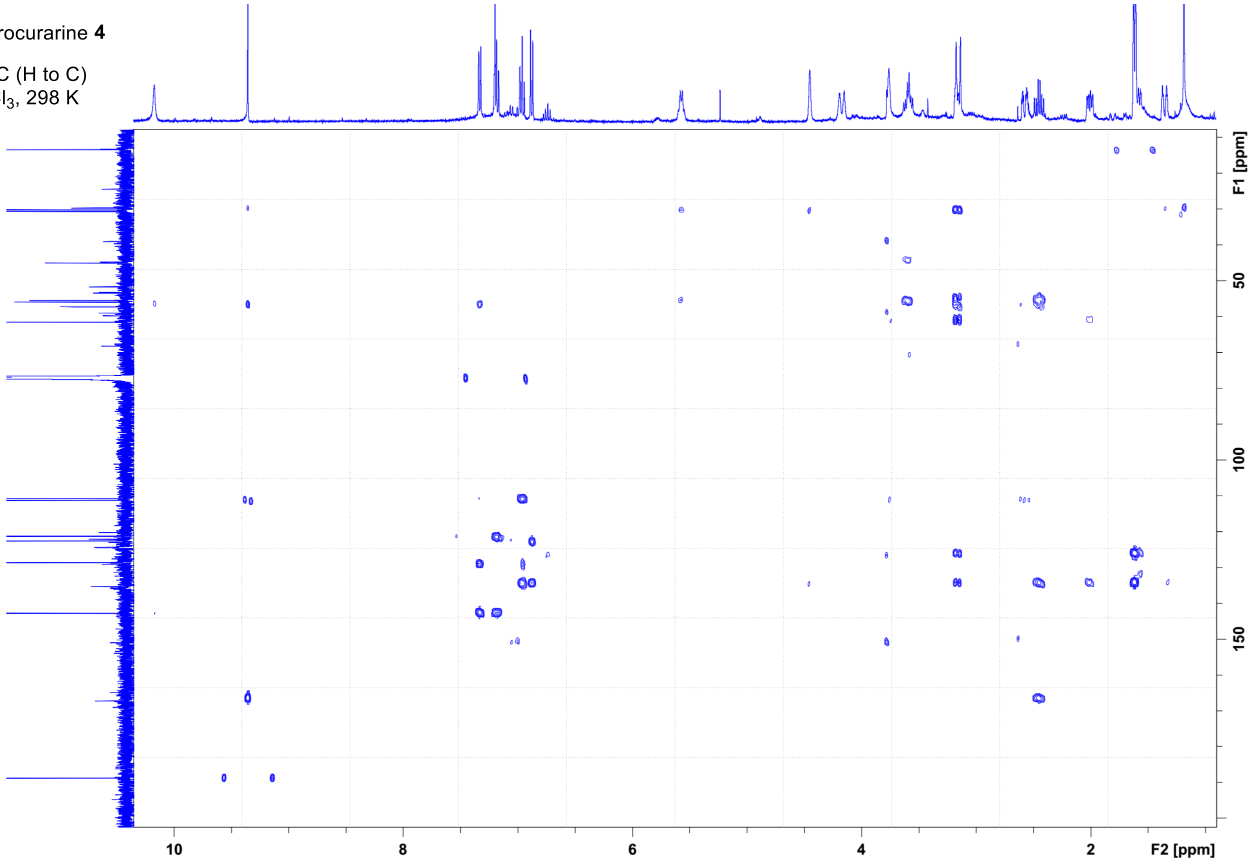

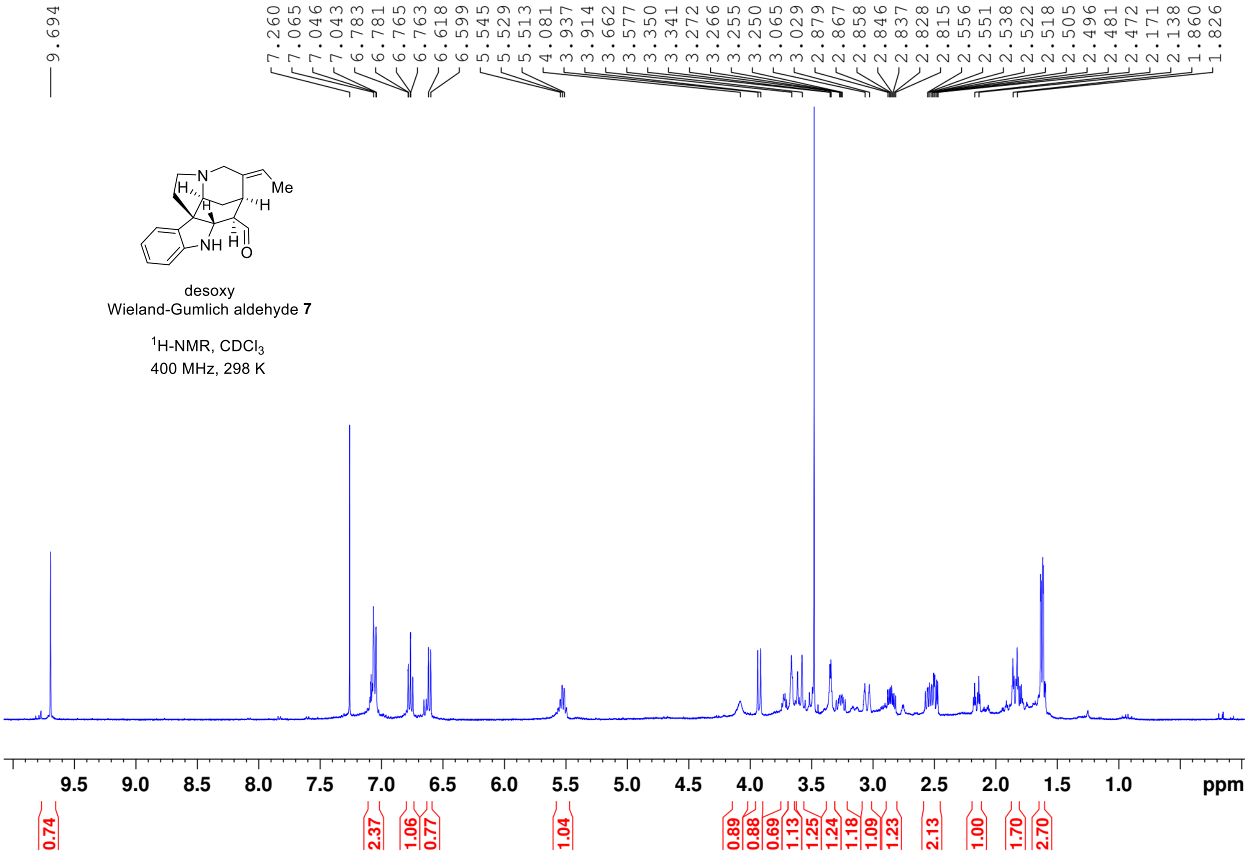
9.694
7.260 7.065 7.046 7.043 6.783 6.781 6.765 6.763 6.618 6.599 5.545 5.529 5.513 4.081 3.937 3.914 3.662 3.577 3.350 3.341 3.272 3.266 3.255 3.250 3.065 3.029 2.879 2.867 2.858 2.846 2.837 2.828 2.815 2.556 2.551 2.538 2.522 2.518 2.505 2.496 2.481 2.472 2.171 2.138 1.860 1.826
Me
NH
O
desoxy
Wieland-Gumlich aldehyde 7
^{1}H-NMR, $CDCl_3$
400 MHz, 298 K
9.5 9.0 8.5 8.0 7.5 7.0 6.5 6.0 5.5 5.0 4.5 4.0 3.5 3.0 2.5 2.0 1.5 1.0 ppm
0.74 2.37 1.06 0.77 1.04 0.89 0.88 0.69 1.13 1.25 1.24 1.18 1.09 1.23 2.13 1.00 1.70 2.70

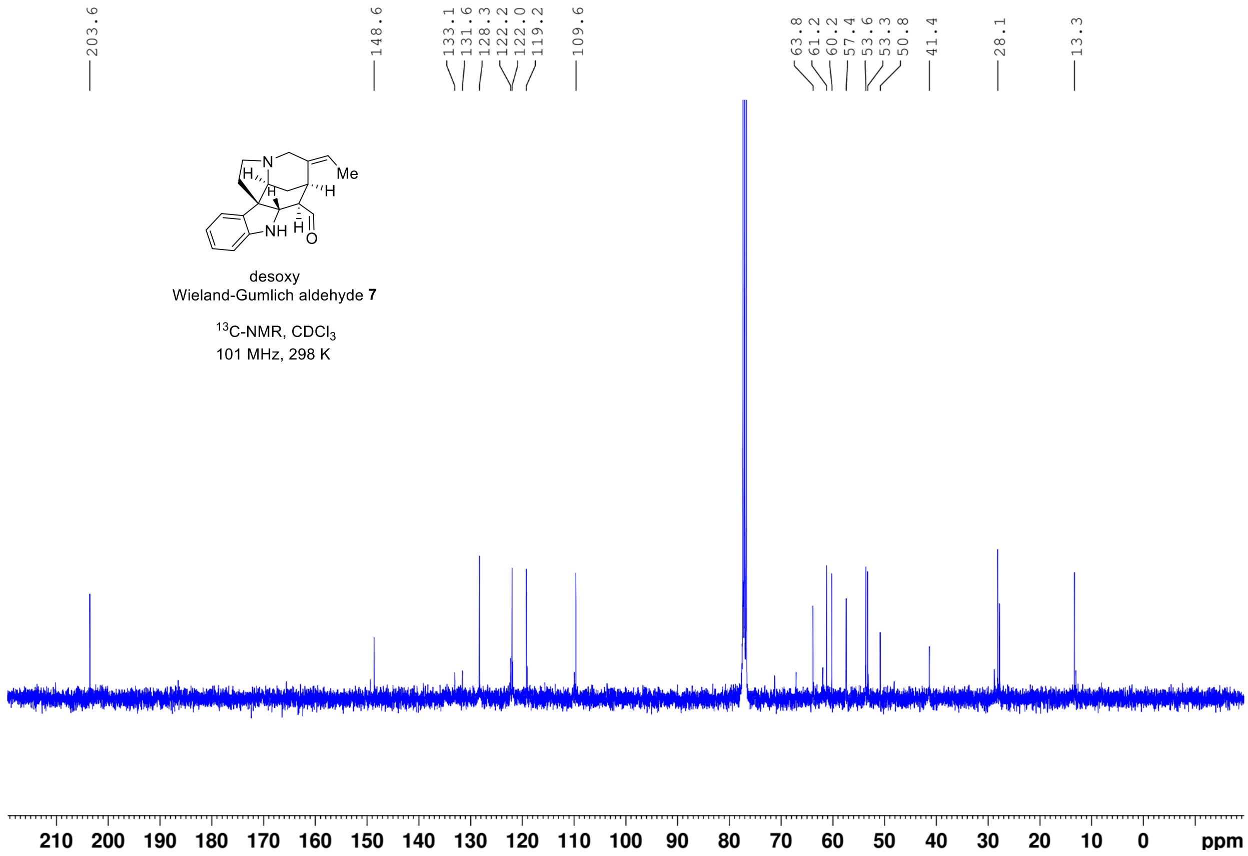
203.6
148.6
133.1 131.6 128.3 122.2 122.0 119.2
109.6
63.8 61.2 60.2 57.4 53.6 53.3 50.8
41.4
28.1
13.3
Me
NH
O
desoxy
Wieland-Gumlich aldehyde 7
^{13}C-NMR, $CDCl_3$
101 MHz, 298 K
210 200 190 180 170 160 150 140 130 120 110 100 90 80 70 60 50 40 30 20 10 0 ppm

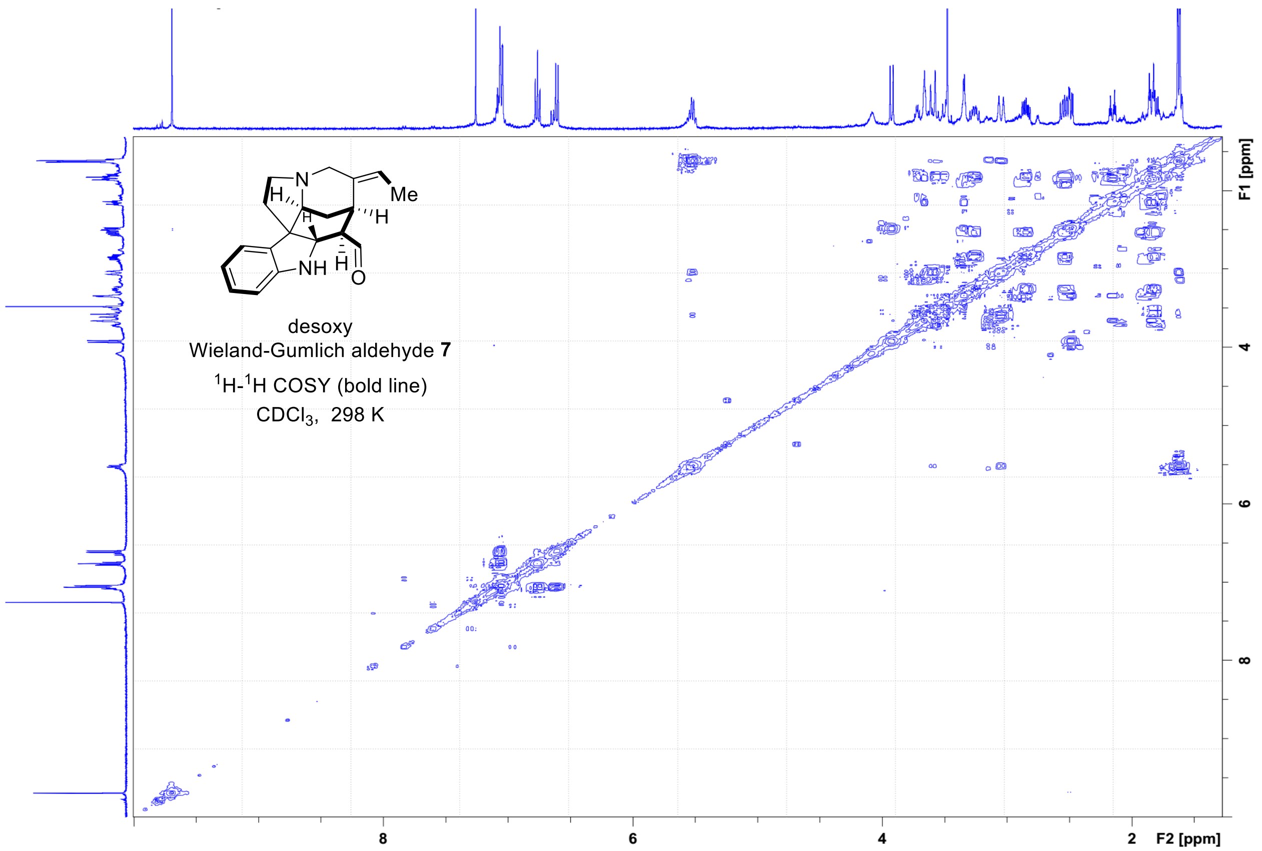

Me
H
H
N
NH
H
O
desoxy
Wieland-Gumlich aldehyde 7
^{1}H-^{1}H COSY (bold line)
$CDCl_3$, 298 K
F1 [ppm]
F2 [ppm]

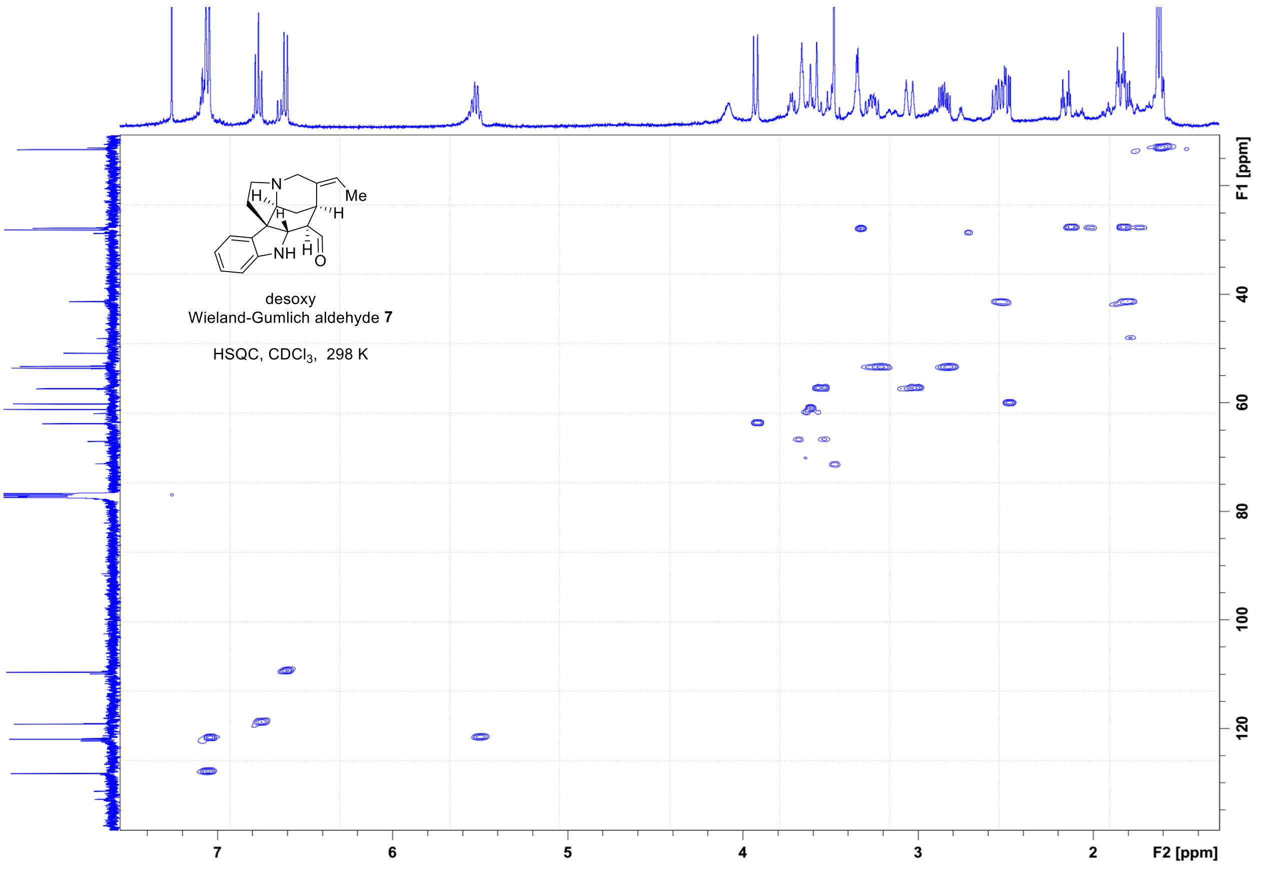

Me
H
H
N
NH
H
O
desoxy
Wieland-Gumlich aldehyde 7
HSQC, $CDCl_3$, 298 K
F1 [ppm]
F2 [ppm]

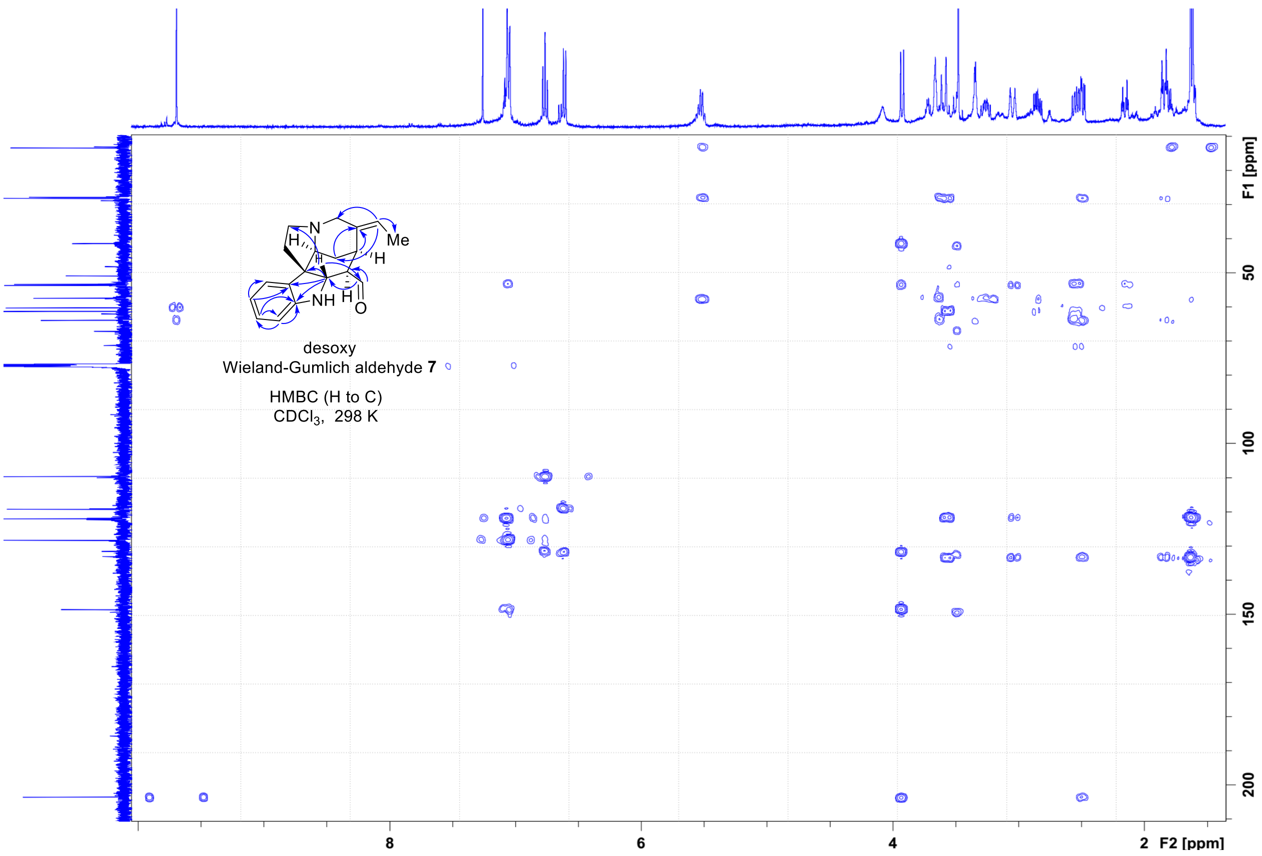
N
Me
H
NH
O
desoxy
Wieland-Gumlich aldehyde 7
HMBC (H to C)
CDCl3, 298 K
F1 [ppm]
50
100
150
200
8
6
4
2
F2 [ppm]

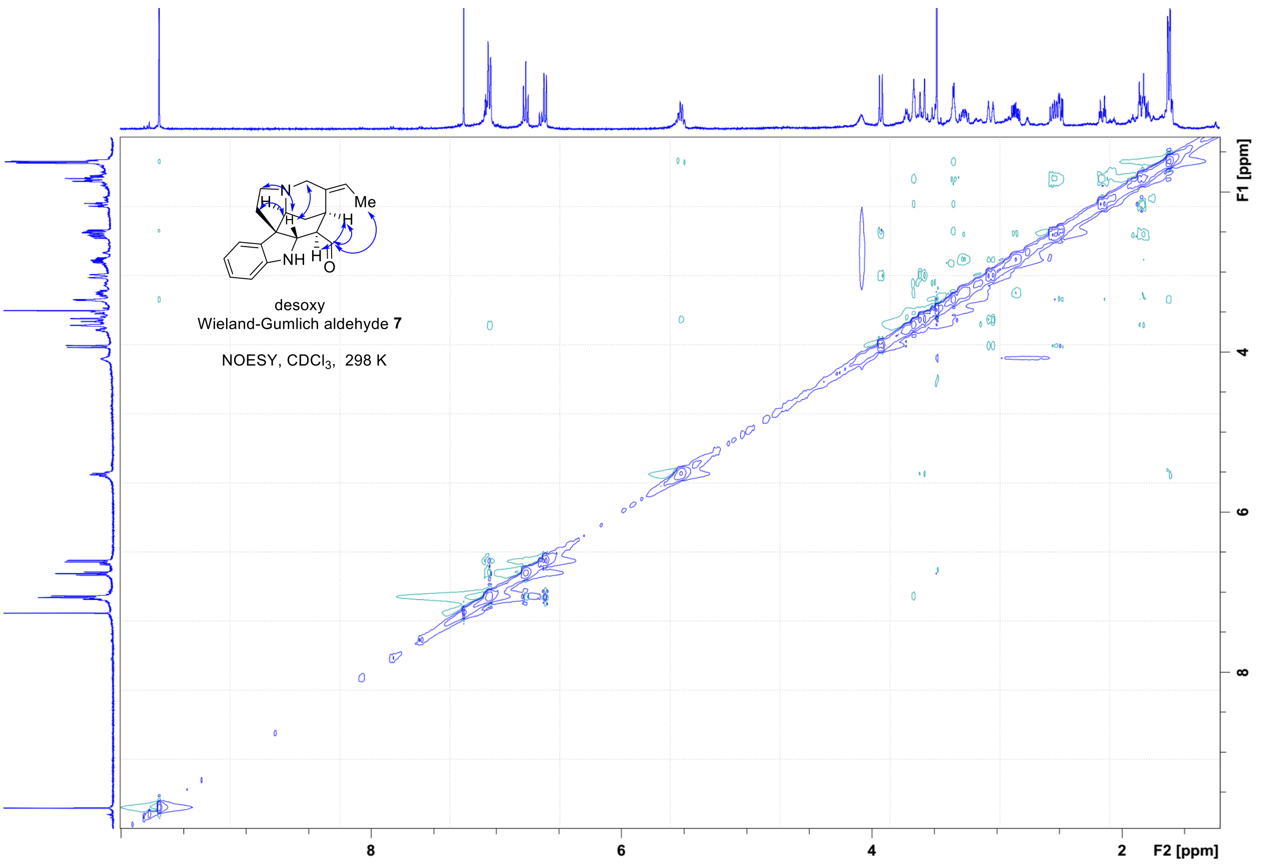
N
Me
H
NH
O
desoxy
Wieland-Gumlich aldehyde 7
NOESY, CDCl3, 298 K
F1 [ppm]
2
4
6
8
F2 [ppm]

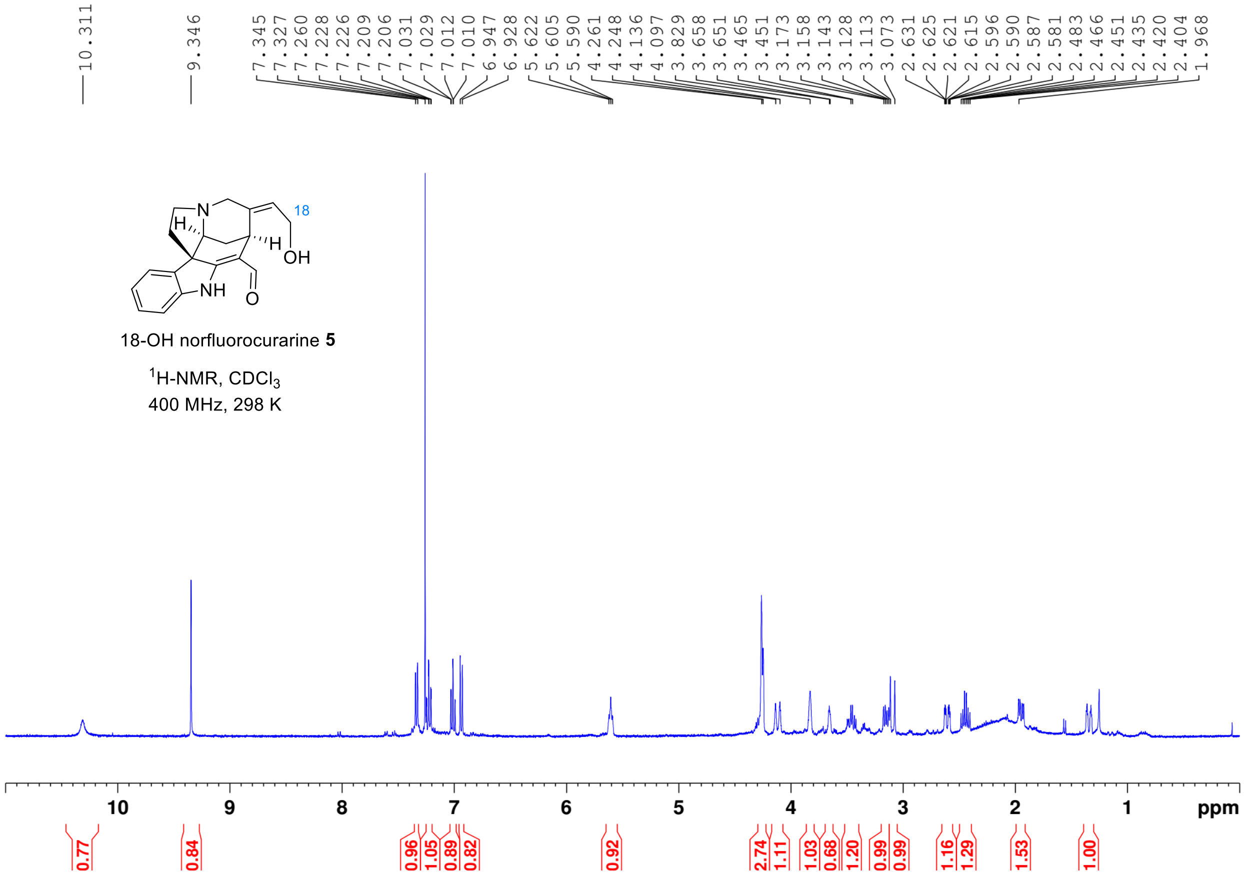
10.311
9.346
7.345 7.327 7.260 7.228 7.226 7.209 7.206 7.031 7.029 7.012 7.010 6.947 6.928
5.622 5.605 5.590
4.261 4.248 4.136 4.097 3.829 3.658 3.651 3.465 3.451 3.173 3.158 3.143 3.128 3.113 3.073
2.631 2.625 2.621 2.615 2.596 2.590 2.587 2.581 2.483 2.466 2.451 2.435 2.420 2.404 1.968
18
H
N
H
OH
NH
O
18-OH norfluorocurarine 5
^{1}H-NMR, $CDCl_3$
400 MHz, 298 K
10 9 8 7 6 5 4 3 2 1 ppm
0.77 0.84 0.96 1.05 0.89 0.82 0.92 2.74 1.11 1.03 0.68 1.20 0.99 0.99 1.16 1.29 1.53 1.00

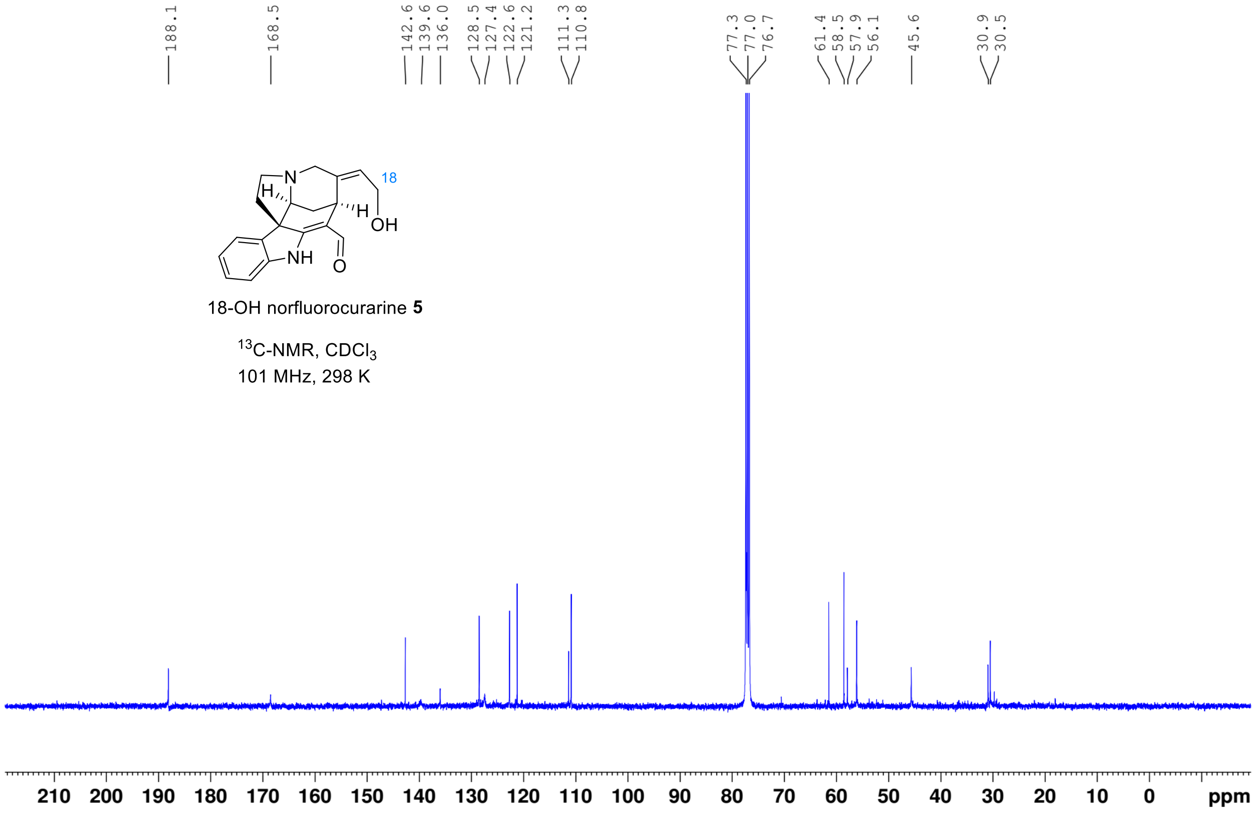
188.1
168.5
142.6 139.6 136.0 128.5 127.4 122.6 121.2 111.3 110.8
77.3 77.0 76.7
61.4 58.5 57.9 56.1 45.6 30.9 30.5
18
H
N
H
OH
NH
O
18-OH norfluorocurarine 5
^{13}C-NMR, $CDCl_3$
101 MHz, 298 K
210 200 190 180 170 160 150 140 130 120 110 100 90 80 70 60 50 40 30 20 10 0 ppm

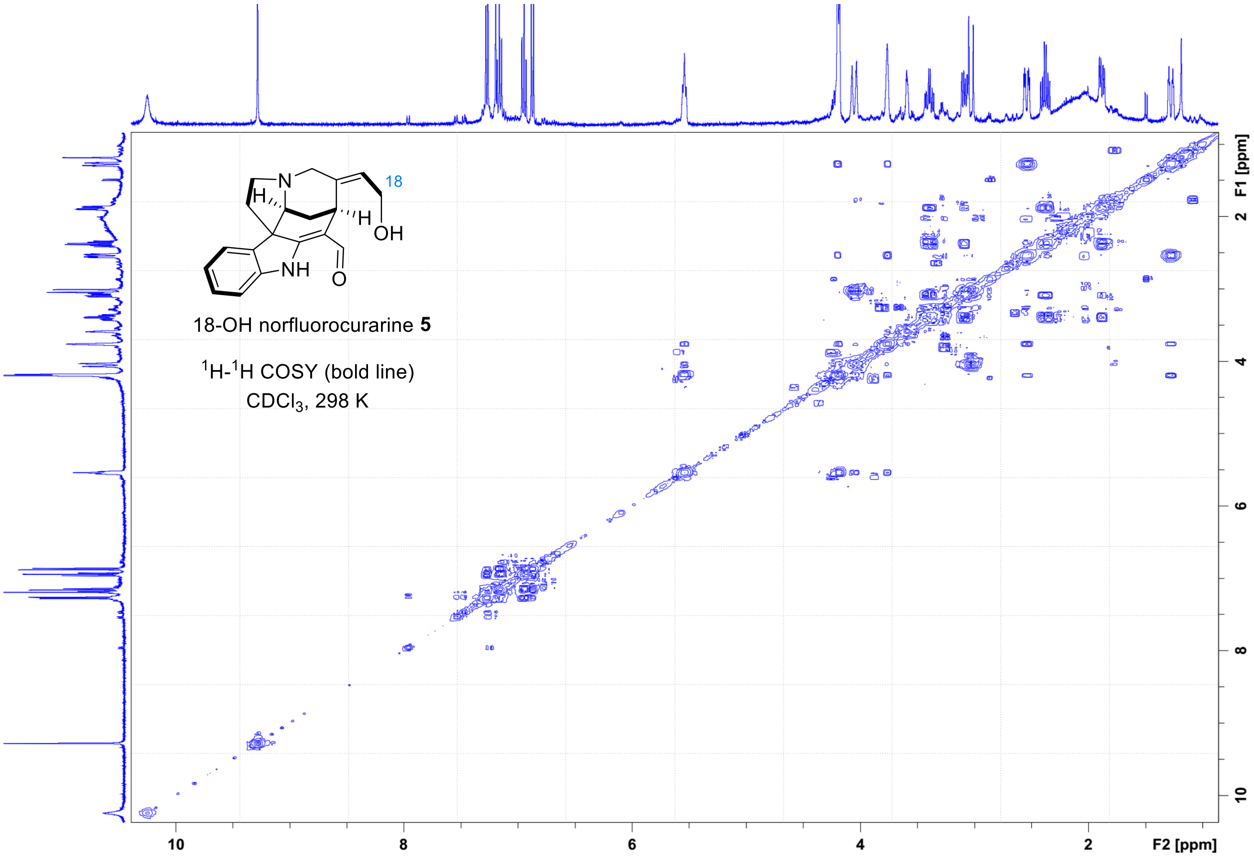
18
OH
NH
O
N
H
H
18-OH norfluorocurarine **5**
^{1}H-^{1}H COSY (bold line)
$CDCl_3$, 298 K
F1 [ppm]
F2 [ppm]
2
4
6
8
10

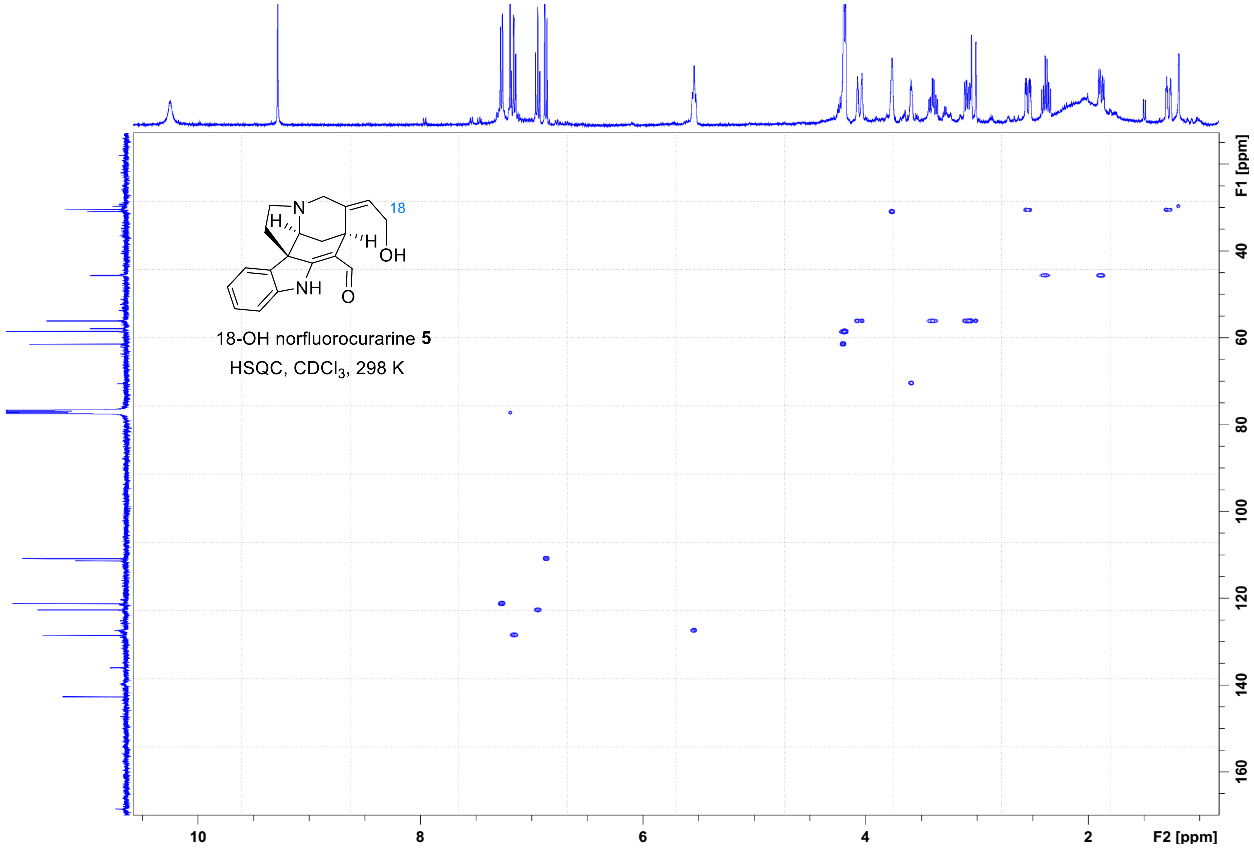
18
OH
NH
O
N
H
H
18-OH norfluorocurarine **5**
HSQC, $CDCl_3$, 298 K
F1 [ppm]
F2 [ppm]
40
60
80
100
120
140
160
10
8
6
4
2

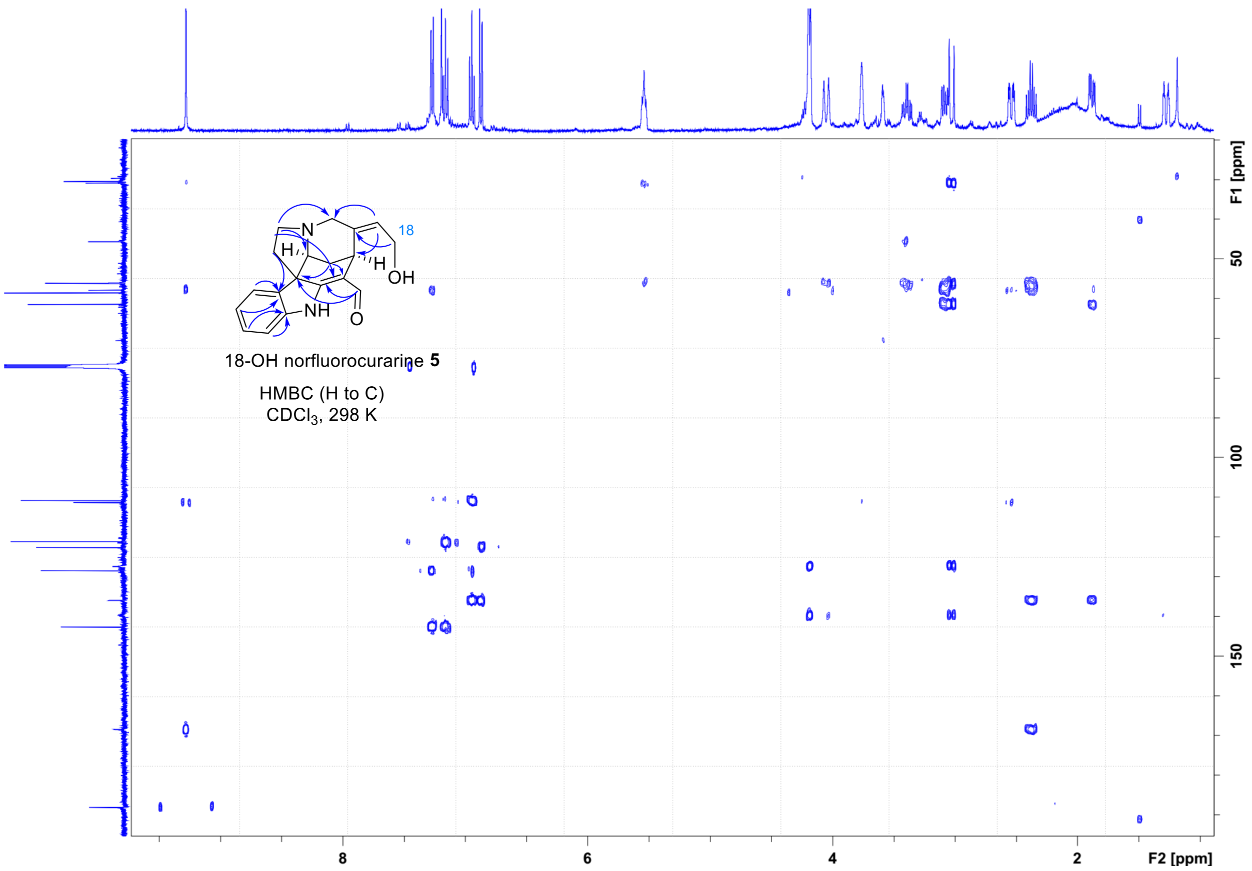
N
18
H
H
OH
NH
O
18-OH norfluorocurarine 5
HMBC (H to C)
CDCl3, 298 K
F1 [ppm]
50
100
150
8
6
4
2
F2 [ppm]

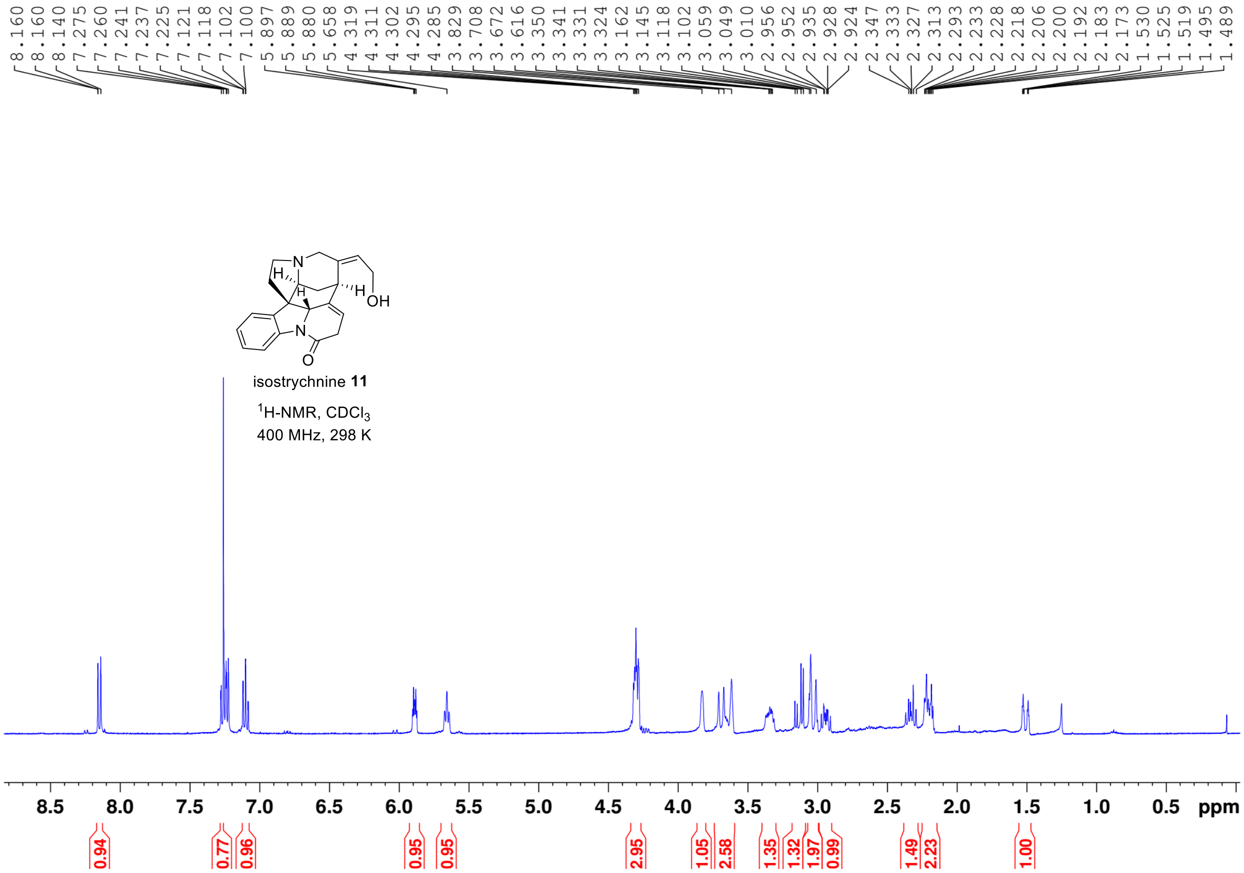
8.160 8.160 8.140 7.275 7.260 7.241 7.237 7.225 7.121 7.118 7.102 7.100 5.897 5.889 5.880 5.658 4.319 4.311 4.302 4.295 4.285 3.829 3.708 3.672 3.616 3.350 3.341 3.331 3.324 3.162 3.145 3.118 3.102 3.059 3.049 3.010 2.956 2.952 2.935 2.928 2.924 2.347 2.333 2.327 2.313 2.293 2.233 2.228 2.218 2.206 2.200 2.192 2.183 2.173 1.530 1.525 1.519 1.495 1.489
H
H
N
H
OH
N
O
isostrychnine 11
1H-NMR, CDCl3
400 MHz, 298 K
8.5 8.0 7.5 7.0 6.5 6.0 5.5 5.0 4.5 4.0 3.5 3.0 2.5 2.0 1.5 1.0 0.5 ppm
0.94 0.77 0.96 0.95 0.95 2.95 1.05 2.58 1.35 1.32 1.97 0.99 1.49 2.23 1.00

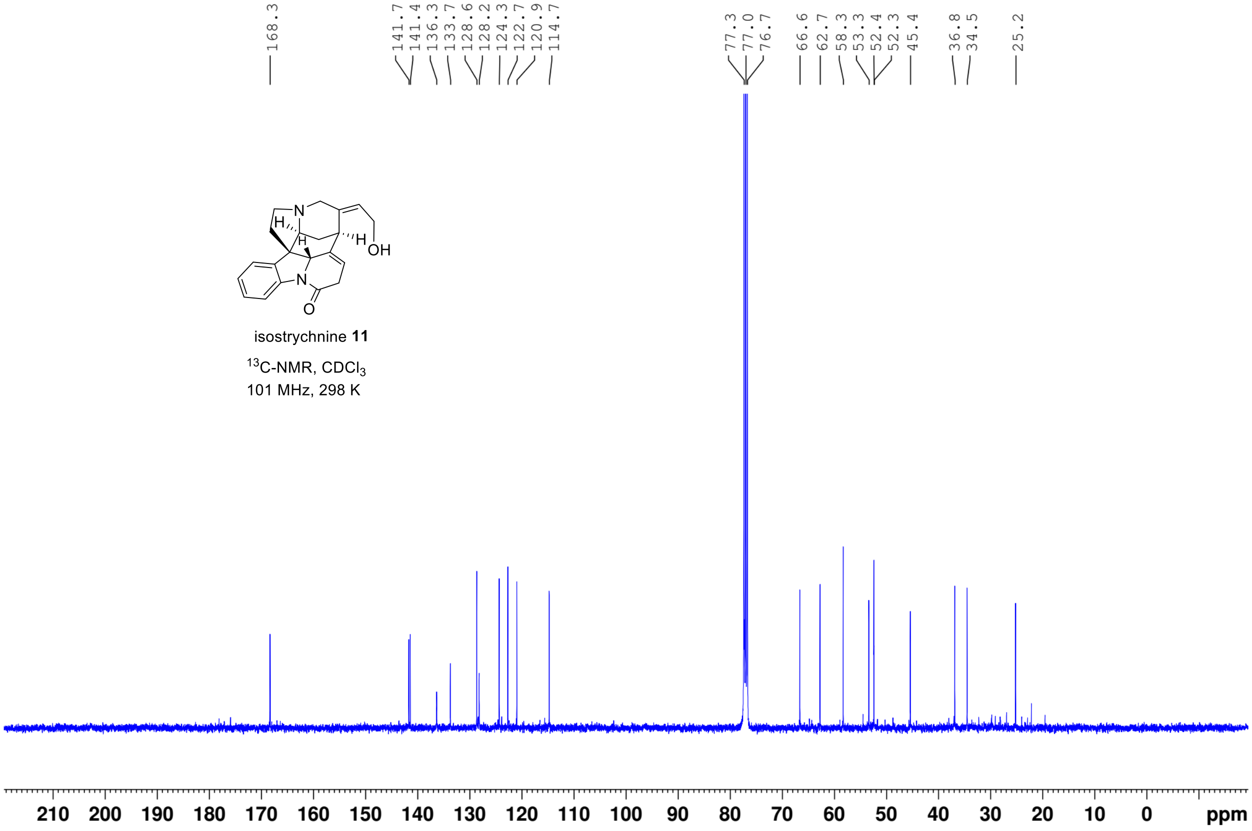
168.3 141.7 141.4 136.3 133.7 128.6 128.2 124.3 122.7 120.9 114.7 77.3 77.0 76.7 66.6 62.7 58.3 53.3 52.4 52.3 45.4 36.8 34.5 25.2
H
H
N
H
OH
N
O
isostrychnine 11
13C-NMR, CDCl3
101 MHz, 298 K
210 200 190 180 170 160 150 140 130 120 110 100 90 80 70 60 50 40 30 20 10 0 ppm

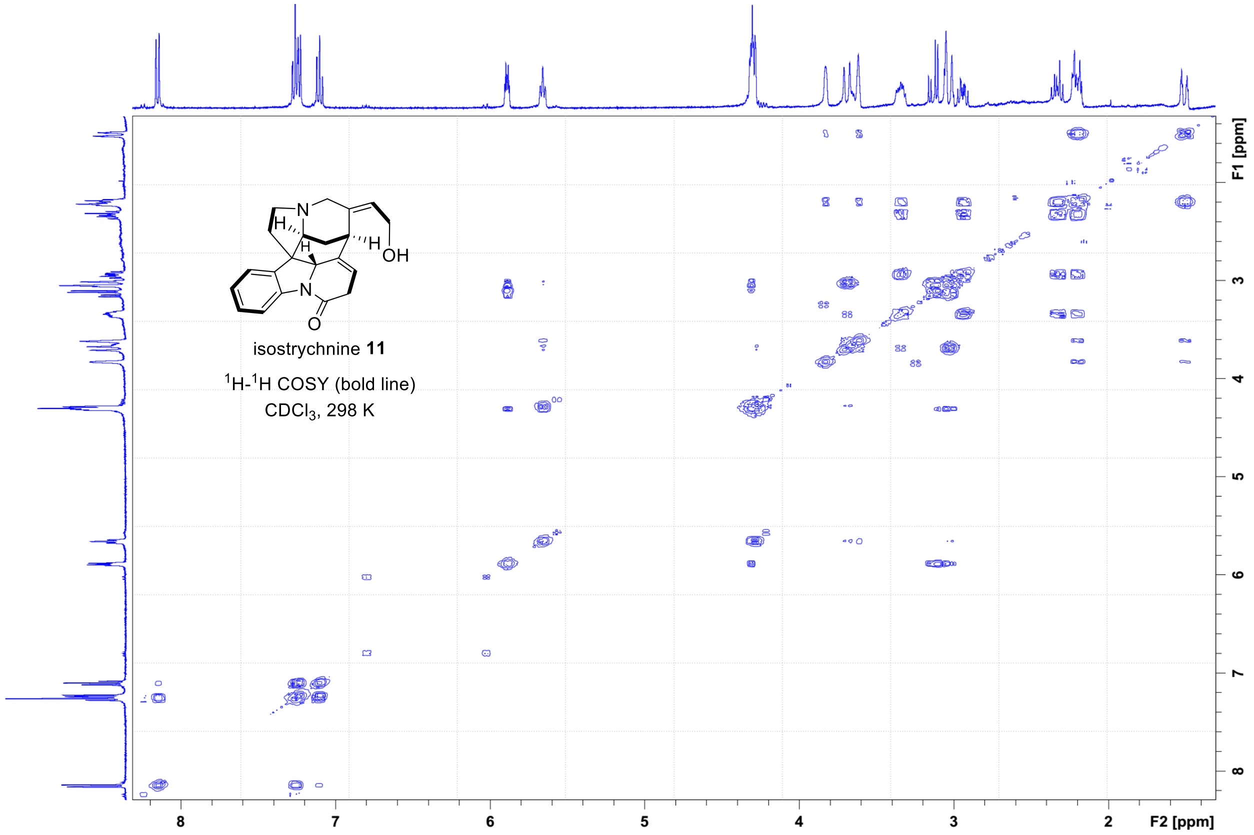
isostrychnine **11**
^{1}H-^{1}H COSY (bold line)
$CDCl_3$, 298 K
F1 [ppm]
F2 [ppm]

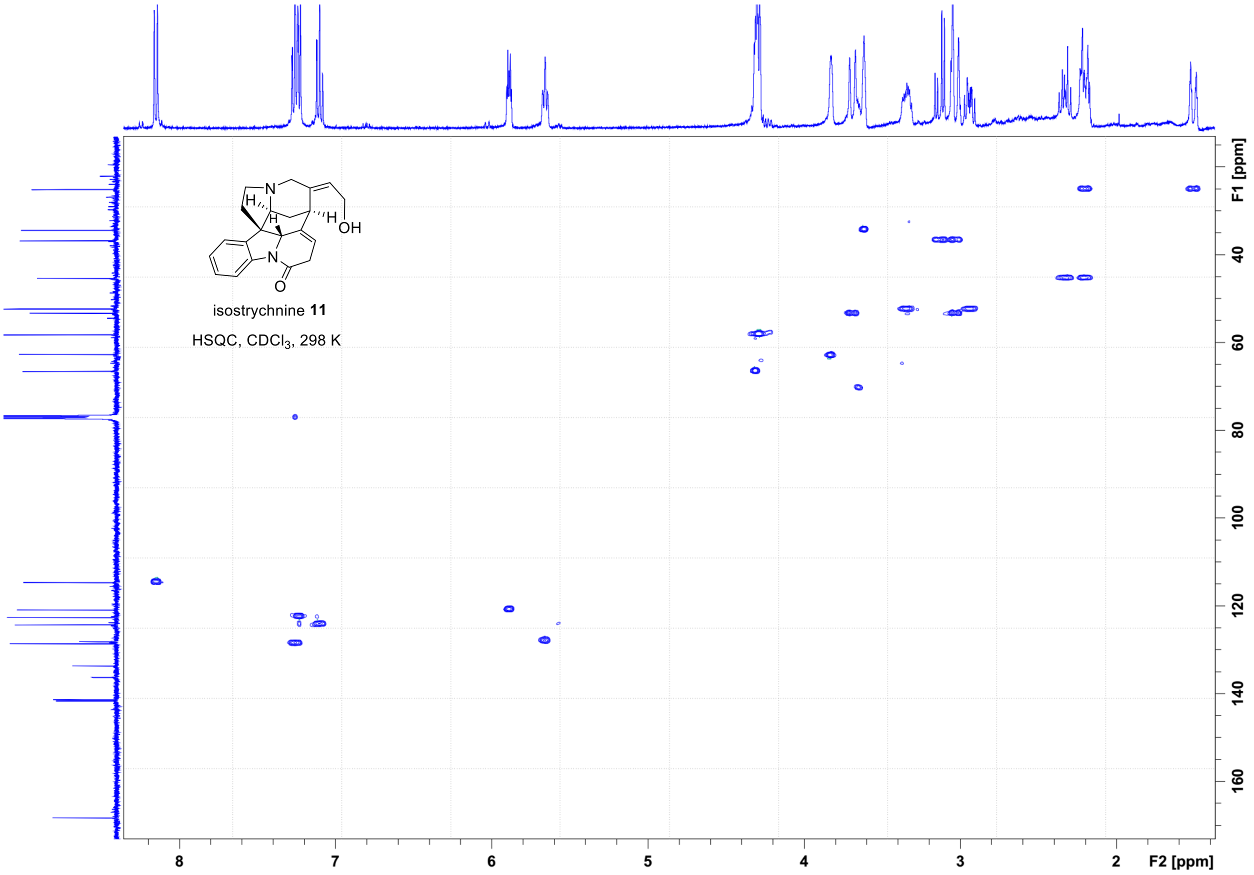
isostrychnine **11**
HSQC, $CDCl_3$, 298 K
F1 [ppm]
F2 [ppm]

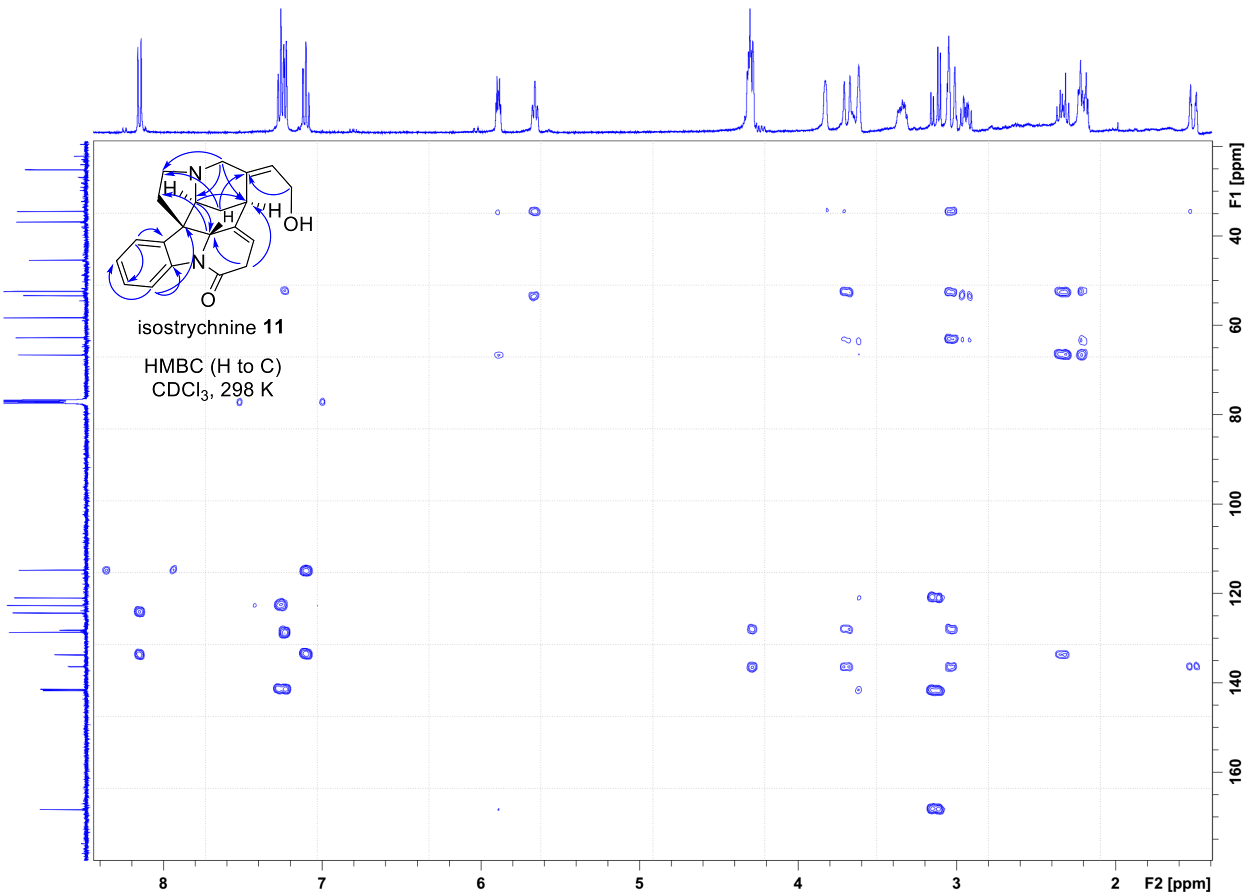

N
H
H
H
OH
N
O
isostrychnine **11**
HMBC (H to C)
$CDCl_3$, 298 K
F1 [ppm]
40
60
80
100
120
140
160
8
7
6
5
4
3
2
F2 [ppm]

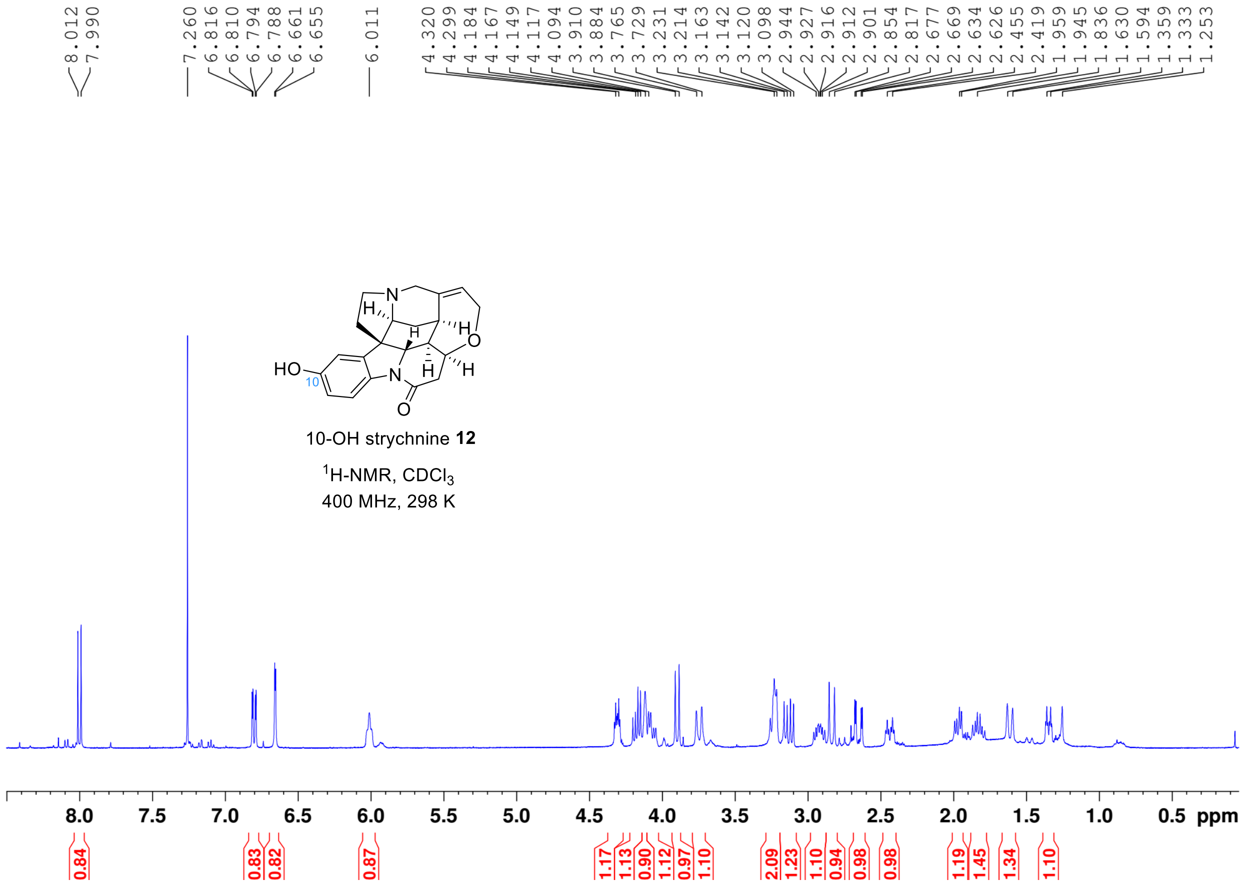
HO
10
10-OH strychnine 12
1H-NMR, CDCl3
400 MHz, 298 K
ppm

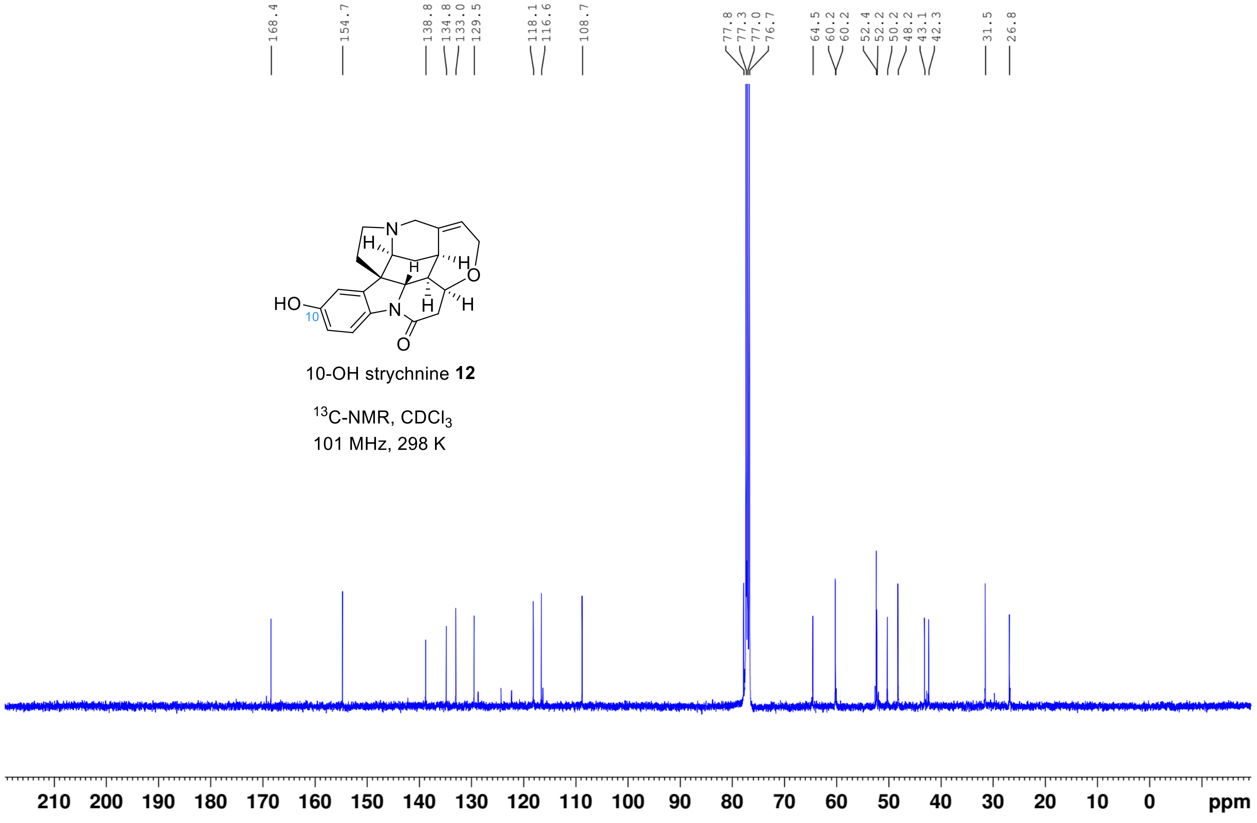
HO
10
10-OH strychnine 12
13C-NMR, CDCl3
101 MHz, 298 K
ppm

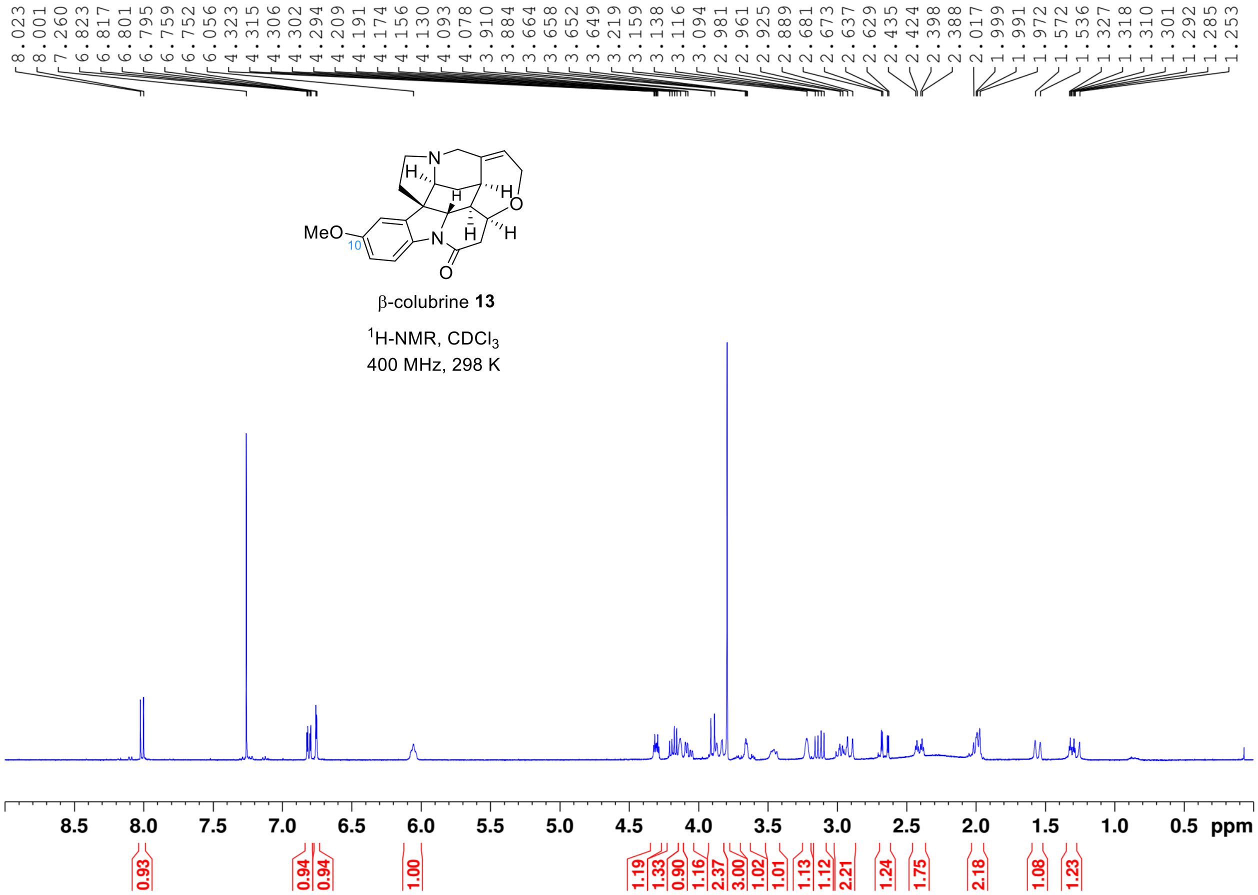
β-colubrine 13
^{1}H-NMR, $CDCl_3$
400 MHz, 298 K

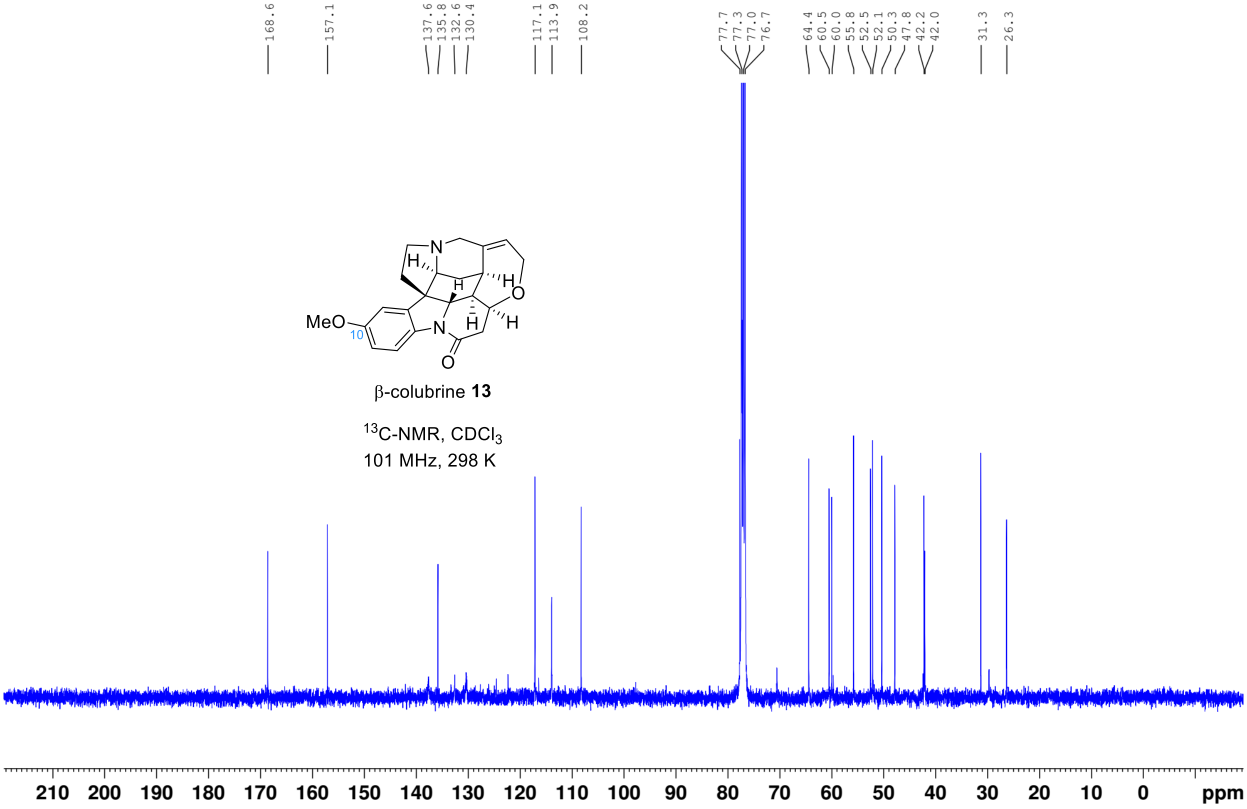
β-colubrine 13
^{13}C-NMR, $CDCl_3$
101 MHz, 298 K

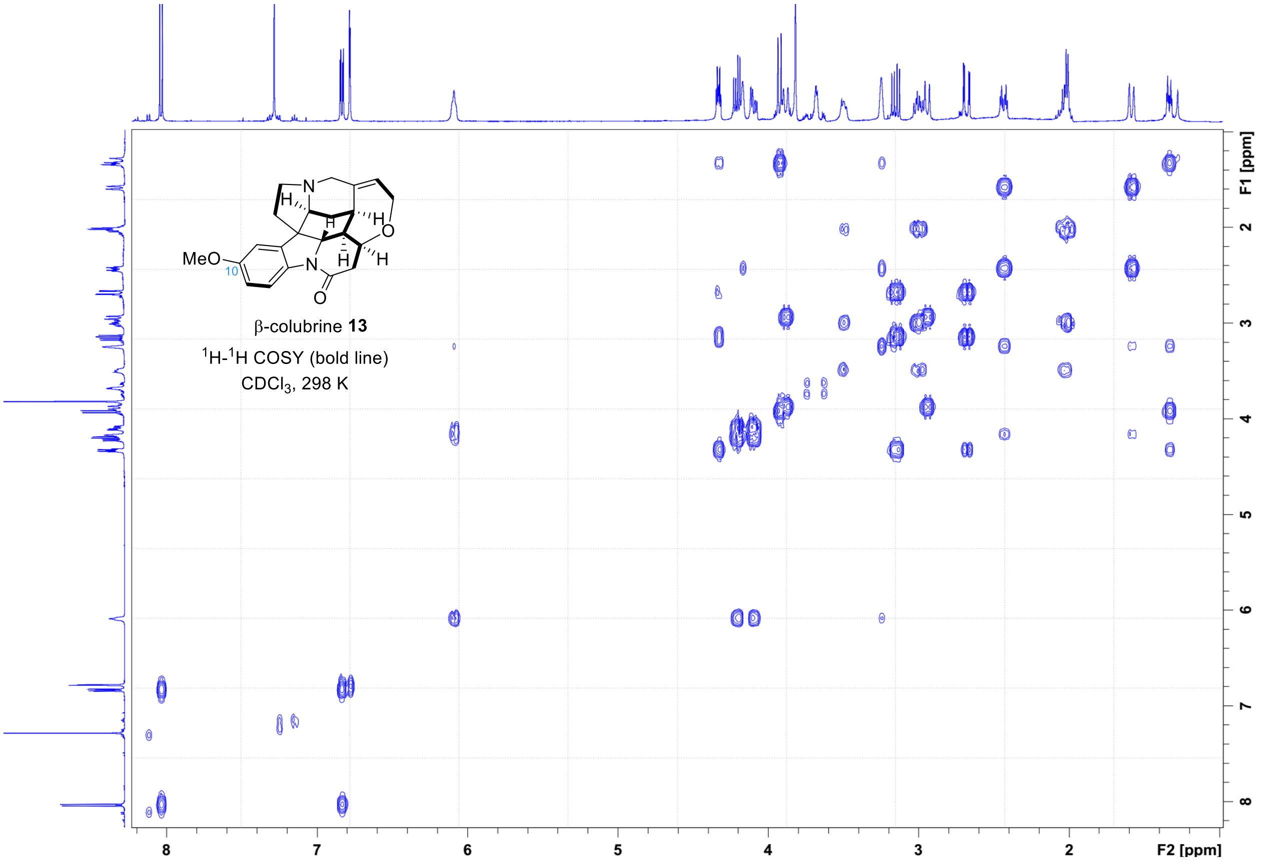
MeO
10
β-colubrine 13
1H-1H COSY (bold line)
CDCl3, 298 K
F1 [ppm]
F2 [ppm]

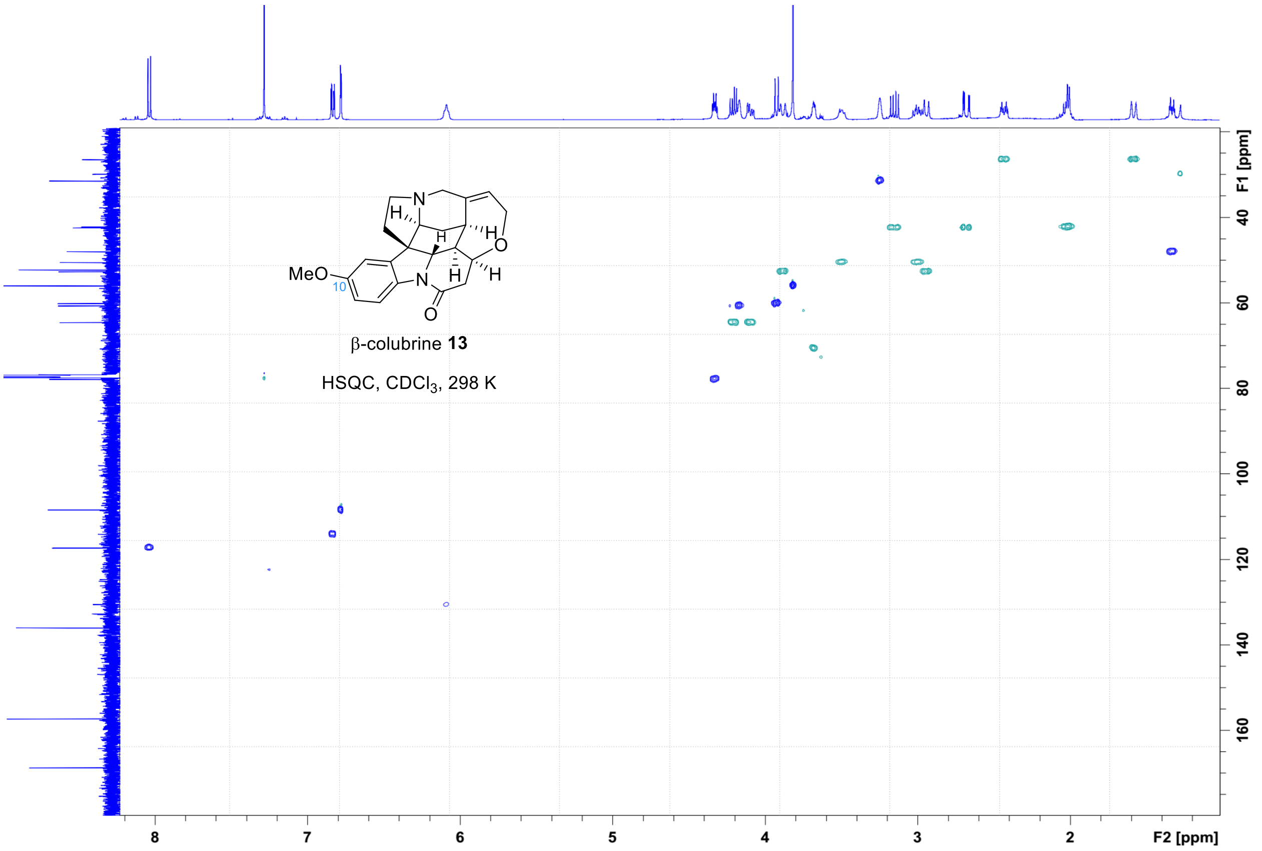
MeO
10
β-colubrine 13
HSQC, CDCl3, 298 K
F1 [ppm]
F2 [ppm]

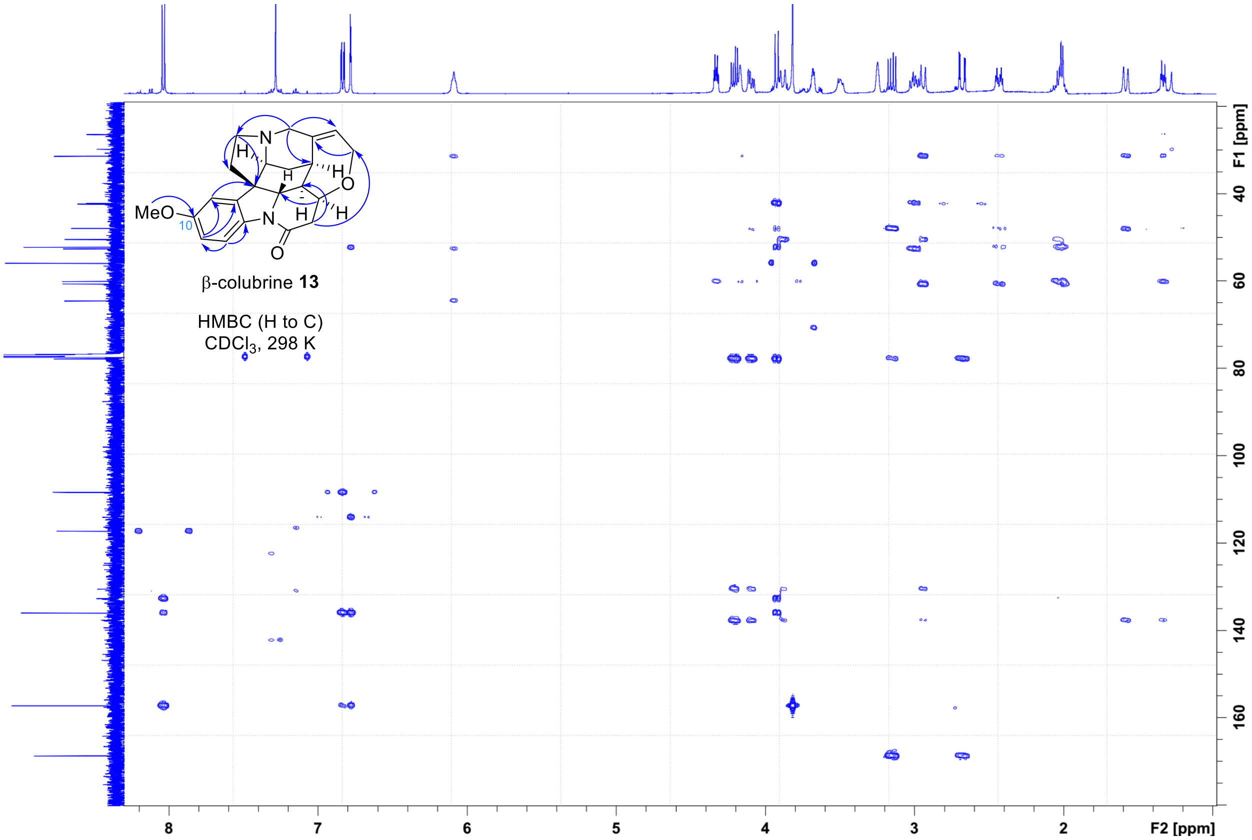
MeO
10
N
N
O
H
H
H
H
O
β-colubrine **13**
HMBC (H to C)
$CDCl_3$, 298 K
F1 [ppm]
40
60
80
100
120
140
160
8
7
6
5
4
3
2
F2 [ppm]

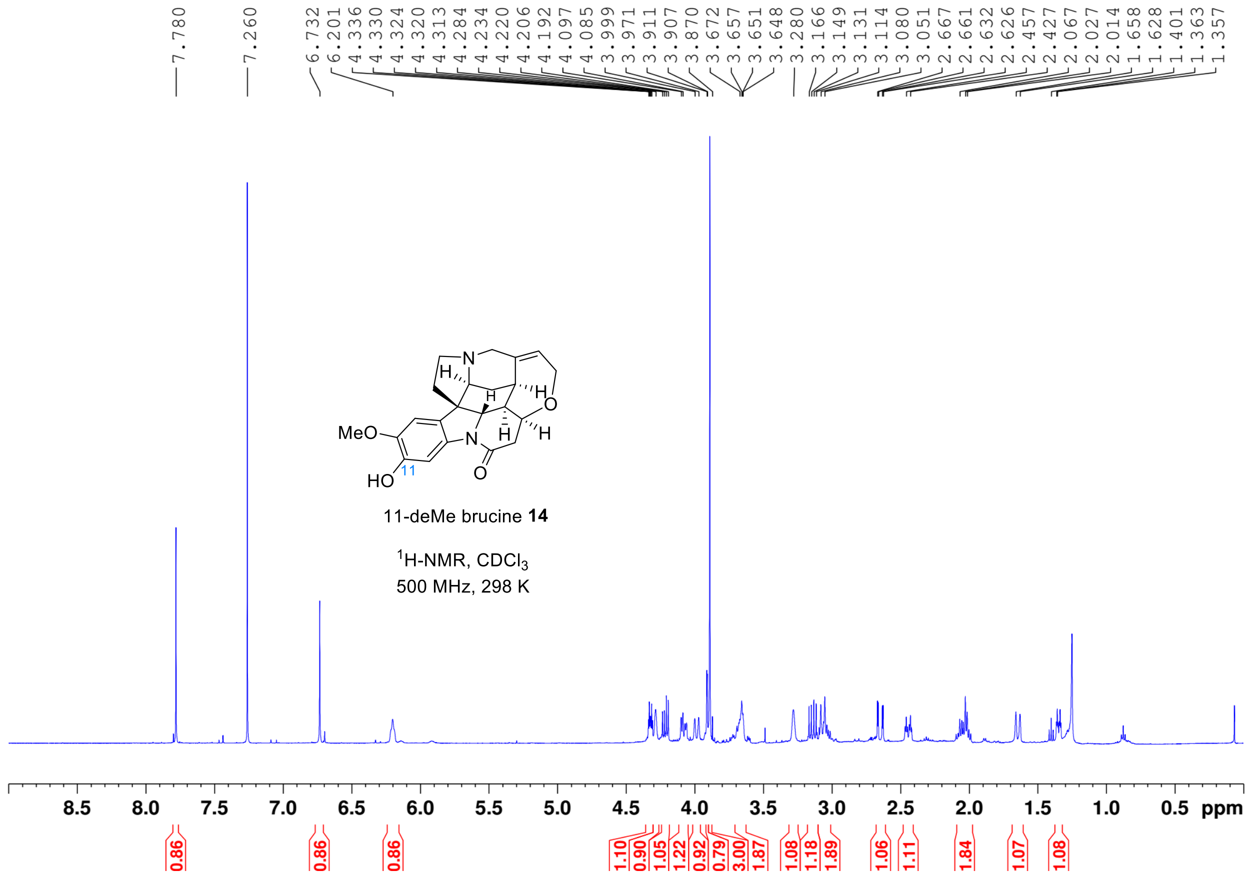
7.780
7.260
6.732
6.201
4.336
4.330
4.324
4.320
4.313
4.284
4.234
4.220
4.206
4.192
4.097
4.085
3.999
3.971
3.911
3.907
3.870
3.672
3.657
3.651
3.648
3.280
3.166
3.149
3.131
3.114
3.080
3.051
2.667
2.661
2.632
2.626
2.457
2.427
2.067
2.027
2.014
1.658
1.628
1.401
1.363
1.357
MeO
HO
11
11-deMe brucine **14**
^{1}H-NMR, $CDCl_3$
500 MHz, 298 K
8.5 8.0 7.5 7.0 6.5 6.0 5.5 5.0 4.5 4.0 3.5 3.0 2.5 2.0 1.5 1.0 0.5 ppm
0.86 0.86 0.86 1.10 0.90 1.05 1.22 0.92 0.79 3.00 1.87 1.08 1.18 1.89 1.06 1.11 1.84 1.07 1.08

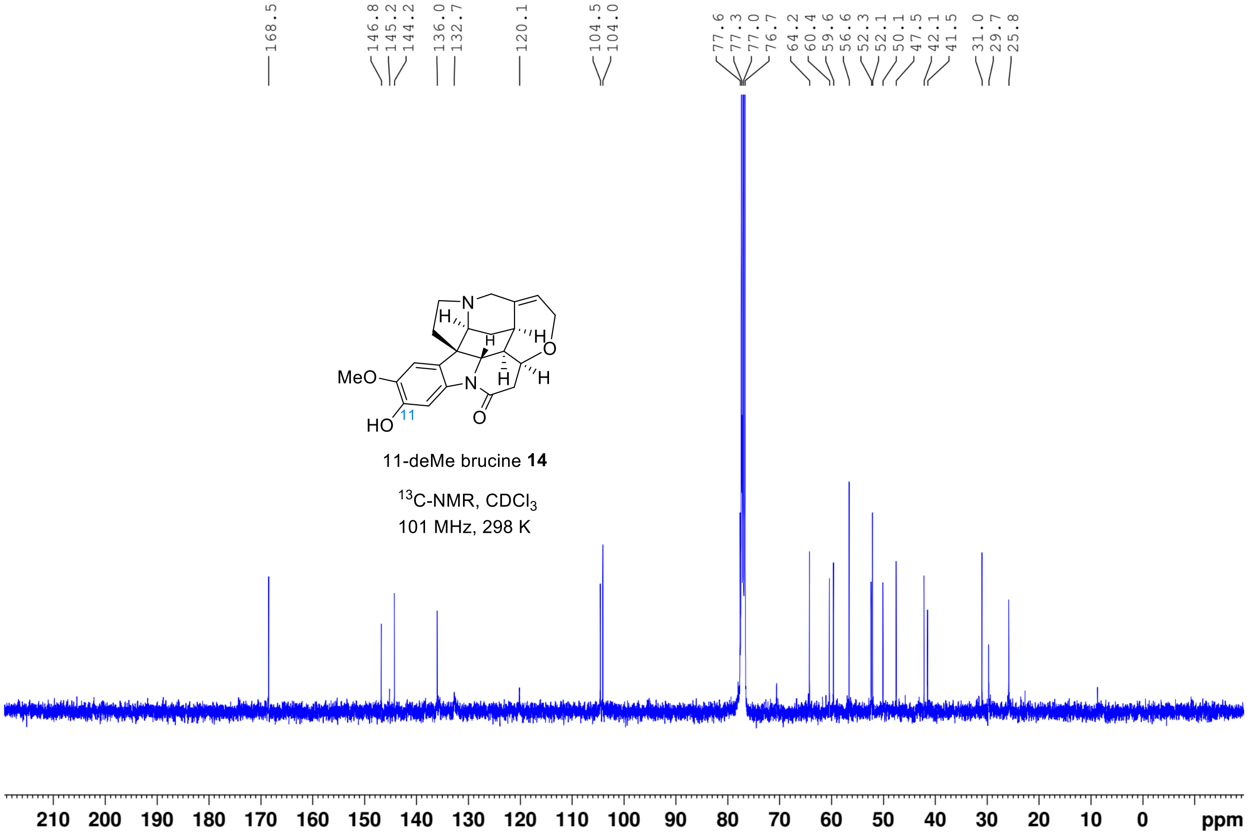
168.5
146.8
145.2
144.2
136.0
132.7
120.1
104.5
104.0
77.6
77.3
77.0
76.7
64.2
60.4
59.6
56.6
52.3
52.1
50.1
47.5
42.1
41.5
31.0
29.7
25.8
MeO
HO
11
11-deMe brucine **14**
^{13}C-NMR, $CDCl_3$
101 MHz, 298 K
210 200 190 180 170 160 150 140 130 120 110 100 90 80 70 60 50 40 30 20 10 0 ppm

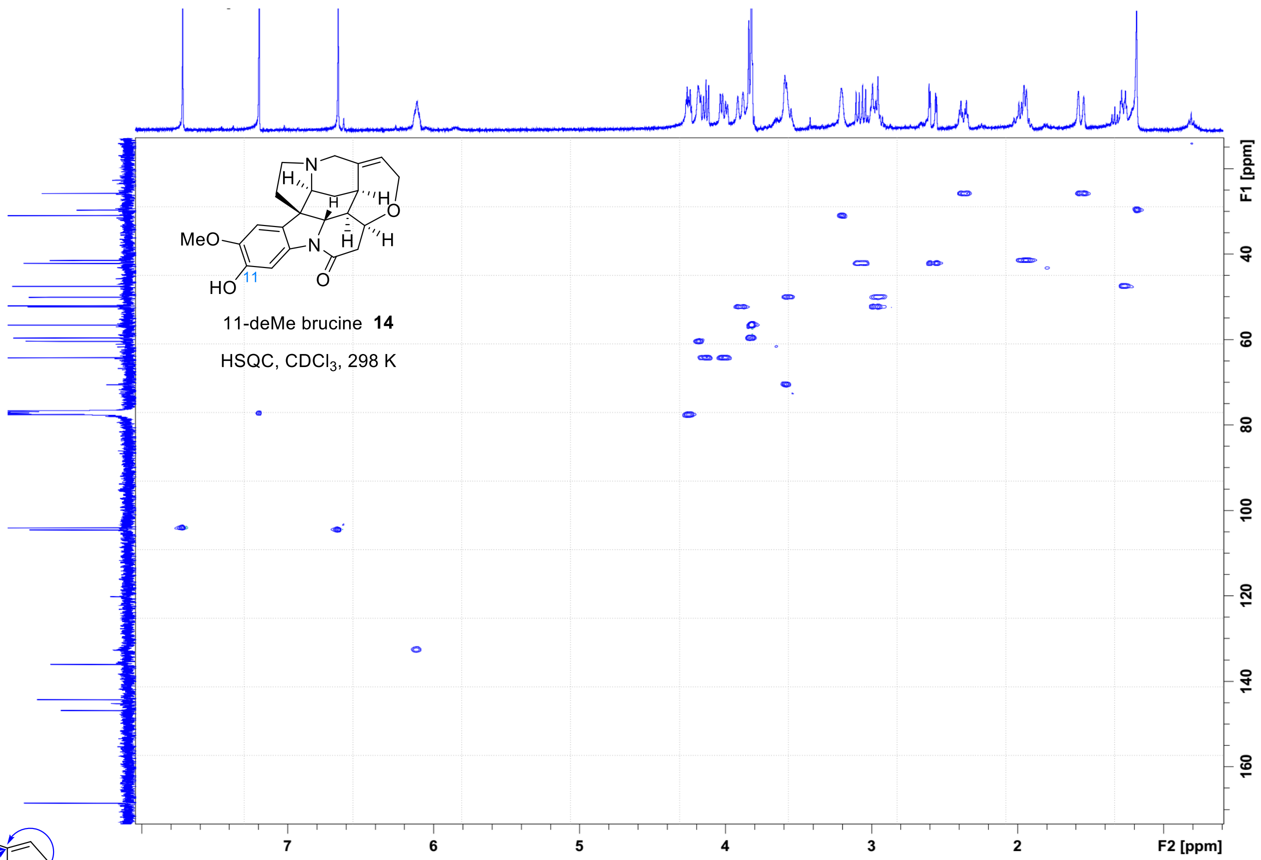
MeO
HO
11
11-deMe brucine **14**
HSQC, $CDCl_3$, 298 K
F1 [ppm]
F2 [ppm]

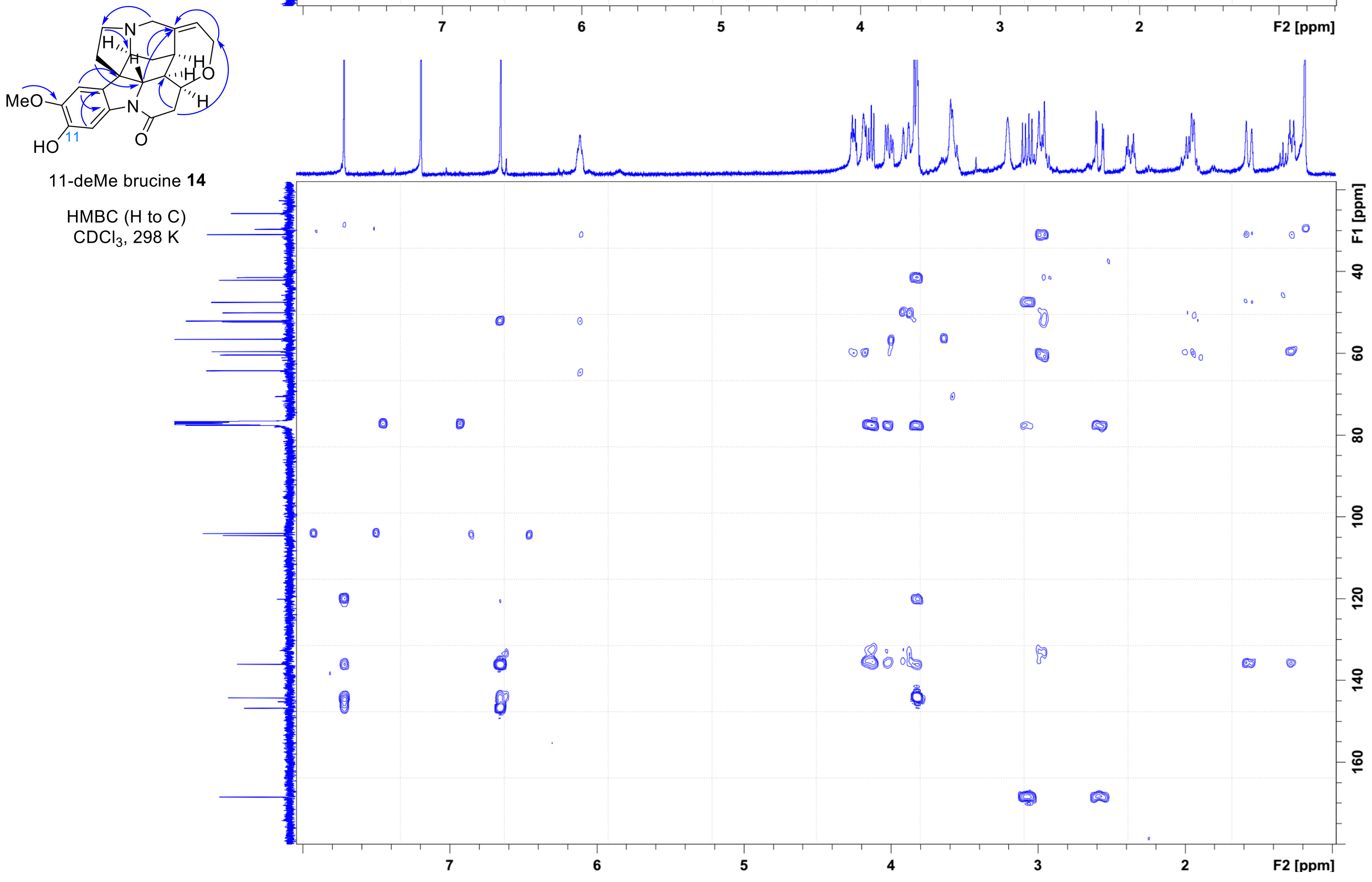
MeO
HO
11
11-deMe brucine **14**
HMBC (H to C)
$CDCl_3$, 298 K
F1 [ppm]
F2 [ppm]

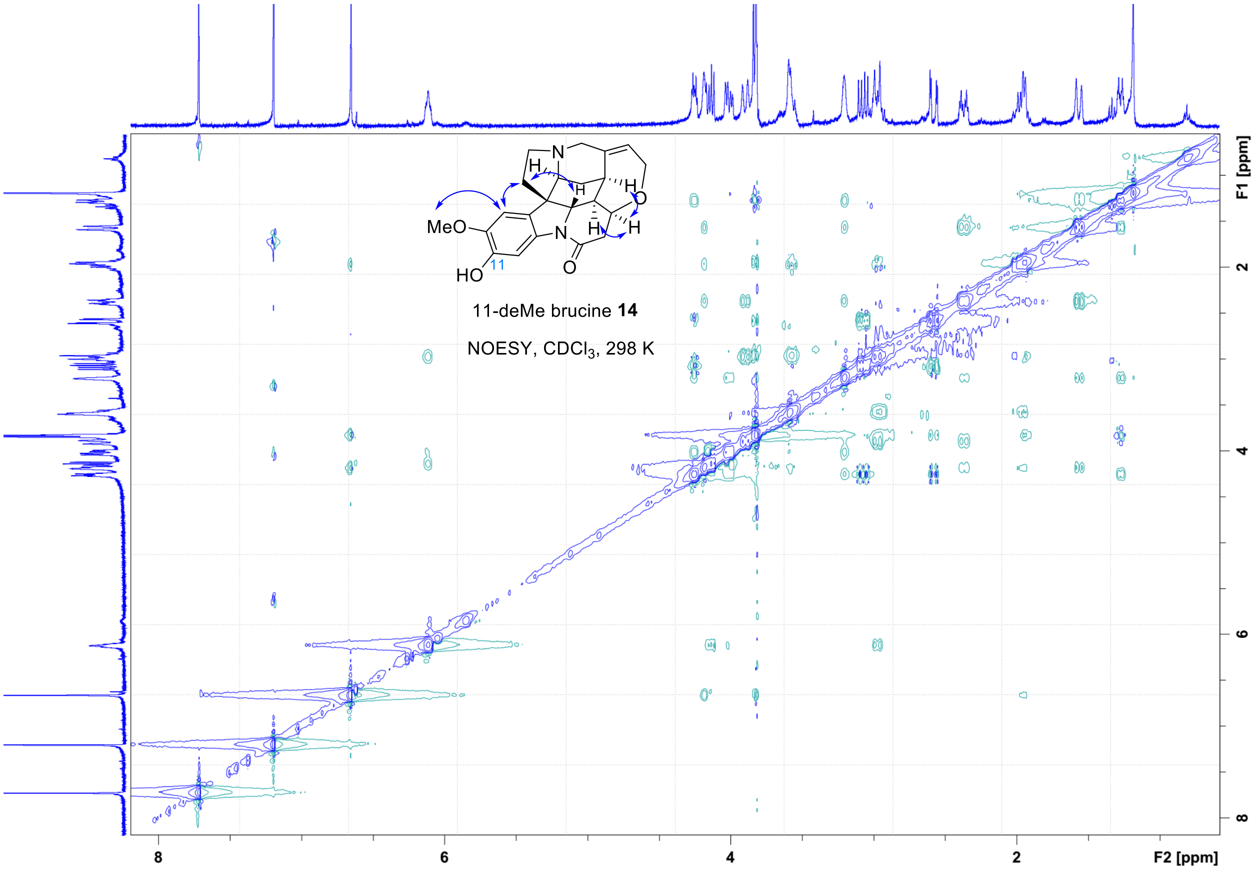
MeO
HO
11
11-deMe brucine **14**
NOESY, $CDCl_3$, 298 K
F1 [ppm]
F2 [ppm]
8
6
4
2

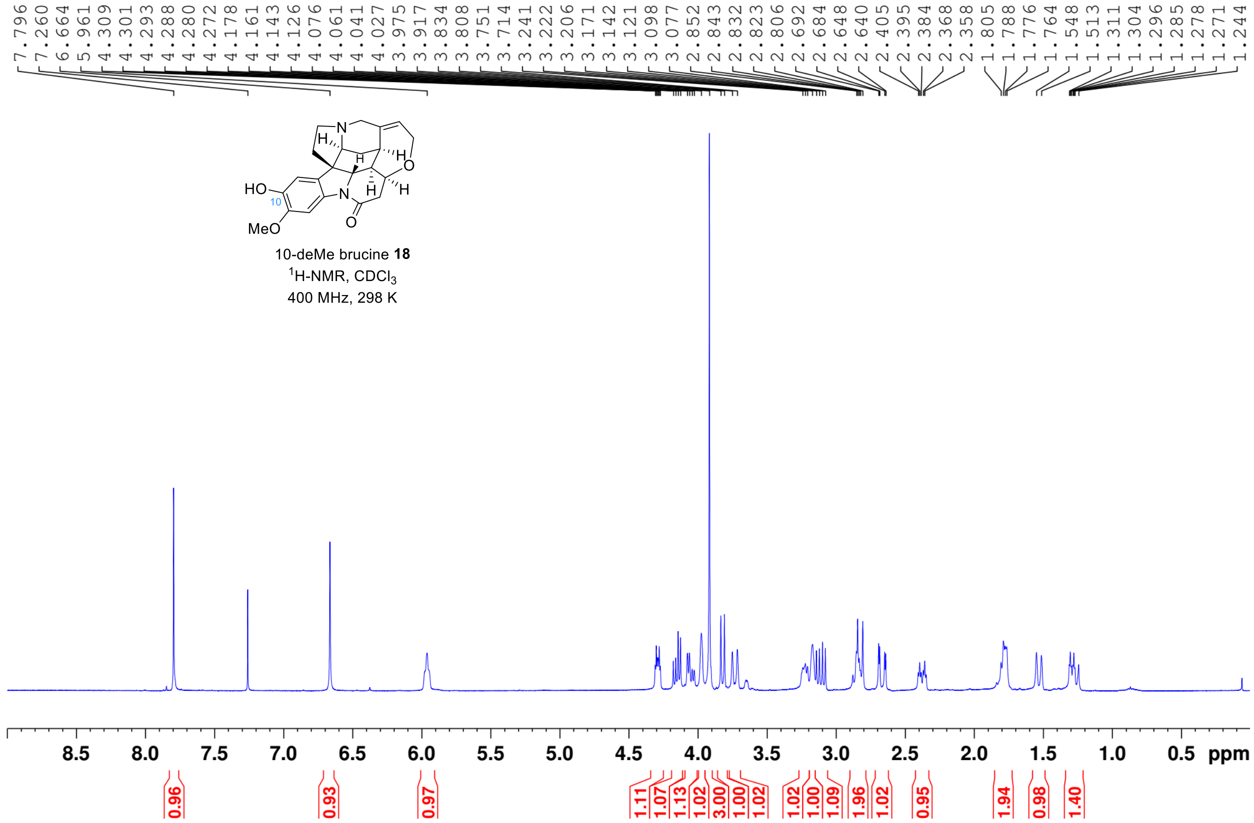
7.796
7.260
6.664
5.961
4.309
4.301
4.293
4.288
4.280
4.272
4.178
4.161
4.143
4.126
4.076
4.061
4.041
4.027
3.975
3.917
3.834
3.808
3.751
3.714
3.241
3.222
3.206
3.171
3.142
3.121
3.098
3.077
2.852
2.843
2.832
2.823
2.806
2.692
2.684
2.648
2.640
2.405
2.395
2.384
2.368
2.358
1.805
1.788
1.776
1.764
1.548
1.513
1.311
1.304
1.296
1.285
1.278
1.271
1.244
HO
10
MeO
N
O
H
10-deMe brucine **18**
^{1}H-NMR, $CDCl_3$
400 MHz, 298 K
8.5
8.0
7.5
7.0
6.5
6.0
5.5
5.0
4.5
4.0
3.5
3.0
2.5
2.0
1.5
1.0
0.5
ppm
0.96
0.93
0.97
1.11
1.07
1.13
1.02
3.00
1.00
1.02
1.02
1.00
1.09
1.96
1.02
0.95
1.94
0.98
1.40

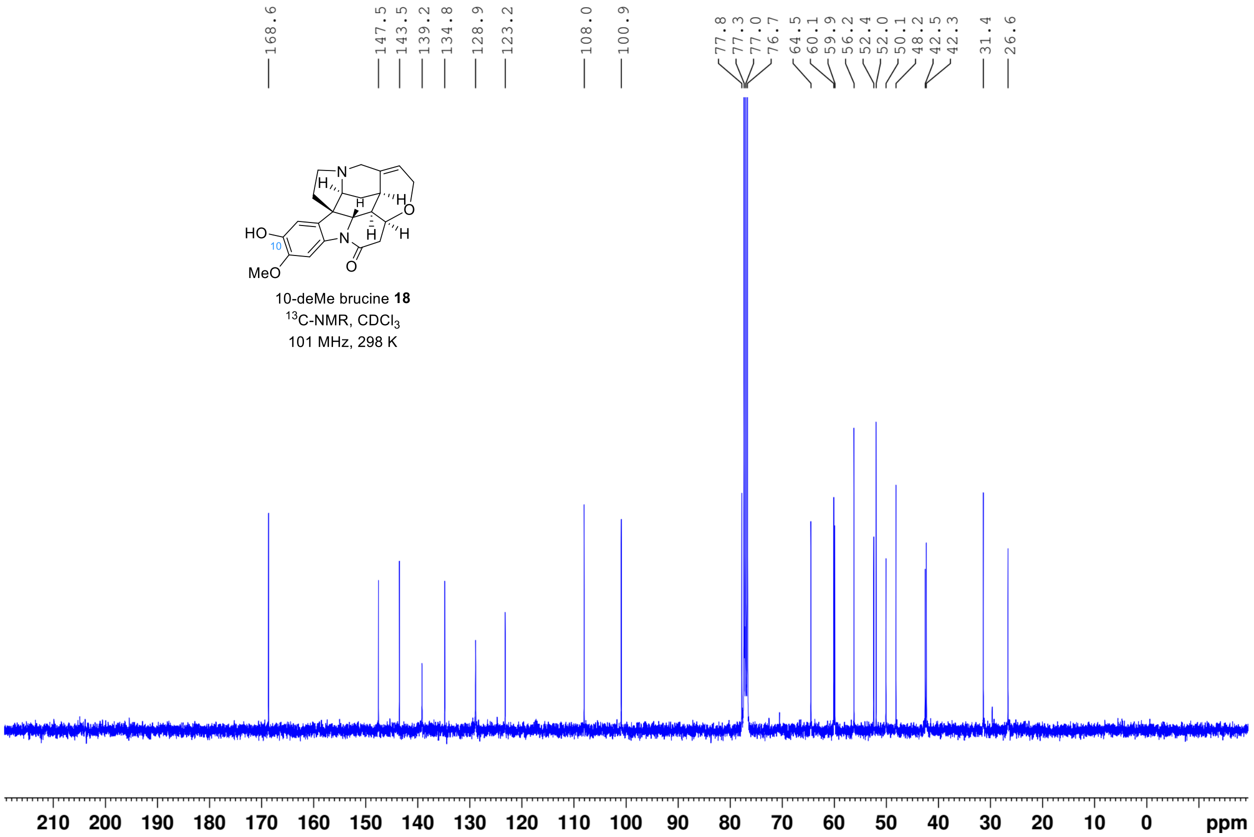
168.6
147.5
143.5
139.2
134.8
128.9
123.2
108.0
100.9
77.8
77.3
77.0
76.7
64.5
60.1
59.9
56.2
52.4
52.0
50.1
48.2
42.5
42.3
31.4
26.6
HO
10
MeO
N
O
H
10-deMe brucine **18**
^{13}C-NMR, $CDCl_3$
101 MHz, 298 K
210
200
190
180
170
160
150
140
130
120
110
100
90
80
70
60
50
40
30
20
10
0
ppm

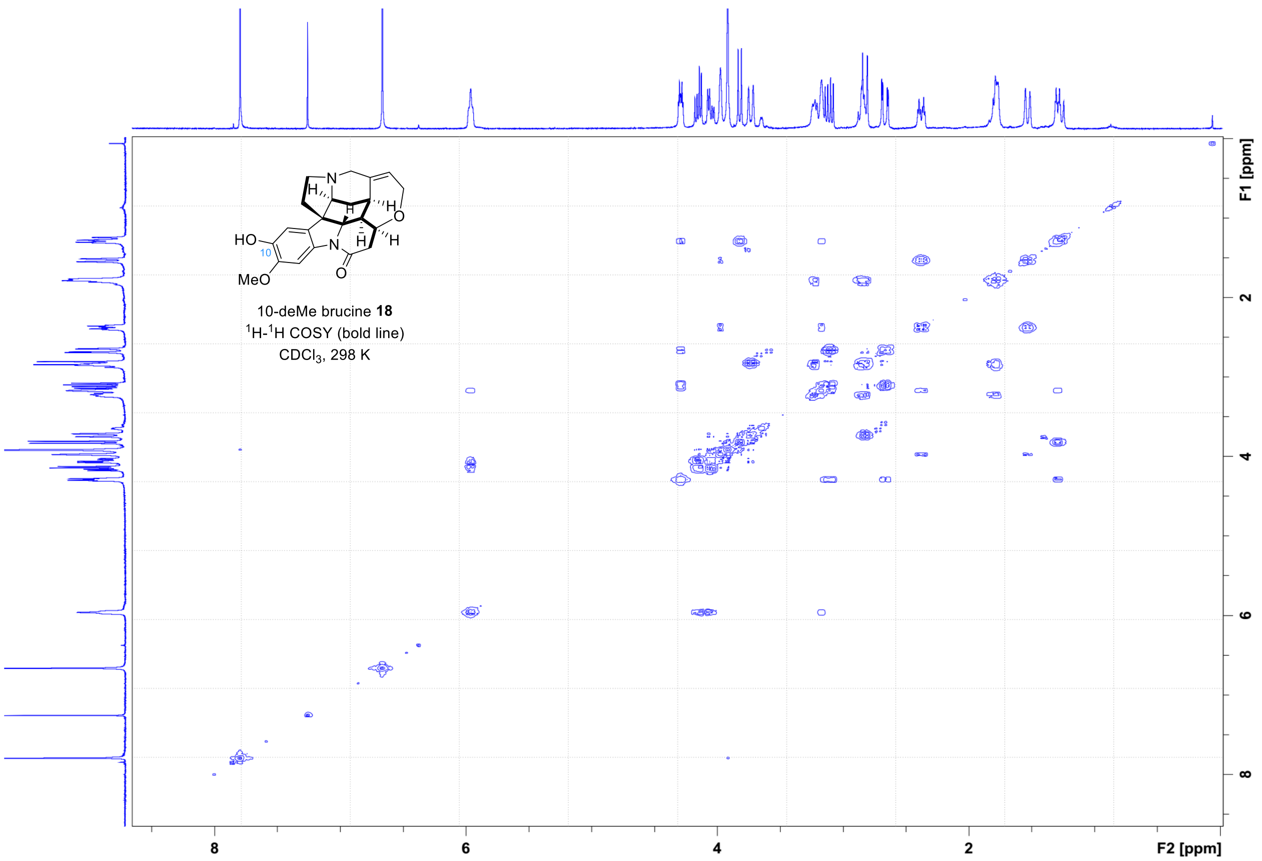
HO
10
MeO
N
O
10-deMe brucine **18**
^{1}H-^{1}H COSY (bold line)
$CDCl_3$, 298 K
F1 [ppm]
F2 [ppm]

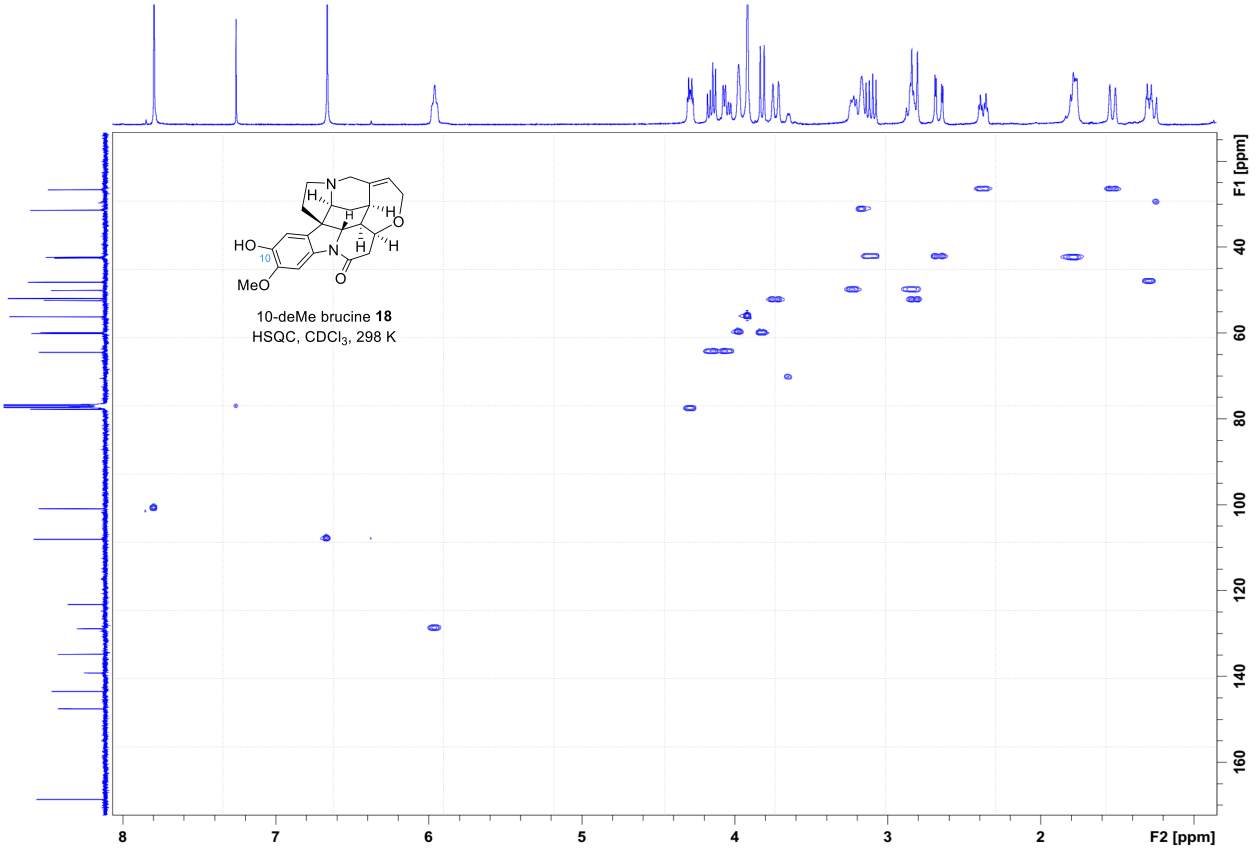
HO
10
MeO
N
O
10-deMe brucine **18**
HSQC, $CDCl_3$, 298 K
F1 [ppm]
F2 [ppm]

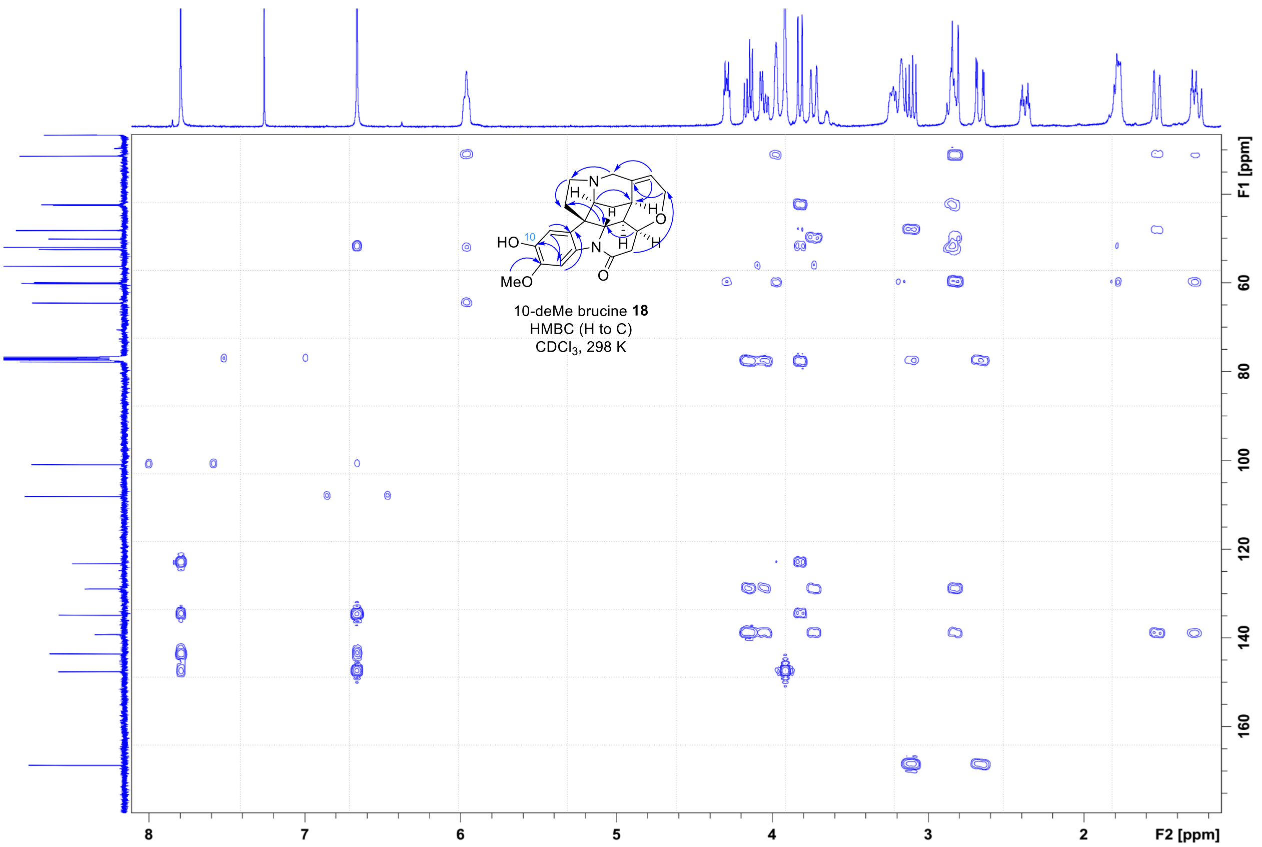
HO
10
MeO
N
O
10-deMe brucine **18**
HMBC (H to C)
$CDCl_3$, 298 K
F1 [ppm]
F2 [ppm]

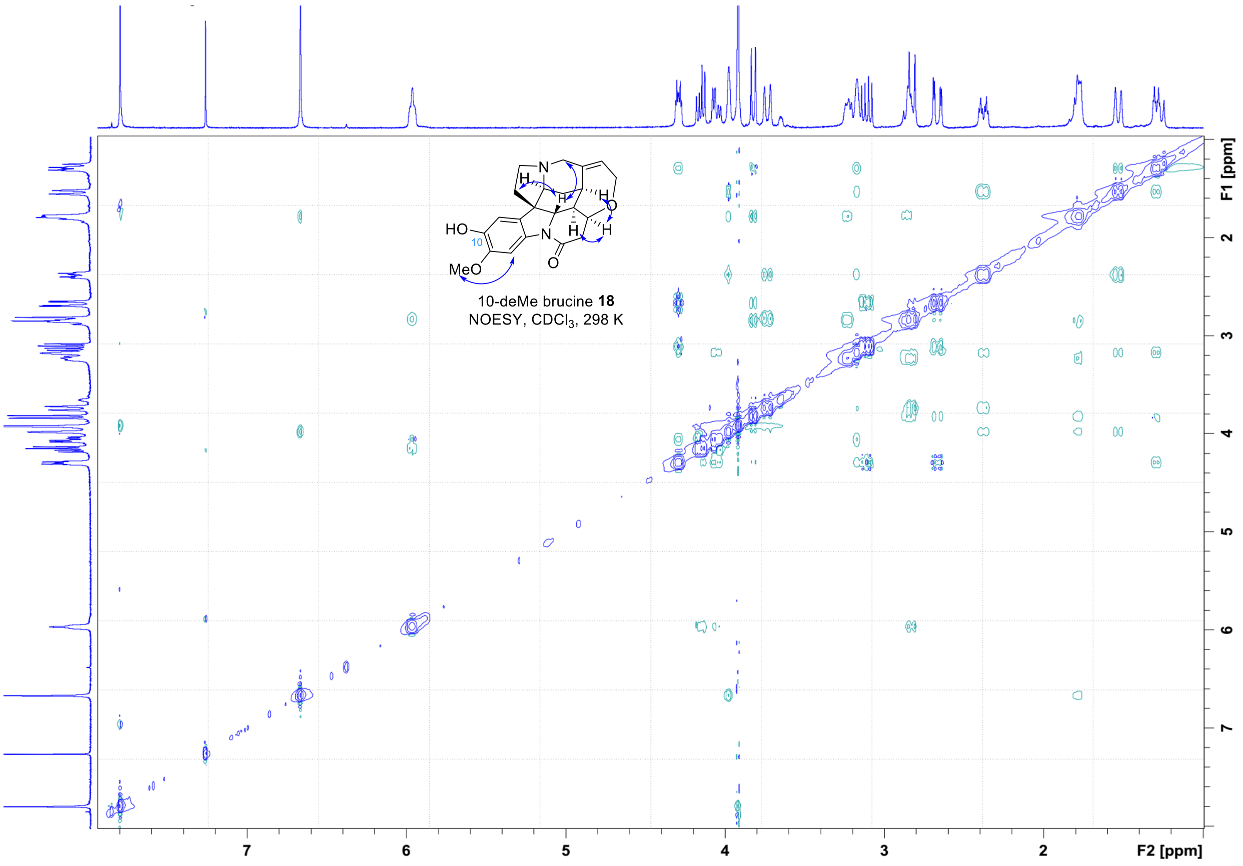
HO
10
MeO
N
O
10-deMe brucine **18**
NOESY, $CDCl_3$, 298 K
F1 [ppm]
F2 [ppm]

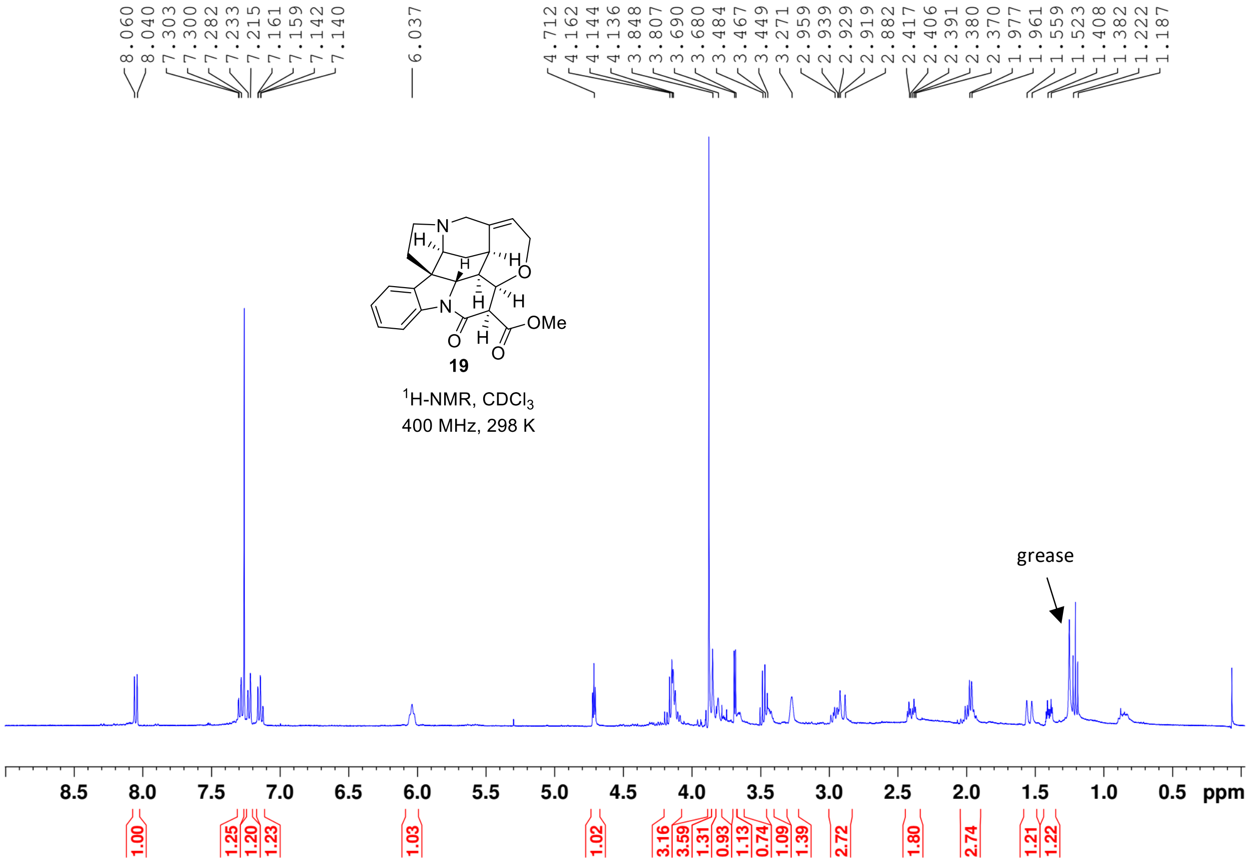
8.060 8.040 7.303 7.300 7.282 7.233 7.215 7.161 7.159 7.142 7.140
6.037
4.712 4.162 4.144 4.136 3.848 3.807 3.690 3.680 3.484 3.467 3.449 3.271 2.959 2.939 2.929 2.919 2.882 2.417 2.406 2.391 2.380 2.370 1.977 1.961 1.559 1.523 1.408 1.382 1.222 1.187
19
^{1}H-NMR, $CDCl_3$
400 MHz, 298 K
grease
8.5 8.0 7.5 7.0 6.5 6.0 5.5 5.0 4.5 4.0 3.5 3.0 2.5 2.0 1.5 1.0 0.5 ppm
1.00 1.25 1.20 1.23 1.03 1.02 3.16 3.59 1.31 0.93 1.13 0.74 1.09 1.39 2.72 1.80 2.74 1.21 1.22

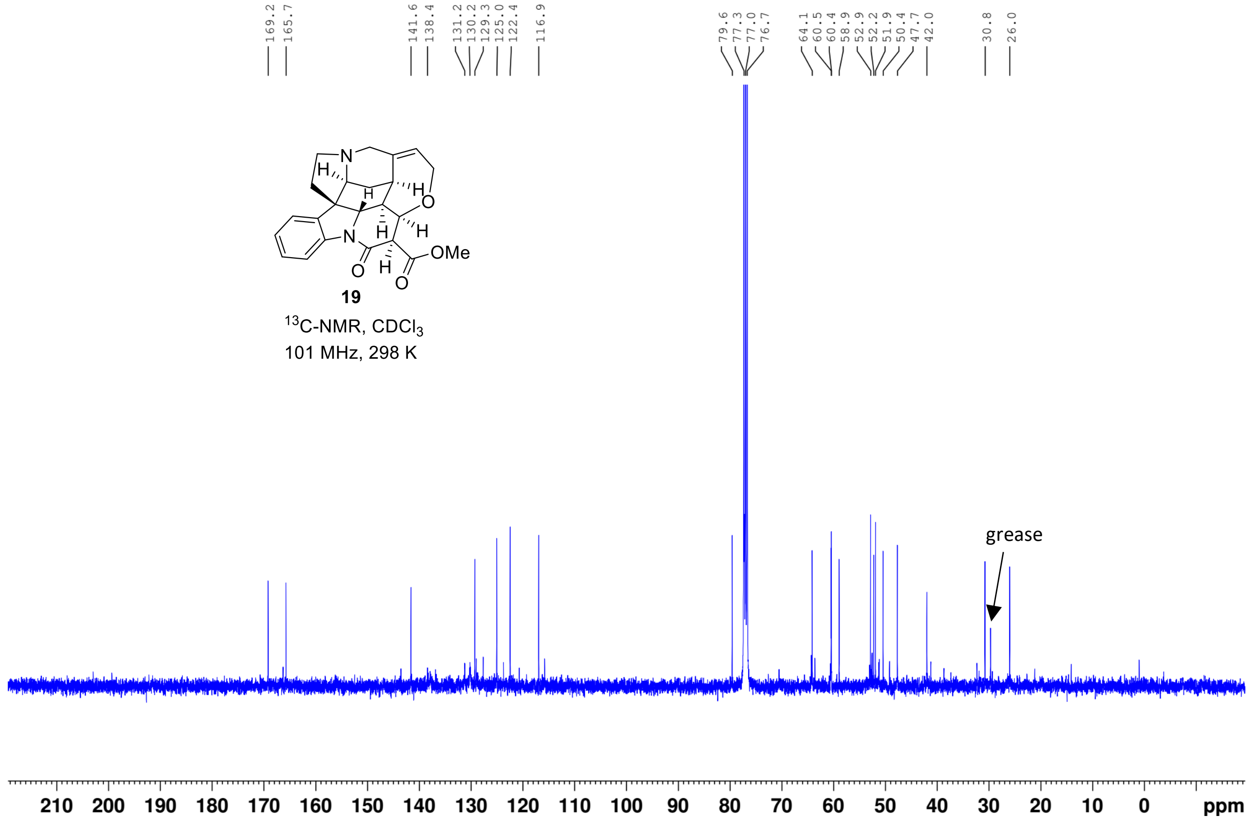
169.2 165.7 141.6 138.4 131.2 130.2 129.3 125.0 122.4 116.9 79.6 77.3 77.0 76.7 64.1 60.5 60.4 58.9 52.9 52.2 51.9 50.4 47.7 42.0 30.8 26.0
19
^{13}C-NMR, $CDCl_3$
101 MHz, 298 K
grease
210 200 190 180 170 160 150 140 130 120 110 100 90 80 70 60 50 40 30 20 10 0 ppm

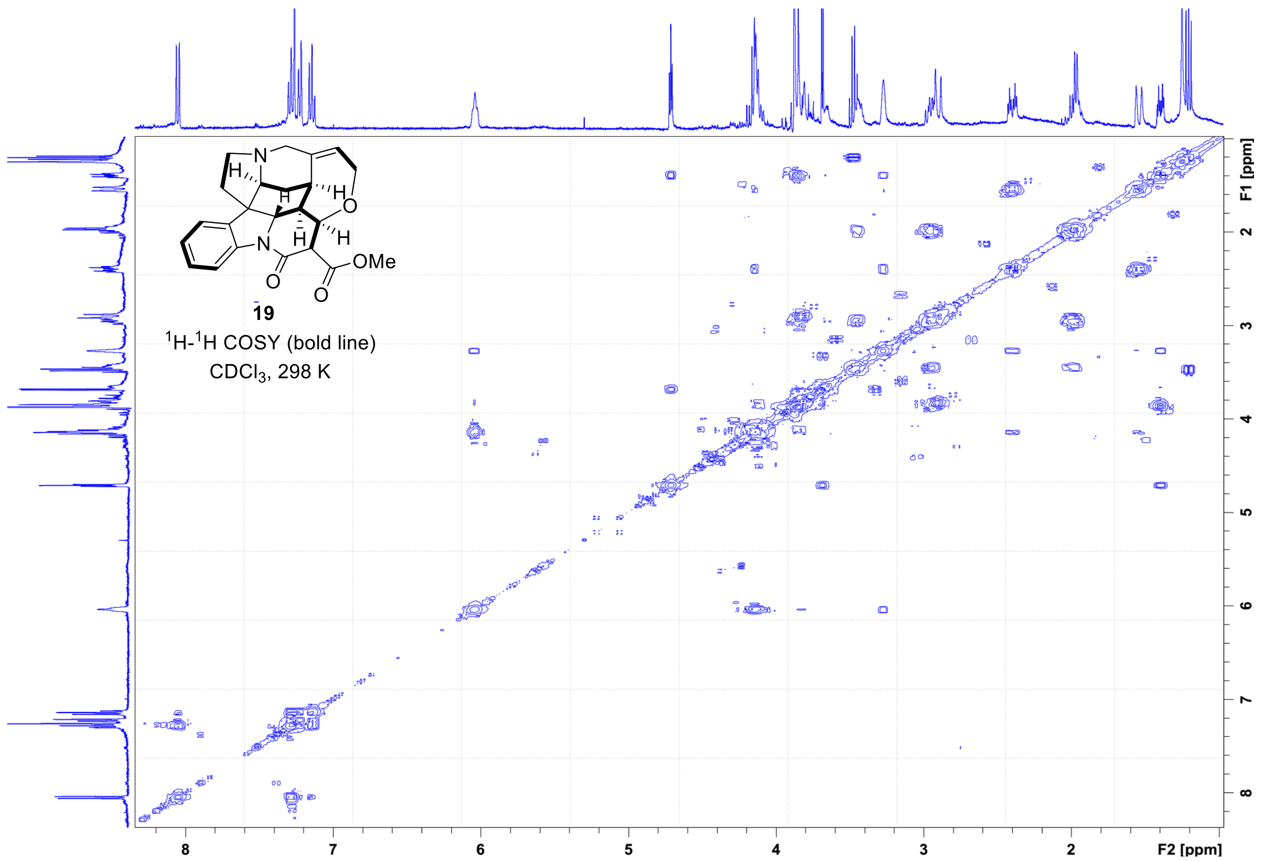

19
^{1}H-^{1}H COSY (bold line)
CDCl$_3$, 298 K
OMe
F1 [ppm]
F2 [ppm]

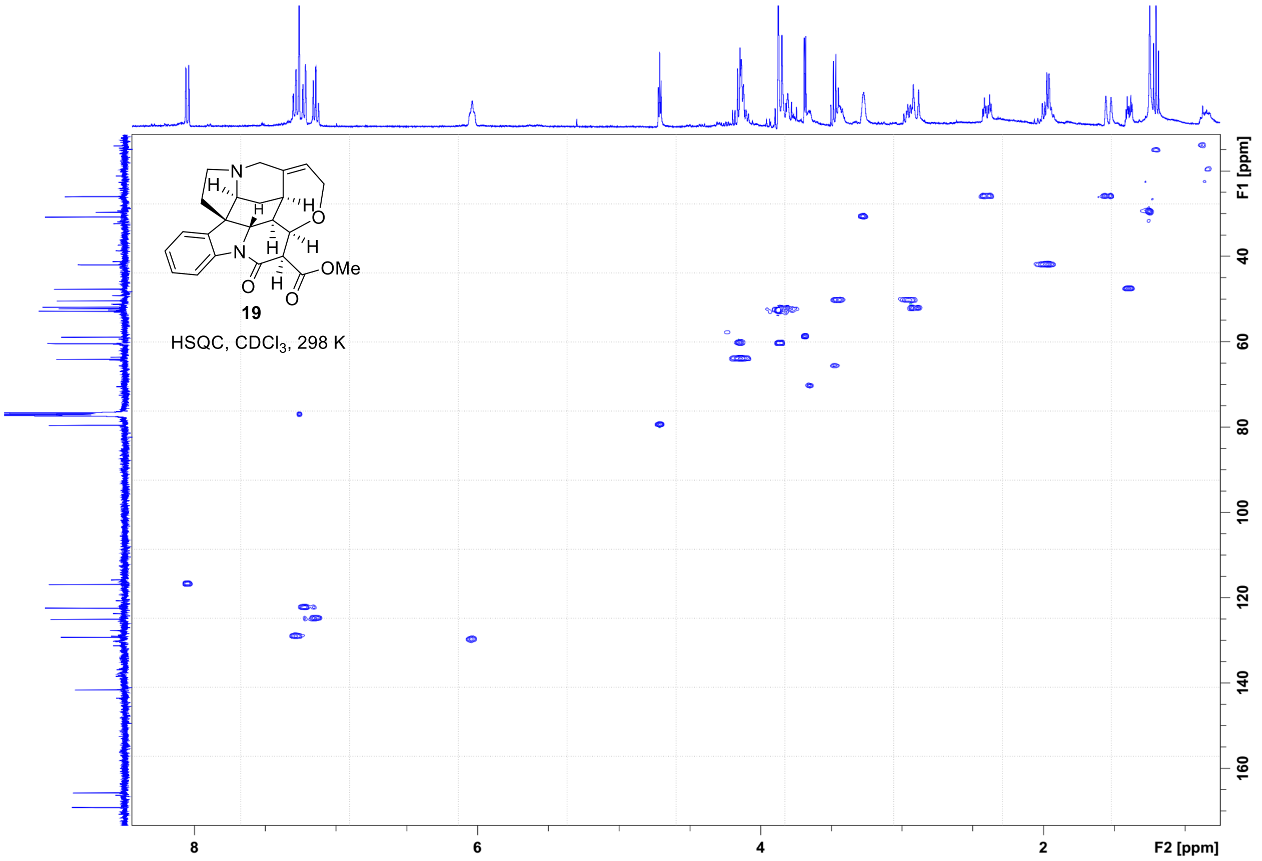

19
HSQC, CDCl$_3$, 298 K
OMe
F1 [ppm]
F2 [ppm]

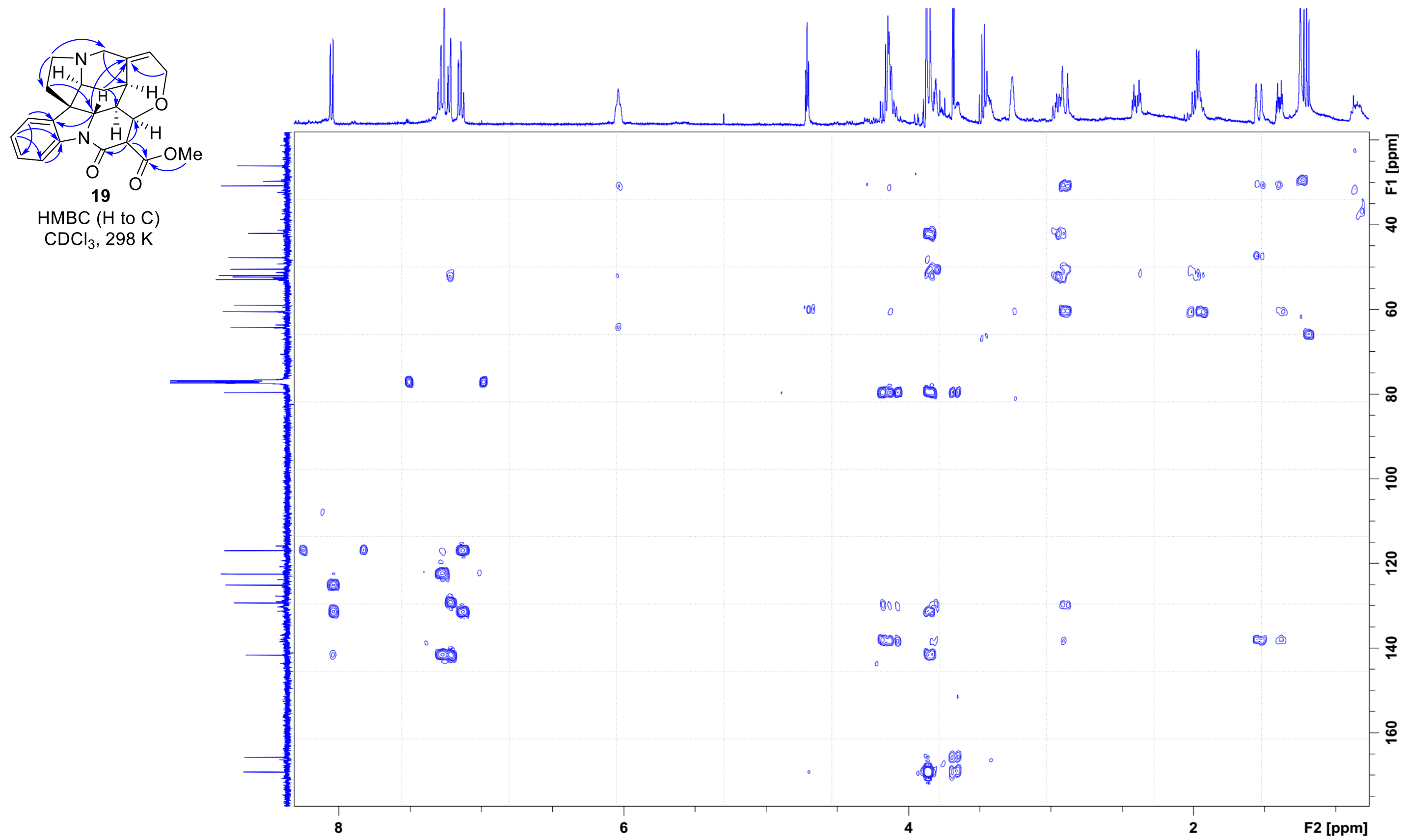

N
H
H
H
H
O
N
O
OMe
O
19
HMBC (H to C)
CDCl3, 298 K
F1 [ppm]
F2 [ppm]

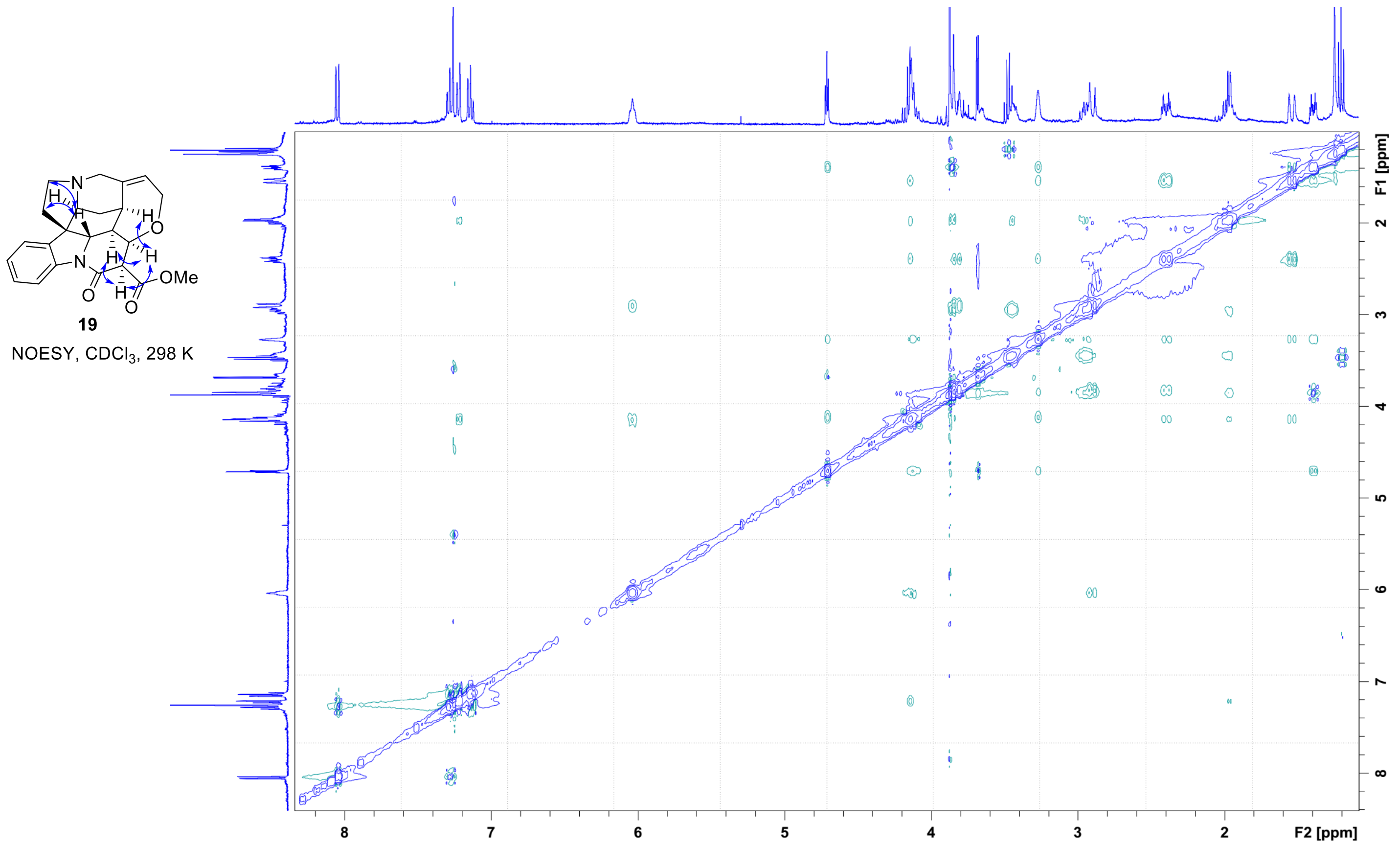

N
H
H
H
O
H
N
O
H
OMe
O
19
NOESY, CDCl3, 298 K
F1 [ppm]
F2 [ppm]

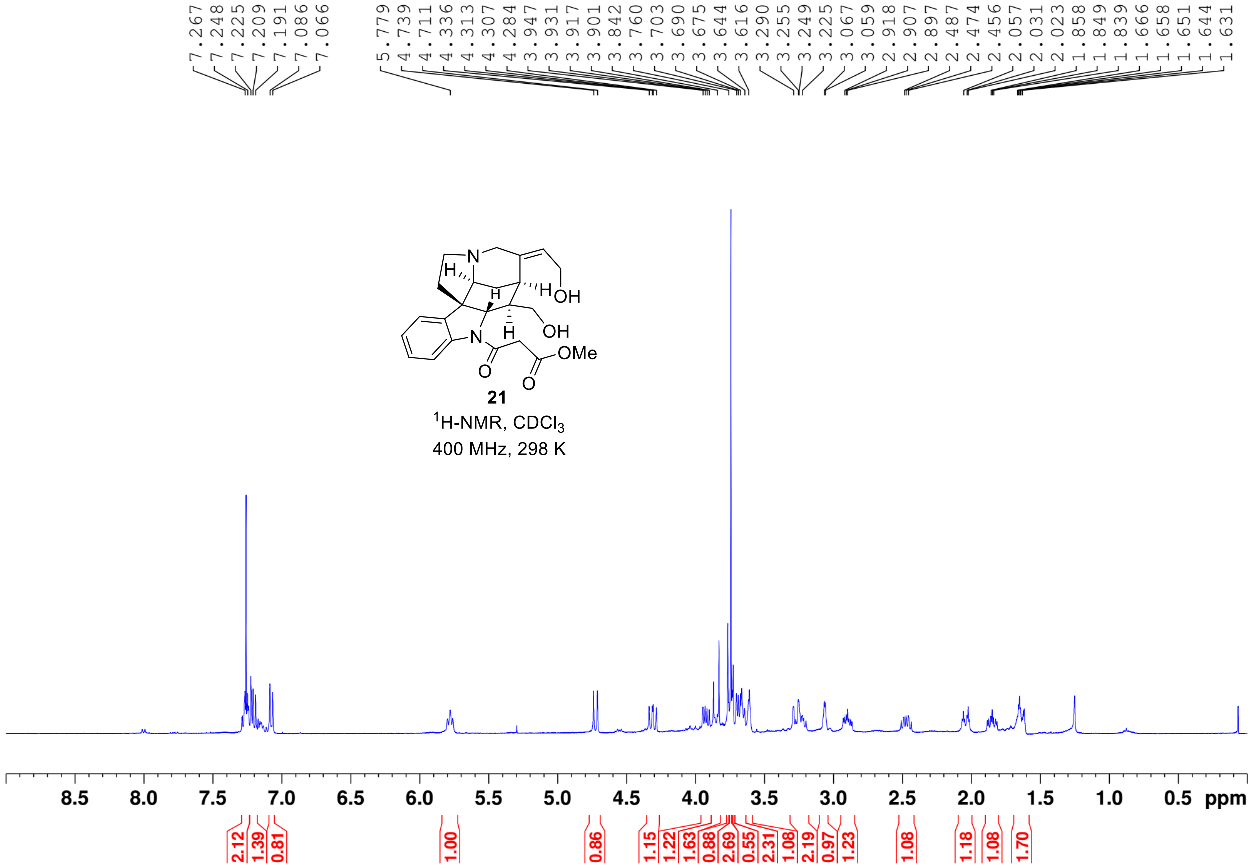
7.267 7.248 7.225 7.209 7.191 7.086 7.066 5.779 4.739 4.711 4.336 4.313 4.307 4.284 3.947 3.931 3.917 3.901 3.842 3.760 3.703 3.690 3.675 3.644 3.616 3.290 3.255 3.249 3.225 3.067 3.059 2.918 2.907 2.897 2.487 2.474 2.456 2.057 2.031 2.023 1.858 1.849 1.839 1.666 1.658 1.651 1.644 1.631
H
N
H
H
OH
H
OH
N
O
OMe
O
21
1H-NMR, CDCl3
400 MHz, 298 K
8.5 8.0 7.5 7.0 6.5 6.0 5.5 5.0 4.5 4.0 3.5 3.0 2.5 2.0 1.5 1.0 0.5 ppm
2.12 1.39 0.81 1.00 0.86 1.15 1.22 1.63 0.88 2.69 0.55 2.31 1.08 2.19 0.97 1.23 1.08 1.18 1.08 1.70

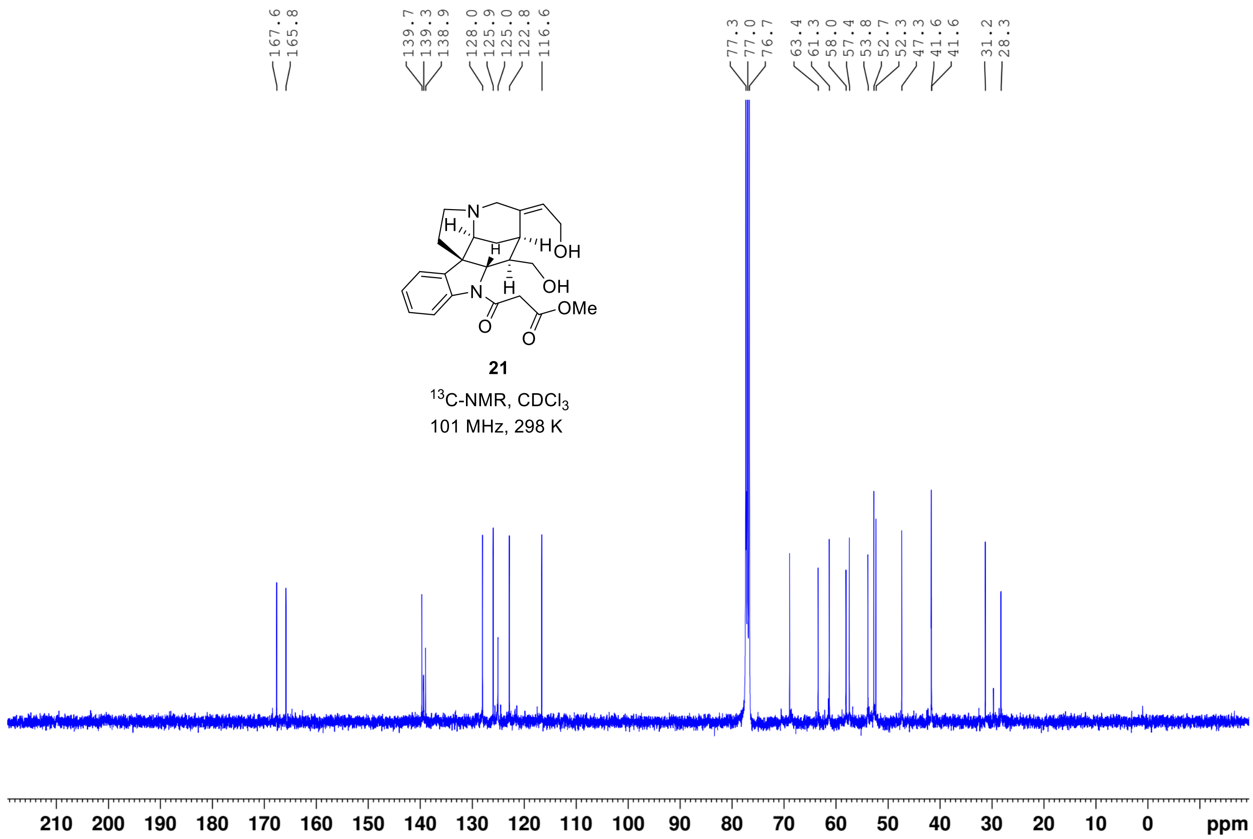
167.6 165.8 139.7 139.3 138.9 128.0 125.9 125.0 122.8 116.6 77.3 77.0 76.7 63.4 61.3 58.0 57.4 53.8 52.7 52.3 47.3 41.6 41.6 31.2 28.3
H
N
H
H
OH
H
OH
N
O
OMe
O
21
13C-NMR, CDCl3
101 MHz, 298 K
210 200 190 180 170 160 150 140 130 120 110 100 90 80 70 60 50 40 30 20 10 0 ppm

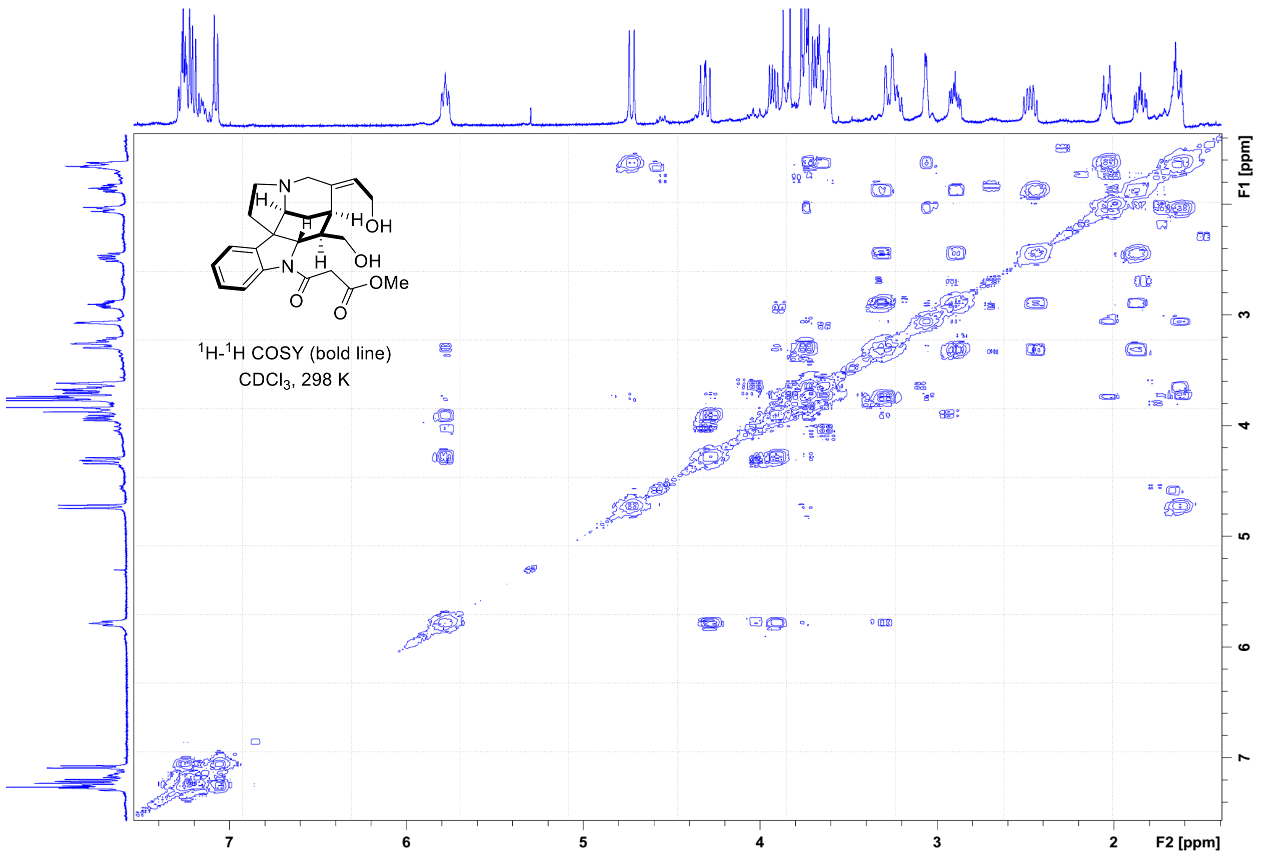
N
H
H
H
OH
H
OH
N
O
OMe
O
^{1}H-^{1}H COSY (bold line)
$CDCl_3$, 298 K
F1 [ppm]
F2 [ppm]
7
6
5
4
3
2

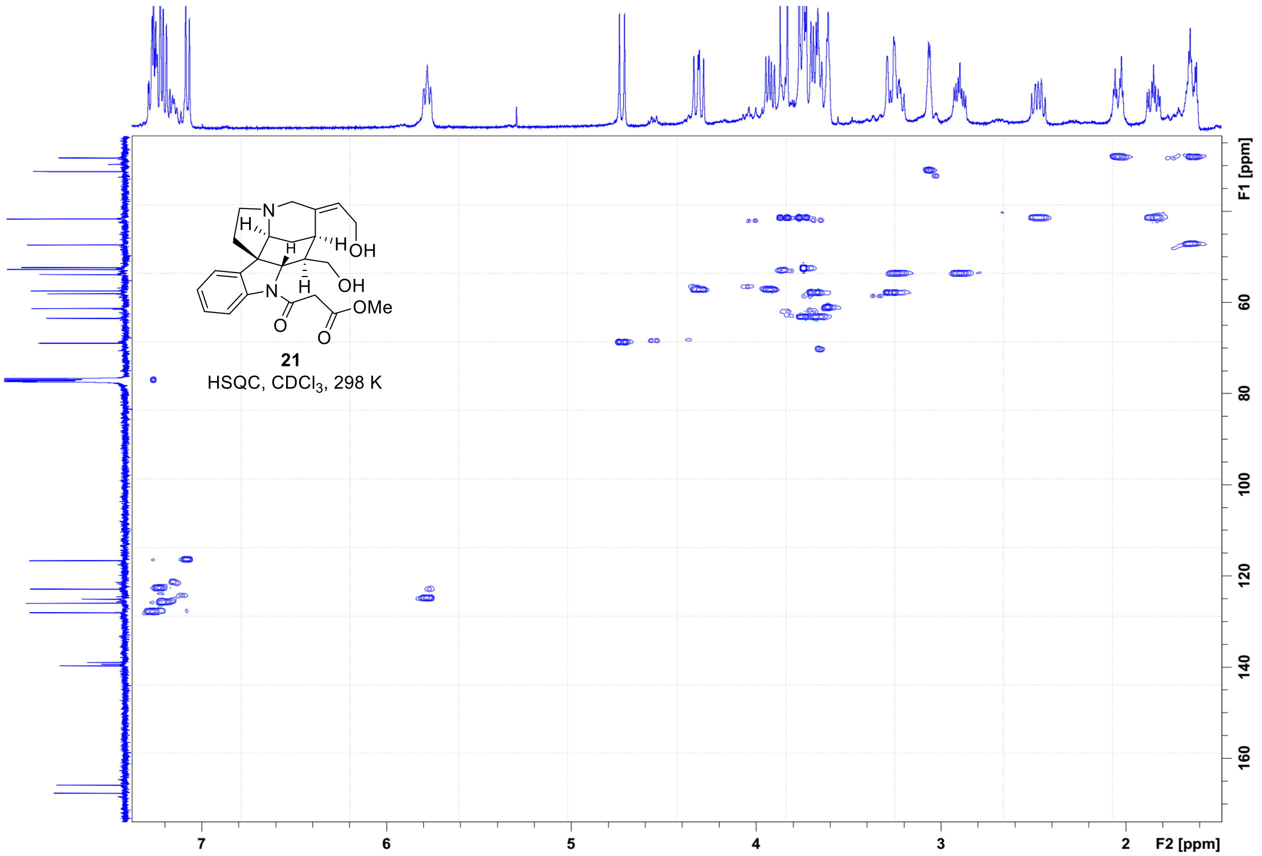
N
H
H
H
OH
H
OH
N
O
OMe
O
21
HSQC, $CDCl_3$, 298 K
F1 [ppm]
F2 [ppm]
60
80
100
120
140
160
7
6
5
4
3
2

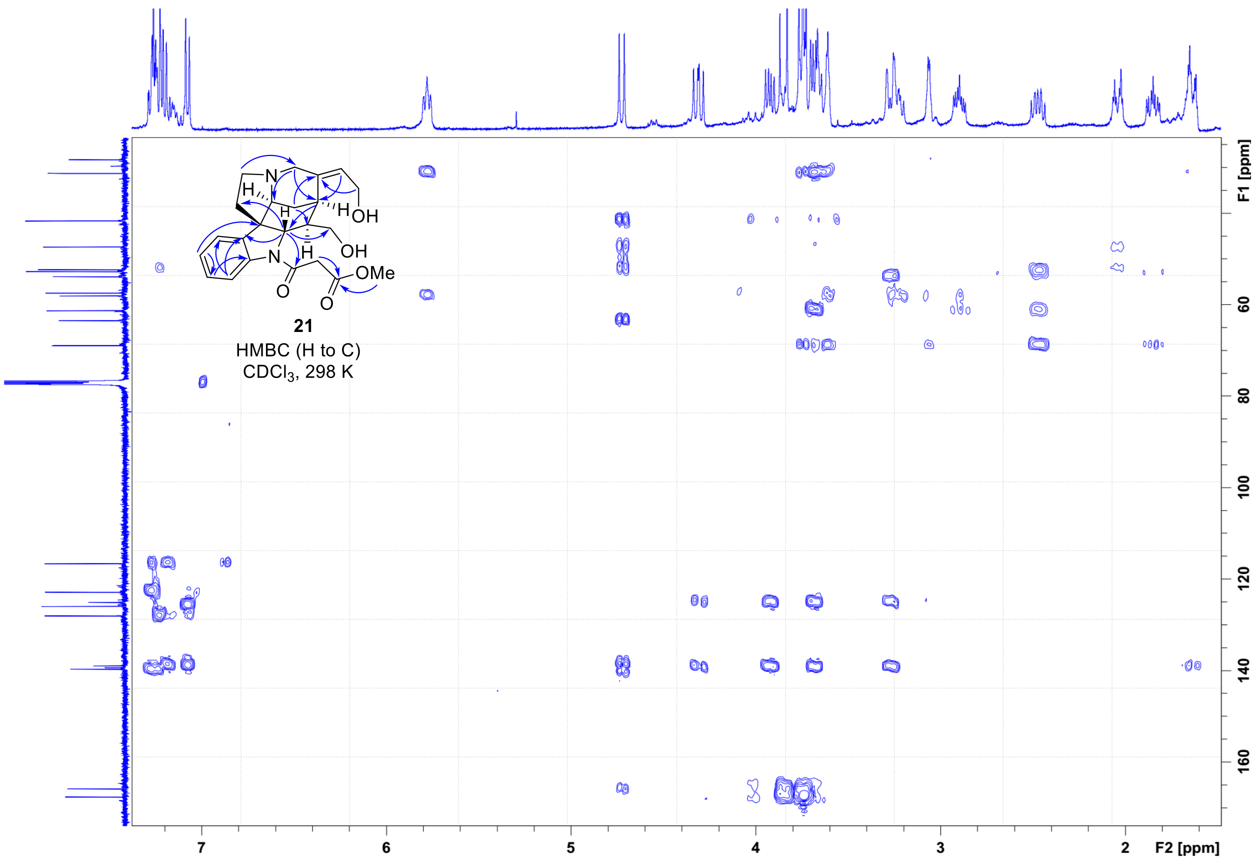
21
HMBC (H to C)
CDCl3, 298 K
F1 [ppm]
F2 [ppm]

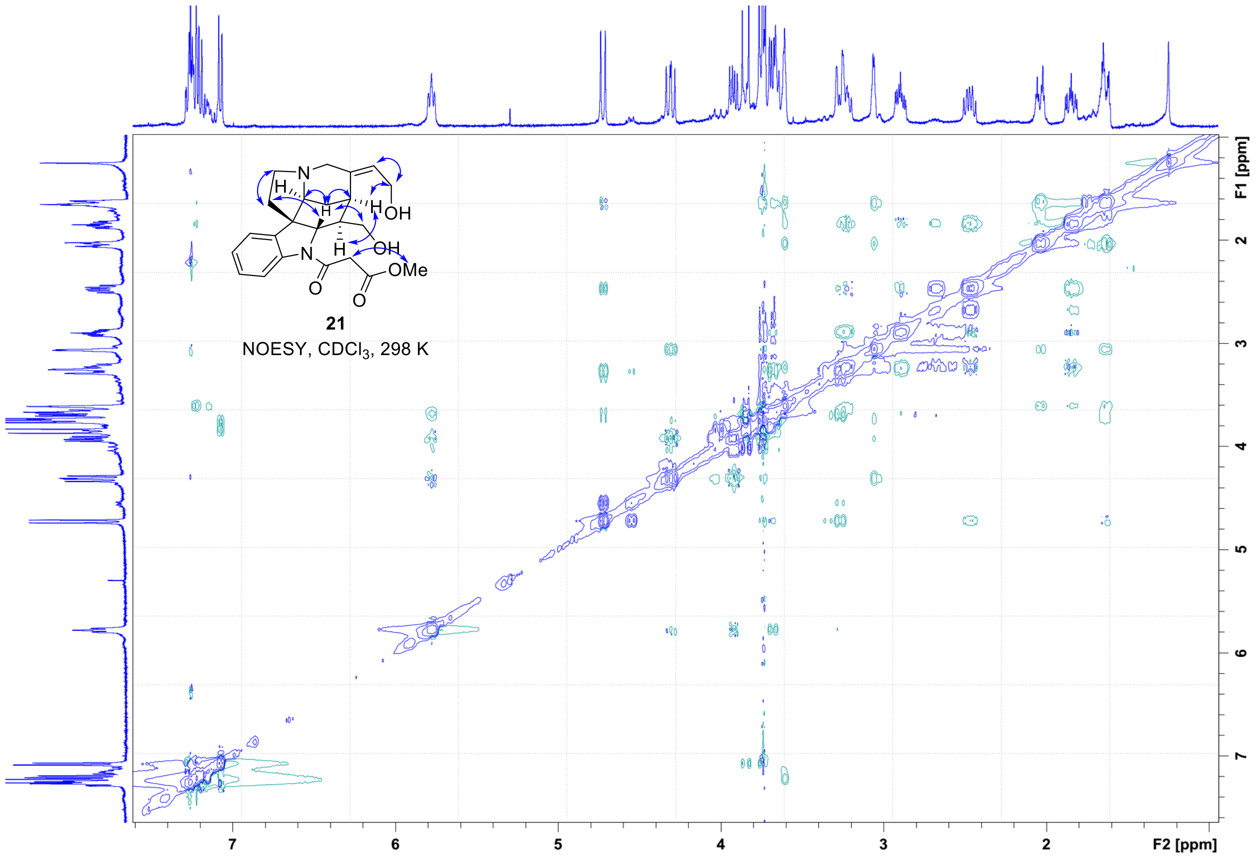
21
NOESY, CDCl3, 298 K
F1 [ppm]
F2 [ppm]

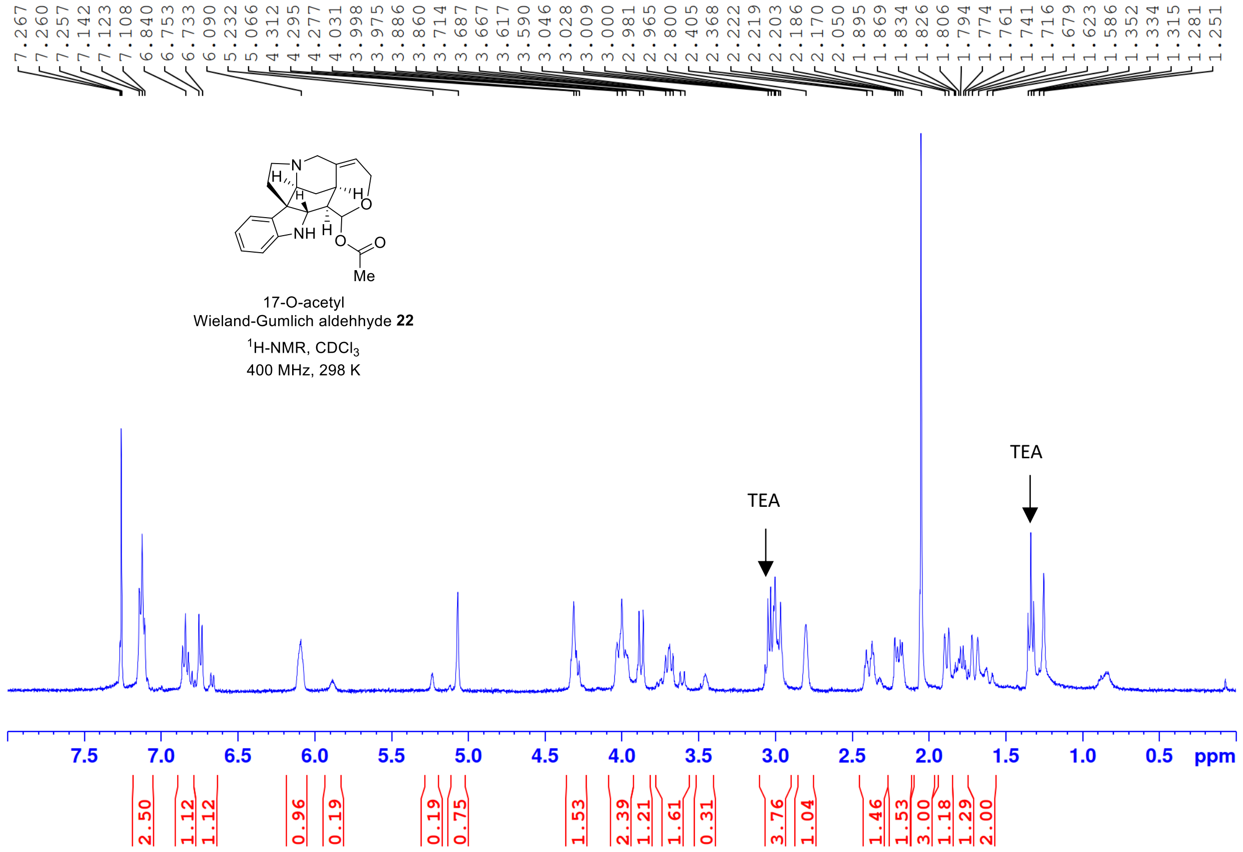
17-O-acetyl
Wieland-Gumlich aldehhyde **22**
^{1}H-NMR, $CDCl_3$
400 MHz, 298 K
TEA
TEA
ppm

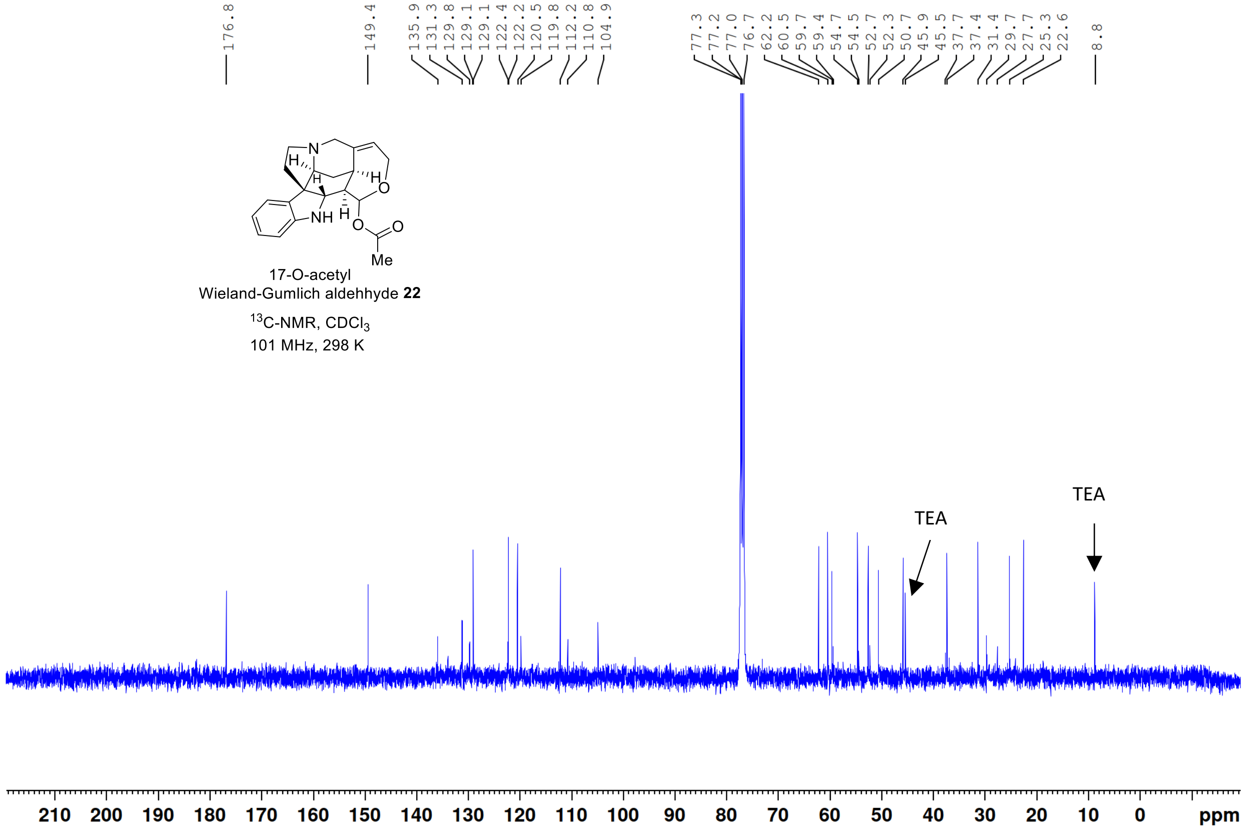
17-O-acetyl
Wieland-Gumlich aldehhyde **22**
^{13}C-NMR, $CDCl_3$
101 MHz, 298 K
TEA
TEA
ppm

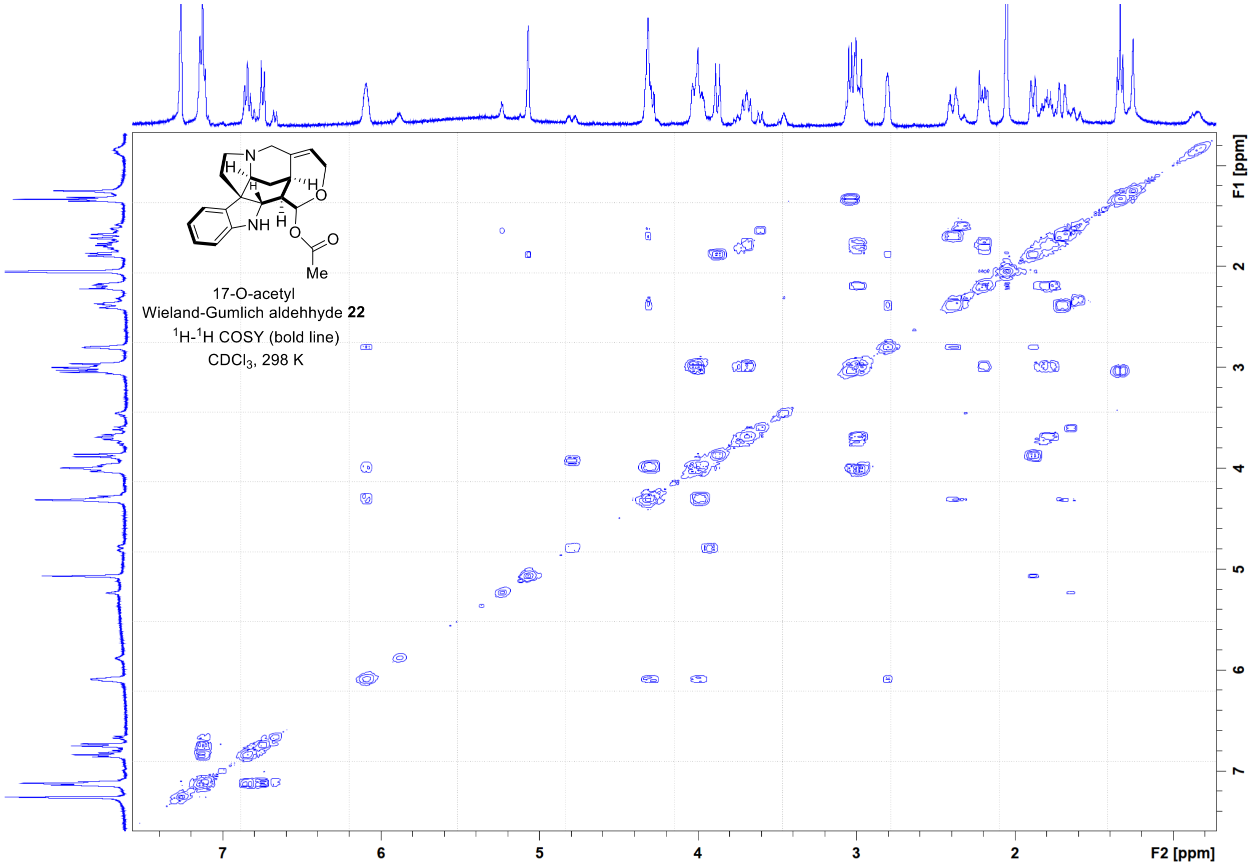
17-O-acetyl
Wieland-Gumlich aldehhyde **22**
^{1}H-^{1}H COSY (bold line)
$CDCl_3$, 298 K
F1 [ppm]
F2 [ppm]

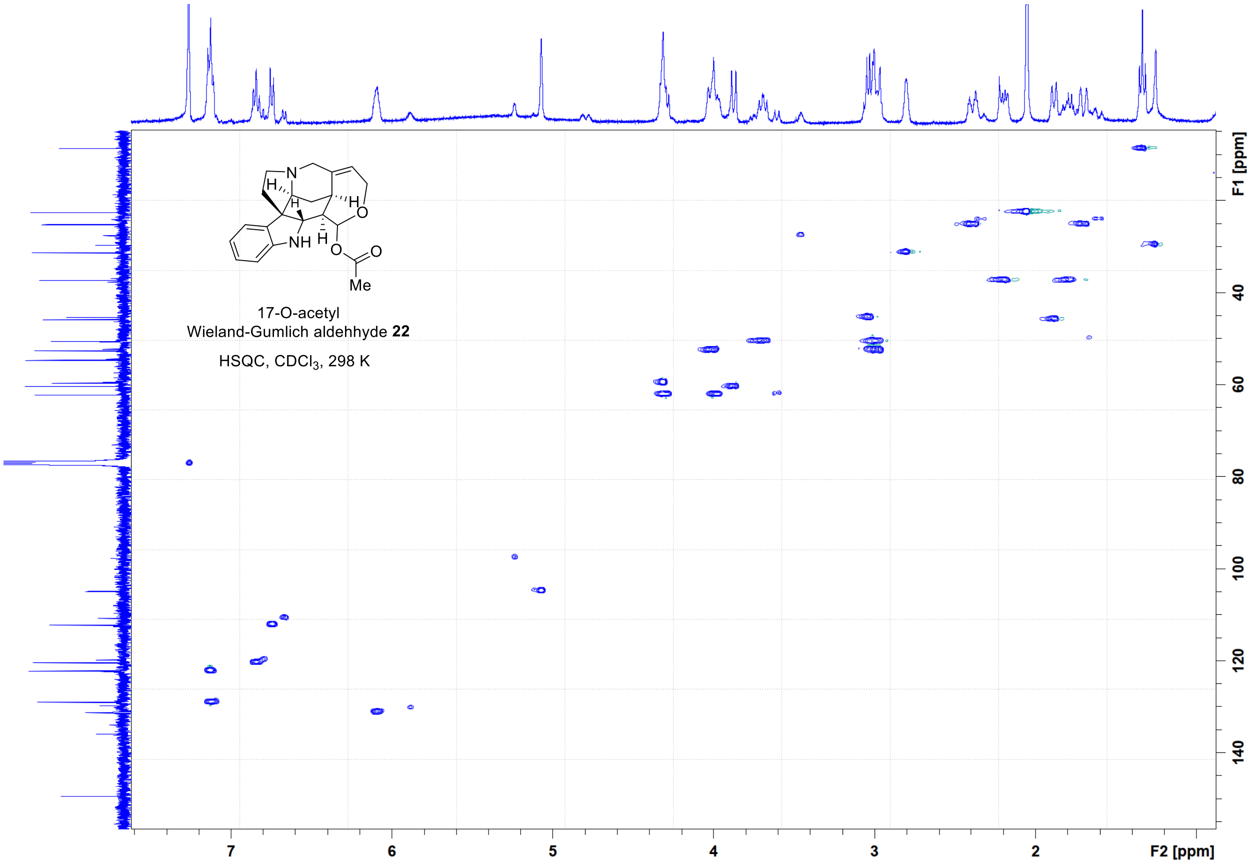
17-O-acetyl
Wieland-Gumlich aldehhyde **22**
HSQC, $CDCl_3$, 298 K
F1 [ppm]
F2 [ppm]

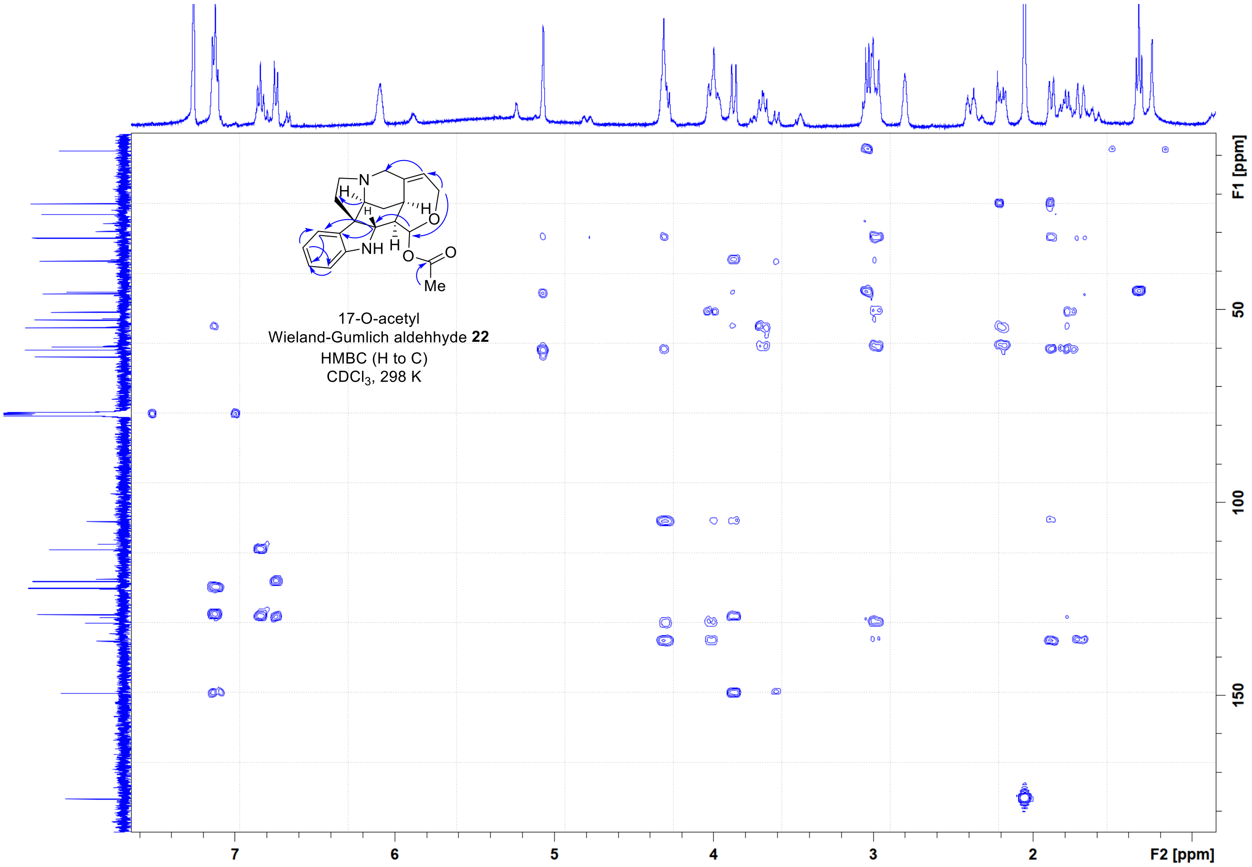
N
O
NH
H
O
O
Me
17-O-acetyl
Wieland-Gumlich aldehhyde **22**
HMBC (H to C)
$CDCl_3$, 298 K
F1 [ppm]
F2 [ppm]
50
100
150
7
6
5
4
3
2

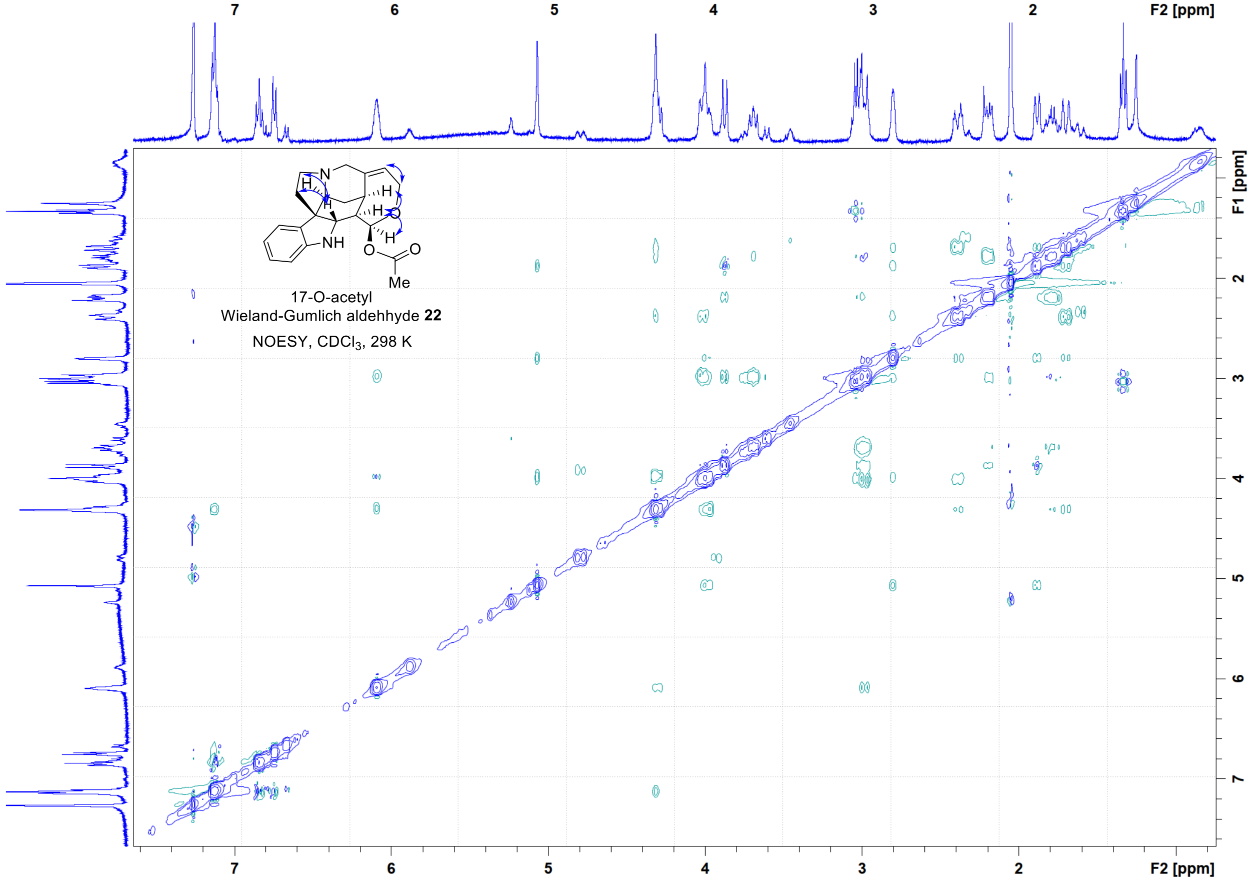
N
H
O
NH
O
O
Me
17-O-acetyl
Wieland-Gumlich aldehhyde **22**
NOESY, $CDCl_3$, 298 K
F1 [ppm]
F2 [ppm]
2
3
4
5
6
7

REFERENCES

1. *US EPA Strychnine.* EPA-738-F-96-033 (1996); https://www3.epa.gov/pesticides/chem_search/reg_actions/reregistration/fs_PC-076901_1-Jul-96.pdf.

2. Valdes, F. & Orrego, F. Strychnine inhibits the binding of glycine to rat brain-cortex membrane. *Nature* **226**, 761–762 (1970).

3. Huang, X., Chen, H., Michelsen, K., Schneider, S. & Shaffer, P. L. Crystal structure of human glycine receptor-α3 bound to antagonist strychnine. *Nature* **526**, 277–280 (2015).

4. Cannon, J. S. & Overman, L. E. Is there no end to the total syntheses of strychnine? Lessons learned in strategy and tactics in total synthesis. *Angew. Chem. Int. Ed*. **51**, 4288–4311 (2012).

5. Pelletier, P. J. & Caventou, J. B. Note sur un nouvel alcali. *Ann. Chim. Phys.* ***8,*** 323 (1818).

6. Openshaw, H. T. & Robinson, R. Constitution of strychnine and the biogenetic relationship of strychnine and quinine. *Nature* **157**, 438 (1946).

7. Robinson, R. The constitution of strychnine. *Experientia* **2**, 28-29, (1946).

8. Woodward, R. B., Brehm, W. J. & Nelson, A. L. The structure of strychnine. *J. Am. Chem. Soc.* **69**, 2250 (1947).

9. Robinson, R. *Progress in Organic Chemistry* (ed. J. W. Cook) (Butterworths, 1952).

10. Woodward, R. B. et al. The total synthesis of (−)-strychnine. *J. Am. Chem. Soc.* **76**, 4749-4751 (1954).

11. Woodward, R. B. Biogenesis of the Strychnos alkaloids. *Nature* **162**, 155-156 (1948).

12. Schlatter, C. et al. Über die Herkunft der C-Atome 22 und 23 im Strychnin. *Helv. Chim. Acta.* **49**. 1714-1175 (1966).

13. Schlatter, C., Waldner, E. E., Schmid, H., Maier, W. & Groger, D. Zur Biosynthese des Strychnins. 135. Mitteilung über Alkaloide, *Helv. Chim. Acta.* **52**, 776 (1969).

14. Heimberger, S. I. & Scott, I. A. Biosynthesis of Strychnine. *J. Chem. Soc. Chem. Commun.* 217-218 (1973).

15. Heimberg, S. I. The Biosynthesis of Strychnine. Thesis, Yale University (1973).

16. Maier, W. & Groger, D. Nichtverwertung von 5-Hydroxy-tryptophan für die Biosynthese von Strychnos-Alkaloiden. *Z. Naturforsch.* **25B**. 1192 (1970).

17. Baser, K. H. C. & Bisset, N. G. Alkaloids of Sri Lankan *Strychnos nux-vomica*. *Phytochemistry* **21**, 1423-1429 (1982).

18. Singh, H., Kapoor, V. K., Phillipson, J. D. & Bisset, N. G. Diaboline from *Strychnos potatorum*. *Phytochemistry* **14**, 587-588 (1975).

19. Tatsis, E. C. et al. A three enzyme system to generate the Strychnos alkaloid scaffold from a central biosynthetic intermediate. *Nat. Comm*. **8**, 316 (2017).

20. Mindrebo, J. T., Nartey, C. M., Seto, Y., Burkart, M. D. & Noel, J. P. Unveiling the functional diversity of the alpha/beta hydrolase superfamily in the plant kingdom. *Curr. Opin. Struct. Biol.* **41**, 233-246 (2016).

21. Dogru, E. et al. The gene encoding polyneuridine aldehyde esterase of monoterpenoid indole alkaloid biosynthesis in plants is an ortholog of the α/β hydrolase super family. *Eur. J. Biochem*. **267**, 1397-1406 (2000).

22. Nguyen, T.-D. & Dang, T.-T. T. Cytochrome P450 Enzymes as Key Drivers of Alkaloid Chemical Diversification in Plants. *Front. Plant Sci*. **12**, 682181 (2021).

23. Stavrinides, A. et al. Structural investigation of heteroyohimbine alkaloid synthesis reveals active site elements that control stereoselectivity. *Nat. Comm.* **7**, 12116 (2016).

24. Reed, J. & Osbourn, A. Engineering terpenoid production through transient expression in *Nicotiana benthamiana*. *Plant Cell Rep.* **37**, 1431–1441 (2018).

25. Lau, W. & Sattely, E. S. Six enzymes from mayapple that complete the biosynthetic pathway to the etoposide aglycone. *Science* **349**, 1224–1228 (2015).

26. Nett, R. S., Lau, W. & Sattely, E. S. Discovery and engineering of colchicine alkaloid biosynthesis. *Nature* **584**, 148–153 (2020).

27. D'Auria, J. C. Acyltransferases in plants: a good time to be BAHD. *Curr. Opin. Plant Biol*. **9**, 331–340 (2006).

28. Chen, H., Kim, H. U., Weng, H. & Browse, J. Malonyl-CoA synthetase, encoded by ACYL ACTIVATING ENZYME13, is essential for growth and development of Arabidopsis. *Plant Cell*. **23**, 2247–2262 (2011).

29. Wang, C. et al. Structural and Biochemical Insights Into Two BAHD Acyltransferases (*At*SHT and *At*SDT) Involved in Phenolamide Biosynthesis. *Front. Plant Sci*. **11**, 610118 (2021).

30. Manjasetty, B. A. et al. Structural basis for modification of flavonol and naphthol glucoconjugates by *Nicotiana tabacum* malonyltransferase (*Nt*MaT1). *Planta* **236**, 781–793 (2012).

31. Oefner, C., Schulz, H., D'Arcy, A. & Dale, G. E. Mapping the active site of *Escherichia coli* malonyl-CoA-acyl carrier protein transacylase (FabD) by protein crystallography, *Acta Cryst.* D**62**, 613-618 (2006).

32. Auldridge, M. E. et al. Emergent Decarboxylase Activity and Attenuation of α/β-Hydrolase Activity during the Evolution of Methylketone Biosynthesis in Tomato. *Plant Cell*. **24**, 1596-1607 (2012).

33. Austin, M. B. & Noel, J. P. The chalcone synthase superfamily of type III polyketide synthases. *Nat. Prod. Rep.* **20**, 79–110 (2003).

34. Levac, D., Murata, J., Kim, W. S. & de Luca, V. Application of carborundum abrasion for investigating the leaf epidermis: molecular cloning of Catharanthus roseus 16-hydroxytabersonine-16-O-methyltransferase. *Plant J.* **53**, 225-236 (2008).

35. Tzfadia, O. et al. CoExpNetViz: comparative co-expression networks construction and visualization tool. *Front. Plant Sci.* **6**, 1–7 (2016).

36. Shannon, P. et al. Cytoscape: a software environment for integrated models. *Genome Res.* **13**, 2498–2504 (2003).

37. Cárdenas, P. D. et al. Pathways to defense metabolites and evading fruit bitterness in genus *Solanum* evolved through 2-oxoglutarate-dependent dioxygenases. *Nat. Comm.* **10**, 5169 (2019).

38. Berrow, N. S. et al. A versatile ligation-independent cloning method suitable for high-throughput expression screening applications. *Nucleic Acids Res.* **35**, e45 (2007).

39. Topolewska, A., Czarnowska, K., Haliński, Ł. P. & Stepnowski, P. Evaluation of four derivatization methods for the analysis of fatty acids from green leafy vegetables by gas chromatography. *J Chromatogr B* **990**, 150–157 (2015)

40. Zuckerkandl, E. & Pauling, L. Evolutionary Divergence and Convergence in Proteins. *Evolving Genes and Proteins 97-166,* (1965)

41. Kumar, S. et al. MEGA X: Molecular evolutionary genetics analysis across computing platforms. *Mol. Biol. Evol.* 1547–1549 (2018).

42. Felsenstein, J. Confidence Limits on Phylogenies: An Approach Using the Bootstrap. *Evolution (N. Y).* 783–791 (1985).

43. Letunic, I. & Bork, P. Interactive tree of life (iTOL) v3: an online tool for the display and annotation of phylogenetic and other trees. *Nucleic Acids Res.* **44**, W242–W245 (2016).

44. Waterhouse, A. et al. SWISS-MODEL: Homology modelling of protein structures and complexes. *Nucleic Acids Res.* W296–W303 (2018).

45. Studer, G. et al. QMEANDisCo—distance constraints applied on model quality estimation. *Bioinformatics*, **36**, 1765–1771 (2020).

46. Trott, O. & Olson, A. J. AutoDock Vina: Improving the Speed and Accuracy of Docking with a New Scoring Function, Efficient Optimization, and Multithreading. *Journal of Computational Chemistry*, **31**, 455–461 (2010).

47. Jumper, J. et al. Highly accurate protein structure prediction with AlphaFold. *Nature*, **596**, 583–589 (2021).

48. Mirdita, M. et al. (2021). ColabFold - Making protein folding accessible to all. BioRxiv. https://doi.org/https://doi.org/10.1101/2021.08.15.456425.

49. Mariani, V., Biasini, M., Barbato, A. & Schwede, T. lDDT: A local superposition-free score for comparing protein structures and models using distance difference tests. *Bioinformatics*, **29**, 2722–2728 (2013).

50. Seeman, J. I. & Tantillo, D. J. History of Chemistry From Decades to Minutes: Steps Toward the Structure of Strychnine 1910-1948 and the Application of Today's Technology. *Angew. Chem. Int. Chem.* **59**, 10702–10721 (2020).

51. Fishbein, M. et al. Evolution on the backbone: *Apocynaceae* phylogenomics and new perspectives on growth forms, flowers, and fruits. American Journal of Botany **105**, 495–513 (2018).

52. Jarret, M. et al. Bioinspired Oxidative Cyclization of the Geissoschizine Skeleton for the Total Synthesis of (−)-17-nor-Excelsinidine. *Angew. Chem. Int. Chem.* **57**, 12294–12298 (2018).

52. Hymon, J. R. et al. 160. Die Chemie des WIELAND-GUMLICH-Aldehyds und seiner Derivate [I]. *Helv. Chim. Acta*. **52**, 1564-1602. (1969).

54. Zlotos, D. P. et al. Probing the Pharmacophore for Allosteric Ligands of Muscarinic M 2 Receptors: SAR and QSAR Studies in a Series of Bisquaternary Salts of Caracurine V and Related Ring Systems. *J. Med. Chem.* **47**, 3561-3571 (2004).

55. Fritz, H., Besch, E. & Wieland, Th. Synthese von C-Curarin-III (C-Fluorocurarin) aus Strychnin Über die Alkaloide aus Calebassen-Curare XVI. *Angew. Chem.* **71**, 126 (1959).

56. Fritz, H., Besch, E. & Wieland, Th. Über die Alkaloide aus Calebassencurare, XIX. Synthese von Nor-C-curarin-III und C-Curarin-III Aus Strychnin. *Just. Lieb. Ann. Chem*. **663**, 150–156 (1963).

57. Grdinic, M., Nelson, D. A. & Boekelheide, V. Calabash Curare Alkaloids. Specific Deuterium Labeling and Nuclear Magnetic Resonance Studies. *J. Am. Chem. Soc.* **86**. 3357-3363 (1964).

58. Deyrup, J. A., Schmid, H. & Karrer, P. Zur Chemie des WIELAND-GUMLICH-Aldehyds. 55. Mitteilung über Curar-Alkaloide. *Helv. Chim. Act.* **262** .2266-2272 (1962).

59. Zhao, S. et al. Synthesis of bis-strychnos alkaloids (–)-sungucine, (–)-isosungucine, and (–)-strychnogucine B from (–)-strychnine. *J. Braz. Chem. Soc.* **30**, 436–453 (2019).

60. Tedeschi, E., Dukler, S., Pfeffer, P. & Lavie, D. Studies on strychnine derivatives and conversion into brucine. *Tetrehedron* **24**, 4573-4580 (1968).

61. Verpoorte, R. et al. Carbon-13 NMR spectroscopy of some *Strychnos* alkaloids. Part 2. *Org. Magn. Res.* **22**, 345–348 (1984).
