# Supplementary material for: Biosynthesis of strychnine
Source: Nature. 2022 Jul 6;607(7919):617–22. doi: 10.1038/s41586-022-04950-4 (PMC9300463; doi:10.1038/s41586-022-04950-4)
Supplement: Supplementary file 2 — Reporting Summary [file 41586_2022_4950_MOESM2_ESM.pdf]

nature portfolio

Corresponding author(s): Sarah E. O'Connor

Last updated by author(s): May 24, 2022

Reporting Summary

Nature Portfolio wishes to improve the reproducibility of the work that we publish. This form provides structure for consistency and transparency in reporting. For further information on Nature Portfolio policies, see our Editorial Policies and the Editorial Policy Checklist.

Statistics

For all statistical analyses, confirm that the following items are present in the figure legend, table legend, main text, or Methods section.

n/a	Confirmed	
☐	☒	The exact sample size (*n*) for each experimental group/condition, given as a discrete number and unit of measurement
☐	☒	A statement on whether measurements were taken from distinct samples or whether the same sample was measured repeatedly
☐	☒	The statistical test(s) used AND whether they are one- or two-sided<br>*Only common tests should be described solely by name; describe more complex techniques in the Methods section.*
☒	☐	A description of all covariates tested
☒	☐	A description of any assumptions or corrections, such as tests of normality and adjustment for multiple comparisons
☐	☒	A full description of the statistical parameters including central tendency (e.g. means) or other basic estimates (e.g. regression coefficient) AND variation (e.g. standard deviation) or associated estimates of uncertainty (e.g. confidence intervals)
☐	☒	For null hypothesis testing, the test statistic (e.g. *F*, *t*, *r*) with confidence intervals, effect sizes, degrees of freedom and *P* value noted<br>*Give P values as exact values whenever suitable.*
☒	☐	For Bayesian analysis, information on the choice of priors and Markov chain Monte Carlo settings
☒	☐	For hierarchical and complex designs, identification of the appropriate level for tests and full reporting of outcomes
☒	☐	Estimates of effect sizes (e.g. Cohen's *d*, Pearson's *r*), indicating how they were calculated

Our web collection on statistics for biologists contains articles on many of the points above.

Software and code

Policy information about availability of computer code

Data collection	All presented data have been acquired using existing and routinely used software. LC-MS data was collected by Bruker otofControl 5.2.109/ Hystar 5.1.5.1. NMR data was collected by Bruker TopSpin 3.6.1. Confocal microscopy images were collected by ZEN black 2.1 v.14.0.18.201 (Zeiss, Oberkochen, Germany).
Data analysis	The phylogenetic tree was constructed in MEGAX v10.2.0. and visualized with iTOL. Protein homology models were built using the Swiss-Model server and visualized with PyMOL. Molecular docking was performed using AutoDock Vina. The software used for confocal microscopy analysis was ZEN black 2.1 v.14.0.18.201 (Zeiss, Oberkochen, Germany). NMR data were processed with Bruker TopSpin ver. 3.6.1. LC-MS data was processed with Bruker DataAnalysis 5.0 and MetaboScape 4.0. Chemical structures were generated in ChemDraw Professional 17.1. Kinetics data was analysed by GraphPad Prism 8.0.2. Heatmaps were generated by Morpheus: (https://software.broadinstitute.org/ morpheus). Trinity v.2.6.6 was used to perform the transcriptome assembly. CORSET v.4.6 software was used to remove the redundance from Trinity results. Gene expression levels were estimated by RSEM v.1.2.28 and differential expression analysis using DESeq2 v.1.26.0. Coexpression analysis was done using CoExpNetViz software (http://bioinformatics.psb.ugent.be/webtools/coexpr/) and visualized with the Cytoscape v.3.9.0.

For manuscripts utilizing custom algorithms or software that are central to the research but not yet described in published literature, software must be made available to editors and reviewers. We strongly encourage code deposition in a community repository (e.g. GitHub). See the Nature Portfolio guidelines for submitting code & software for further information.

Data

Policy information about availability of data

All manuscripts must include a data availability statement. This statement should provide the following information, where applicable:

- Accession codes, unique identifiers, or web links for publicly available datasets
- A description of any restrictions on data availability
- For clinical datasets or third party data, please ensure that the statement adheres to our policy

There are no restrictions on the availability of data. All reported data within this study are available via database or by request. The sequence of genes characterized in this article are deposited in National Center for Biotechnology (NCBI) GenBank under the accession numbers: SnvGO (OM304290), SnvNS1(OM304291), SnvNS2 (OM304292), SnvNO (OM304293), SnvWS (OM304294), SnvAT (OM304295), Snv10H (OM304296), SnvOMT (OM304297), Snv11H (OM304298), SpGO (OM304299), SpNS1 (OM304300), SpNS2 (OM304301), SpNO (OM304302), SpWS (OM304303), SpAT (OM304304). TThe raw reads from the RNA-seq profiling analysis of Strychnos nux-vomica and Strychnos Sp. are deposited in the NCBI Sequence Read Archive (SRA) database under the BioProject accession PRJNA825510 and PRJNA826736, respectively.

Field-specific reporting

Please select the one below that is the best fit for your research. If you are not sure, read the appropriate sections before making your selection.

☒ Life sciences ☐ Behavioural & social sciences ☐ Ecological, evolutionary & environmental sciences

For a reference copy of the document with all sections, see nature.com/documents/nr-reporting-summary-flat.pdf

Life sciences study design

All studies must disclose on these points even when the disclosure is negative.

Sample size	Prior determination of sample size was not a consideration for our data. Replicates of 3 were chosen for heterologous expression experiments in Nicotiana benthamiana.
Data exclusions	No data were excluded from the analyses.
Replication	The majority of the data presented in this study is representative of three experiments done in different days. All attempts at replication were successful.
Randomization	For heterologous expression in Nicotiana benthamiana leaves, each experiment was tested 3 times. Three biological replicates are from different Nicotiana benthamiana plants. Each of these plants would contain one replicate from each different condition. The second pair of fully expanded leaves (counting from the apical meristem side) in each plant were used for experiment.
Blinding	Blinding was not relevant for our study. Functional characterization of enzymes or genes required the insight of researchers about the tested samples.

Reporting for specific materials, systems and methods

We require information from authors about some types of materials, experimental systems and methods used in many studies. Here, indicate whether each material, system or method listed is relevant to your study. If you are not sure if a list item applies to your research, read the appropriate section before selecting a response.

Materials & experimental systems

n/a	Involved in the study
☒	☐ Antibodies
☒	☐ Eukaryotic cell lines
☒	☐ Palaeontology and archaeology
☒	☐ Animals and other organisms
☒	☐ Human research participants
☒	☐ Clinical data
☒	☐ Dual use research of concern

Methods

n/a	Involved in the study
☒	☐ ChIP-seq
☒	☐ Flow cytometry
☒	☐ MRI-based neuroimaging

March 2021
